# Supplementary material for: Loss of p300 in proximal tubular cells reduces renal fibrosis and endothelial-mesenchymal transition
Source: EMBO Mol Med. 2025 Jul 1;17(7):1575–98. doi: 10.1038/s44321-025-00243-1 (PMC12254316; doi:10.1038/s44321-025-00243-1)
Supplement: Supplementary file 1 — Appendix [file 44321_2025_243_MOESM1_ESM.pdf]

<Appendix>

Loss of p300 in proximal tubular cells reduces renal fibrosis and Endothelial-Mesenchymal Transition

Hyunsik Kim[1], Soo-Yeon Park[1], Soo Yeon Lee[1], Jae-Hwan Kwon[1], Seunghee Byun[1], Byounghwi Ko[4], Jung-Yoon Yoo[2], Beom Seok Kim[3,*], Beom Jin Lim[4,*] and Ho-Geun Yoon[1,*]

[1]Department of Biochemistry and Molecular Biology, Severance Medical Research Institute, Brain Korea 21 PLUS Project for Medical Sciences, Yonsei University College of Medicine, Seoul, Korea 03722;

[2]Department of Biomedical Laboratory Science, Yonsei University MIRAE Campus, Wonju 26493, Korea.

[3]Department of Pathology, Yonsei University College of Medicine, Seoul, Korea 03722;

[4]Department of Internal Medicine, Yonsei University College of Medicine, Seoul, Korea 03722;

Appendix Table S1. Clinical characteristics••Pg 4
Appendix Table S2. Comparison between FSGS and MCD patients••••••••••••••••••••••••••••Pg 4
Appendix Table S3. Sequence of mutagenesis primers•••Pg 5
Appendix Table S4. Antibody information•••Pg 6
Appendix Table S5. Sequence of qPCR primers••Pg 7
Appendix Figure S1. Elevation of p300 in mouse kidney fibrosis models. •••••••••••••••••••Pg 9
Appendix Figure S2. Expression of PCAF and GCN5 in UUO-induced mouse kidney fibrosis models. ••Pg 10
Appendix Figure S3. p300 expression is up-regulated in PTCs during renal fibrosis. ••••••Pg 11
Appendix Figure S4. PTC-specific elevation of p300 in UUO and UNx-STZ-induced fibrosis models. ••Pg 12
Appendix Figure S5. Generation of proximal tubular cell-specific p300 knock-out mice. Pg 13
Appendix Figure S6. Proximal tubular cell-specific p300 knockout attenuates fibrosis progression in UUO-induced renal fibrosis mouse model. •••••••••••••••••••••••••••••••••••••Pg 14
Appendix Figure S7. Evaluation of fibrosis and renal function in p300 cKO Adriamycin-induced fibrosis mouse models. ••Pg 15
Appendix Figure S8. Proximal tubular cell p300 is elevated at the protein level, not the mRNA level, in response to TGFβ signaling. ••Pg 16
Appendix Figure S9. Phosphorylation of p300 at Serine 1834 via AKT signaling increases protein stabilization in response to TGFβ signaling. •••Pg 17
Appendix Figure S10. PPM1K binds to and dephosphorylates Serine 1834 of p300. •••••••Pg 18
Appendix Figure S11. TGFβ stimulation induces PPM1K dissociation from p300. ••••••••Pg 19
Appendix Figure S12. Inverse correlation of p300 and PPM1K in CKD patients and the UUO-induced mouse fibrosis model. ••Pg 20
Appendix Figure S13. PPM1K regulates fibrosis-related protein and gene expression in HK2 cells treated with TGFβ. •••Pg 21
Appendix Figure S14. Confirmation of intra-renal Adenovirus delivery. •••••••••••••••••••Pg 22
Appendix Figure S15. Overexpression of wild-type PPM1K significantly decreases both phosphorylation and stability of p300 in the UUO-induced mouse model. •••••••••••••••••••Pg 23
Appendix Figure S16. p300 in proximal tubular cells regulates mesenchymal transition-related gene expression. ••Pg 24
Appendix Figure S17. Increase in endothelial-to-mesenchymal transition in CKD patients. ••Pg 25

Appendix Figure S18. p300 binds to the promoter regions of FSTL1, FSCN1, and POSTN during renal fibrosis. ••Pg 26
Appendix Figure S19. Increased expression levels of FSTL1, FSCN1, and POSTN in CKD patients. ••Pg 27
Appendix Figure S20. p300 regulates the expression of FSTL1, FSCN1, and POSTN in kidney proximal tubular cells. •••Pg 28
Appendix Figure S21. PTC-specific p300 mediates endothelial-to-mesenchymal transition. ••Pg 29
Appendix Figure S22. Renal microvascular structures in WT and p300 cKO UUO-induced mouse fibrosis model. ••Pg 30
Appendix Figure S23. p300-specific inhibitors reduce the expression of mesenchymal transition-related genes and suppress EndMT progression. ••••••••••••••••••••••••••••••••••••••Pg 31

Appendix Table S1. Clinical characteristics

Patients no.	Diagnosis	sex	age	Fibrosis	BUN	Creatinine	Uric acid	UPCR(g/gCr)	UACR(mg/gCr)
1.	MCD	M	76	-	45.9	2.14	3.8	11.67	13465.63
2.	MCD	M	69	-	27.7	1.28	6.2	10.94	7078.51
3.	MCD	M	50	-	15.8	1.19	3.9	1.66	1116.74
4.	MCD	M	31	-	44.9	1.33	10.2	9.15	7056.98
5.	MCD	M	28	-	16.1	0.72	5.6	2.04	1495.32
6.	MCD	M	25	-	34.2	1.09	8	6.88	5034.44
7.	MCD	F	28	-	7.5	0.45	2.6	4.48	3579.78
8.	MCD	F	74	-	28.5	0.61	4.8	25.71	20277.97
9.	MCD	F	28	-	28	0.58	8.1	22.74	15333.5
10.	MCD	F	46	-	14.6	0.64	6.4	4.09	3148.94
11.	MCD	F	51	-	10.5	0.54	5.3	0.84	485.42
12.	FSGS	M	60	+	18.2	0.58	4.1	7.37	4933.74
13.	FSGS	M	29	+	53.4	2.45	4.8	0.13	28.05
14.	FSGS	M	68	±	18.7	0.89	5	0.15	87.35
15.	FSGS	F	39	+	10.2	0.67	5.9	1.9	1536.79
16.	FSGS	F	30	±	9.8	0.54	5.3	11.88	8369.33
17.	FSGS	M	20	±	12	0.86	7.1	3.65	3016.84
18.	FSGS	M	39	±	14.4	1.11	7.4	1.14	895.36
19.	FSGS	F	41	+	16.7	1.15	6.3	0.33	228.79
20.	FSGS	F	43	++	45.9	3.03	6.2	6.07	5162.25
21.	FSGS	M	76	++	23.2	1.86	7.6	8.54	5872.36
22.	FSGS	M	66	±	12.6	0.86	6	0.34	223.75

Appendix Table S2. Comparison between FSGS and MCD patients

Diagnosis	MCD	FSGS	*P* value
Age	46±5.9	46.5±5.5	ns
Sex(male,%)	54.5	63.6	ns
BUN	24.9±4.0	21.4±4.4	ns
Creatinine	1±0.2	1.3±0.2	ns
Uric_acid	5.9±0.7	6±0.3	*(0.0407)
UPCR(g/gCr)	9.1±2.5	3.8±1.2	*(0.034)
UACR(mg/gCr)	7097.6±1966.1	2759.5±874.5	*(0.0172)

Appendix Table S3. Sequence of mutagenesis primers

Gene name<br>(Forward: F; Reverse: R)	Sequence 5'-3'
P300 S1834A_F	CGCAGGAGGATGGCCGCCATGCAGCGGACTGG
P300 S1834A_R	CCAGTCCGCTGCATGGCGGCCATCCTCCTGCG
PPM1K N94K_F	ATCAGCTTGGAAAAAGTGGGGTGCG
PPM1K N94K_R	CGCACCCCACTTTTTCCAAGCTGAT

Appendix Table S4. Antibody information

	Antibody information (aplication, manufactur, product no.)
Anti-p300	IHC,IF(tissue), Cell signalling Technology, 86377s
Anti-p300	IF(cell),PLA,Western blot, Santacruz biotech, SC48343
Anti-PPM1K	IHC(human tissue), Invitrogen, PA5-21658
Anti-PPM1K	IHC,IF(mouse tisseue),Western blot,PLA, Abcam, ab135286
Anti-AQP1	IHC, IF, Western blot, Merck millipore ,ab2219
Anti-AQP2	IHC, Cell signalling Technology, 3487s
Anti-WT1	IHC, Abcam, ab89901
Anti-CD31	IHC,IF, Abcam, ab28364
Anti-p-p300(S1834)	IHC,Western blot, Invitrogen, PA5-64531
Anti-AKT	Western blot, Cell signalling Technology, 4691s
Anti-p-AKT(S473)	Western blot, Cell signalling Technology, 4060s
Anti-COL1A	IF(cell), Western blot, Santacruz biotech, SC59772
Anti-CTGF	Western blot, Santacruz biotech, SC365970
Anti-FSTL1	IHC,IF,Western blot, Abclonal, A15789
Anti-FSCN1	IHC,IF,Western blot, Abclonal, A9566
Anti-POSTN	IHC,IF,Western blot, Cell signalling Technology, 20302s
Anti-VIMENTIN	IF, Santacruz biotech, SC32322
Anti-KIM1	IHC, Invitrogen, PA1-86790
Anti-ALBUMIN	IHC, Cell signalling Technology, 4929s
Anti-ACTA2	IHC,IF, Western blot, Abcam, ab5694
Anti-ZO1	IF, Cell signalling Technology, 8193p
Anti-p53	Westernblot, Santacruz biotech, SC6243
LTL	IF,Cell sorting, Vector, FL-1321

Appendix Table S5. Sequence of qPCR primers

Gene name (Forward: F; Reverse: R)	Sequence 5'-3'
human_COL1A1_F	TCCTGCTGGTGAGAAAGGAT
human_COL1A1_R	TCCAGCAATACCCTGAGGTC
human_COL3A1_F	TGGTCTGCAAGGAATGCCTGGA
human_COL3A1_R	TCTTTCCCTGGGACACCATCAG
human_CTGF_F	CAAGGGCCTCTTCTGTGACT
human_CTGF_R	ACGTGCACTGGTACTTGCAG
human_TNC_F	CAGAAGCCGAACCGAAGTT
human_TNC_R	TTCATCAGCTGTCCAGGACAGA
human_ACTA2(αSMA)	CTGGCATCGTGCTGGACTCT
human_ACTA2(αSMA)	GATCTCGGCCAGCCAGATC
human_EP300_F	TTGTGAAGAGCCCCATGGAT
human_EP300_R	GCTTTGCATCACTGGGTCAA
human_FN1_F	AAGACCAGCAGAGGCATAAGG
human_FN1_R	TGTAGGGGTCAAAGCACGAG
human_GAPDH_F	TGACCGGTGCCATGGAATTTG
human_GAPDH_R	GTCGGAGTCAACGGATTTGG
human_Vimentin_F	GGAAGAGAACTTTGCCGTTGAA
human_Vimentin_R	GTGACGAGCCATTTCCTCCTT
human_PAI1_F	CCCCACTTCTTCAGGCTGTT
human_PAI1_R	GCCGTTGAAGTAGAGGGCAT
human_tie2_F	GGTCAAGCAACCCAGCCTTTTC
human_tie2_R	CAGGTCATTCCAGCAGAGCCAA
human_PECAM1(CD31)_F	AAGTGGAGTCCAGCCGCATATC
human_PECAM1(CD31)_R	ATGGAGCAGGACAGGTTCAGTC

mouse_COL1A1_F	ATGGATTCCCGTTCGAGTACG
mouse_COL1A1_R	TCAGCTGGATAGCGACATCG
mouse_COL3A1_F	CTAAAATTCTGCCACCCCGAA
mouse_COL3A1_R	AGGATCAACCCAGTATTCTCCACTC
mouse_ACTA2(αSMA)	GTGACTCACAACGTGCCTATC
mouse_ACTA2(αSMA)	CTCGGCCAGTAGTCACGAAGG
mouse_FN1_F	AAGACCATACCTGCCGAATG
mouse_FN1_R	GAACATGACCGATTTGGACC
mouse_EP300_F	CTGTGAACCAACATGAGTGCTAGTCC
mouse_EP300_R	TGAGCTGCTGTTGGCAAAGG
mouse_GAPDH_F	CGACTTCAACAGCAACTCCCACTCTTCC
mouse_GAPDH_R	TGGGTGGTCCAGGGTTTCTTACTCCTT
mouse_Vimentin_F	CCCTCACCTGTGAAGTGGAT
mouse_Vimentin_R	TCCAGCAGCTTCCTGTAGGT
mouse_PAI1_F	AGGGTTGCACTAAACATGTCAG
mouse_PAI1_R	GACACCCTCAGCATGTTCATC
mouse_FSP1_F	TGAGCAACTTGGACAGCAACA
mouse_FSP1_R	TTCCGGGGTTCCTTATCTGGG
mouse_PECAM1(CD31)_F	CCAAAGCCAGTAGCATCATGGTC
mouse_PECAM1(CD31)_R	GGATGGTGAAGTTGGCTACAGG
mouse_tie2_F	GAACTGAGGACGCTTCCACATTC
mouse_tie2_R	TCAGAAACGCCAACAGCACGGT
mouse_CD144_F	GAACGAGGACAGCAACTTCACC
mouse_CD144_R	GTTAGCGTGCTGGTTCCAGTCA

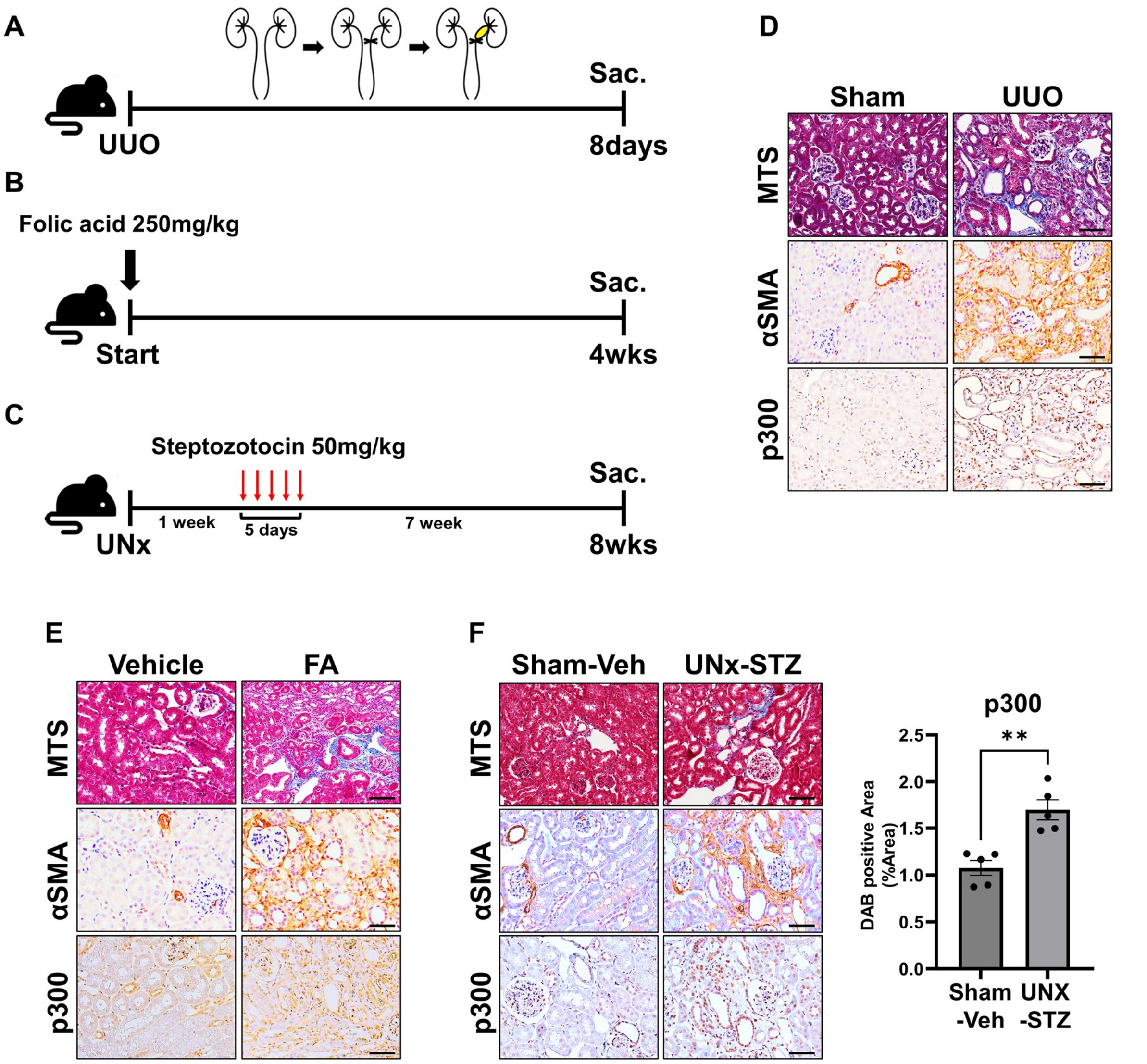

Appendix Figure S1. Elevation of p300 in mouse kidney fibrosis models. (A-C) Schematic images of murine fibrosis models (UUO, UNx-STZ, Folic acid). (D) Representative image of Masson trichrome staining (MTS) and p300, αSMA immunohistochemistry (IHC) in mouse kidney tissue from UUO-induced mouse fibrosis models. bar=100μm. (E) Representative image of Masson trichrome staining (MTS) and p300, αSMA immunohistochemistry (IHC) in mouse kidney tissue from FA-induced mouse fibrosis models. bar=100μm. (F) Representative image of Masson trichrome staining (MTS) and p300, αSMA immunohistochemistry (IHC) in mouse kidney tissue from UNx-STZ-induced mouse fibrosis model. The graph represents quantification of histological staining images (n=5 per group). bar=100μm. Data are presented as mean ± SEM, **P < 0.01 by t-test.

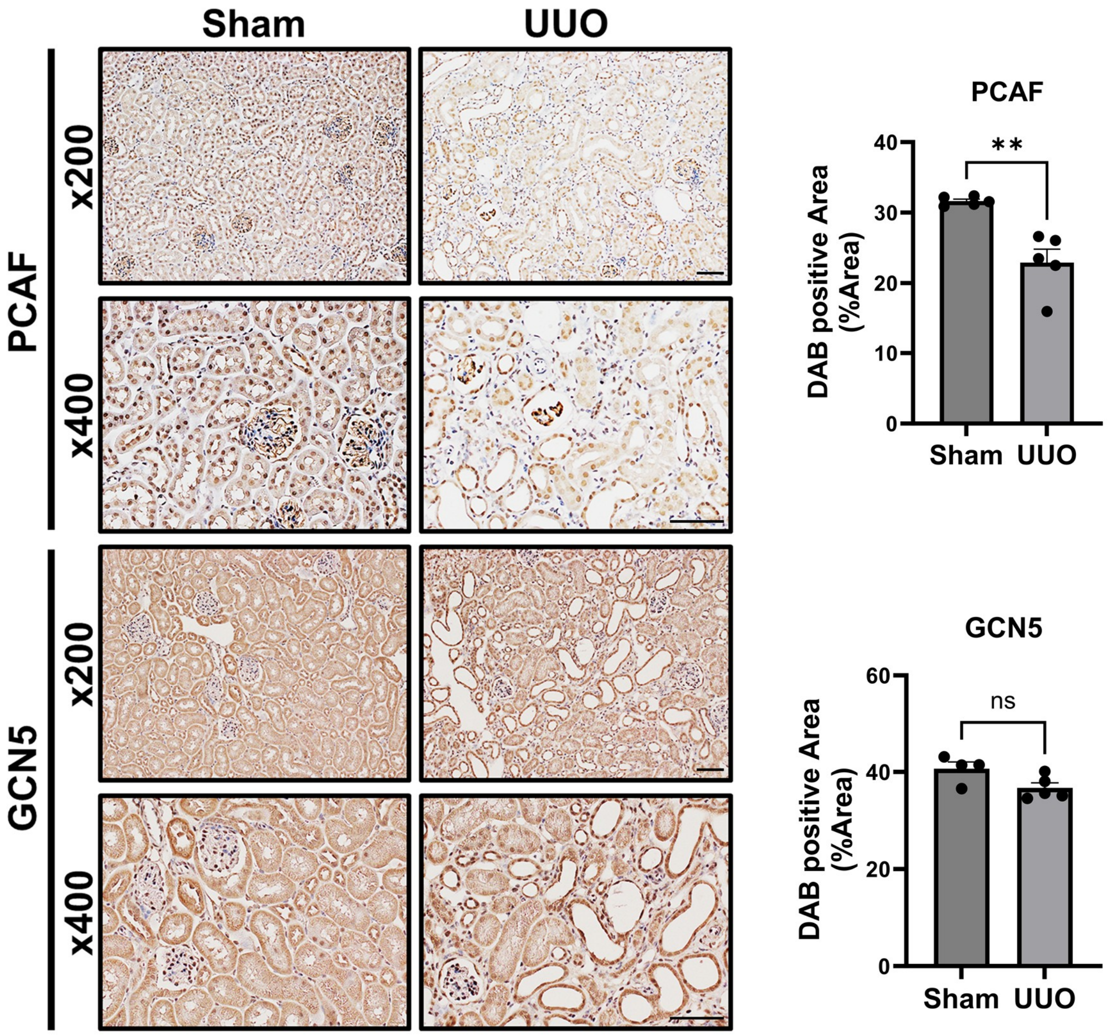

Appendix Figure S2. Expression of PCAF and GCN5 in UUO-induced mouse kidney fibrosis models. Representative image of PCAF and GCN5 immunohistochemistry (IHC) in kidney tissue from UUO-induced mouse fibrosis models. The graph represents quantification of histological staining images (n=5 per group). bar=100μm. Data are presented as mean ± SEM, **P < 0.01 by t-test.

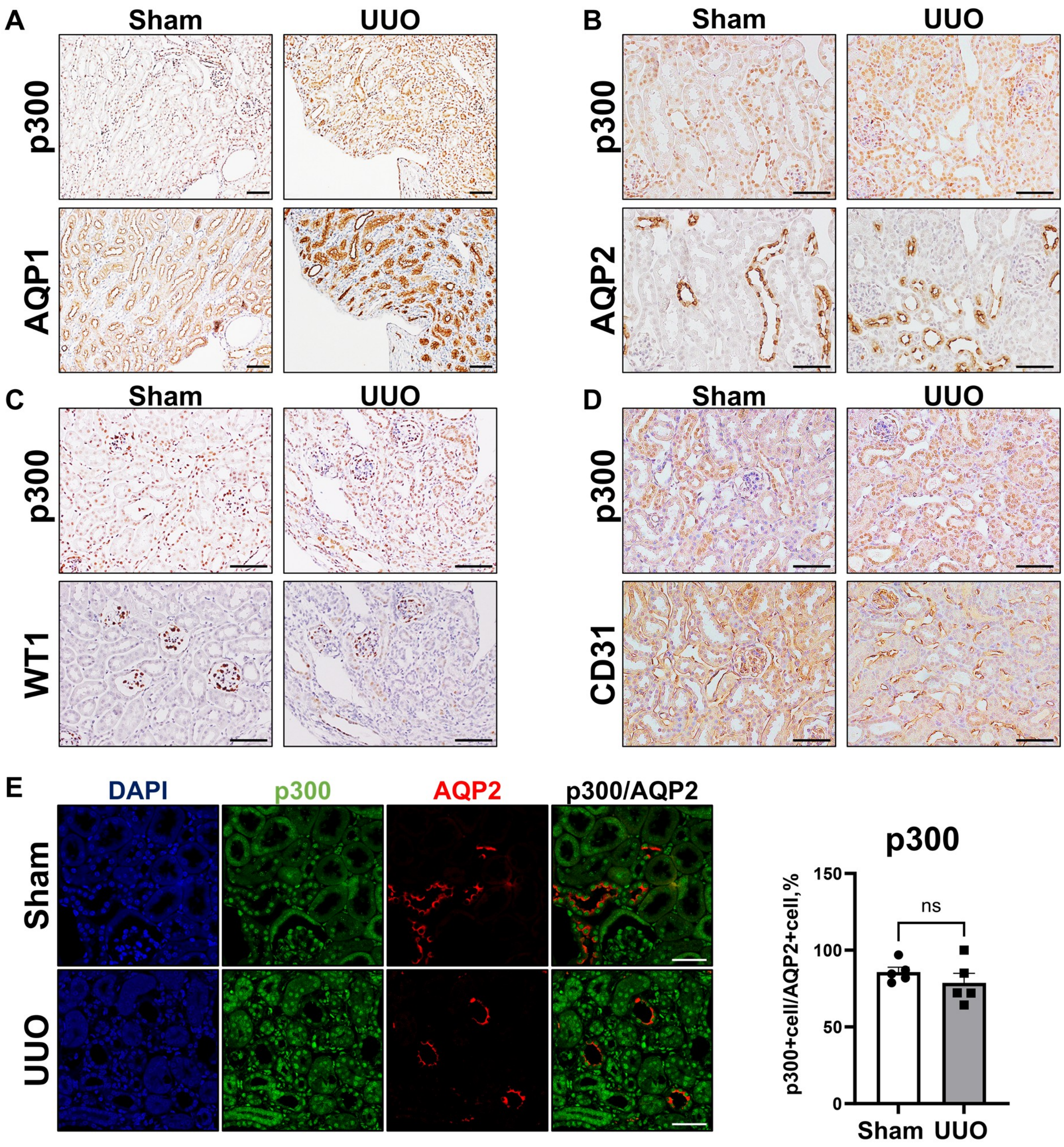

Appendix Figure S3. p300 expression is up-regulated in PTCs during renal fibrosis. (A-D) Kidney tissues from UUO-induced mouse fibrosis models. (A) Representative image of p300 and AQP1 immunohistochemistry (IHC) using serially sectioned samples. bar=100μm. (B) Representative image of p300 and AQP2 immunohistochemistry (IHC) using serially sectioned samples. bar=100μm. (C) Representative image of p300 and WT1 immunohistochemistry (IHC) using serially sectioned samples. bar=100μm. (D) Representative image of p300 and CD31 immunohistochemistry (IHC) using serially sectioned samples. bar=100μm. (E) Representative image of p300 and AQP2 co-immunofluorescence (IF) in mouse kidney tissue from the UUO-induced mouse fibrosis model. The graph represents the proportion of p300-positive cells among AQP2-positive cells (n=5 per group). bar=100μm. Data are presented as mean ± SEM, n.s. not significant by t-test.

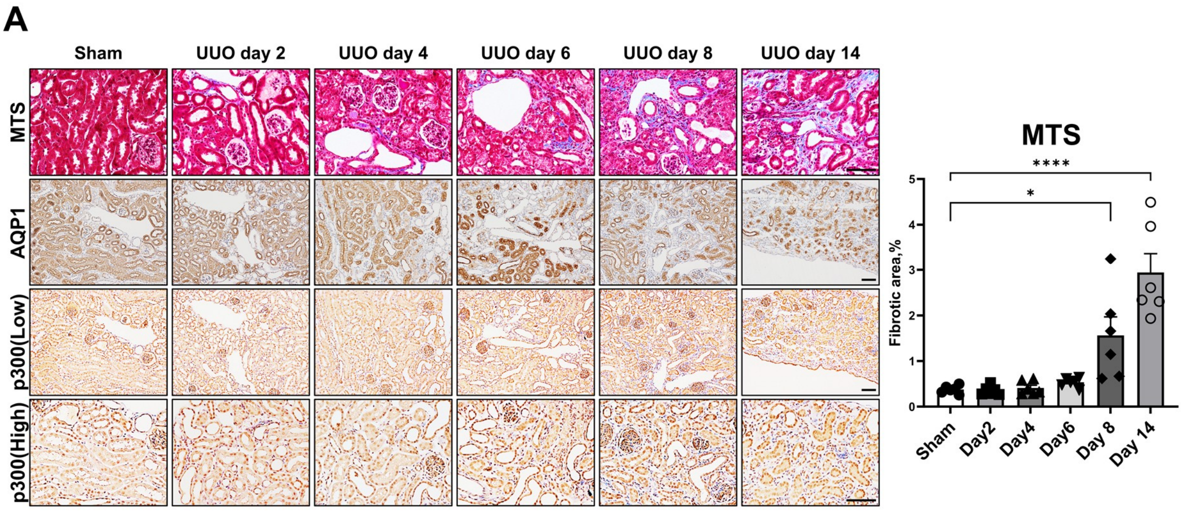

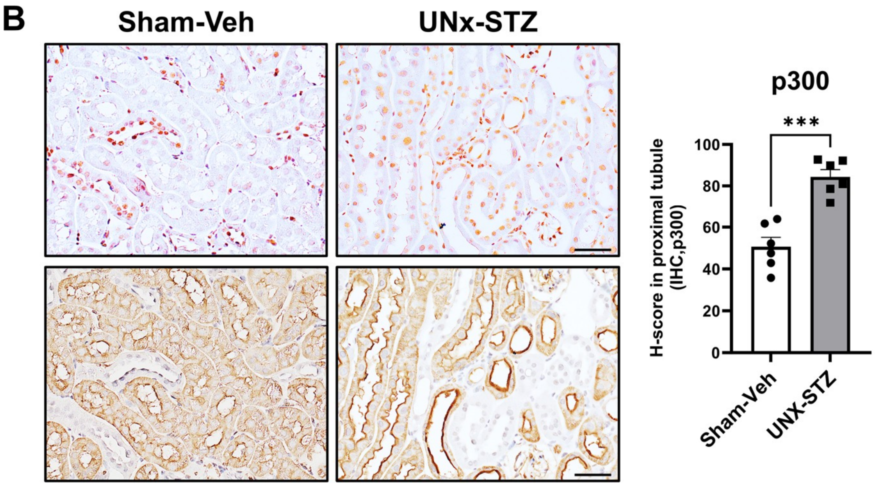

Appendix Figure S4. PTC-specific elevation of p300 in UUO and UNx-STZ-induced fibrosis models. (A) Representative image of Masson trichrome staining (MTS), and p300 and AQP1 immunohistochemistry (IHC) using serially sectioned kidney tissues from UUO-induced mouse kidney fibrosis models at 2, 4, 6, 8, and 14 days after surgery. The graph represents the quantification of fibrotic areas in Masson trichrome staining (MTS) images (n=6 per group). bar=100μm. (B) Representative image of p300 and AQP1 immunohistochemistry (IHC) using serially sectioned kidney tissues from UNx-STZ-induced diabetic fibrosis mouse models. The graph represents the expression of p300 in AQP1-positive cells analyzed by H-scoring (n=6 per group). bar=100μm. Data are presented as mean ± SEM, *P < 0.05, ***P < 0.001, and **P < 0.0001 by t-test (for two-group comparisons) and ordinary one-way ANOVA (for multiple groups).

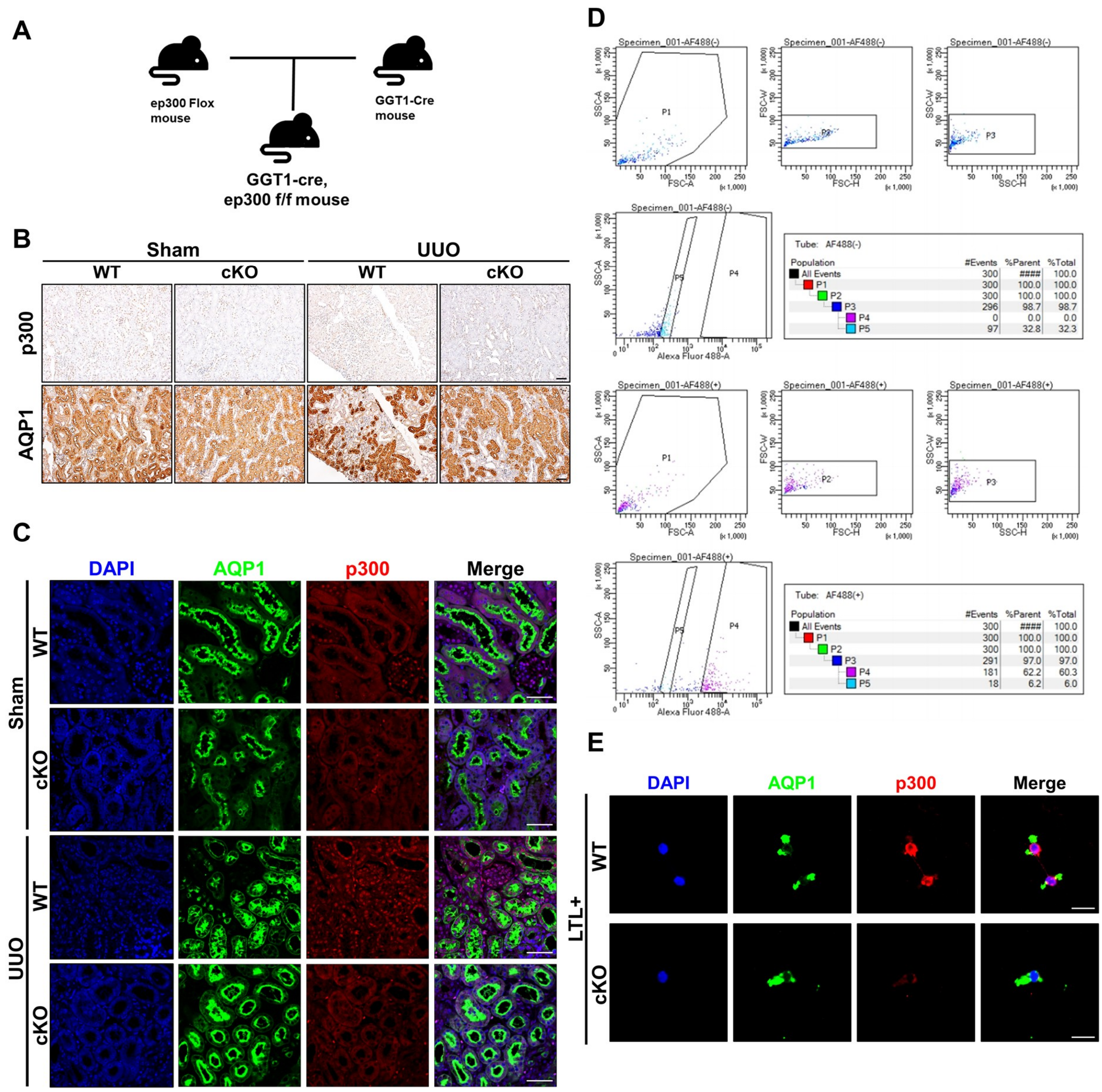

Appendix Figure S5. Generation of proximal tubular cell-specific p300 knock-out mice. (A) Schematic image of the process for generating proximal tubular cell-specific p300 knock-out mice. (B) Representative image of p300 and AQP1 immunohistochemistry (IHC) using serially sectioned kidney tissues from wild-type and p300 knock-out (cKO) UUO-induced fibrosis mouse models. bar=100μm. (C) Representative image of p300 and AQP1 co-immunofluorescence (IF) in kidney tissues from wild-type and p300 knock-out (cKO) UUO-induced mouse fibrosis models. bar=100μm. (D) Gating strategy for cell sorting using the BD ARIA 3 device. The P5 region in the upper graph represents Lotus Tetragonolobus Lectin (LTL)-negative cells, while the P4 region in the lower graph represents LTL-positive cells. (E) Representative image of p300 and AQP1 co-immunofluorescence (IF) in sorted LTL-positive cells from wild-type and p300 knock-out (cKO) mice. bar=25μm.

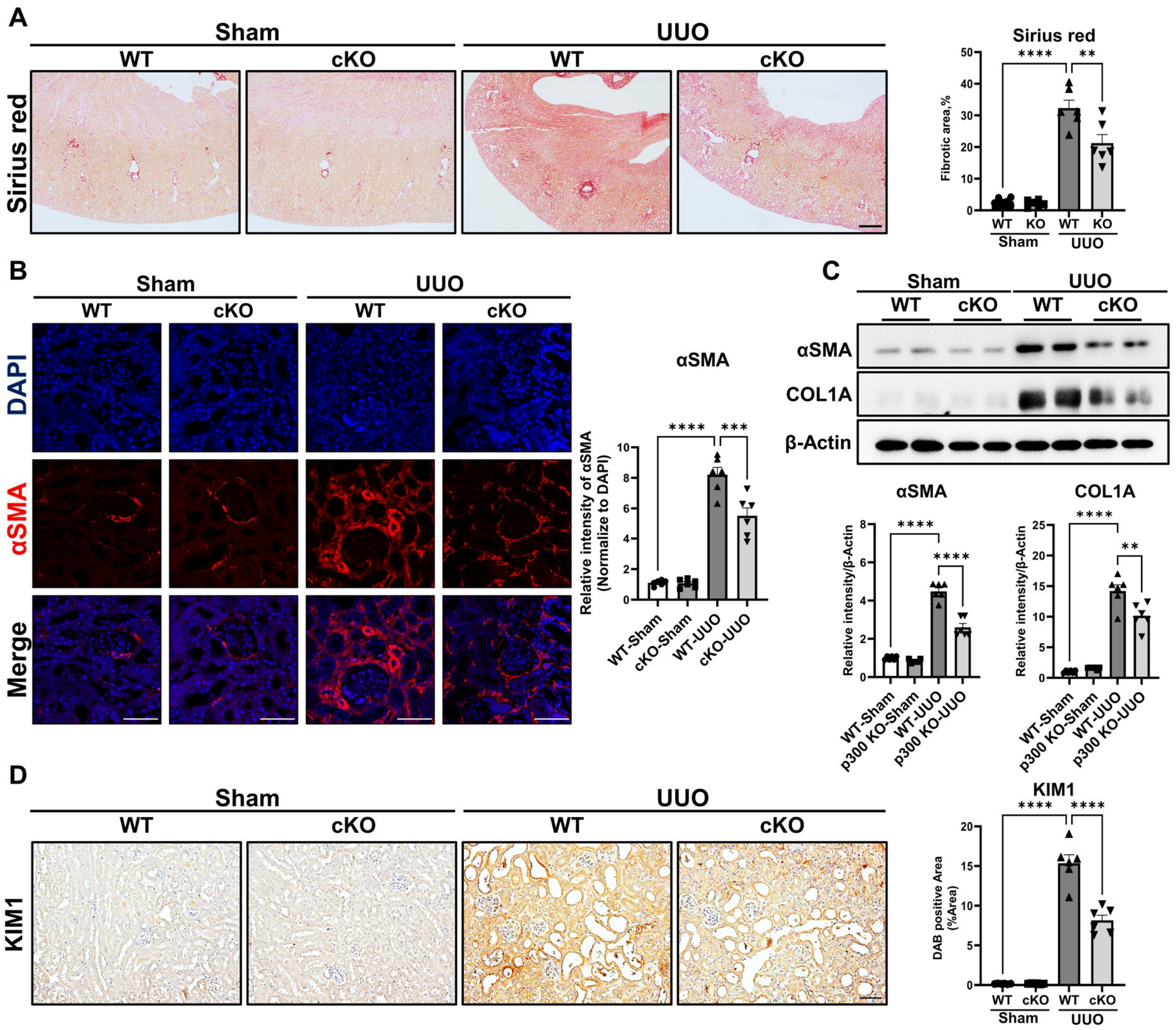

Appendix Figure S6. Proximal tubular cell-specific p300 knockout attenuates fibrosis progression in UUO-induced renal fibrosis mouse model. (A) Representative low-magnification image of Sirius red staining using kidney tissues from wild-type and p300 knock-out (cKO) UUO-induced mouse fibrosis models. The graph represents the fibrotic area quantified from histological staining images (n=6 per group). bar=250μm. (B) Representative image of αSMA immunofluorescence (IF) using kidney tissues from wild-type and p300 knock-out (cKO) UUO-induced mouse fibrosis models. The graph represents quantification of intensity in immunofluorescence (IF) images (n=6 per group). bar=100μm. (C) Protein levels of fibrosis markers, αSMA and COL1A, in kidney tissues from wild-type and p300 knock-out (cKO) UUO-induced mouse fibrosis models. β-Actin was used as the sample loading control. The graph represents quantification of intensity in western blot images, normalized to β-Actin (n=6 per group). (D) Representative image of KIM1 immunohistochemistry (IHC) using kidney tissues from wild-type and p300 knock-out (cKO) UUO-induced mouse fibrosis models. The graph represents quantification of DAB-positive areas (n=6 per group). bar=100μm. Data are presented as mean ± SEM, $**P < 0.01$, $***P < 0.001$, and $****P < 0.0001$ by ordinary one-way ANOVA.

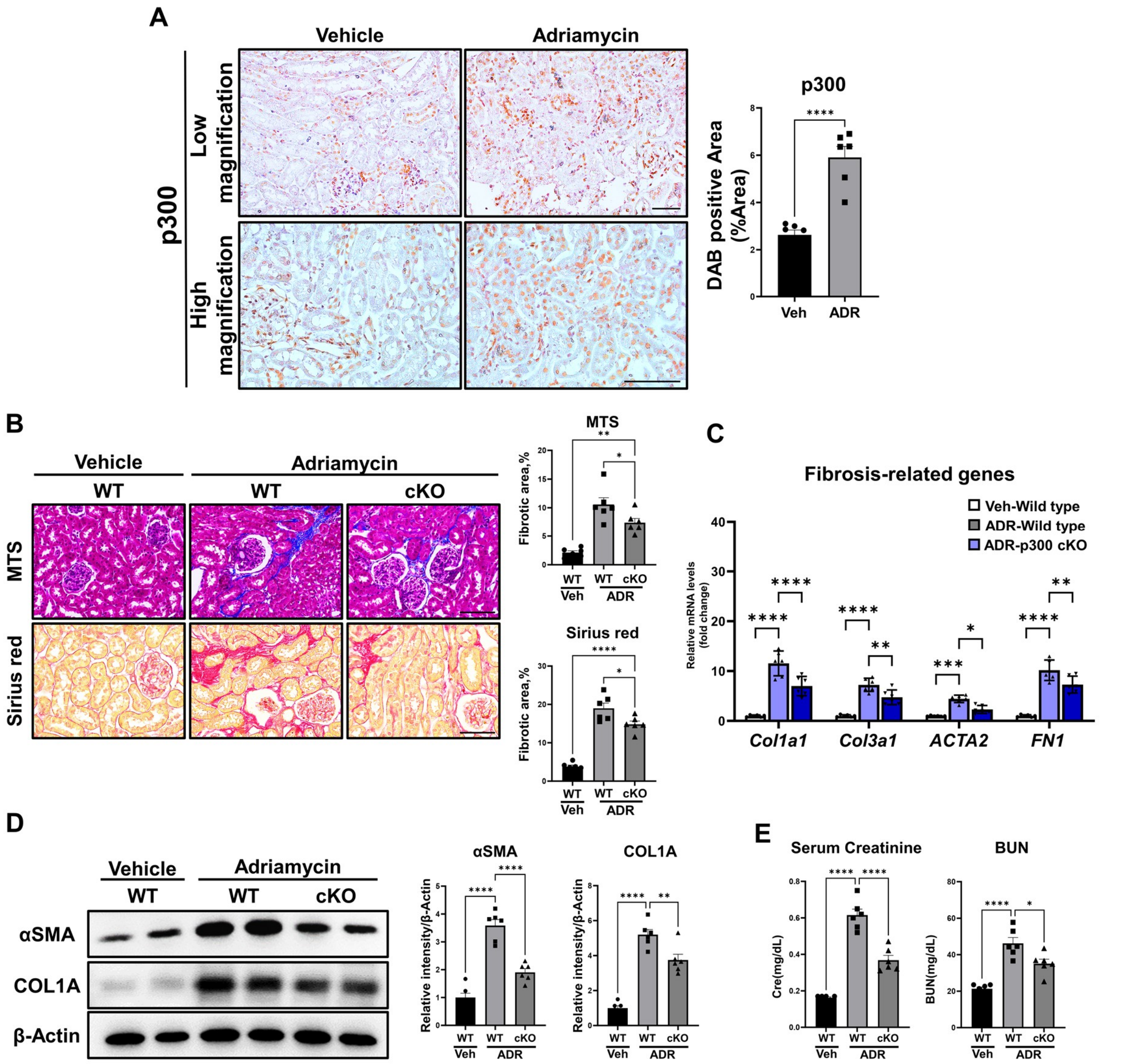

Appendix Figure S7. Evaluation of fibrosis and renal function in p300 cKO Adriamycin-induced fibrosis mouse models. (A) Representative images of p300 immunohistochemistry (IHC) in kidney tissues from ADR-induced mouse fibrosis model. The high-magnification and low-magnification images were taken from the same sample at different magnifications. The graph represents quantification of histological staining images (n=6 per group). bar=100μm. (B) Representative image of Masson trichrome staining (MTS) and Sirius red staining of kidney tissues from wild-type and p300 knockout (cKO) Adriamycin -induced fibrosis mouse models. The graph represents the quantification of fibrotic areas in histological staining images (n=6 per group). bar=100μm. (C) mRNA levels of fibrosis-related genes in kidney tissues from wild-type and p300 knock-out (cKO) Adriamycin-induced fibrosis mouse models (n=6 per group). (D) Protein levels of fibrosis markers, αSMA and COL1A, in kidney tissues from wild-type and p300 knock-out (cKO) Adriamycin-induced fibrosis mouse models. β-Actin was used as the loading control. The graph represents the quantification of intensity in western blot images, normalized to β-Actin (n=6 per group). (E) Renal function was assessed using serum samples from wild-type and p300 knock-out (cKO) Adriamycin-induced fibrosis mouse models (n=6 per group). Data are presented as mean ± SEM, *P < 0.05, **P < 0.01, ***P < 0.001, and ****P < 0.0001 by ordinary one-way ANOVA.

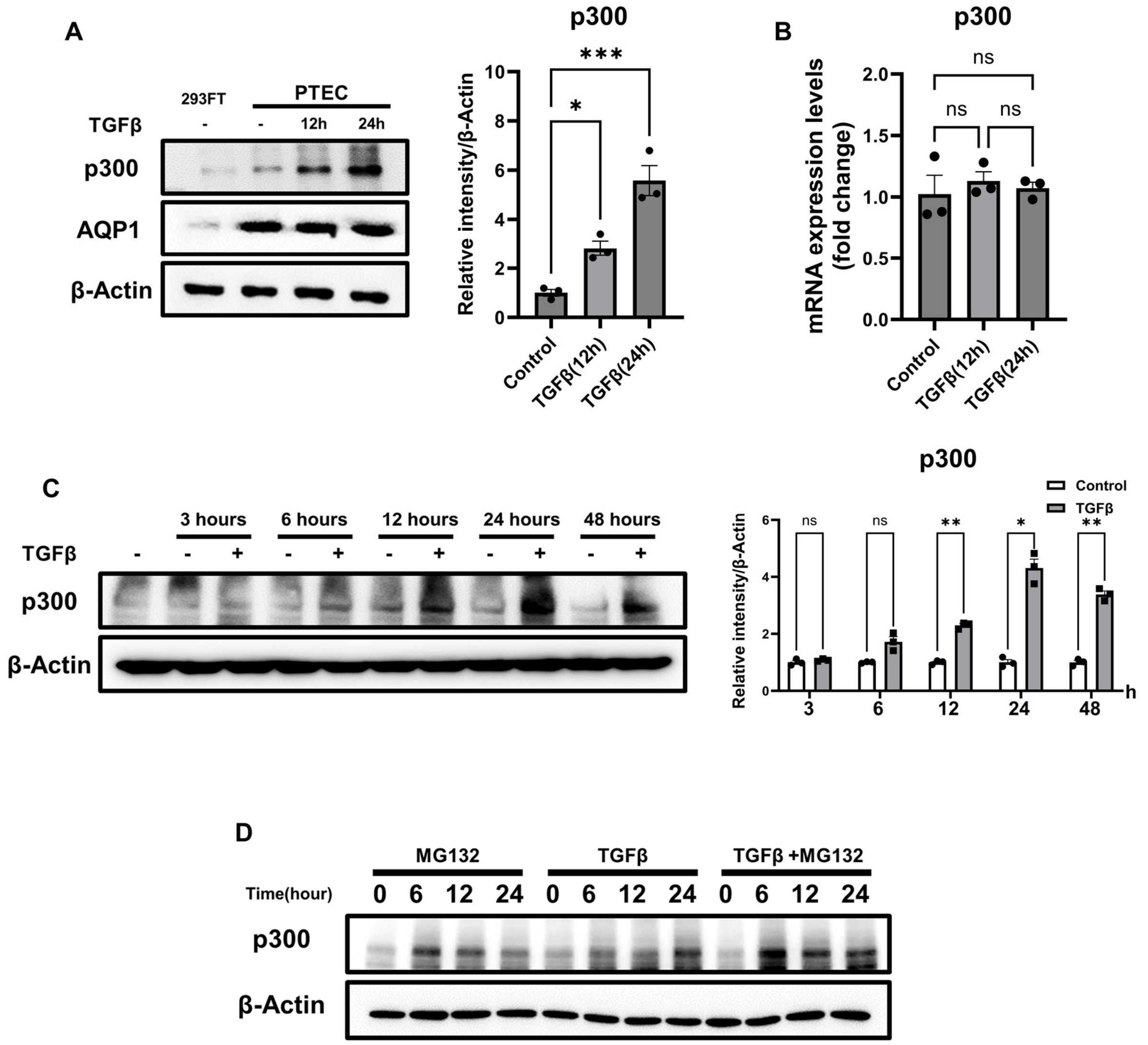

Appendix Figure S8. Proximal tubular cell p300 is elevated at the protein level, not the mRNA level, in response to TGFβ signaling. (A) Protein levels of p300 in primary mouse proximal tubular epithelial cells (PTECs) treated with TGFβ for 12 and 24 hours. The graph represents the quantification of intensity in western blot images, normalized to β-Actin (n=3 per group). (B) mRNA levels of p300 in primary mouse proximal tubular epithelial cells (PTECs) treated with TGFβ for 12 and 24 hours (n=3 per group). (C) Protein levels of p300 in HK2 cells treated with TGFβ for 3, 6, 12, 24, and 48 hours. β-Actin was used as the loading control. The graph represents the quantification of intensity in western blot images, normalized to β-Actin (n=3 per group). (D) Protein levels of p300 in HK2 cells treated with TGFβ and MG132 (a proteasome inhibitor) for 6, 12, and 24 hours. β-Actin was used as the loading control. Data are presented as mean ± SEM, ns: not significant, *P < 0.05 and **P < 0.01 by t-test (for two-group comparisons) and ordinary one-way ANOVA (for multiple groups).

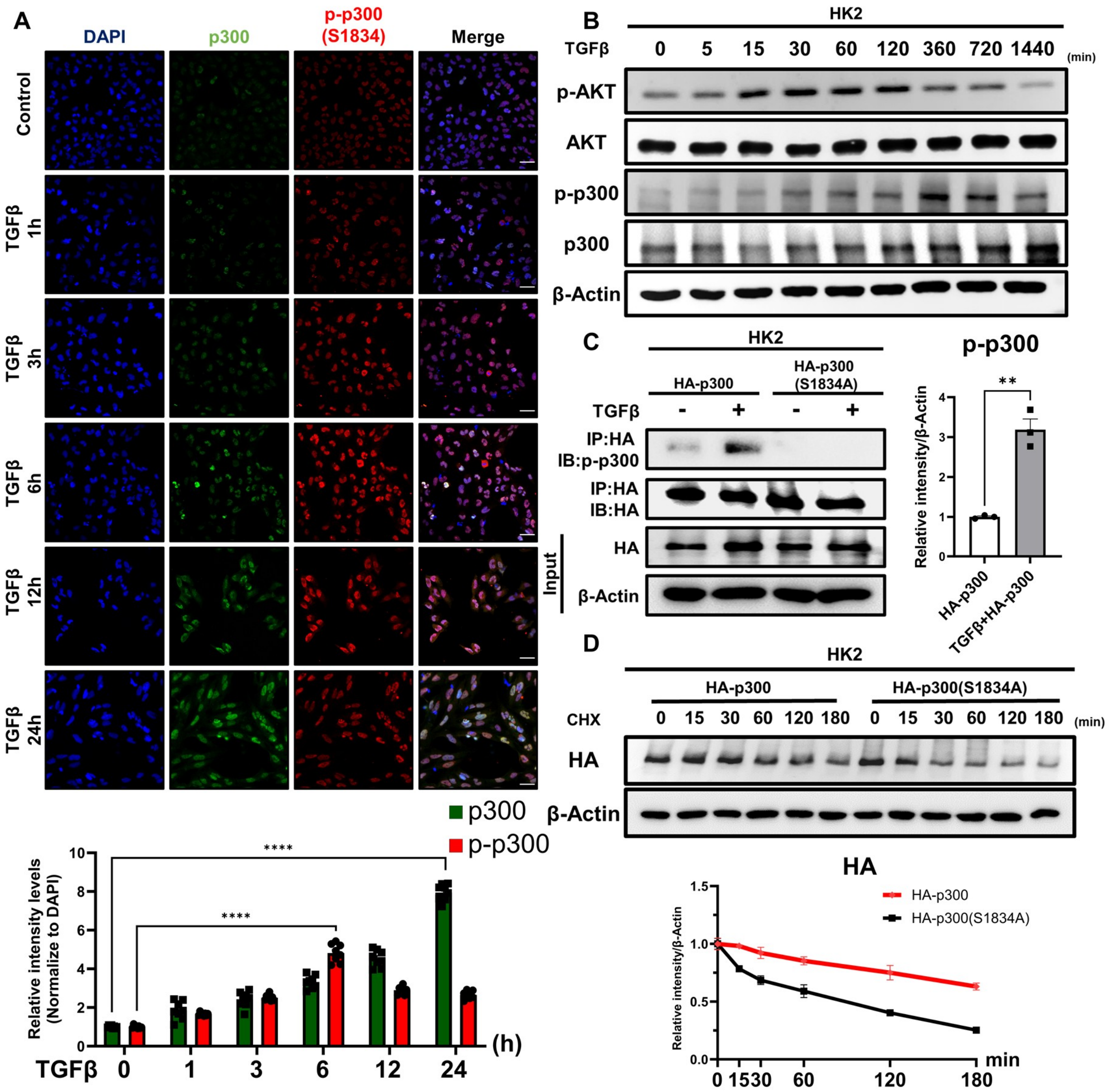

Appendix Figure S9. Phosphorylation of p300 at Serine 1834 via AKT signaling increases protein stabilization in response to TGFβ signaling. (A) Representative image of p300 and phosphorylated p300 (p-p300) immunofluorescence (IF) in HK2 cells treated with TGFβ for the indicated time. The graph represents the quantification of intensity in immunofluorescence (IF) images, normalized to DAPI (n=6 per group). bar=25μm. (B) Protein levels of AKT, phosphorylated AKT at Serine 473 (p-AKT), p300, and phosphorylated p300 at Serine 1834 (p-p300) in HK2 cells treated with TGFβ for the indicated time. β-Actin was used as the loading control. (C) Protein levels of HA, phosphorylated p300 at Serine 1834, HA-p300, and HA-p300Ser1834Ala mutant transfected into HK2 cells treated with TGFβ were analyzed by immunoblotting following immunoprecipitation (IP) using an anti-HA antibody. The graph represents the quantification of intensity in western blot images (n=3 per group). (D) Protein levels of HA and p300 in HA-p300 and HA-p300 Ser1834Ala mutant transfected HK2 cells treated with cycloheximide (CHX, a protein synthesis inhibitor) for the indicated time. β-Actin was used as the loading control. The graph represents the quantification of intensity in western blot images (n=3 per group). Data are presented as mean ± SEM, **P < 0.01 and ****P < 0.0001 by t-test (for two-group comparisons) and one-way ANOVA (for multiple groups).

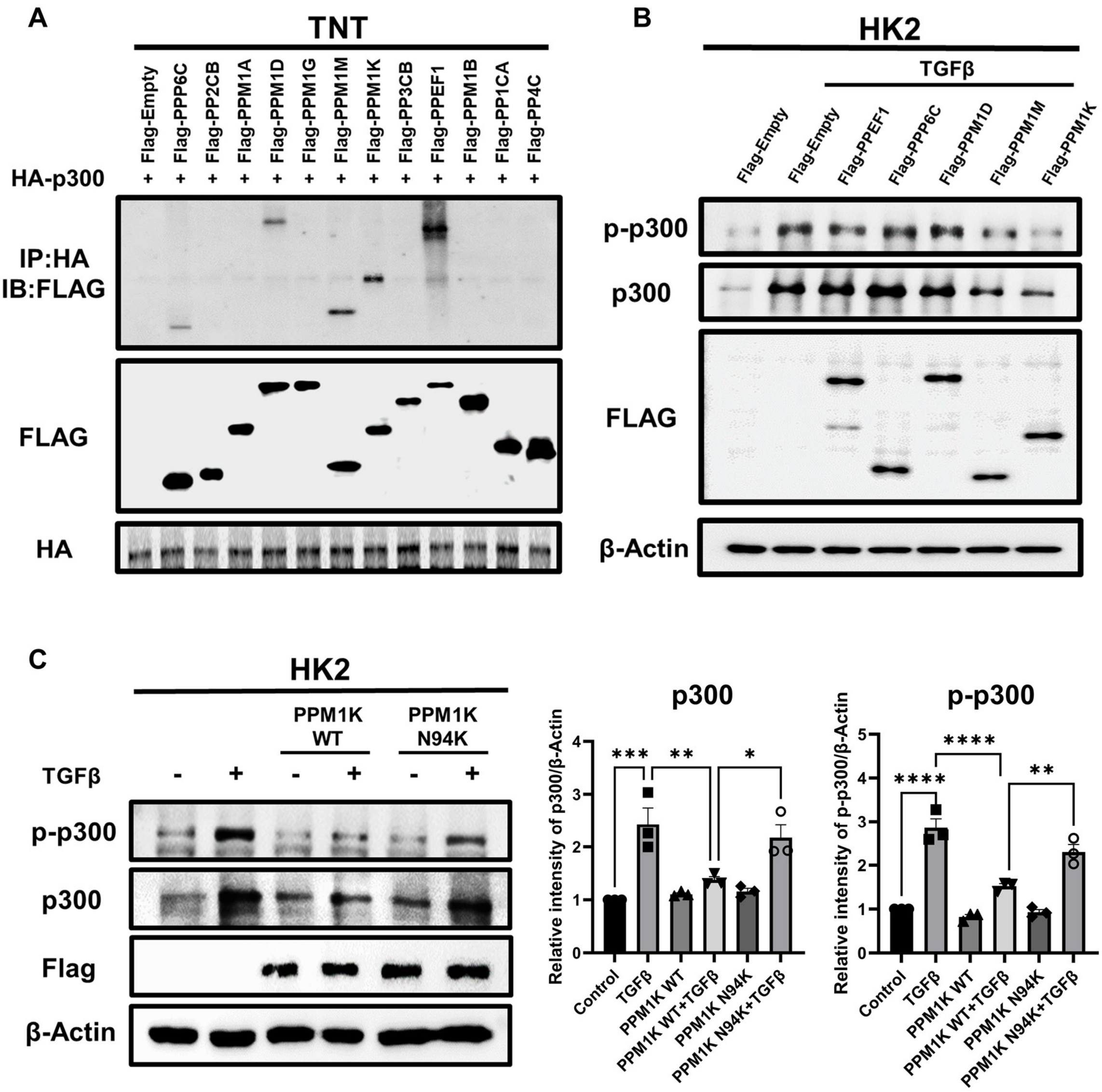

Appendix Figure S10. PPM1K binds to and dephosphorylates Serine 1834 of p300. (A) Screening of p300 partner phosphatases that dephosphorylate the Serine 1834 site via immunoprecipitation assay. (B) Protein levels of p300 and phosphorylated p300 at Serine 1834 in phosphatase-transfected HK2 cells treated with TGFβ for 12 hours. β-Actin was used as the loading control. (C) Protein levels of p300 and phosphorylated p300 at Serine 1834 in PPM1K and PPM1K Asparagine-to-Lysine mutant construct-transfected HK2 cells treated with TGFβ for 12 hours. β-Actin was used as the loading control. The graph represents the quantification of intensity in western blot images, normalized to β-Actin (n=3 per group). Data are presented as mean ± SEM, *P < 0.05, **P < 0.01, ***P < 0.001, and ****P < 0.0001 by ordinary one-way ANOVA.

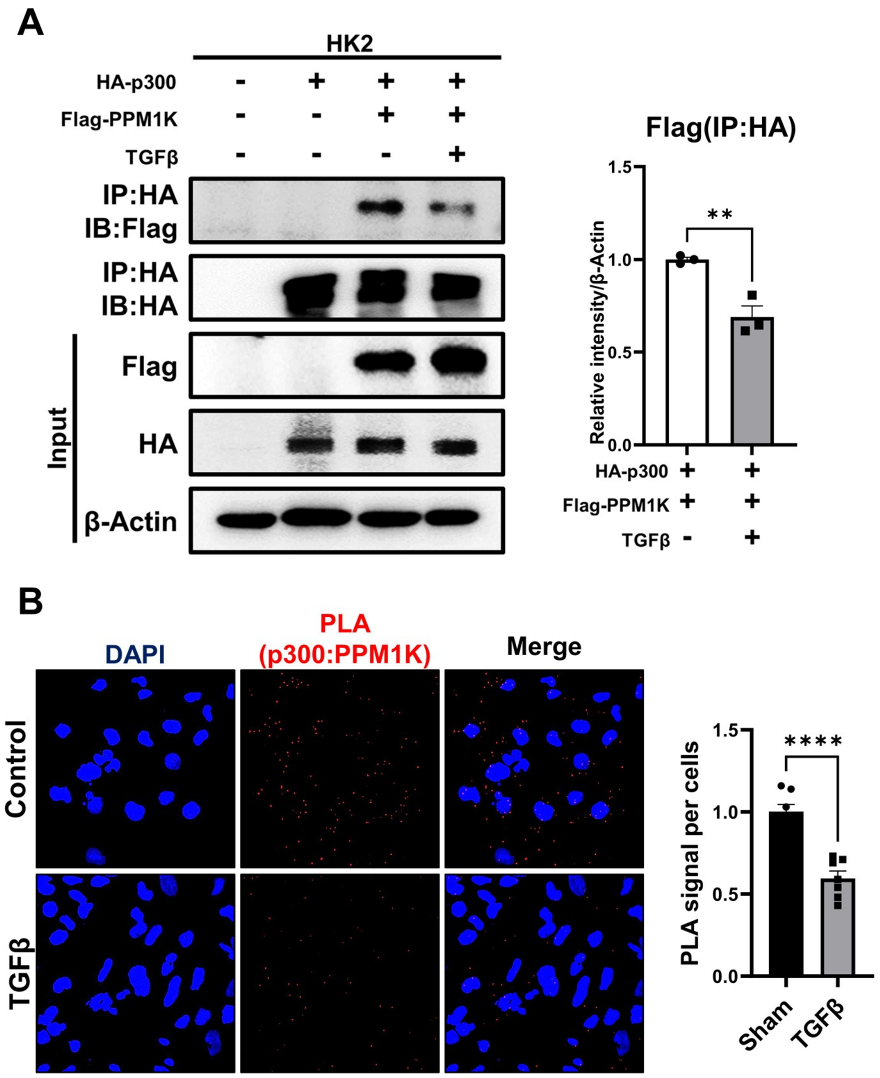

Appendix Figure S11. TGFβ stimulation induces PPM1K dissociation from p300. (A) Interaction of p300 and PPM1K in HK2 cells treated with TGFβ for 24 hours. β-Actin was used as the loading control. The graph represents the quantification of intensity in western blot images, normalized to β-Actin (n=3 per group). (B) Representative image of Proximity Ligation Assay (PLA) in HK2 cells treated with TGFβ for 24 hours. The graph represents the quantification of PLA signal, normalized to DAPI (n=7 per group). bar=25μm. Data are presented as mean ± SEM, , **P < 0.01 and ****P < 0.0001 by t-test.

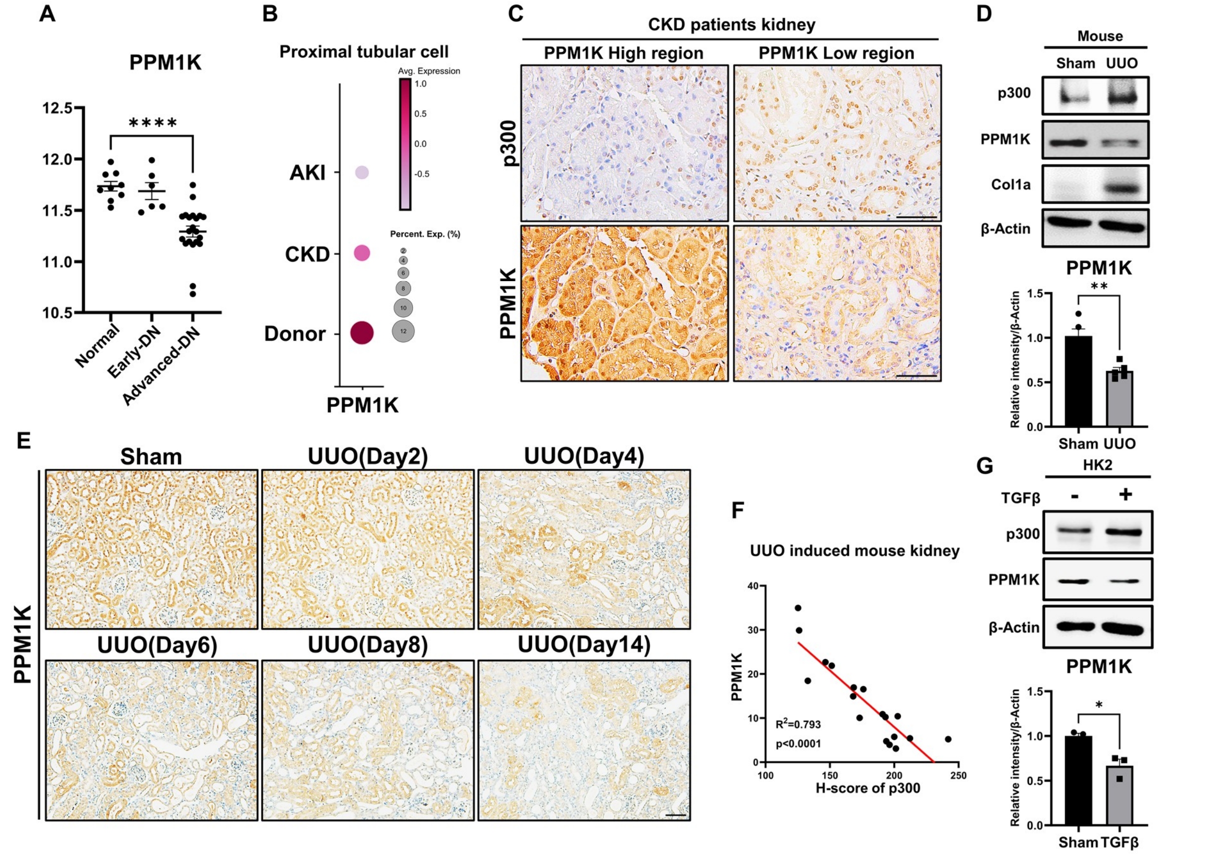

Appendix Figure S12. Inverse correlation of p300 and PPM1K in CKD patients and the UUO-induced mouse fibrosis model. (A) mRNA levels of PPM1K in CKD patients' (DN) kidneys. RNA-sequencing data retrieved from the Gene Expression Omnibus (GEO) database (GSE142025). (B) Analysis of mRNA expression levels of PPM1K in CKD and AKI patients' kidney proximal tubular epithelial cells using a human single-cell RNA-sequencing dataset (GSE183279). (C) Representative image of PPM1K immunohistochemistry (IHC) in kidney tissues from UUO-induced mouse kidneys at 2, 4, 6, 8, and 14 days after surgery. bar=100μm. (D) Protein levels of p300, PPM1K, and Col1A in the UUO-induced mouse fibrosis model kidney. β-Actin was used as the loading control. The graph represents the quantification of intensity in western blot images, normalized to β-Actin (n=5 per group). (E) Representative image of PPM1K and p300 immunohistochemistry (IHC) in PPM1K-high and PPM1K-low regions of CKD patient kidneys. bar=100μm. (F) Correlation analysis of H-scores for p300 and PPM1K expression in UUO-induced mouse kidneys. (n=18) (G) Protein levels of p300 and PPM1K in HK2 cells treated with TGFβ for 24 hours. β-Actin was used as the loading control. The graph represents the quantification of intensity in western blot images, normalized to β-Actin (n=3 per group). Data are presented as mean ± SEM, , *P < 0.05 and **P < 0.01 and ****P < 0.0001 by t-test (for two-group comparisons) and ordinary one-way ANOVA (for multiple groups).

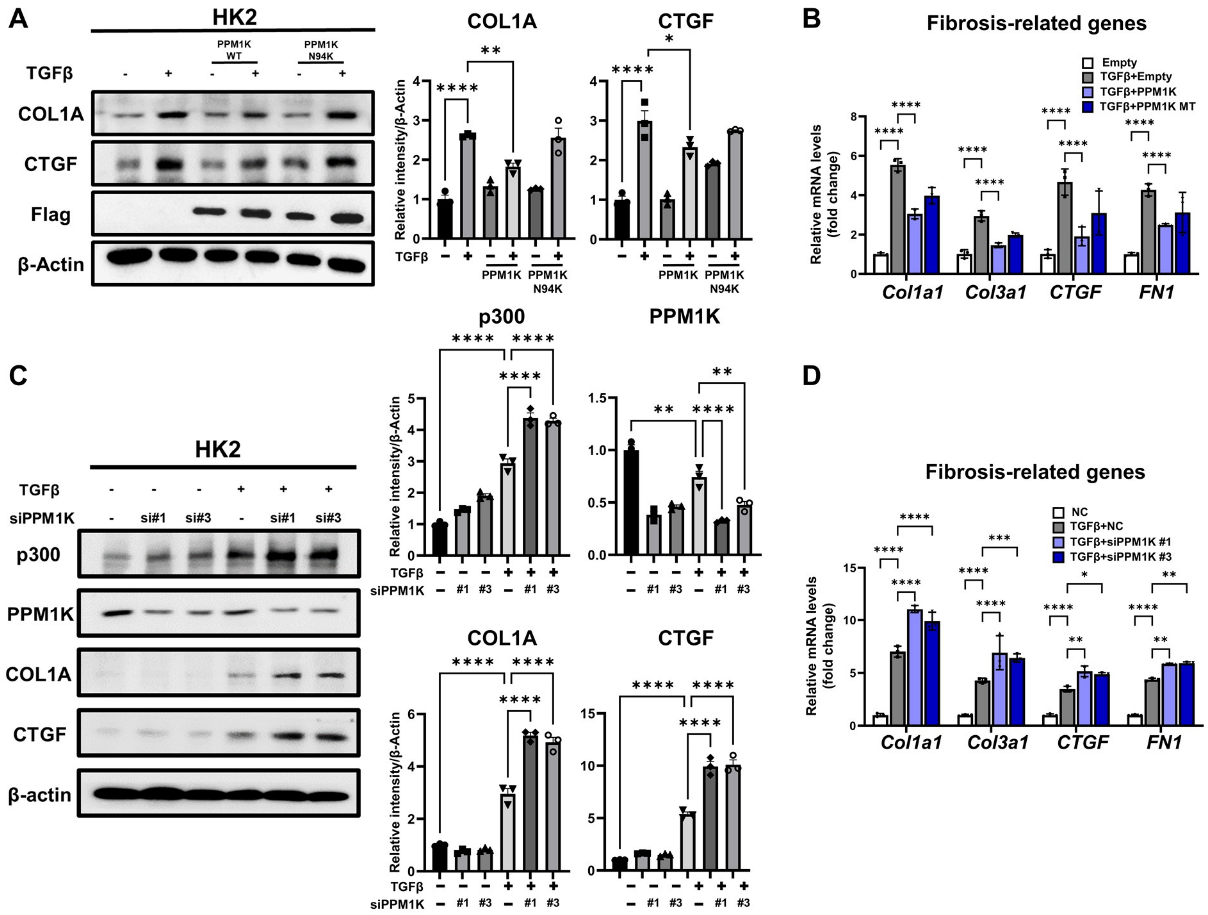

Appendix Figure S13. PPM1K regulates fibrosis-related protein and gene expression in HK2 cells treated with TGFβ. (A) Protein levels of Col1A and CTGF in Flag-PPM1K- and Flag-PPM1K N94K-transfected HK2 cells treated with TGFβ for 24 hours. β-Actin was used as the loading control. The graph represents the quantification of intensity in western blot images, normalized to β-Actin (n=3 per group). (B) mRNA levels of fibrosis-related genes in Flag-PPM1K- or Flag-PPM1K N94K-transfected HK2 cells treated with TGFβ for 24 hours (n=3 per group). (C) Protein levels of p300, PPM1K, Col1A, and CTGF in siPPM1K-transfected HK2 cells treated with TGFβ for 24 hours. β-Actin was used as the loading control. The graph represents the quantification of intensity in western blot images, normalized to β-Actin (n=3 per group). (D) mRNA levels of fibrosis-related genes in siPPM1K #1 and #3-transfected HK2 cells treated with TGFβ for 24 hours (n=3 per group). Data are presented as mean ± SEM, *P < 0.05, **P < 0.01, ***P < 0.001, and ****P < 0.0001 by ordinary one-way ANOVA.

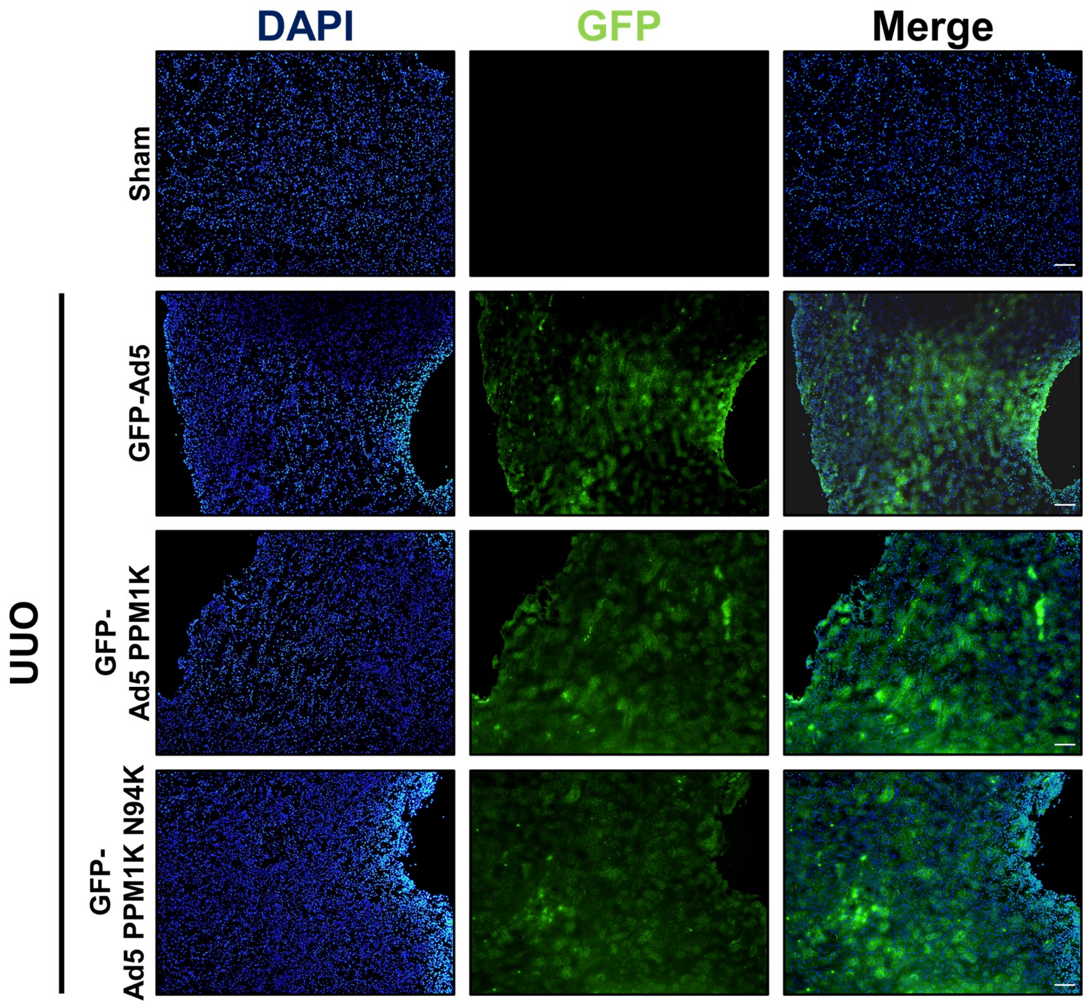

Appendix Figure S14. Confirmation of intra-renal Adenovirus delivery. Representative immunofluorescence (IF) image showing GFP-tagged virus introduction in kidney tissue. bar=100µm.

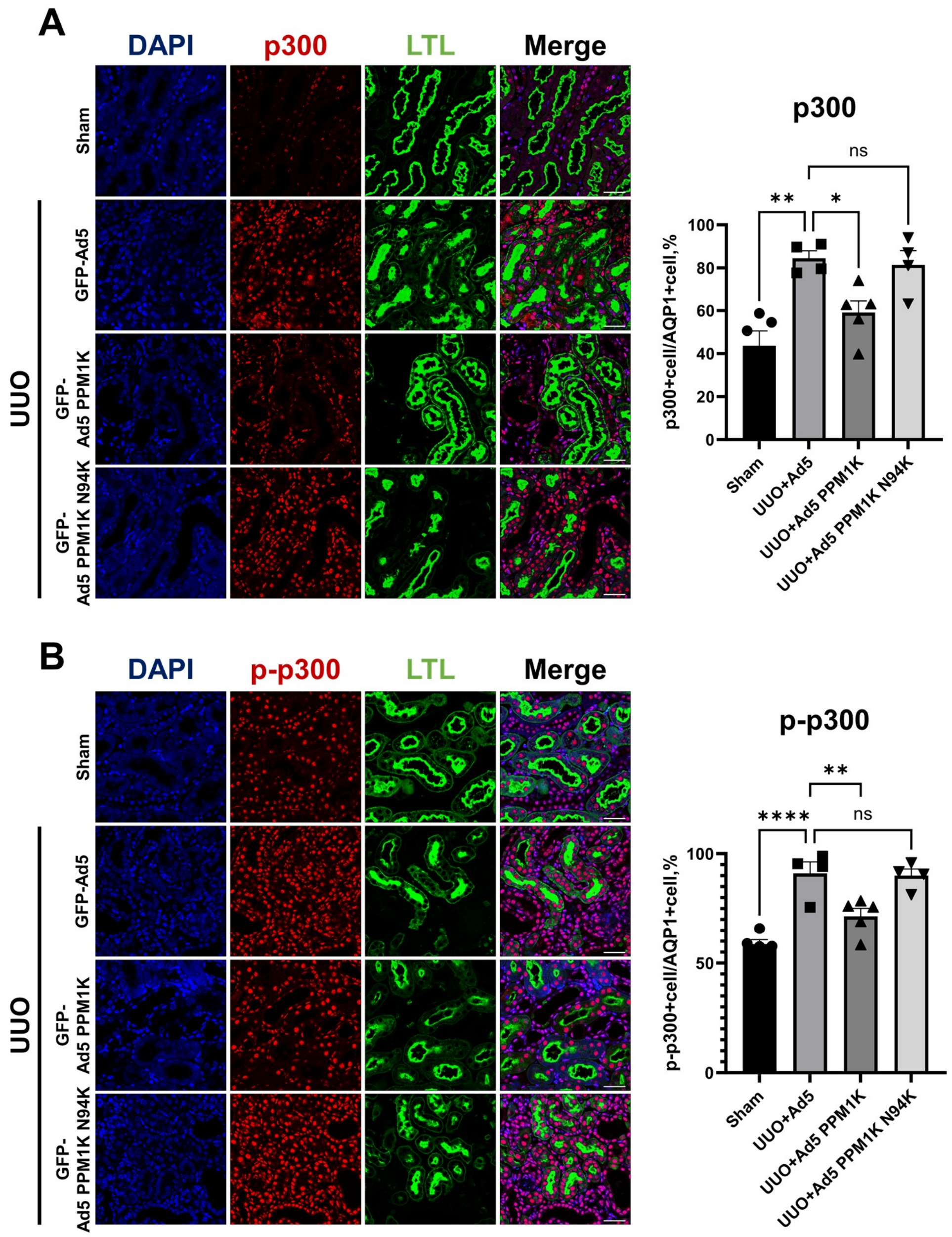

Appendix Figure S15. Overexpression of wild-type PPM1K significantly decreases both phosphorylation and stability of p300 in the UUO-induced mouse model. (A) Representative image of p300 and Lotus Tetragonolobus Lectin (LTL) co-immunofluorescence (IF) in kidney tissue samples from Ad5-PPM1K- and Ad5-PPM1K N94K-injected UUO-induced mouse fibrosis models. The graph represents the proportion of p300-positive cells among LTL-positive cells (Sham, n=5, UUO, n=4, UUO+Ad5-PPM1K, n=5, UUO+Ad5-PPM1K N94K, n=4). bar=100μm. (C) Representative image of p-p300 and Lotus Tetragonolobus Lectin (LTL) co-immunofluorescence (IF) in kidney tissue samples from Ad5-PPM1K- and Ad5-PPM1K N94K-injected UUO-induced mouse fibrosis models. The graph represents the proportion of p-p300-positive cells among LTL-positive cells Sham, n=5, UUO, n=4, UUO+Ad5-PPM1K, n=5, UUO+Ad5-PPM1K N94K, n=4). bar=100μm. Data are presented as mean ± SEM, *P < 0.05,**P < 0.01 and ****P < 0.0001 by ordinary one-way ANOVA.

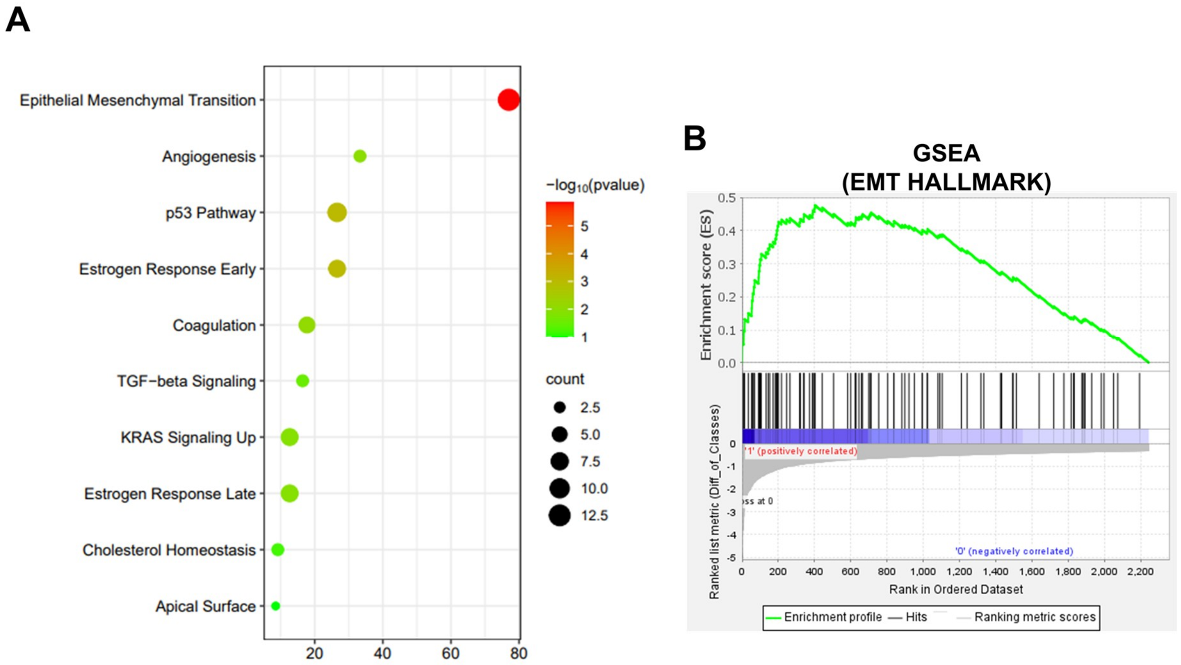

Appendix Figure S16. p300 in proximal tubular cells regulates mesenchymal transition-related gene expression. (A) Pathway analysis using MSigDB Hallmark for differentially expressed genes in proximal tubular cell-specific p300 knockout mouse kidneys compared to wild-type mouse kidneys. (B) Gene Set Enrichment Analysis (GSEA) for epithelial-to-mesenchymal transition (EMT)-related genes (downregulated) in proximal tubular cell-specific p300 knockout mouse kidneys compared to wild-type mouse kidneys.

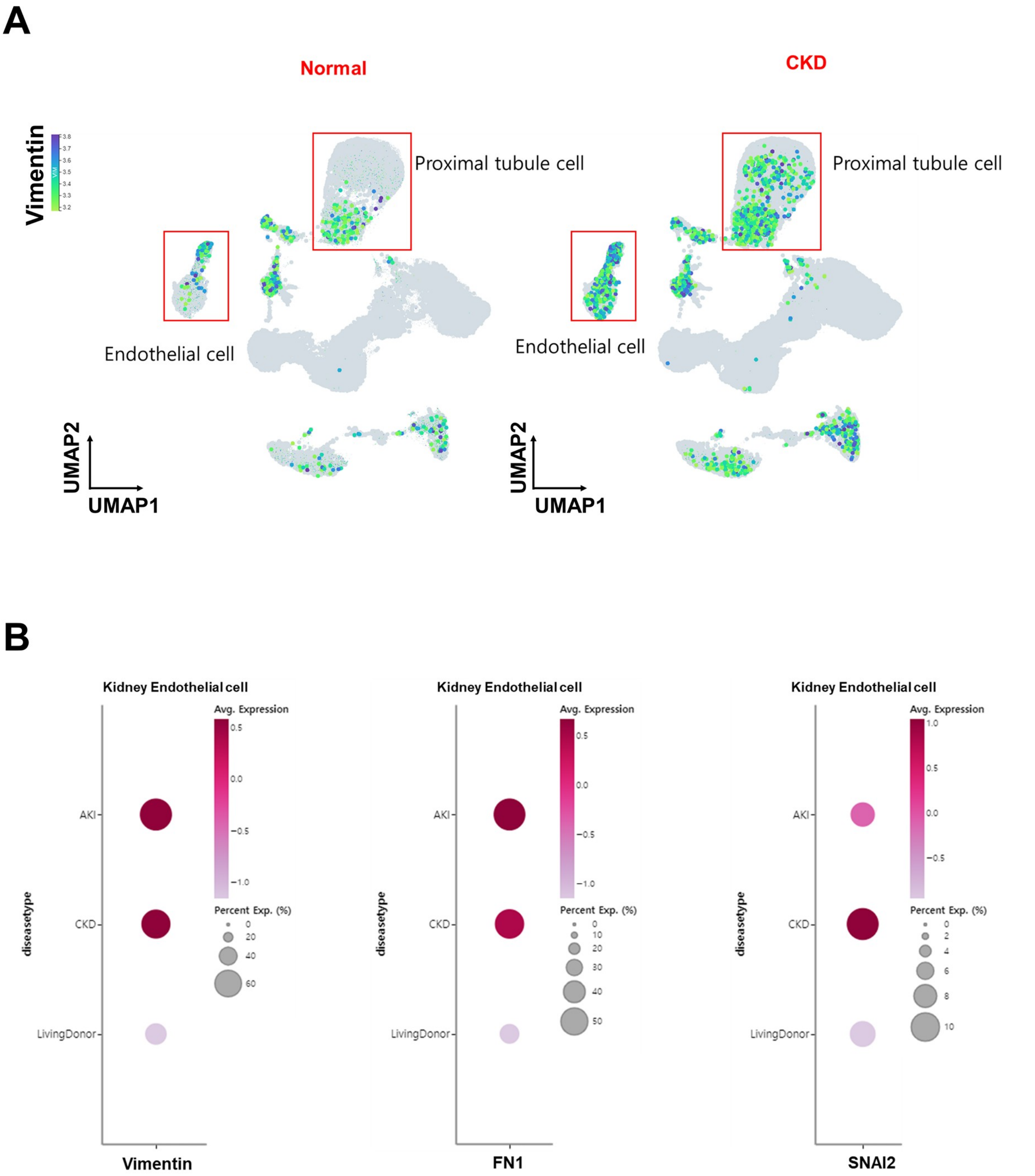

Appendix Figure S17. Increase in endothelial-to-mesenchymal transition in CKD patients. (A) UMAP representation of cell clusters from CKD patient samples. Vimentin expression in proximal tubule cells and endothelial cell populations (red box) is represented by green to blue dots. Single-cell RNA sequencing data were retrieved from a public database. (B) Expression levels of mesenchymal marker genes (*Vimentin, FN1* and *SNAI2*) in endothelial cells. Single-cell RNA sequencing data from CKD patients were retrieved from a public database (GSE183279).

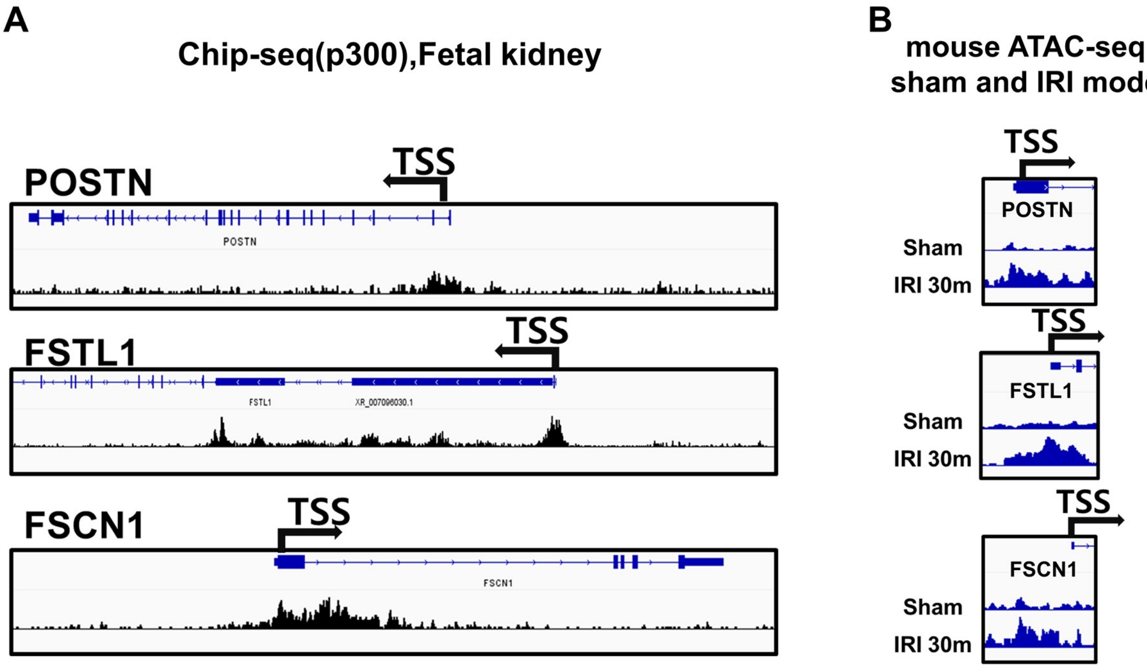

Appendix Figure S18. p300 binds to the promoter regions of FSTL1, FSCN1, and POSTN during renal fibrosis. (A) p300 enrichment in the promoter regions of POSTN, FSTL1, and FSCN1. Chromatin immunoprecipitation (ChIP)-sequencing data of human fetal kidney retrieved from a public database (GSE75948). (B) Chromatin accessibility of the promoter regions of POSTN, FSTL1, and FSCN1 in the ischemia-reperfusion injury (IRI) model mouse kidney. ATAC-sequencing data retrieved from a public database (GSE197815).

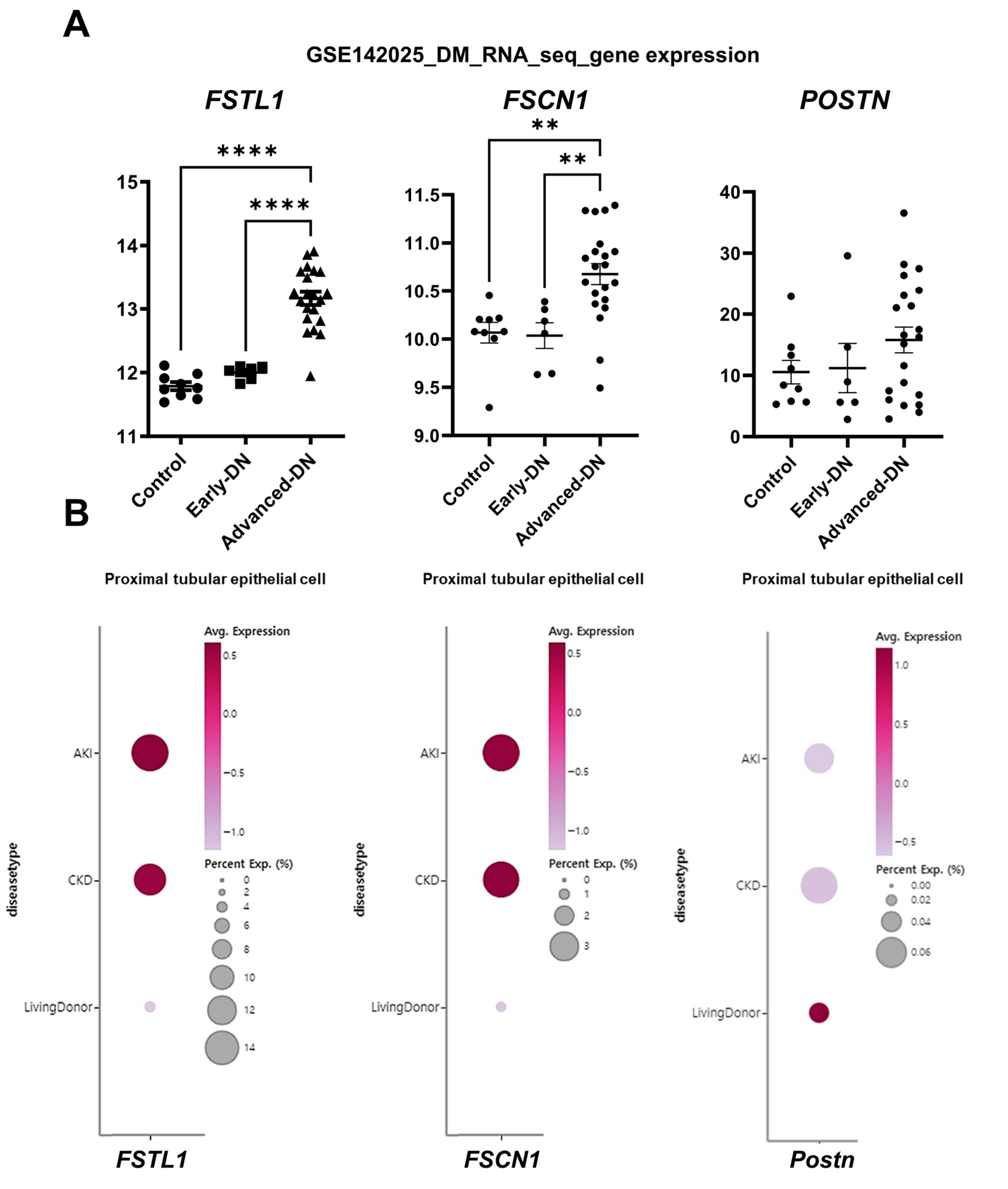

Appendix Figure S19. Increased expression levels of FSTL1, FSCN1, and POSTN in CKD patients. (A) mRNA levels of FSTL1, FSCN1, and POSTN. RNA-sequencing data from CKD patients (DN) retrieved from the Gene Expression Omnibus (GEO) (GSE142025). (B) Expression levels of FSTL1, FSCN1, and POSTN in kidney proximal tubular cells. Single-cell RNA-sequencing data from CKD and AKI patients retrieved from a public database (GSE183279). Data are presented as mean ± SEM, **P < 0.01 and ****P < 0.0001 by ordinary one-way ANOVA.

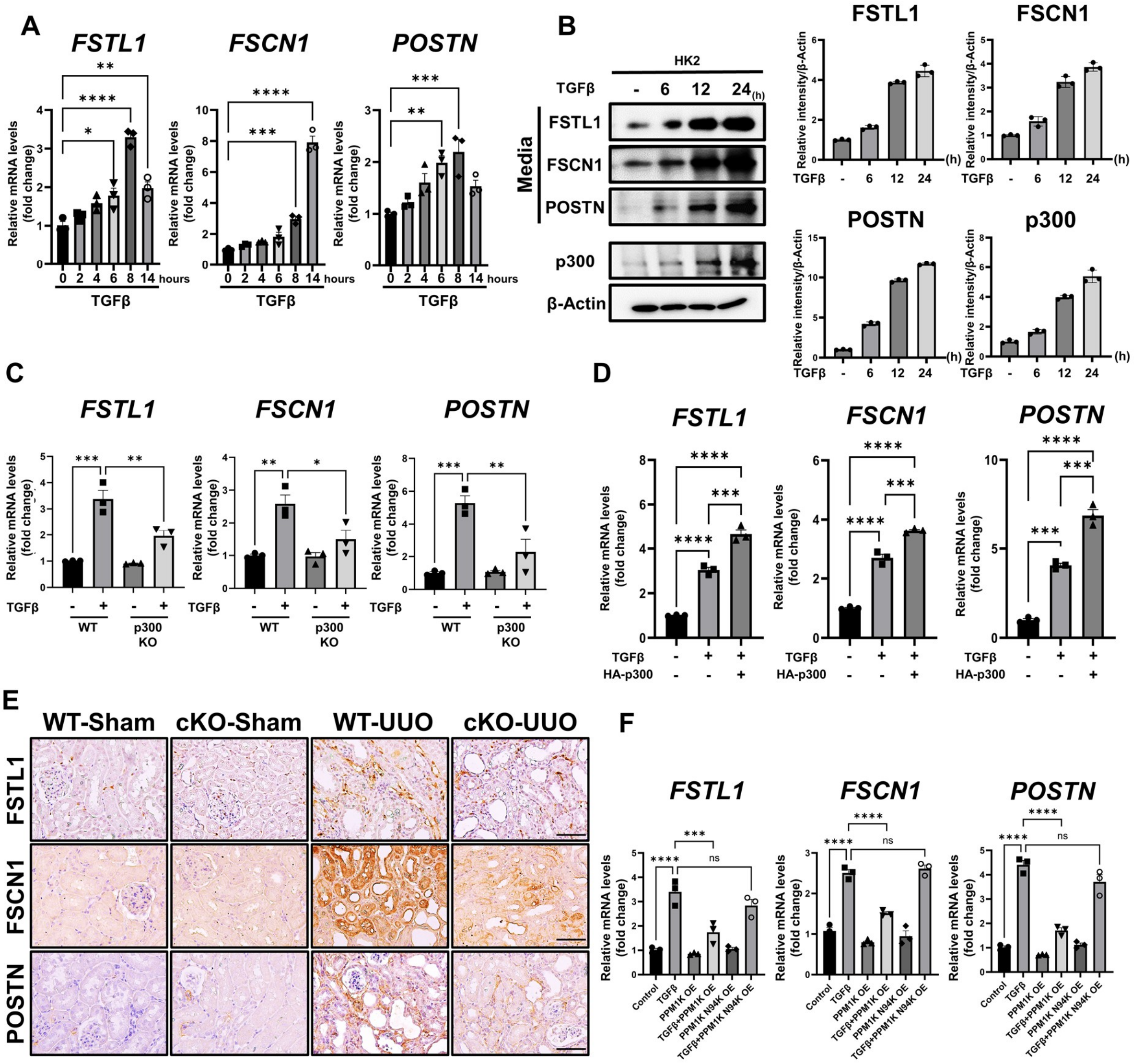

Appendix Figure S20. p300 regulates the expression of FSTL1, FSCN1, and POSTN in kidney proximal tubular cells. (A) mRNA levels of *FSTL1*, *FSCN1*, and *POSTN* in HK2 cells treated with TGFβ (n=3 per group). (B) Protein levels of FSTL1, FSCN1, and POSTN in the culture medium from HK2 cells, and p300 levels in HK2 cell lysates. HK2 cells were stimulated with TGFβ. β-Actin was used as the loading control. The graph represents the quantification of intensity in western blot images, normalized to β-Actin (n=3 per group). (C) mRNA levels of *FSTL1*, *FSCN1*, and *POSTN* in p300 knockout (KO) HK2 cells and wild-type HK2 cells treated with TGFβ for 8 hours (n=3 per group). (D) mRNA levels of FSTL1, FSCN1, and POSTN in p300-overexpressing HK2 cells treated with TGFβ for 8 hours (n=3 per group). (E) Representative image of FSTL1, FSCN1, and POSTN immunohistochemistry (IHC) in kidney tissues from wild-type and p300 knock-out (cKO) UUO-induced fibrosis mouse models. bar=100μm. (F) mRNA levels of *FSTL1*, *FSCN1*, and *POSTN* in PPM1K- and PPM1K N94K-overexpressing HK2 cells treated with TGFβ for 8 hours (n=3 per group). Data are presented as mean ± SEM, *P < 0.05, **P < 0.01, ***P < 0.001 and ****P < 0.0001 by ordinary one-way ANOVA.

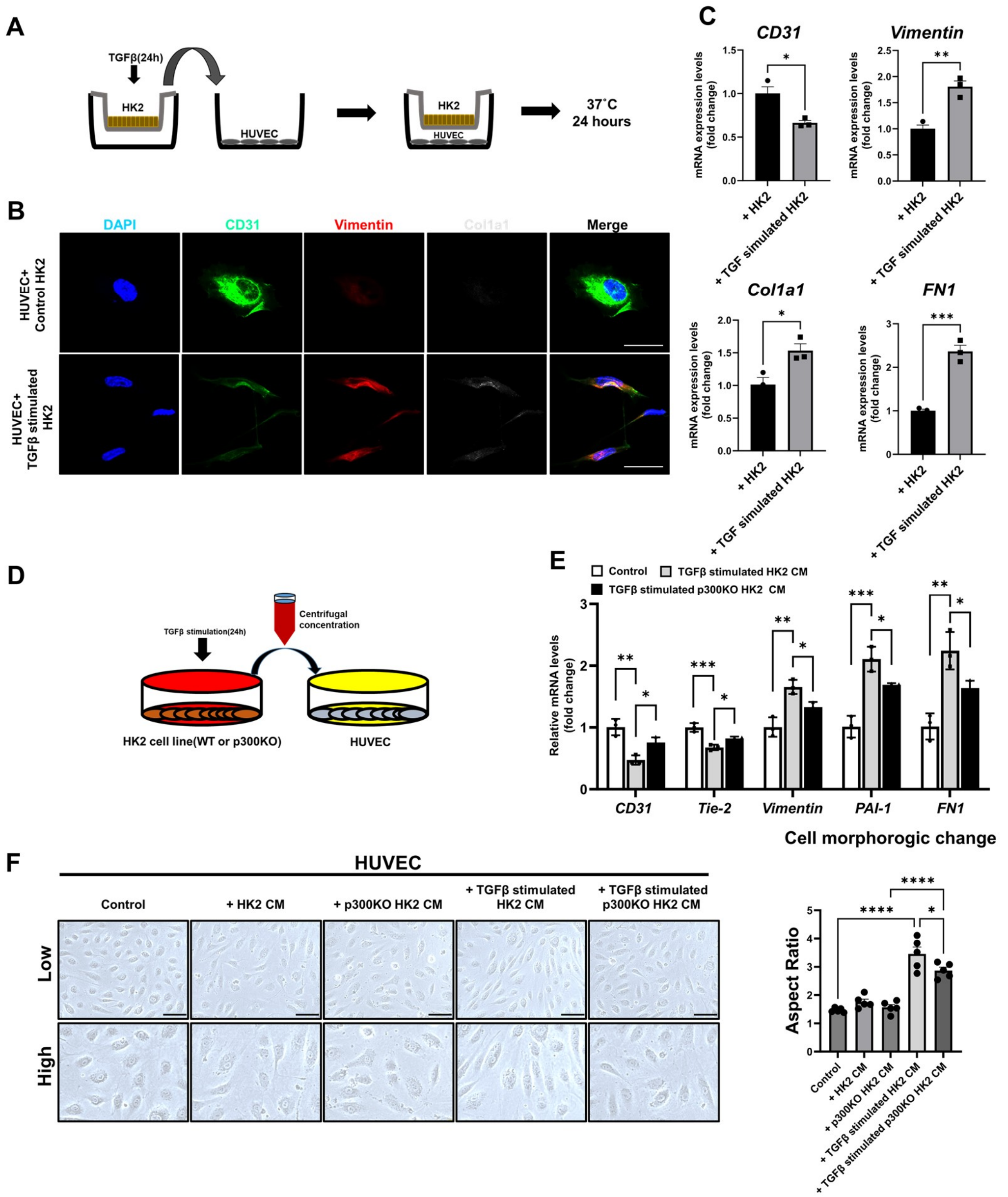

Appendix Figure S21. PTC-specific p300 mediates endothelial-to-mesenchymal transition. (A) Schematic image of the co-culture method. (B) Representative image of endothelial and mesenchymal cell marker co-immunofluorescence (IF) in HUVEC cells co-cultured with TGFβ-stimulated (24 hours) HK2 cells. bar=25μm. (C) mRNA levels of endothelial (*CD31*) and mesenchymal (*Vimentin, Col1a1* and *FN1*) cell marker genes in HUVEC cells co-cultured with TGFβ-stimulated (24 hours) HK2 cells (n=3 per group). (D) Schematic image of the conditioned medium transfer protocol. Conditioned medium from HK2 cell culture plates was concentrated and transferred to a HUVEC culture plate. (E) mRNA levels of endothelial and mesenchymal cell marker genes in HUVEC cells with TGFβ-stimulated HK2 cell-conditioned medium (n=3 per group). (F) Morphologic changes in HUVEC cells treated with TGFβ-stimulated (24 hours) HK2 cell-conditioned medium. The graph represents the aspect ratio (short axis/long axis) of HUVEC cells for evaluation of EndMT (n=5 per group). bar=25μm. Data are presented as mean ± SEM, *P < 0.05, **P < 0.01, ***P < 0.001 and ****P < 0.0001 by t-test (for two-group comparisons) and ordinary one-way ANOVA (for multiple groups).

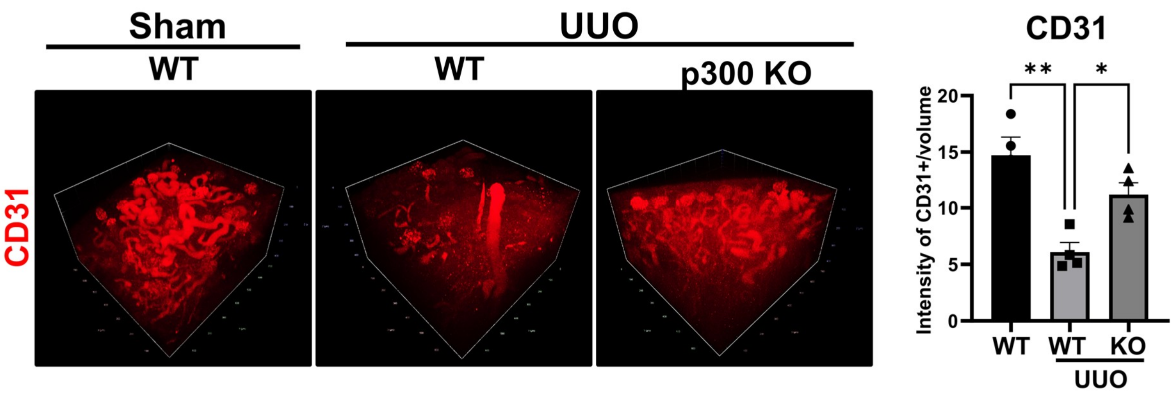

Appendix Figure S22. Renal microvascular structures in WT and p300 cKO UUO-induced mouse fibrosis model. Representative 3-D images of the microvascular structure (CD31) of kidney kidney tissues from wild-type and p300 knock-out (cKO) UUO-induced mouse fibrosis model. The graph represents quantification of CD31 intensity in 3-D images, normalized to tissue volume (n=4 per group). Data are presented as mean ± SEM. *P < 0.05 and **P < 0.01 by ordinary one-way ANOVA.

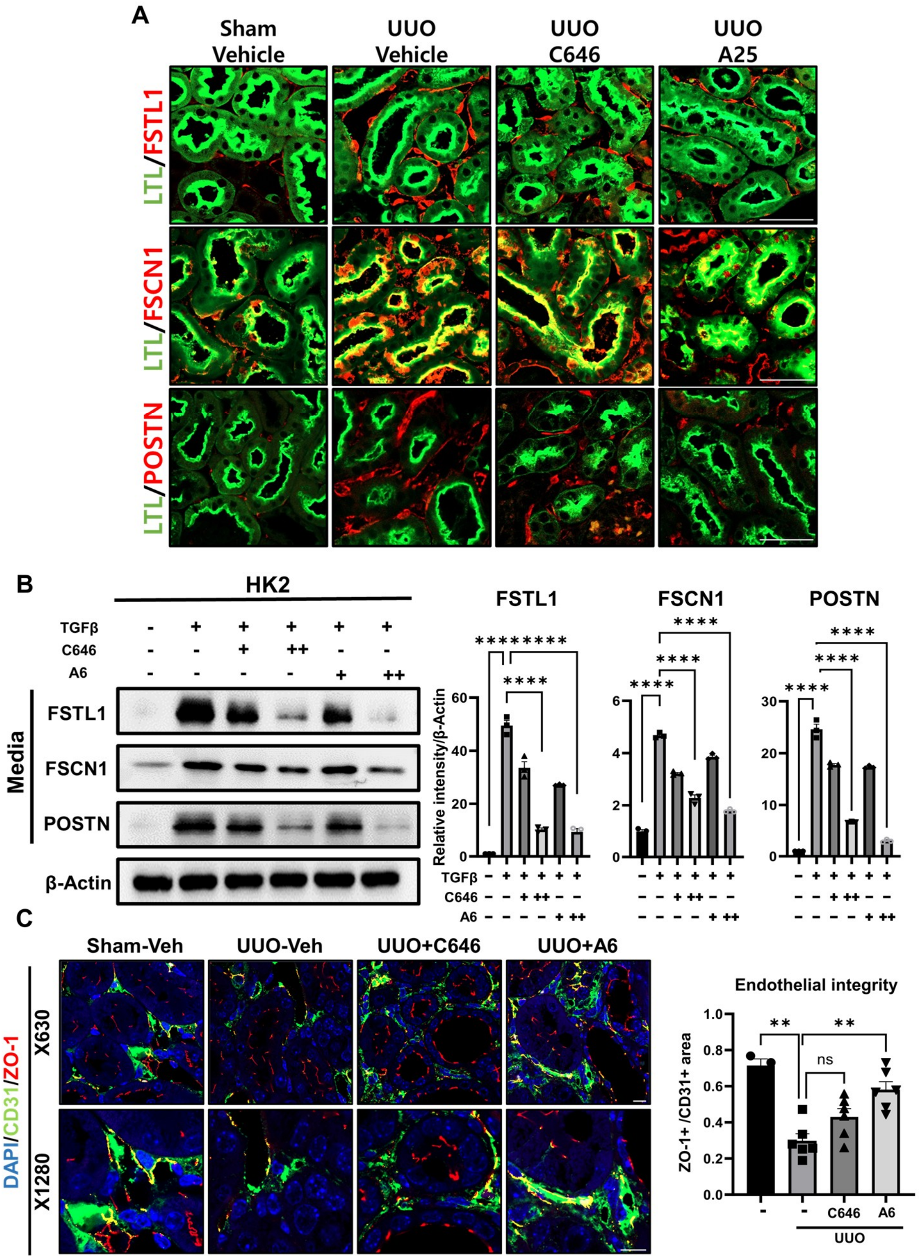

Appendix Figure S23. p300-specific inhibitors reduce the expression of mesenchymal transition-related genes and suppress EndMT progression. (A) Representative image of FSTL1, FSCN1, and POSTN co-immunofluorescence (IF) in kidney tissues from UUO-induced fibrosis mouse models injected with C646 and A6. bar=100μm. (B) Protein levels of FSTL1, FSCN1, and POSTN in the culture medium of HK2 cells treated with TGFβ and co-treated with C646 and A6. β-Actin was used as the loading control. The graph represents the quantification of intensity in western blot images, normalized to β-Actin (n=3 per group). (C) Representative image of CD31 and ZO-1 co-immunofluorescence (IF) in kidney tissue samples from UUO-induced fibrosis mouse models injected with C646 and A6. The graph represents the quantification of the co-localized area (ZO-1 and CD31) in co-immunofluorescence (IF) images of kidney tissues from the UUO-induced fibrosis mouse model injected with C646 and A6 (sham, n=3, UUO+Veh, n=6, UUO+C646, n=6, UUO+A6, n=6). bar=25μm. Data are presented as mean ± SEM, $*P < 0.05$, $**P < 0.01$, $***P < 0.001$ and $****P < 0.0001$ by ordinary one-way ANOVA.
