# Supplementary figures and images for: Loss of p300 in proximal tubular cells reduces renal fibrosis and endothelial-mesenchymal transition
Source: EMBO Mol Med. 2025 Jul 1;17(7):1575–98. doi: 10.1038/s44321-025-00243-1 (PMC12254316; doi:10.1038/s44321-025-00243-1)

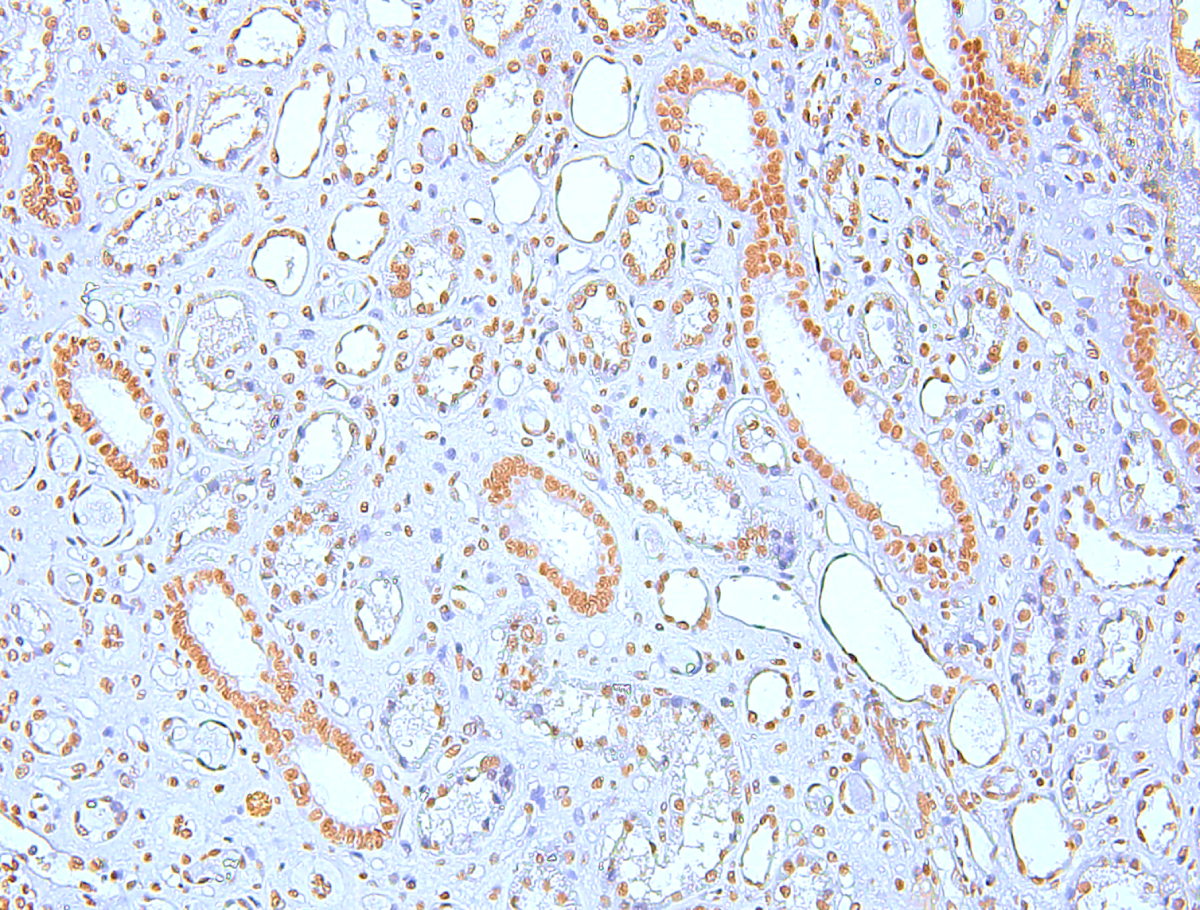

Supplement: Supplementary file 3 — Source data Fig. 1 [file 44321_2025_243_MOESM3_ESM.zip › 1A/A FSGS(400x).tiff]

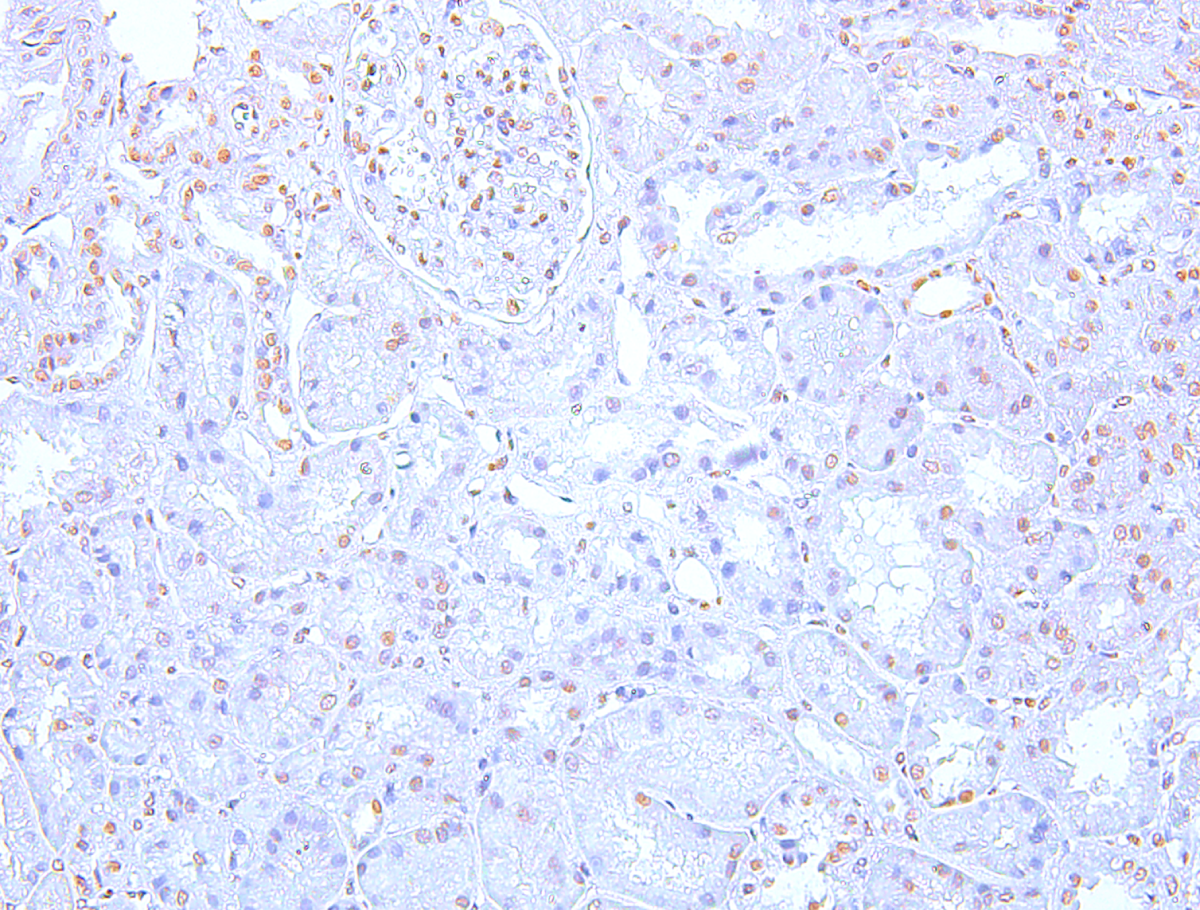

Supplement: Supplementary file 3 — Source data Fig. 1 [file 44321_2025_243_MOESM3_ESM.zip › 1A/A MCD(400x).tiff]

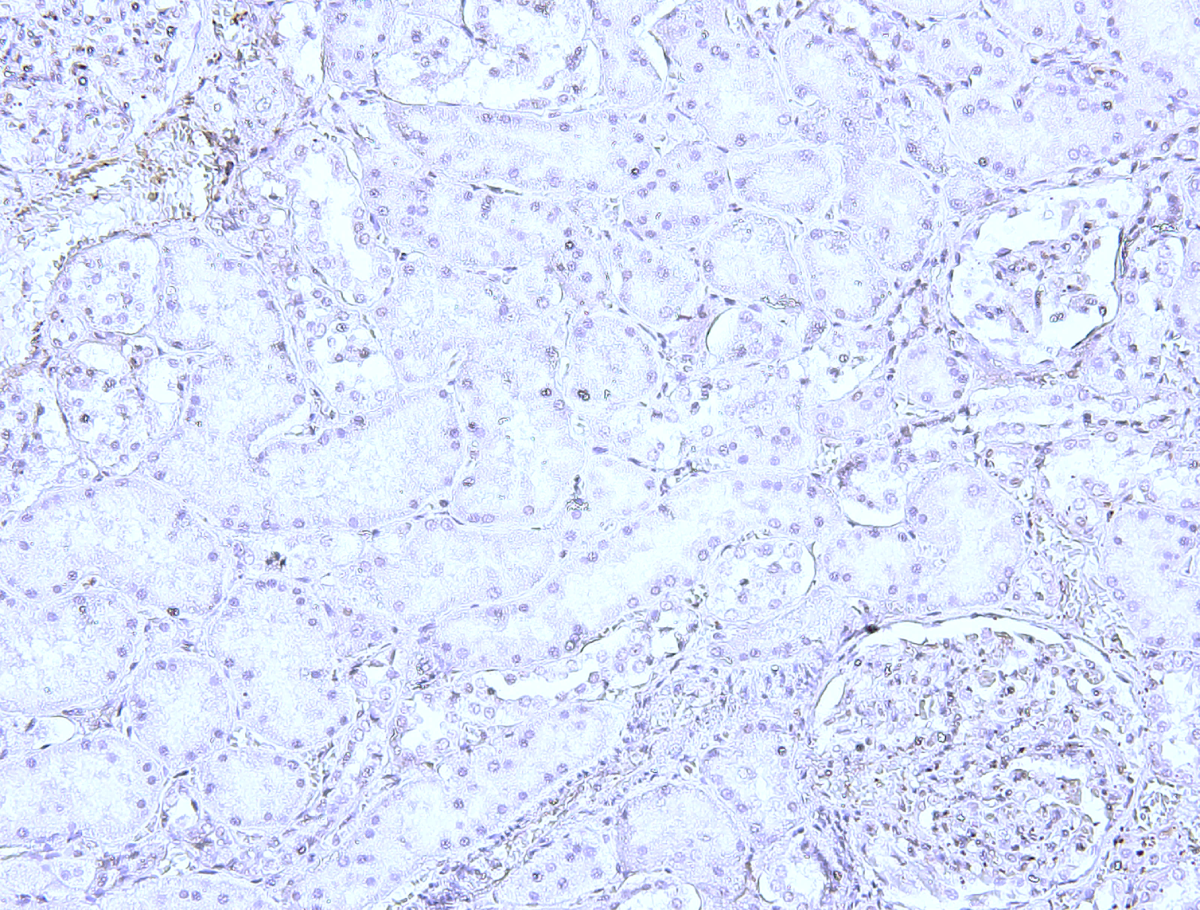

Supplement: Supplementary file 3 — Source data Fig. 1 [file 44321_2025_243_MOESM3_ESM.zip › 1A/A Normal(400x).tiff]

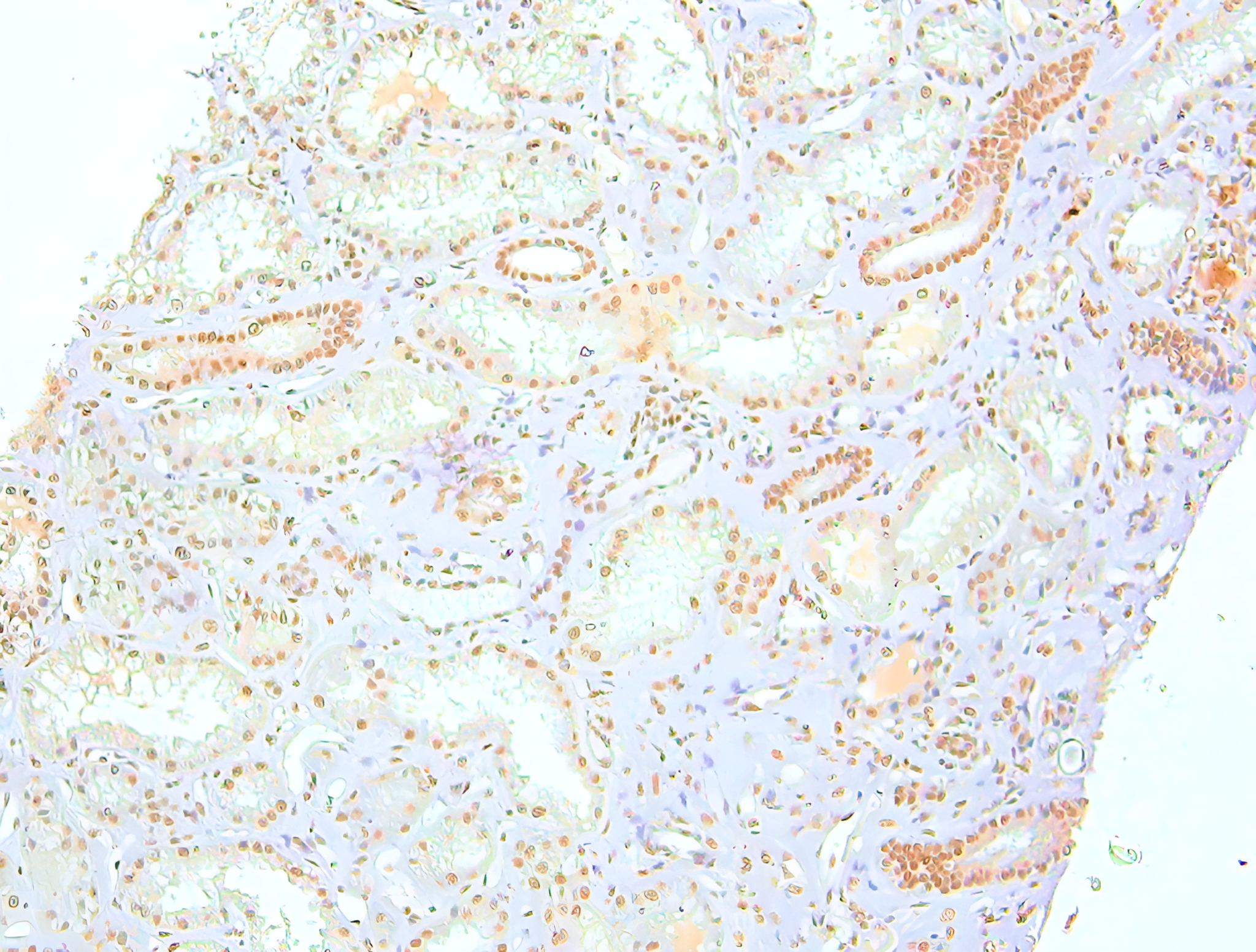

Supplement: Supplementary file 3 — Source data Fig. 1 [file 44321_2025_243_MOESM3_ESM.zip › 1A/A_FSGS(200x).tif]

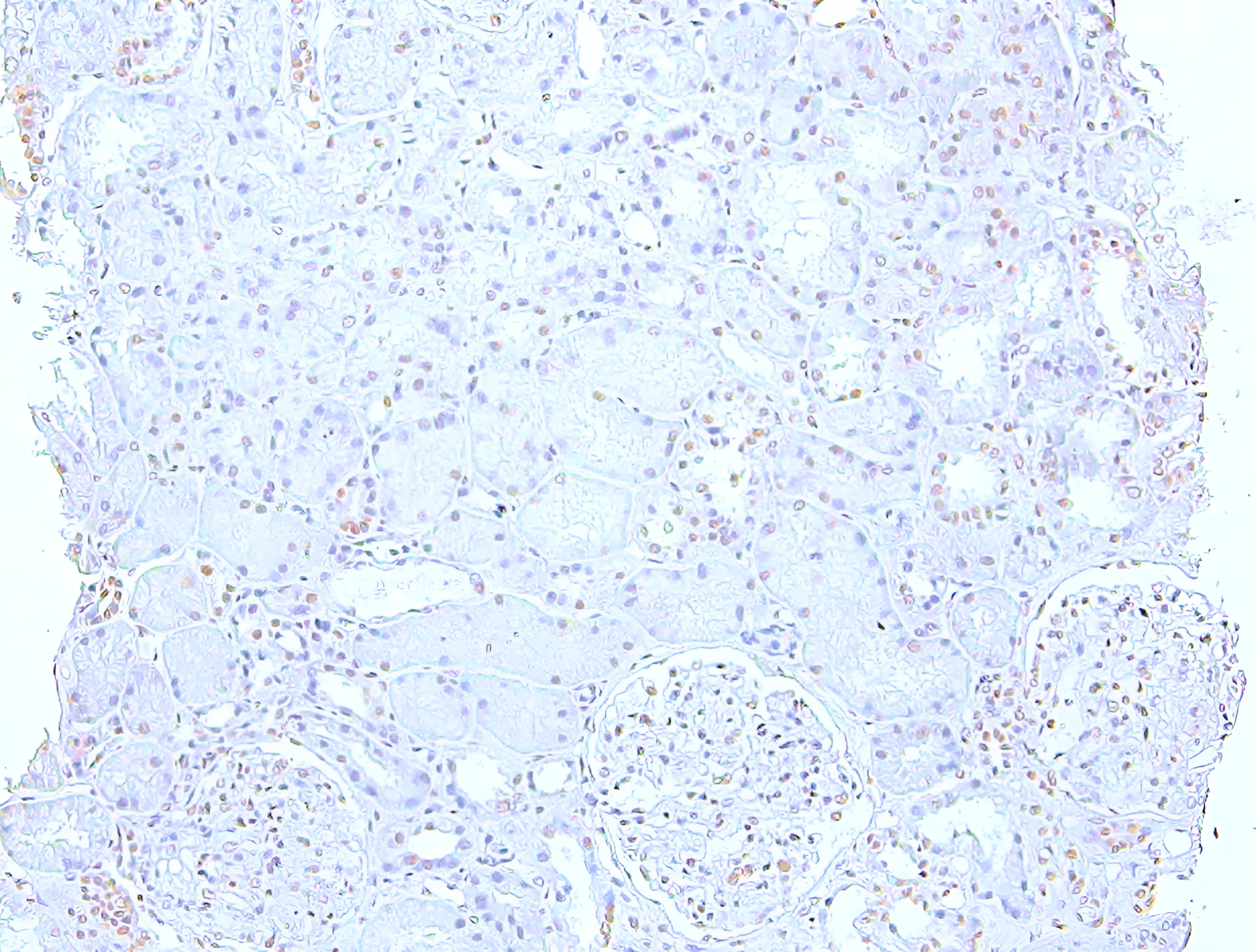

Supplement: Supplementary file 3 — Source data Fig. 1 [file 44321_2025_243_MOESM3_ESM.zip › 1A/A_MCD(200x).tif]

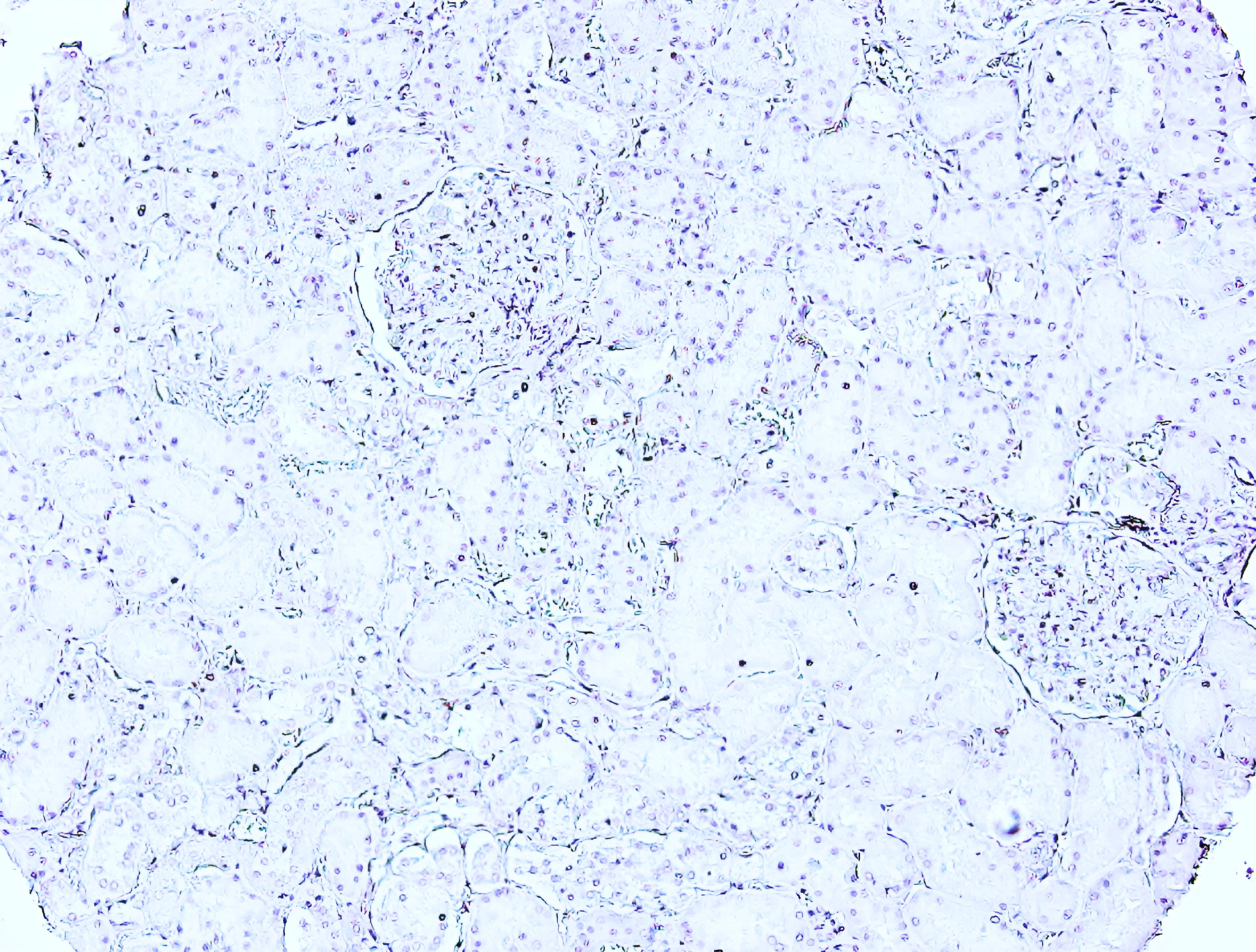

Supplement: Supplementary file 3 — Source data Fig. 1 [file 44321_2025_243_MOESM3_ESM.zip › 1A/A_Normal_(200x).tif]

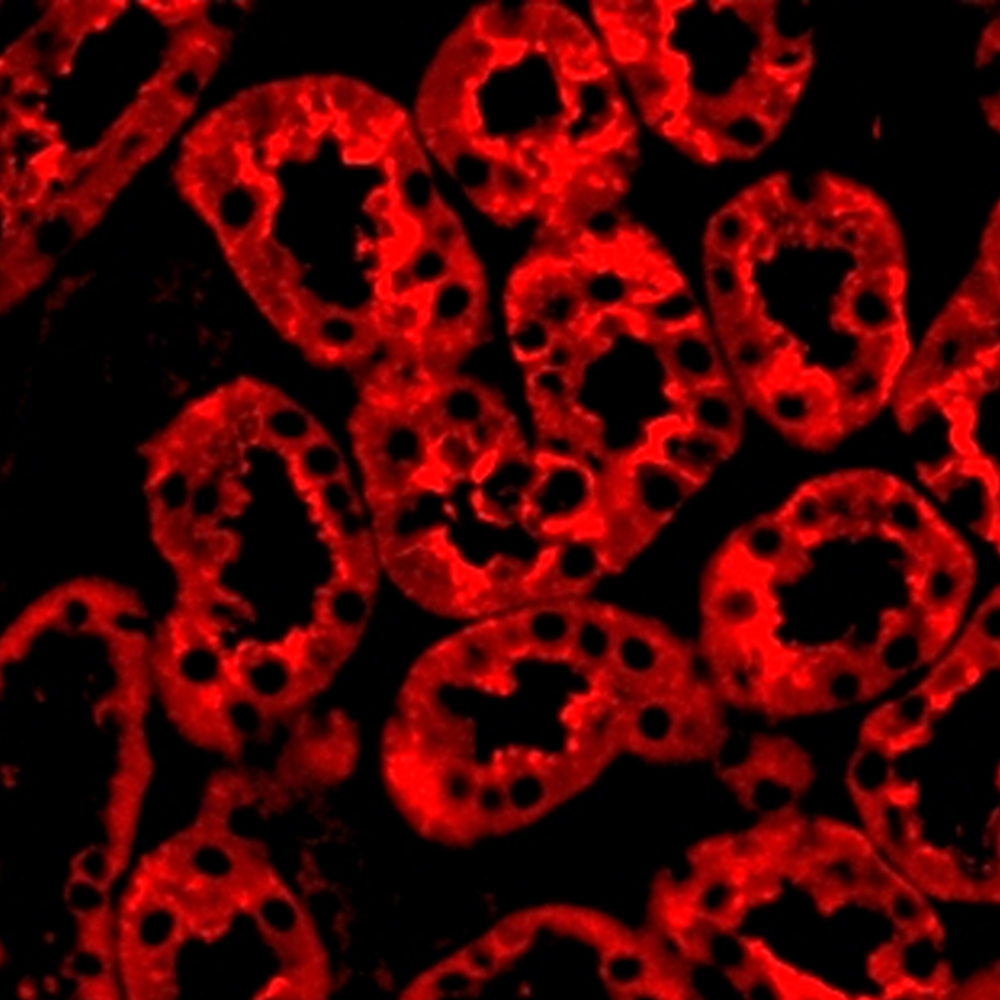

Supplement: Supplementary file 3 — Source data Fig. 1 [file 44321_2025_243_MOESM3_ESM.zip › 1D/Sham AQP1.tiff]

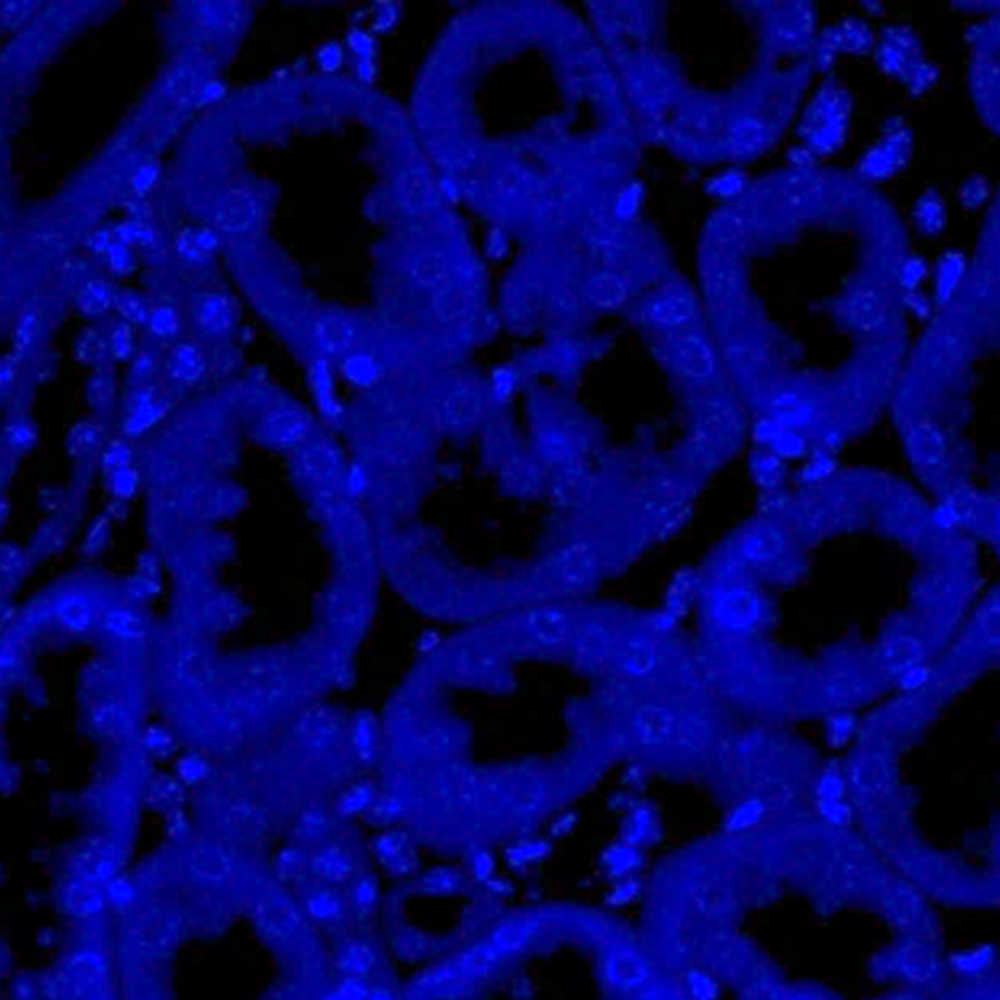

Supplement: Supplementary file 3 — Source data Fig. 1 [file 44321_2025_243_MOESM3_ESM.zip › 1D/Sham DAPI.tiff]

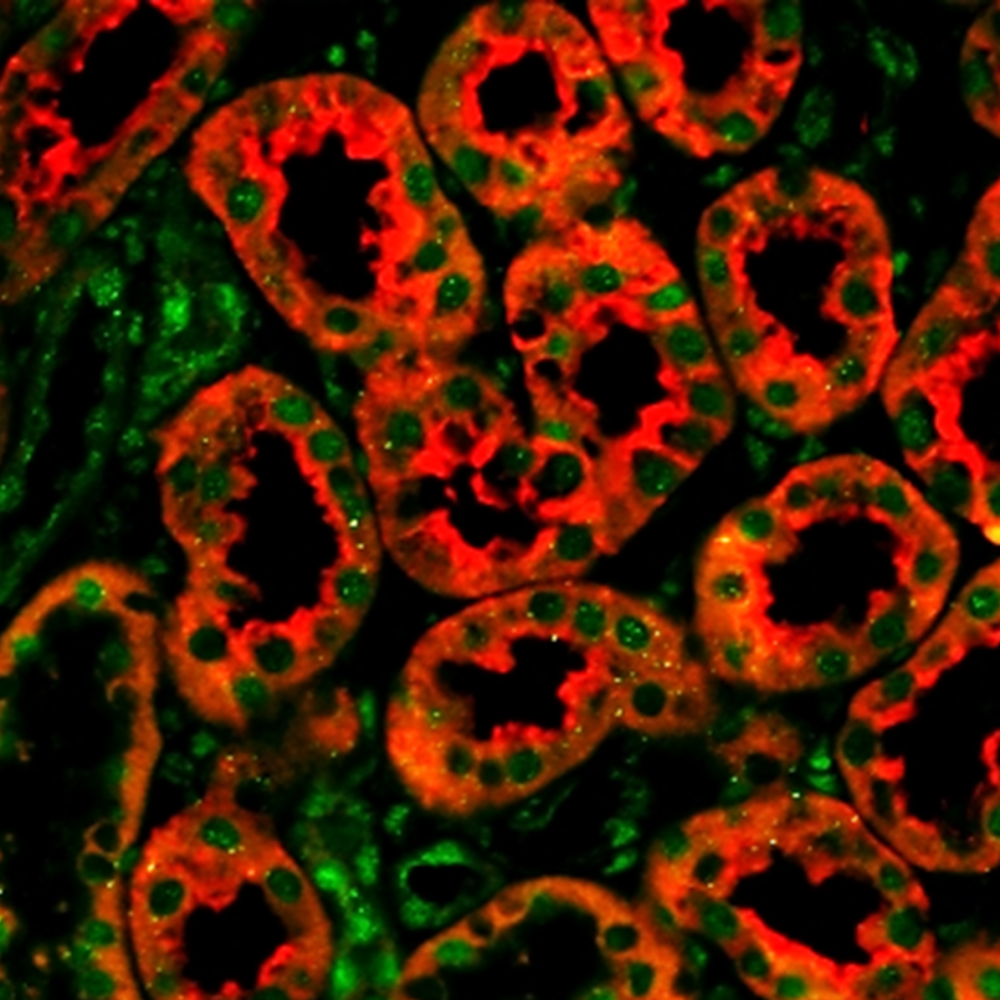

Supplement: Supplementary file 3 — Source data Fig. 1 [file 44321_2025_243_MOESM3_ESM.zip › 1D/Sham Merge.tiff]

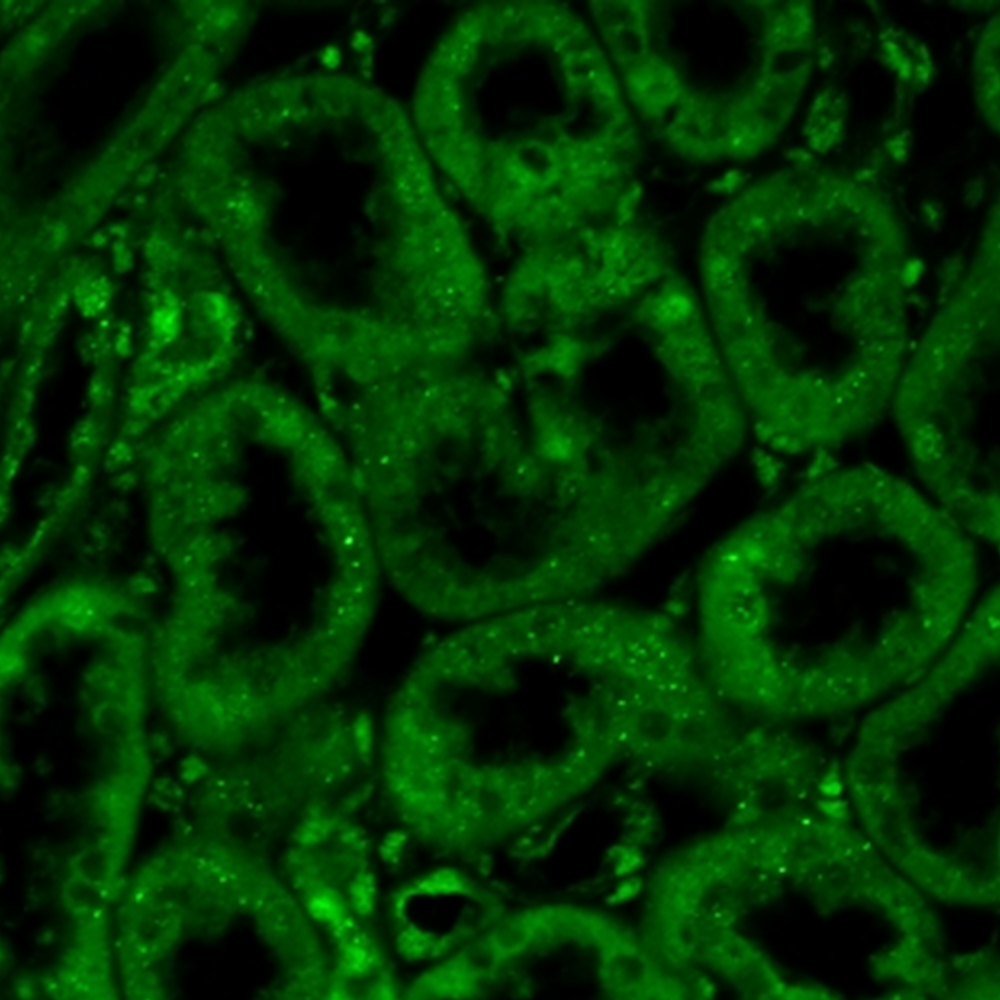

Supplement: Supplementary file 3 — Source data Fig. 1 [file 44321_2025_243_MOESM3_ESM.zip › 1D/Sham p300.tiff]

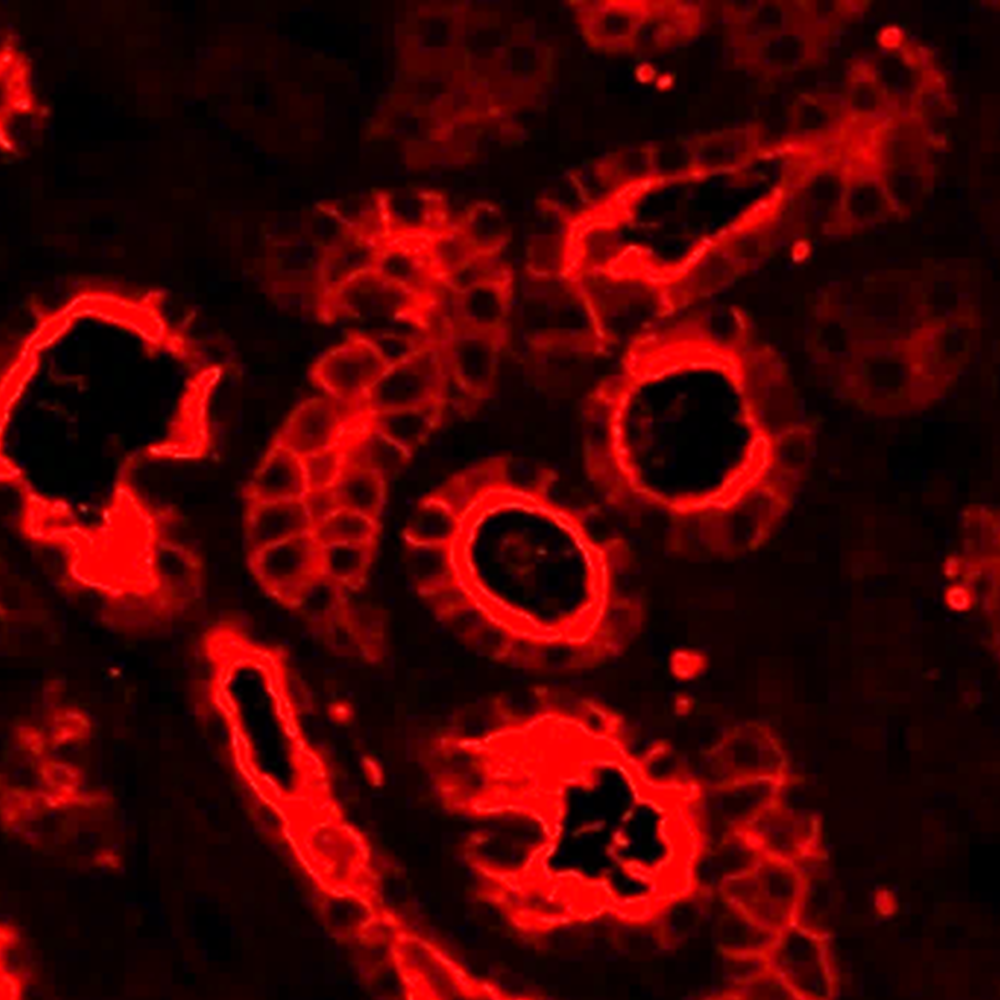

Supplement: Supplementary file 3 — Source data Fig. 1 [file 44321_2025_243_MOESM3_ESM.zip › 1D/UUO AQP1.tiff]

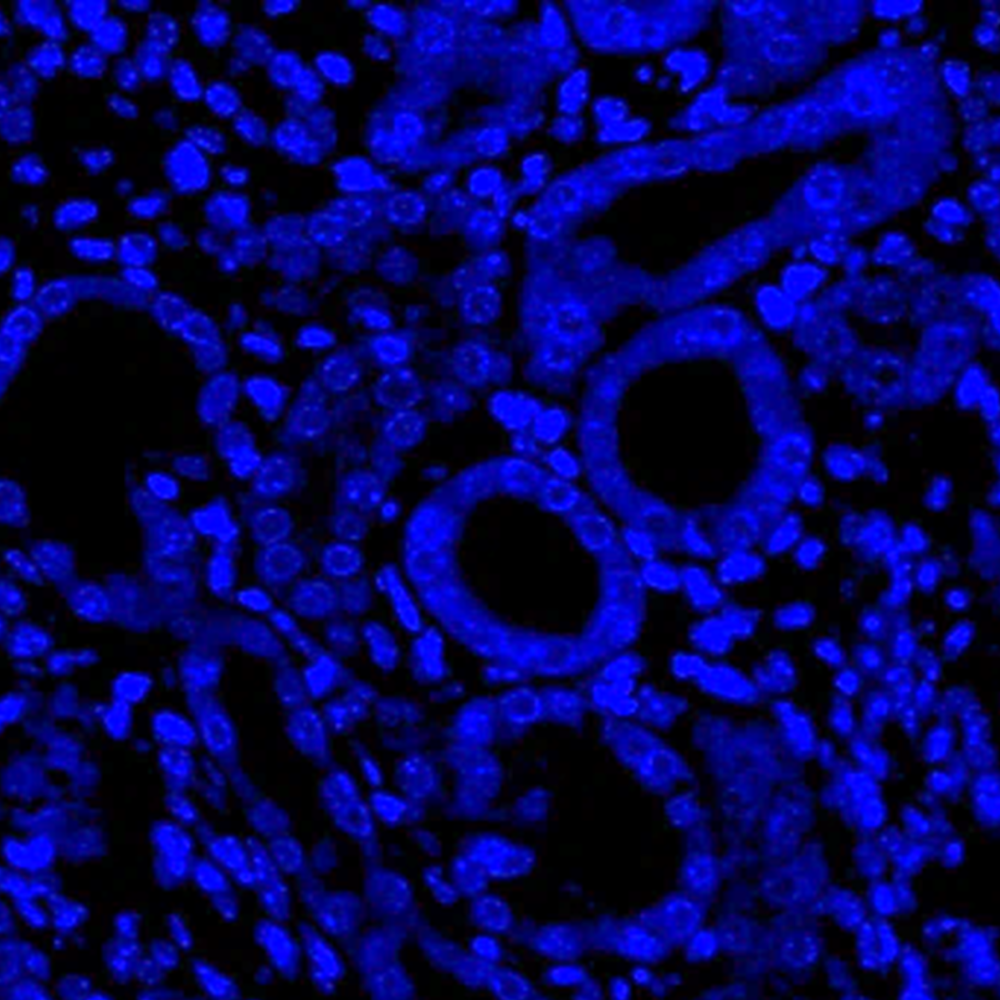

Supplement: Supplementary file 3 — Source data Fig. 1 [file 44321_2025_243_MOESM3_ESM.zip › 1D/UUO DAPI.tiff]

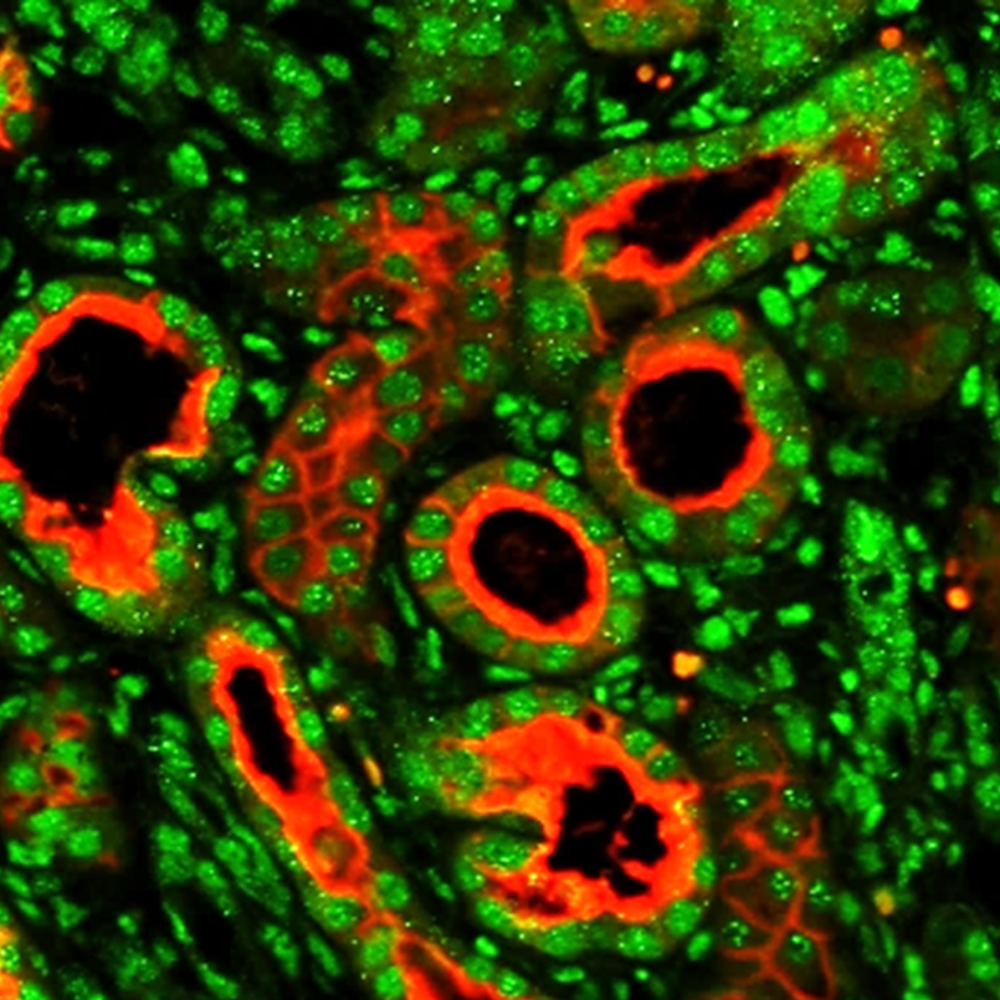

Supplement: Supplementary file 3 — Source data Fig. 1 [file 44321_2025_243_MOESM3_ESM.zip › 1D/UUO Merge.tiff]

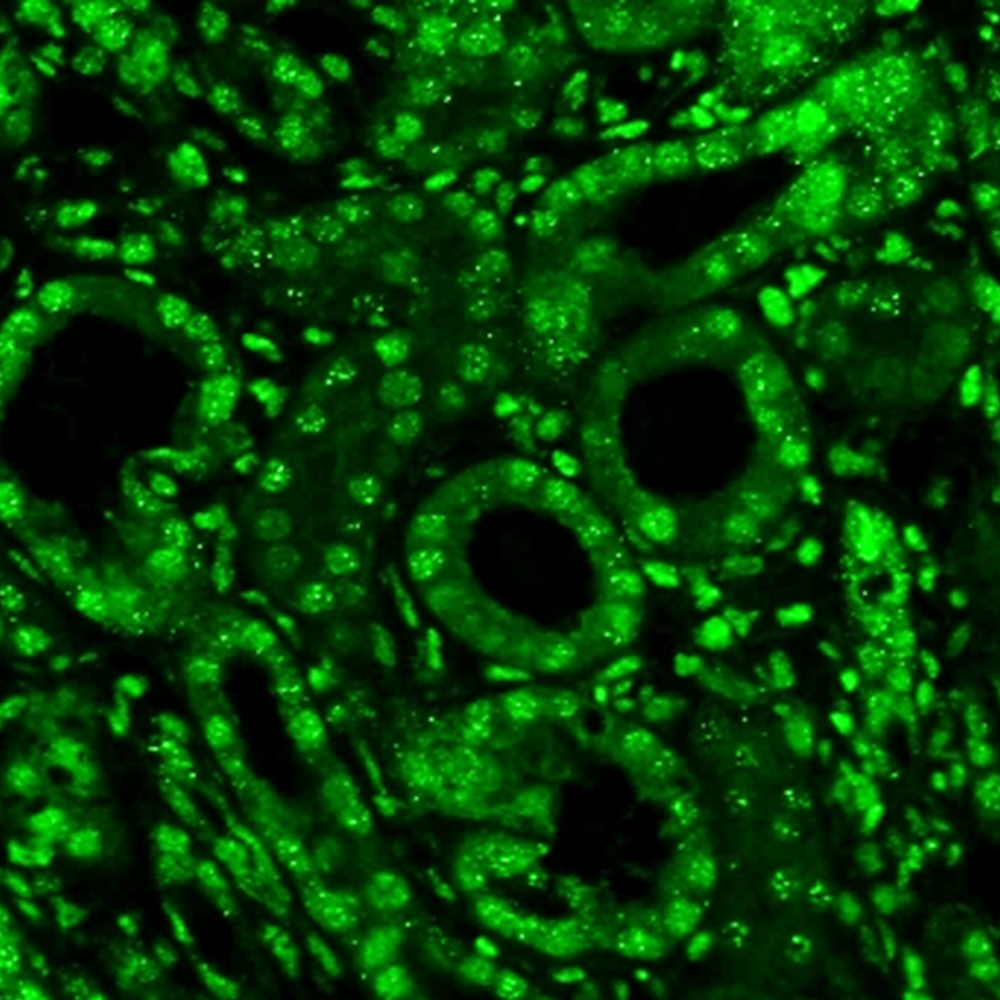

Supplement: Supplementary file 3 — Source data Fig. 1 [file 44321_2025_243_MOESM3_ESM.zip › 1D/UUO p300.tiff]

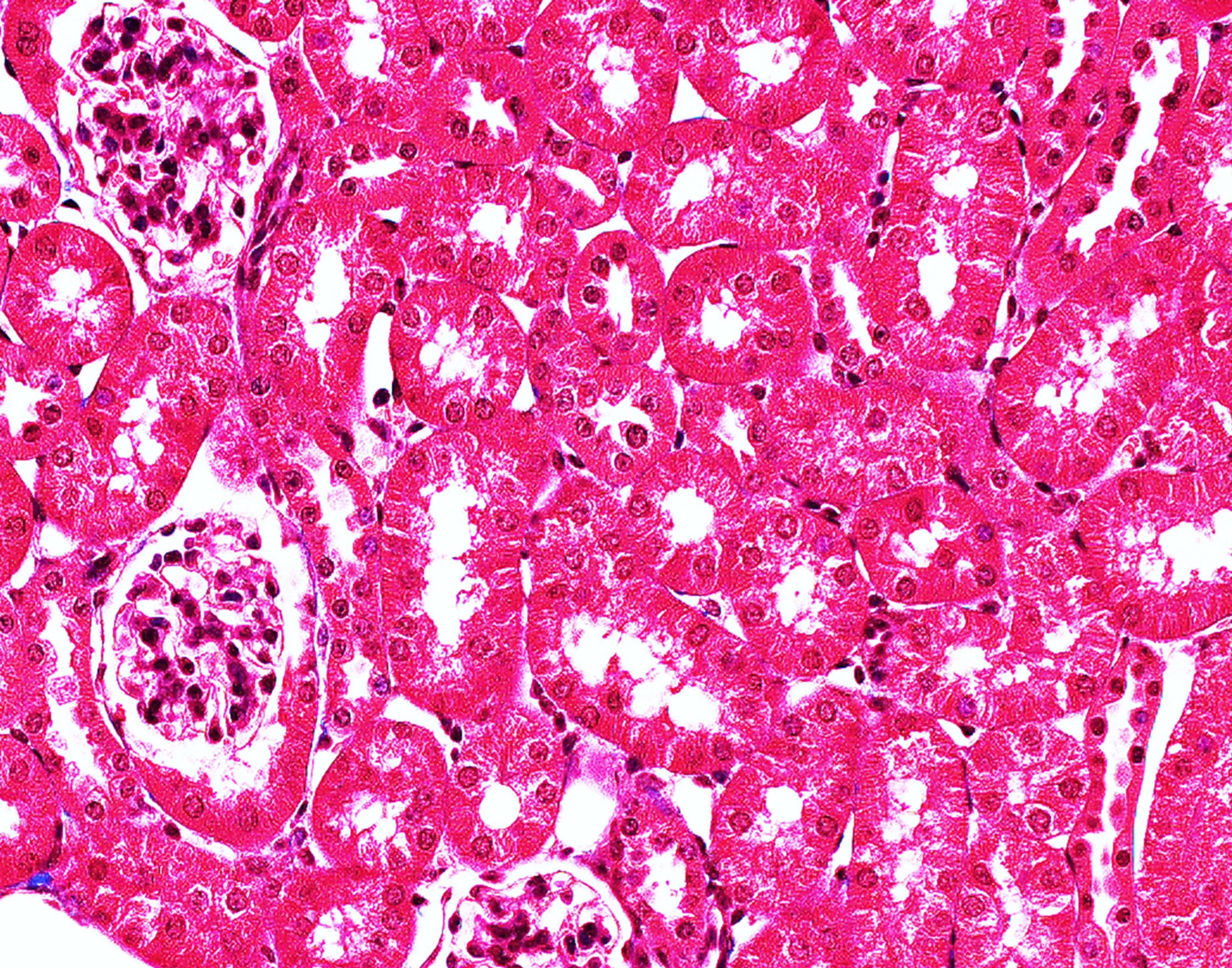

Supplement: Supplementary file 3 — Source data Fig. 1 [file 44321_2025_243_MOESM3_ESM.zip › 1F/MTS Sham KO.tiff]

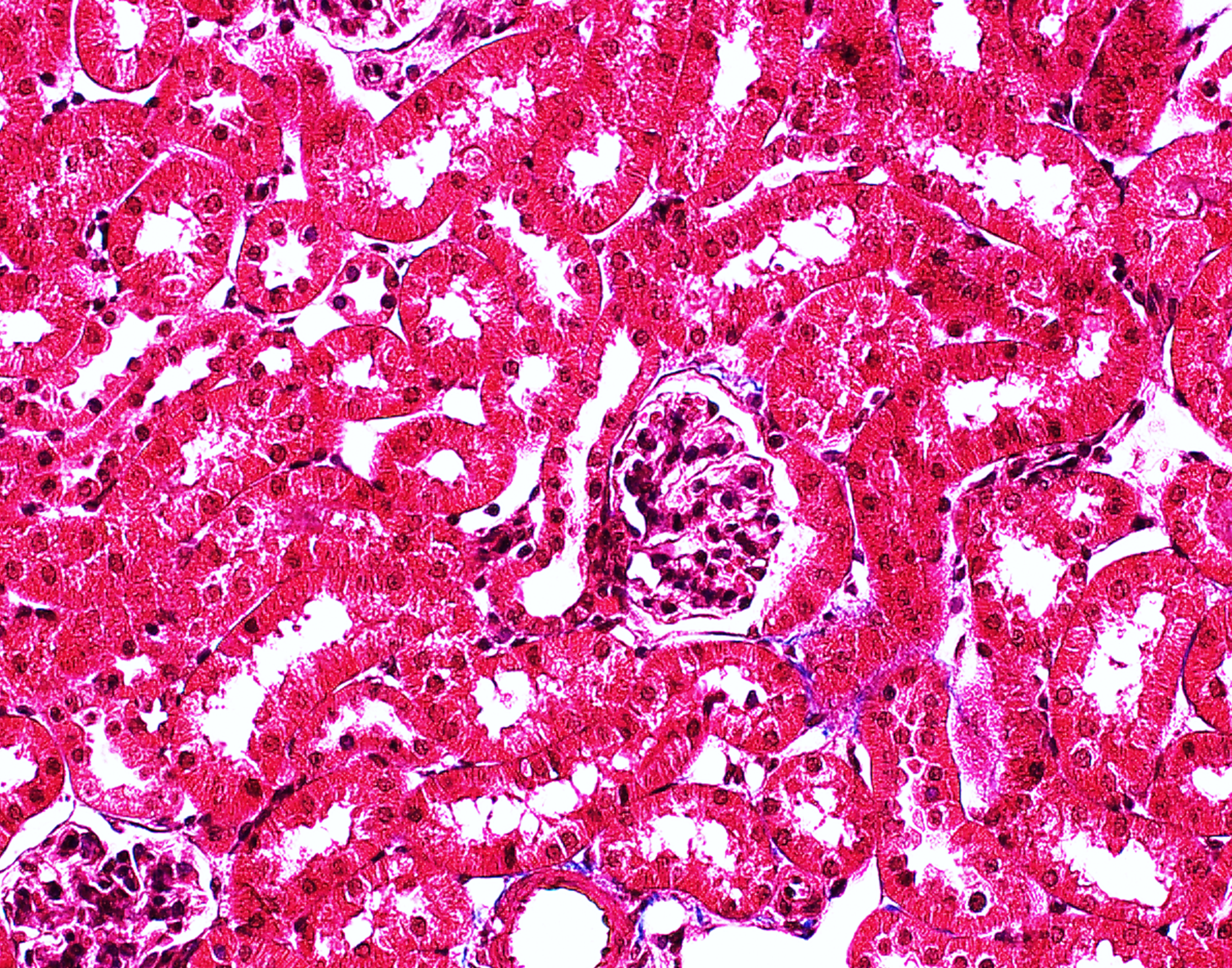

Supplement: Supplementary file 3 — Source data Fig. 1 [file 44321_2025_243_MOESM3_ESM.zip › 1F/MTS Sham WT.tiff]

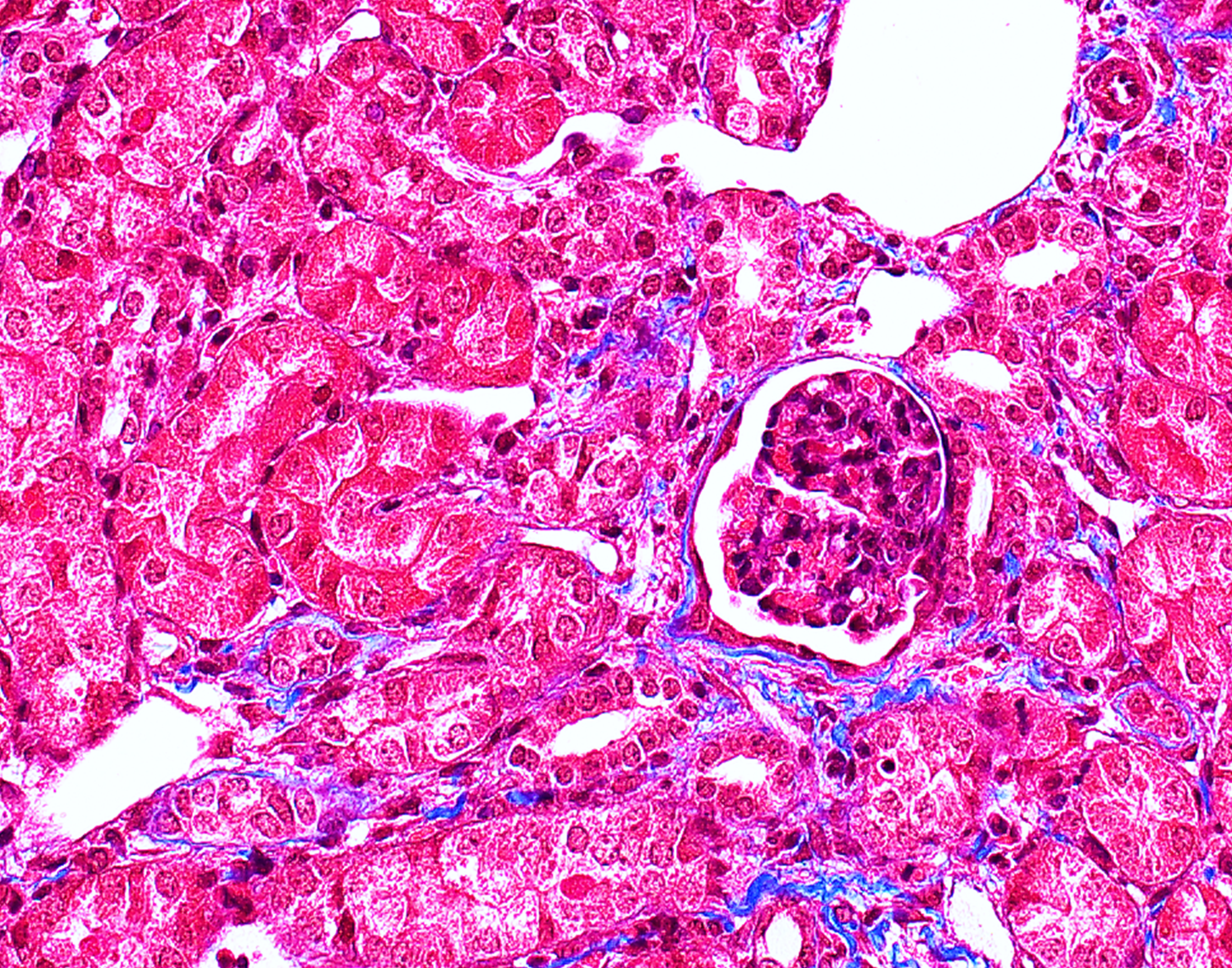

Supplement: Supplementary file 3 — Source data Fig. 1 [file 44321_2025_243_MOESM3_ESM.zip › 1F/MTS UUO KO.tiff]

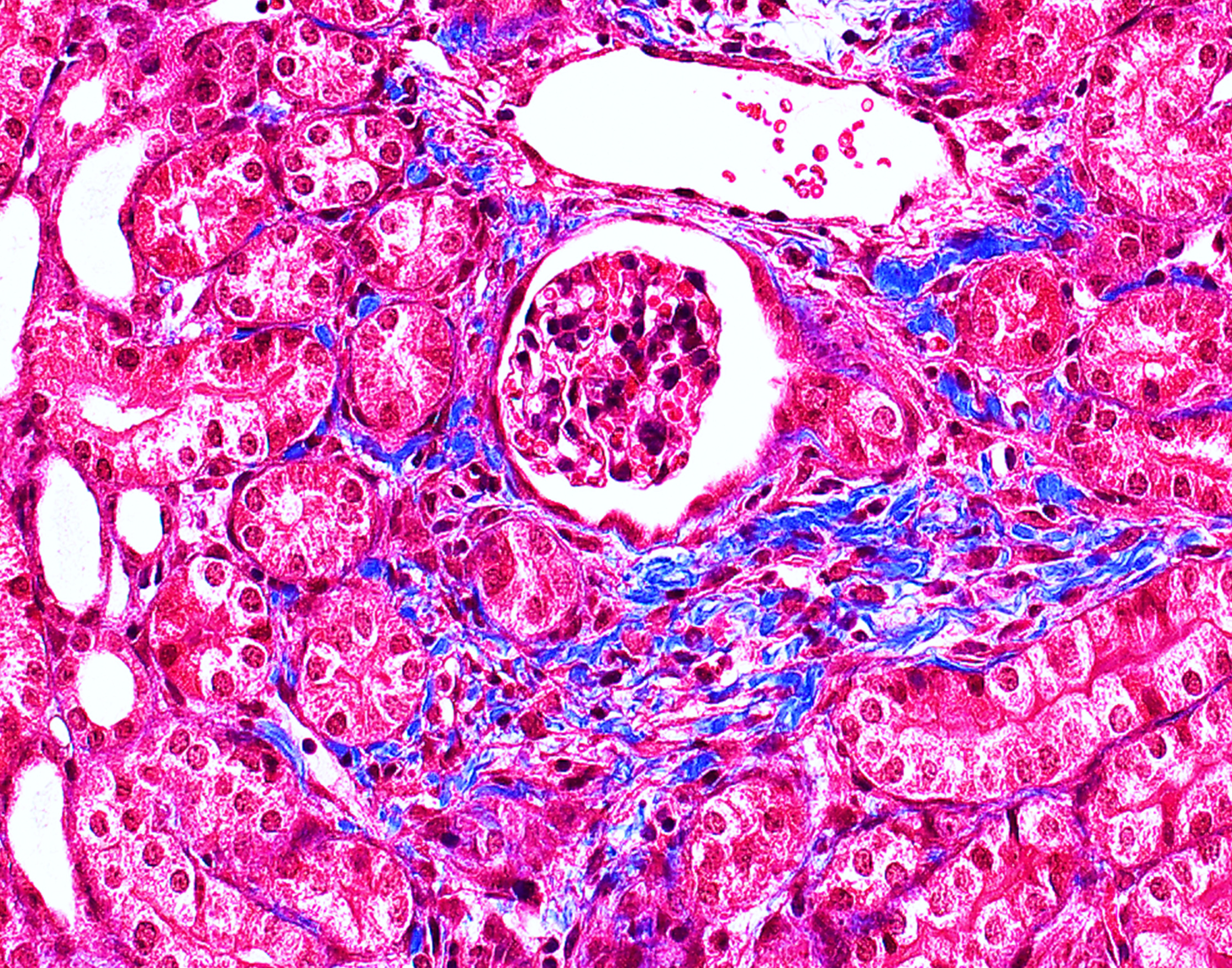

Supplement: Supplementary file 3 — Source data Fig. 1 [file 44321_2025_243_MOESM3_ESM.zip › 1F/MTS UUO WT.tiff]

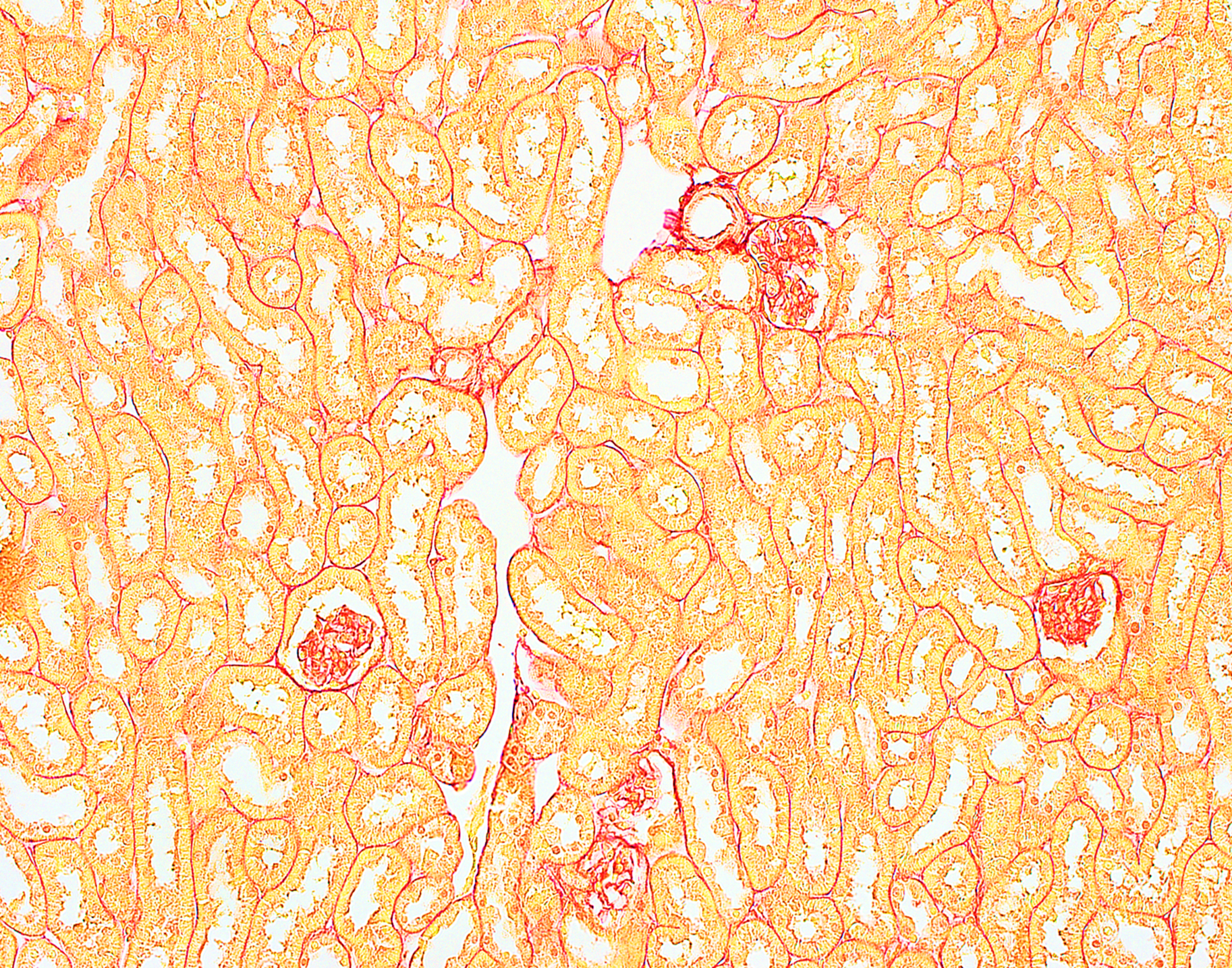

Supplement: Supplementary file 3 — Source data Fig. 1 [file 44321_2025_243_MOESM3_ESM.zip › 1F/Sirius red Sham KO.tiff]

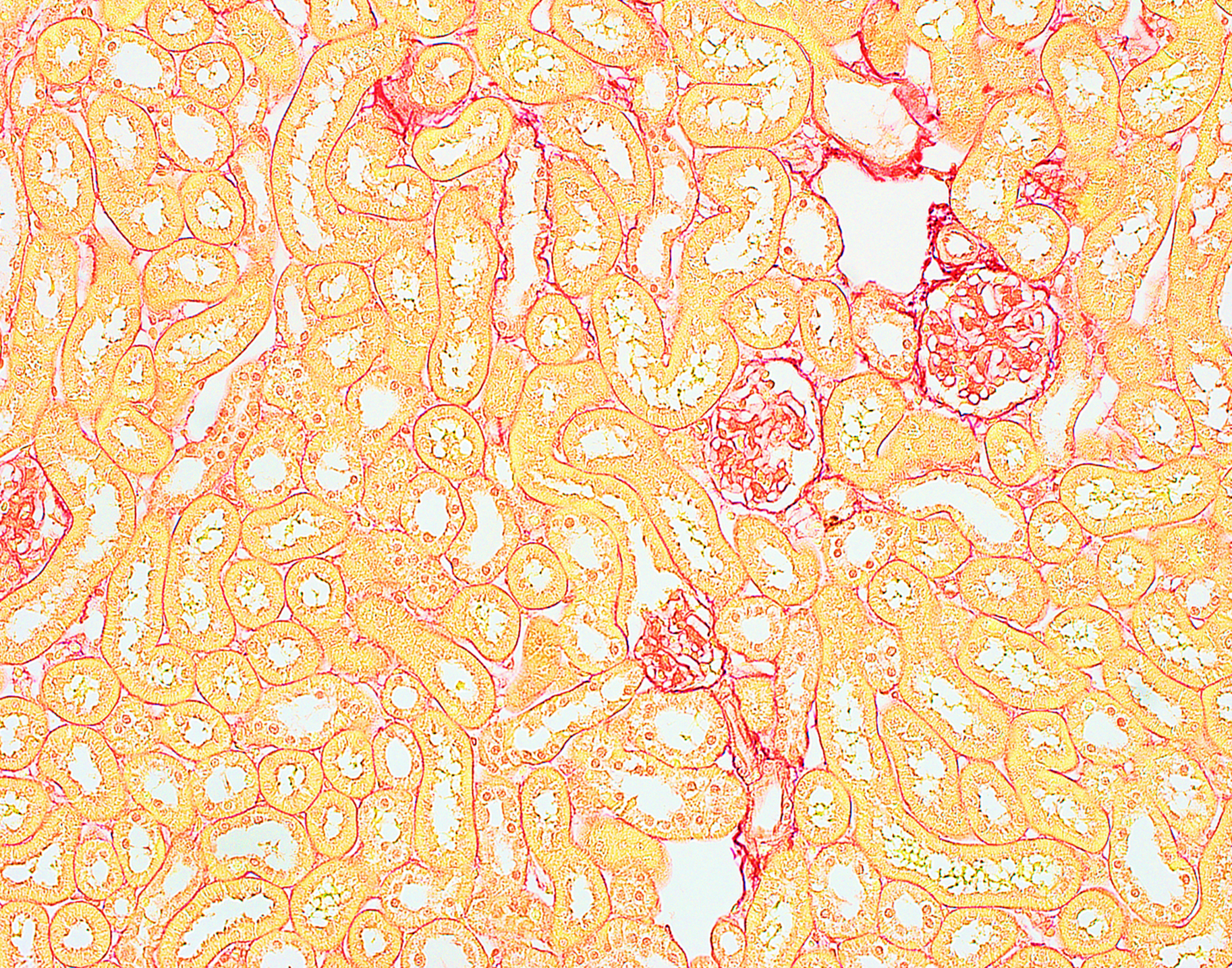

Supplement: Supplementary file 3 — Source data Fig. 1 [file 44321_2025_243_MOESM3_ESM.zip › 1F/Sirius red Sham WT.tiff]

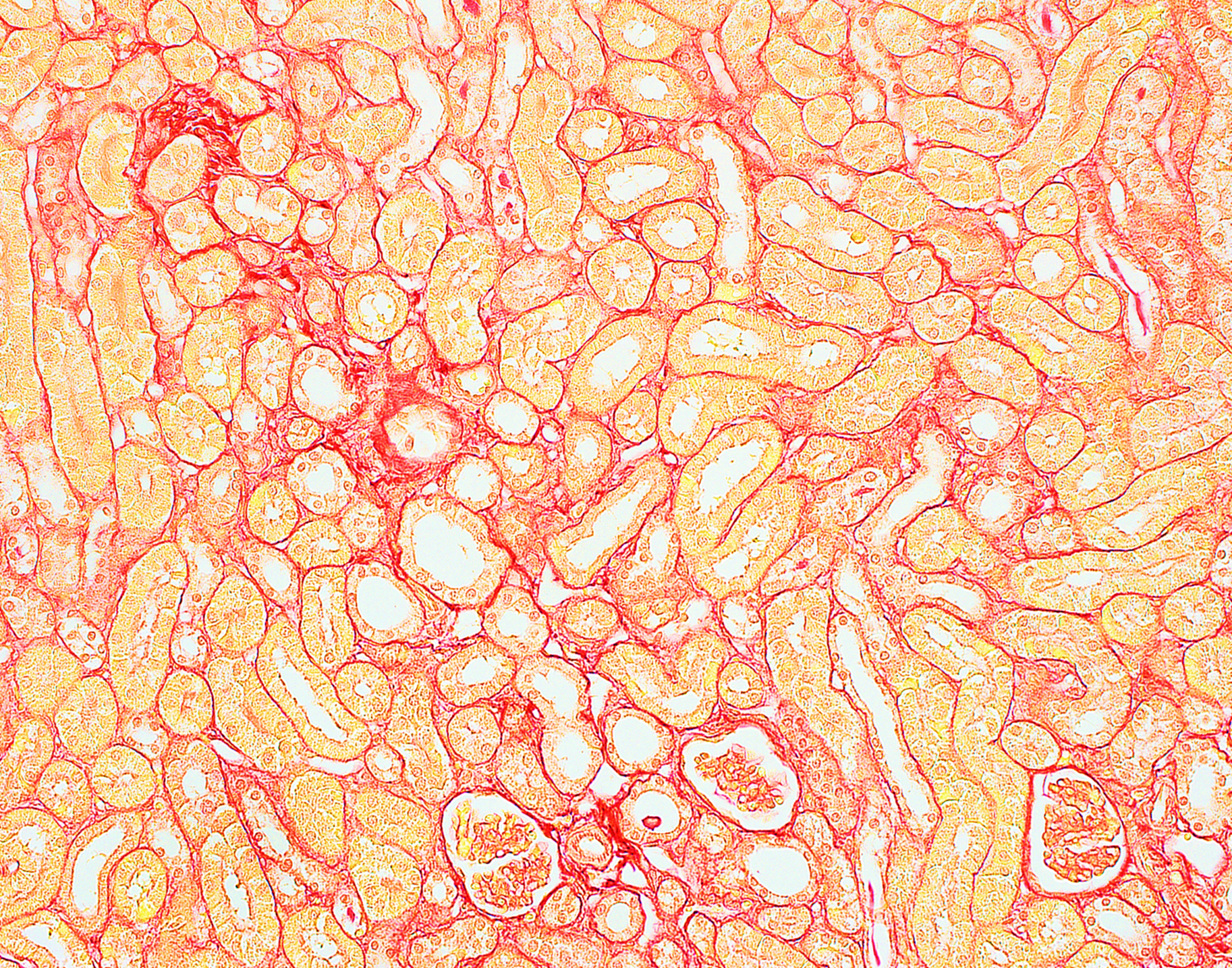

Supplement: Supplementary file 3 — Source data Fig. 1 [file 44321_2025_243_MOESM3_ESM.zip › 1F/Sirius red UUO KO.tiff]

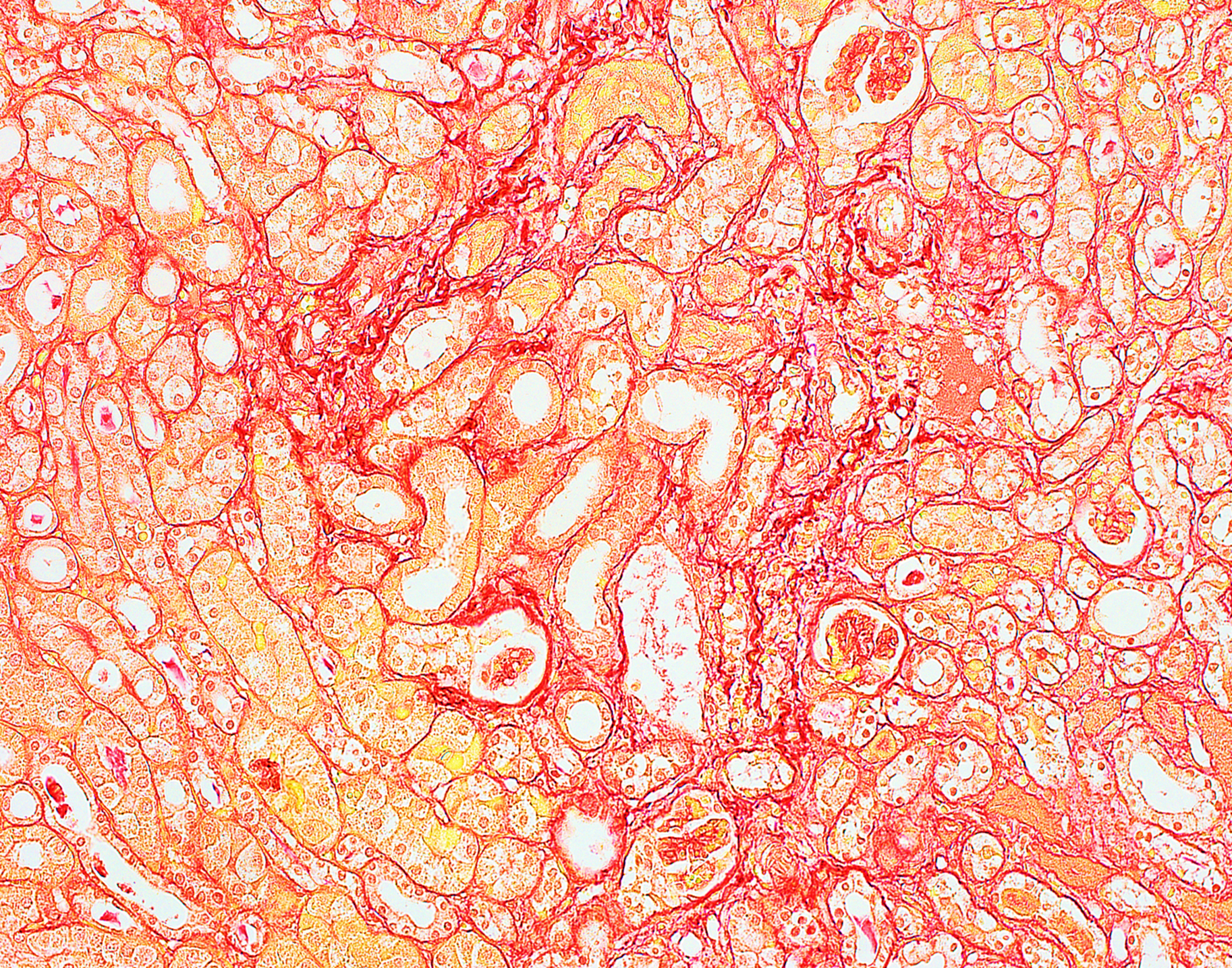

Supplement: Supplementary file 3 — Source data Fig. 1 [file 44321_2025_243_MOESM3_ESM.zip › 1F/Sirius red UUO WT.tiff]

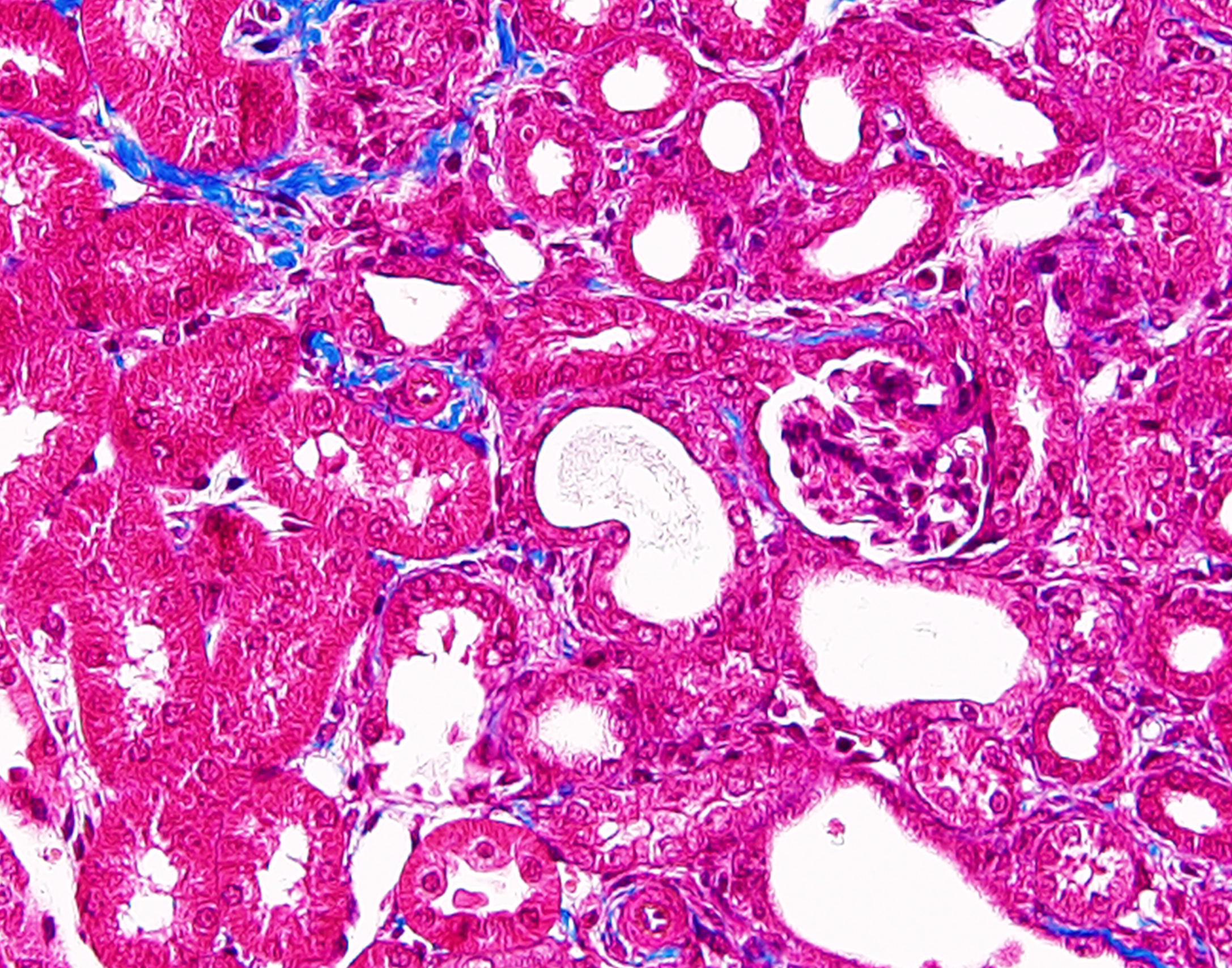

Supplement: Supplementary file 3 — Source data Fig. 1 [file 44321_2025_243_MOESM3_ESM.zip › 1H/MTS FA KO.tiff]

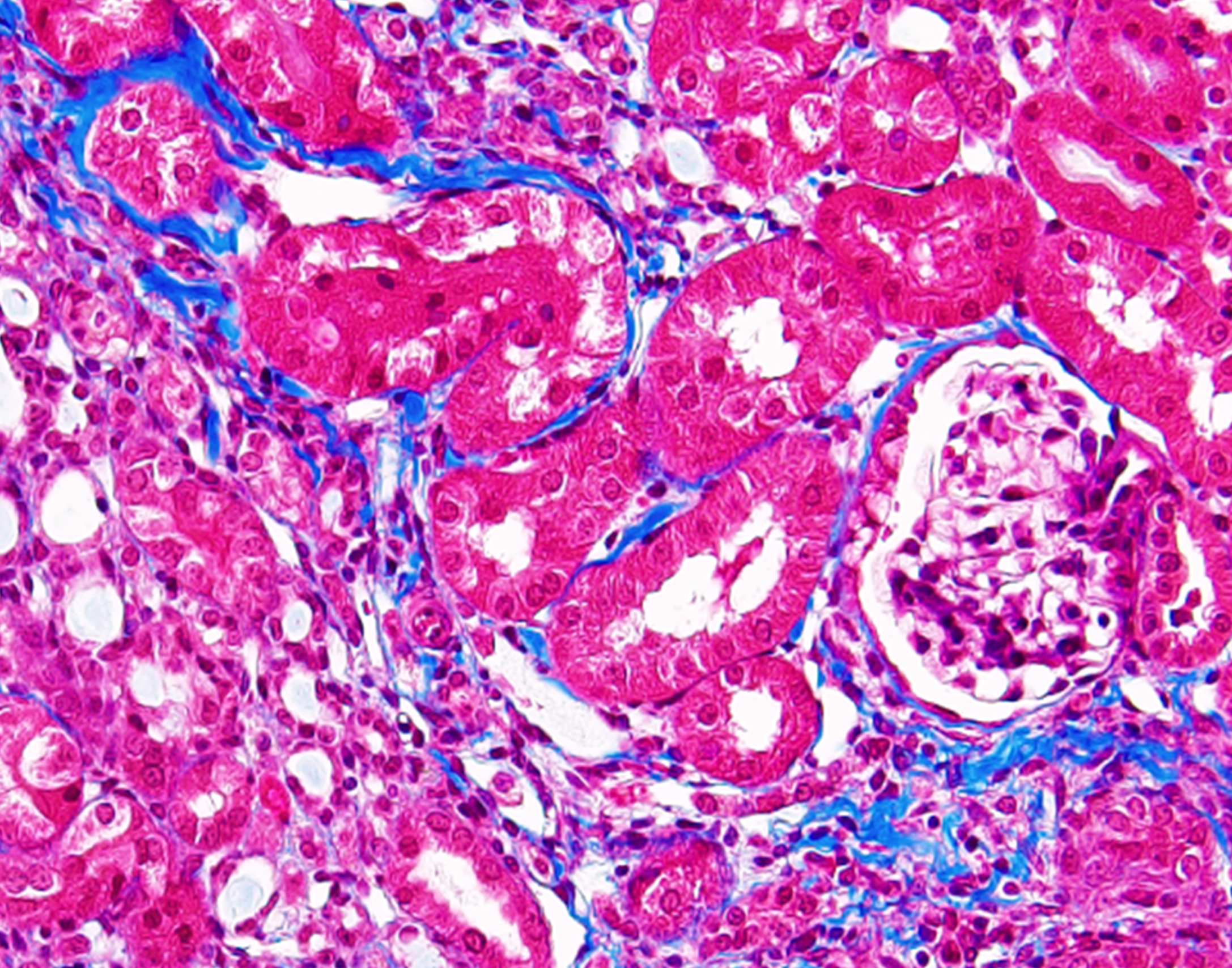

Supplement: Supplementary file 3 — Source data Fig. 1 [file 44321_2025_243_MOESM3_ESM.zip › 1H/MTS FA WT.tiff]

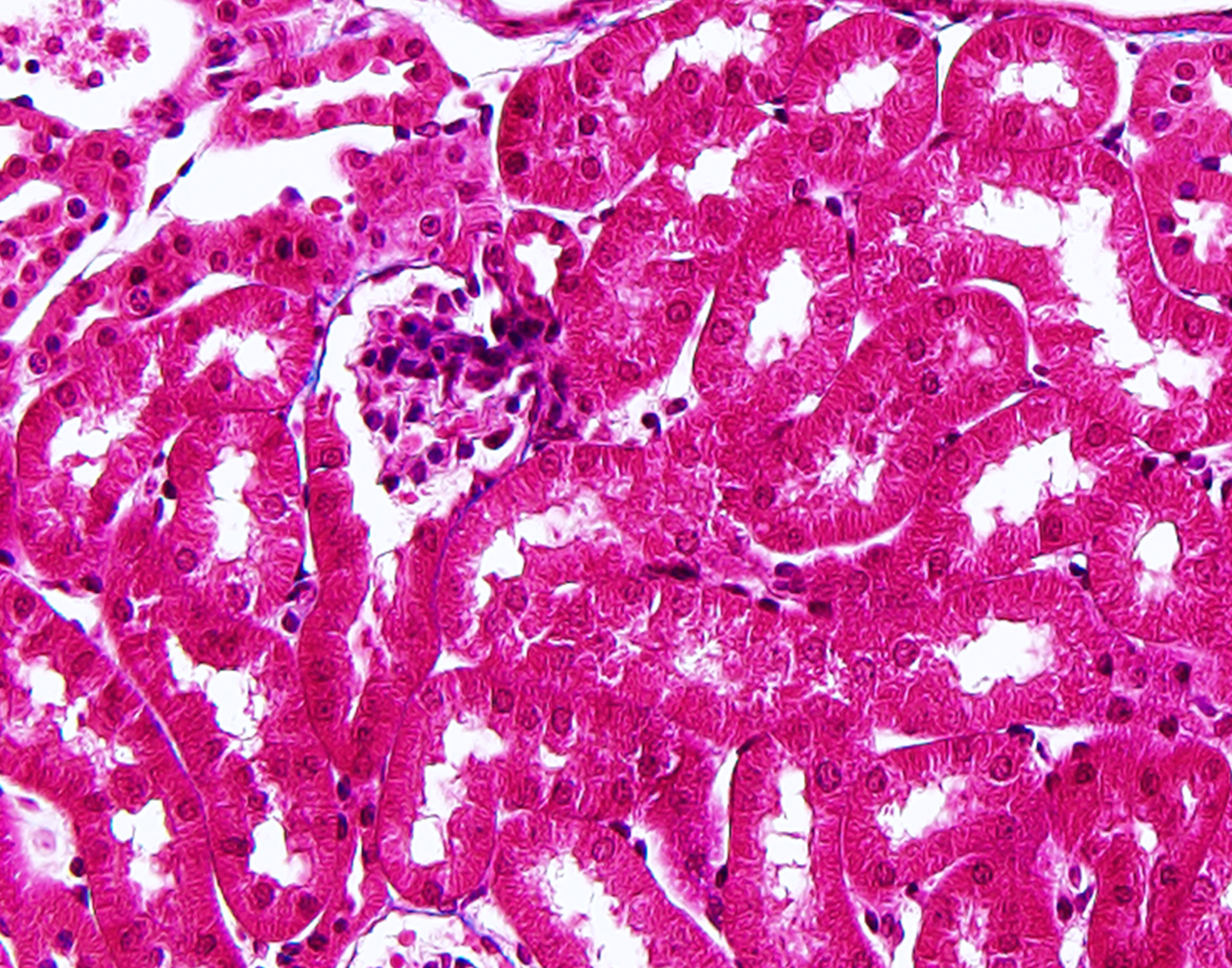

Supplement: Supplementary file 3 — Source data Fig. 1 [file 44321_2025_243_MOESM3_ESM.zip › 1H/MTS Sham KO.tiff]

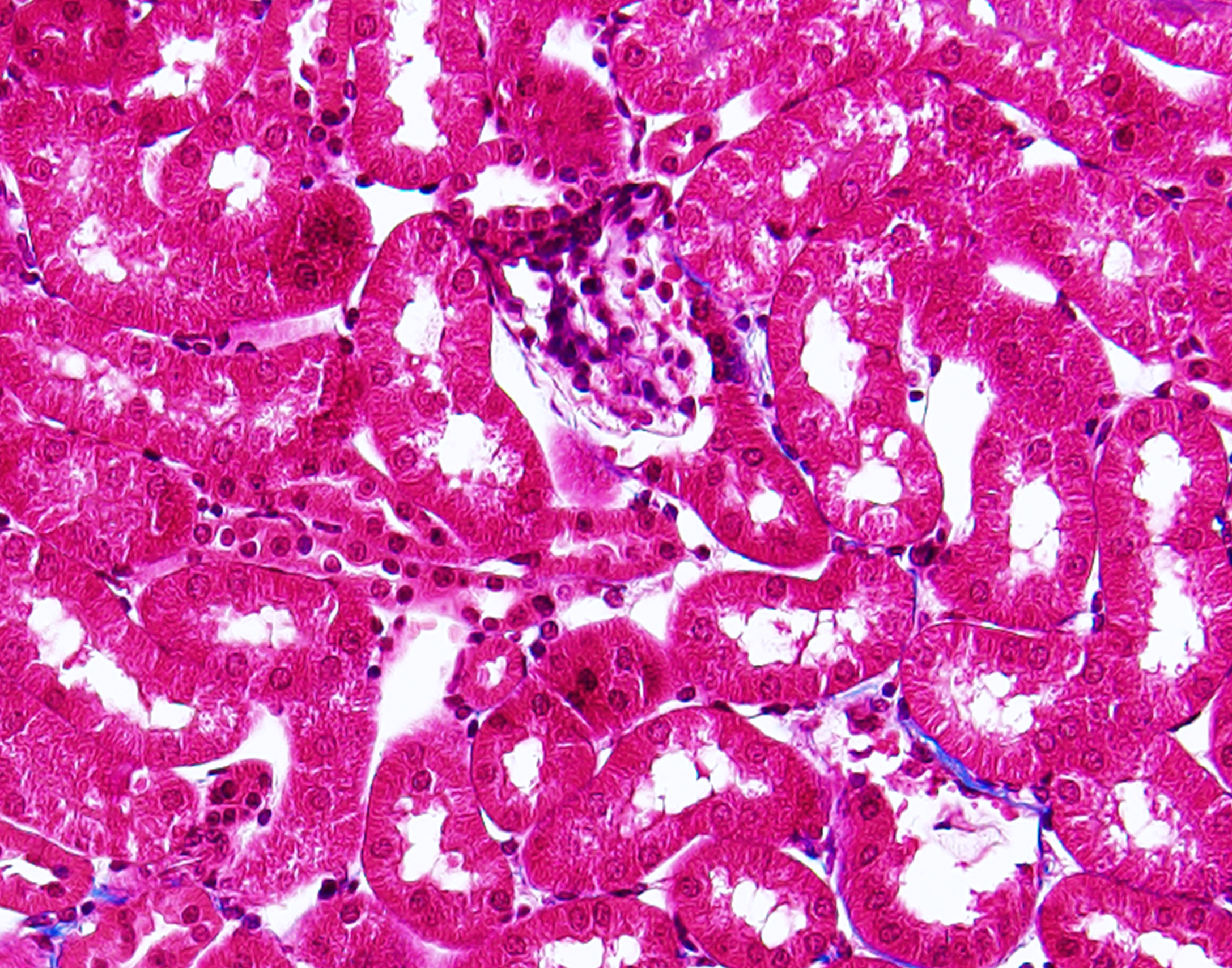

Supplement: Supplementary file 3 — Source data Fig. 1 [file 44321_2025_243_MOESM3_ESM.zip › 1H/MTS Sham WT.tiff]

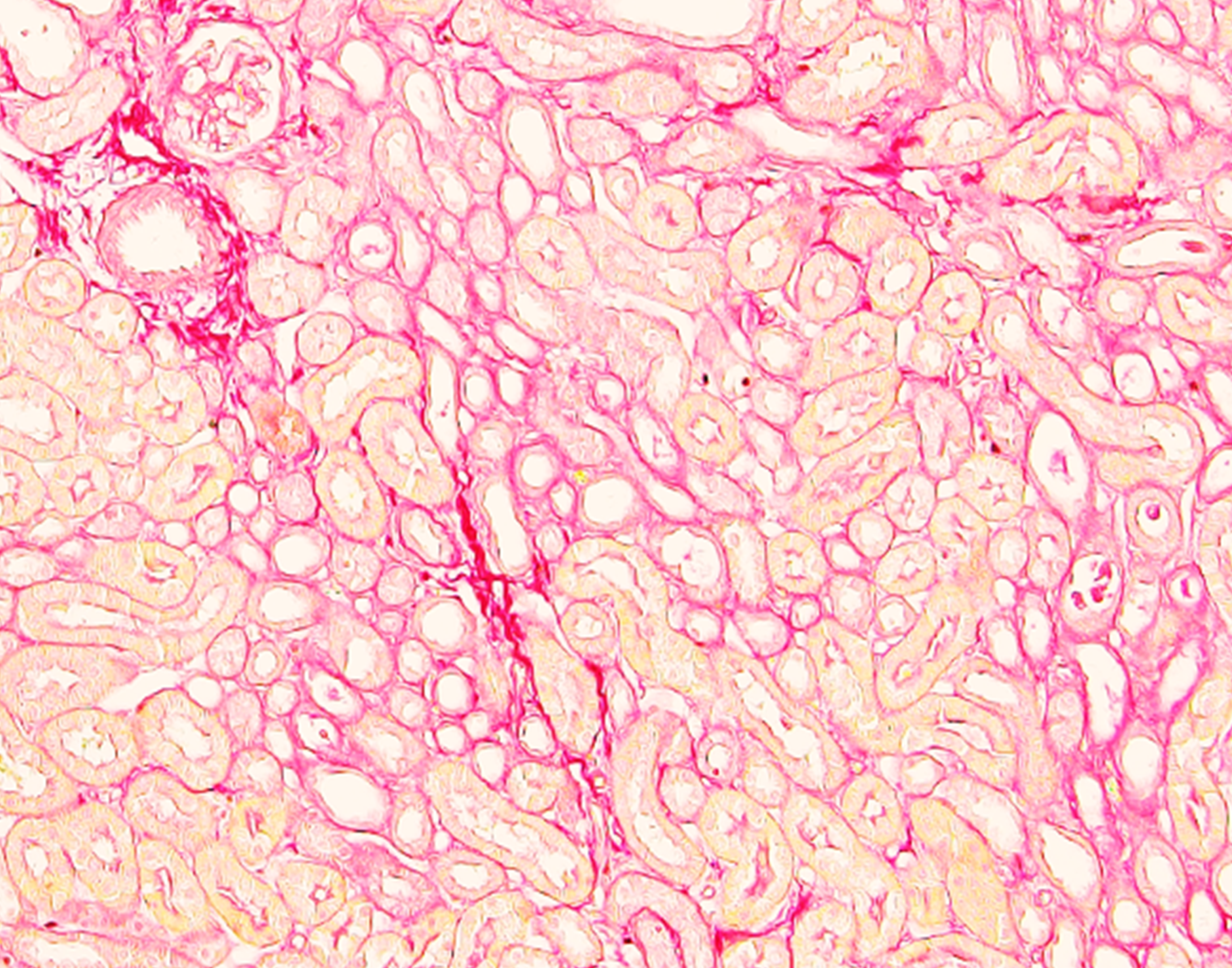

Supplement: Supplementary file 3 — Source data Fig. 1 [file 44321_2025_243_MOESM3_ESM.zip › 1H/Sirius red FA KO.tiff]

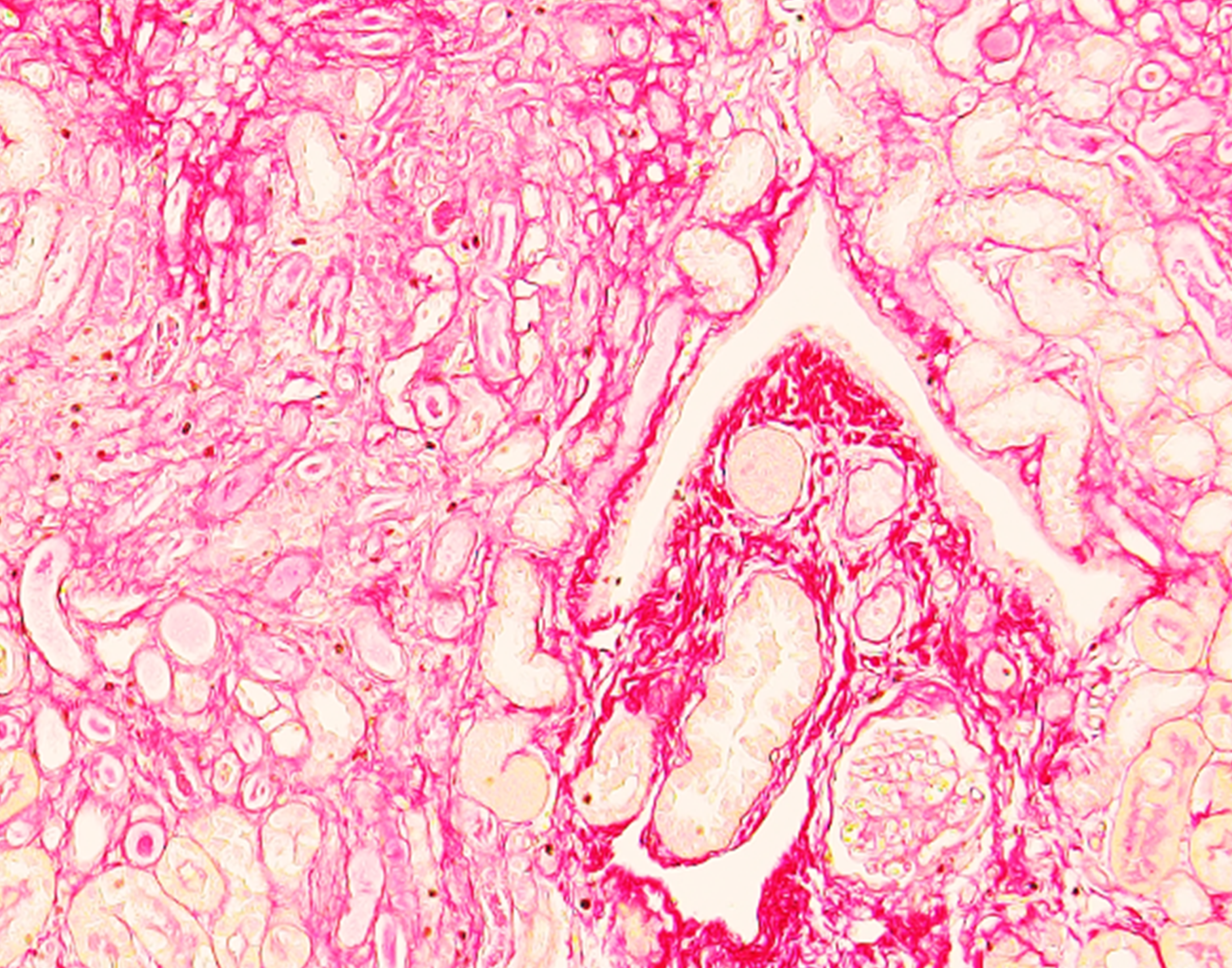

Supplement: Supplementary file 3 — Source data Fig. 1 [file 44321_2025_243_MOESM3_ESM.zip › 1H/Sirius red FA WT.tiff]

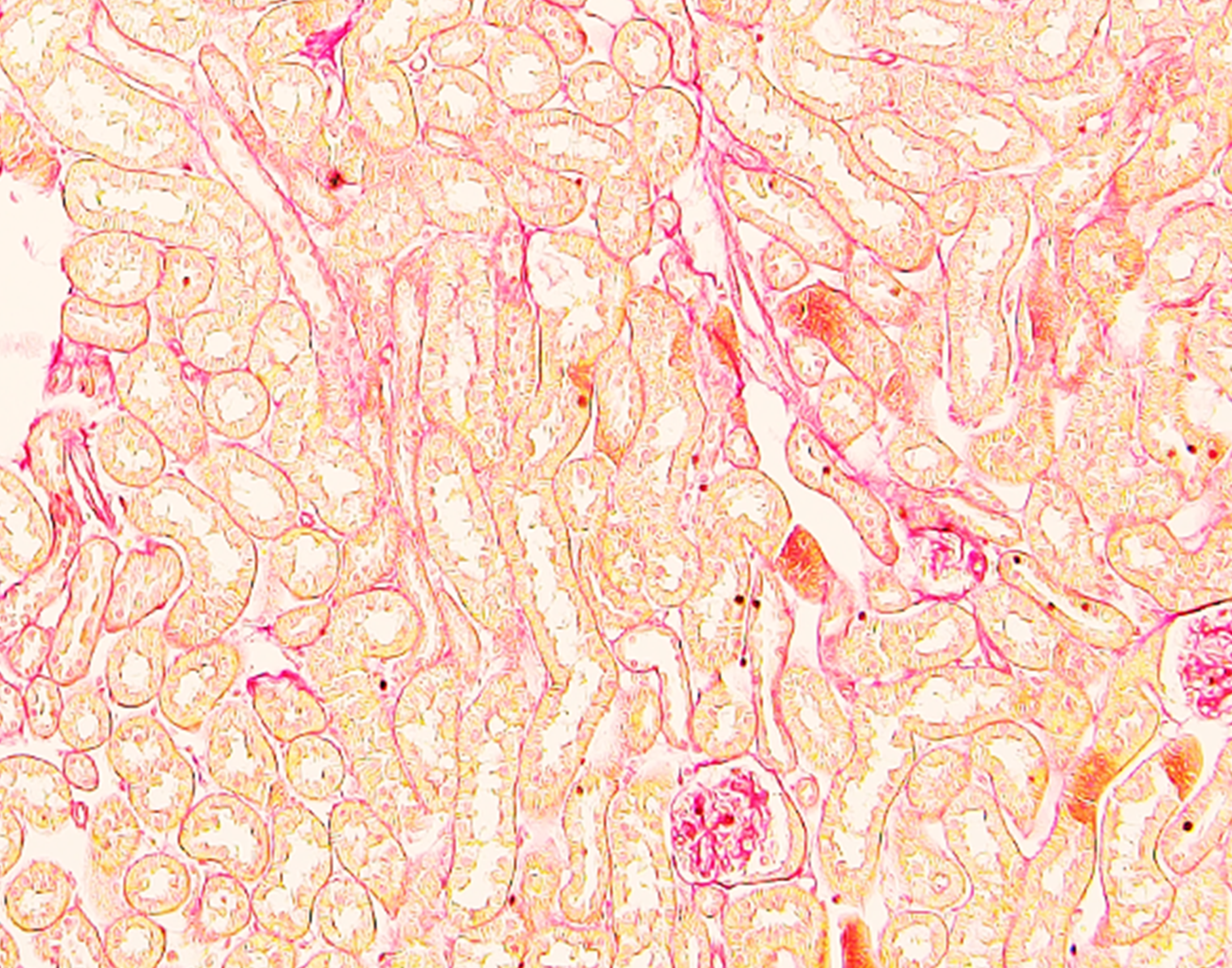

Supplement: Supplementary file 3 — Source data Fig. 1 [file 44321_2025_243_MOESM3_ESM.zip › 1H/Sirius red Sham KO.tiff]

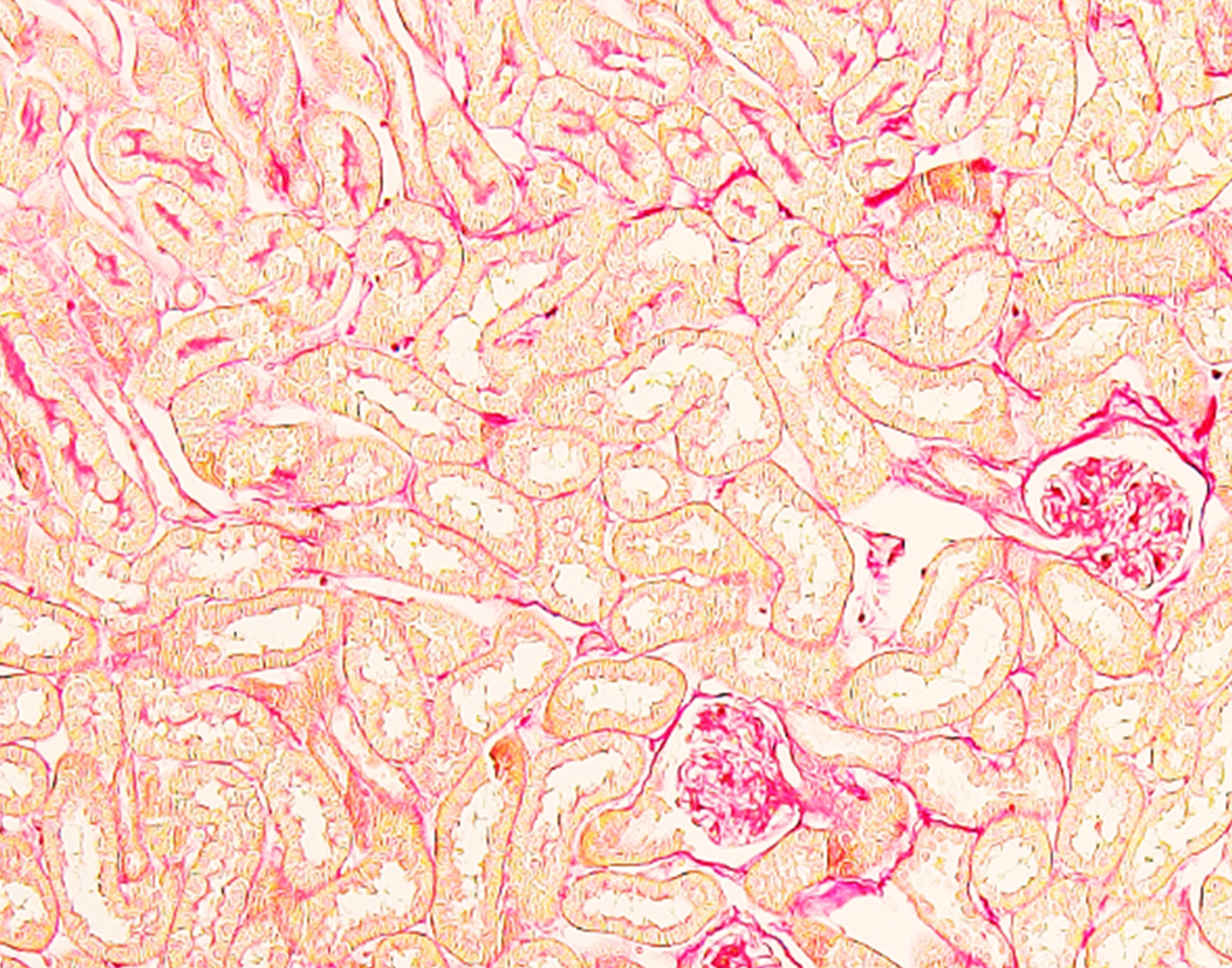

Supplement: Supplementary file 3 — Source data Fig. 1 [file 44321_2025_243_MOESM3_ESM.zip › 1H/Sirius red Sham WT.tiff]

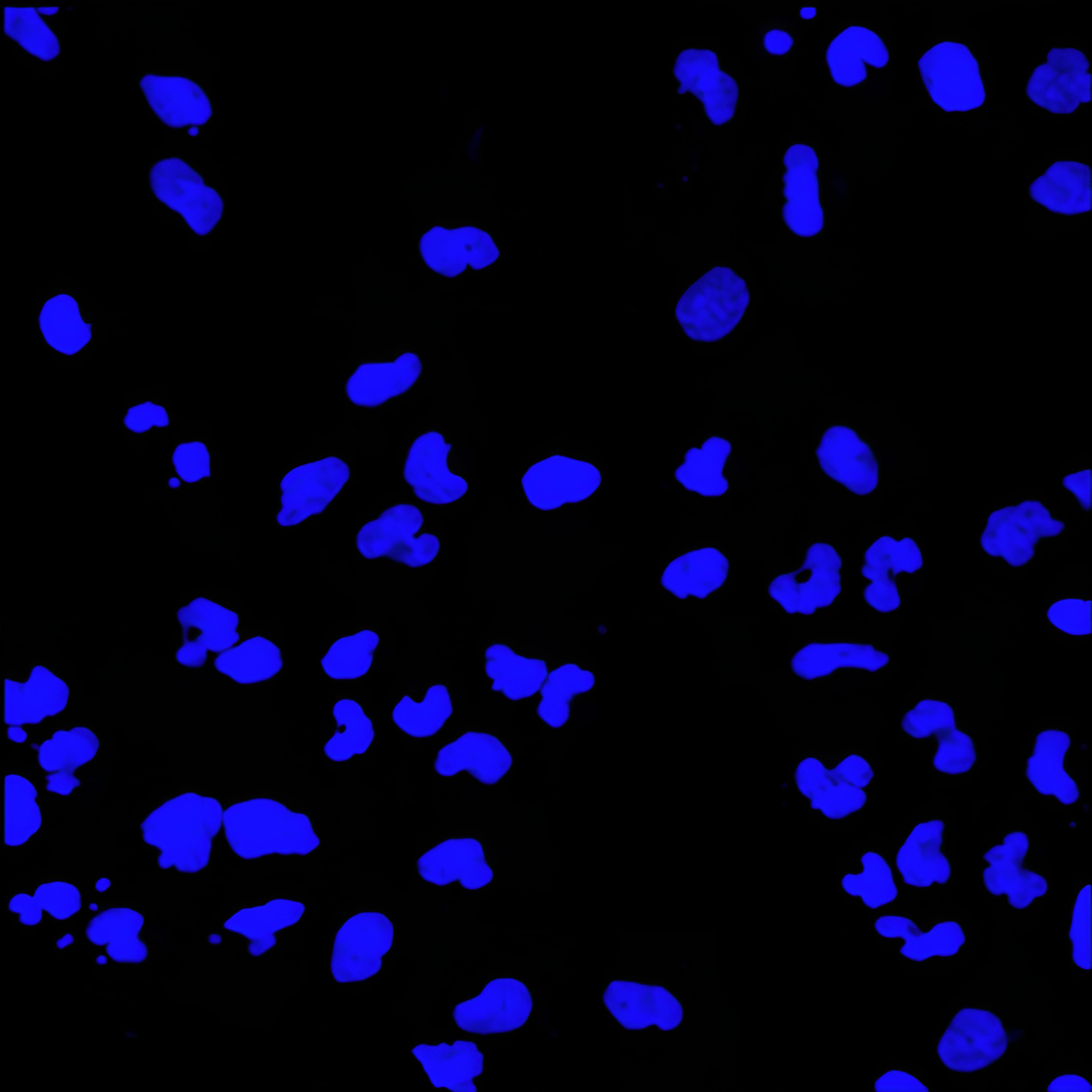

Supplement: Supplementary file 4 — Source data Fig. 2 [file 44321_2025_243_MOESM4_ESM.zip › 2A/Control_DAPI.tif]

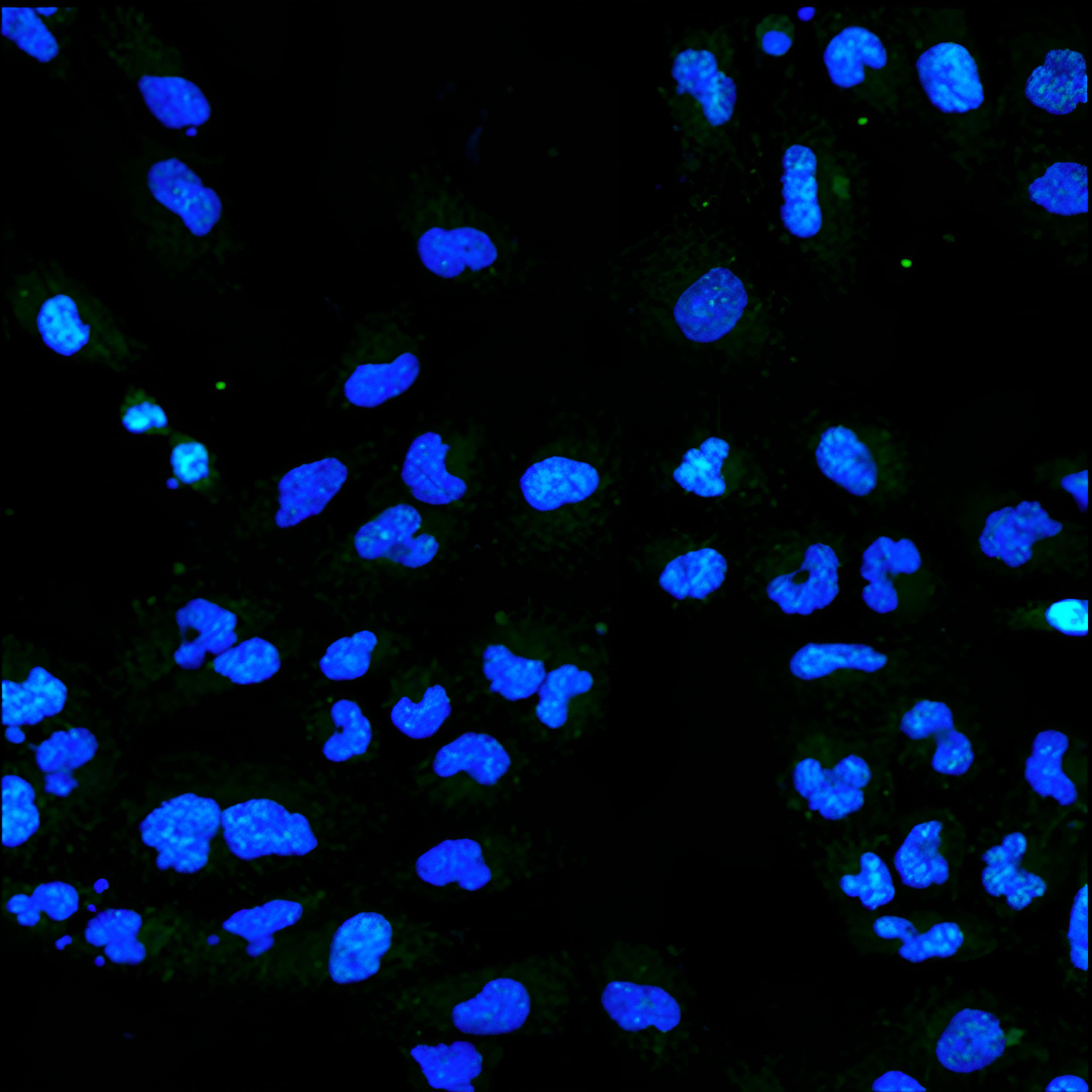

Supplement: Supplementary file 4 — Source data Fig. 2 [file 44321_2025_243_MOESM4_ESM.zip › 2A/Control_merge.tif]

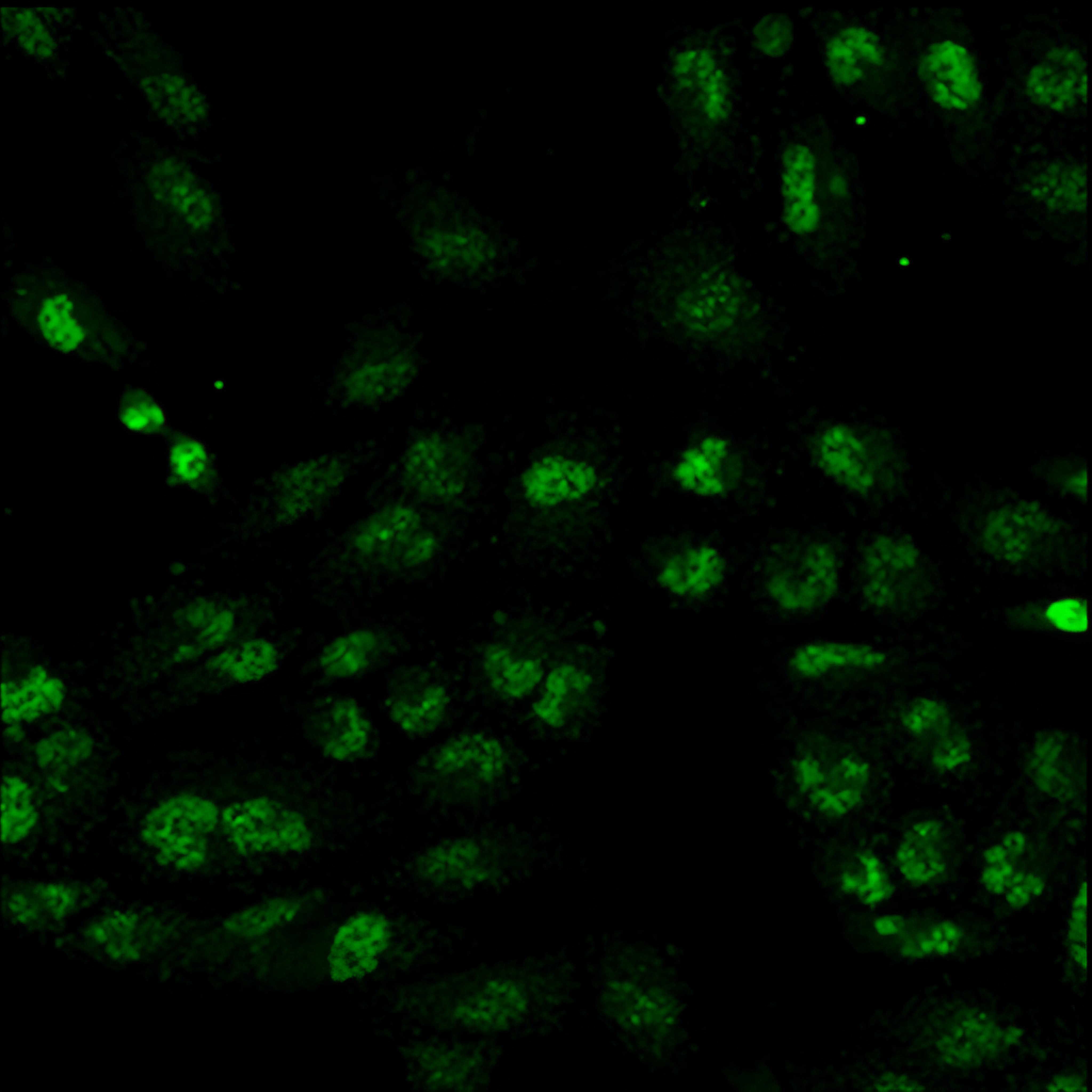

Supplement: Supplementary file 4 — Source data Fig. 2 [file 44321_2025_243_MOESM4_ESM.zip › 2A/Control_p300.tif]

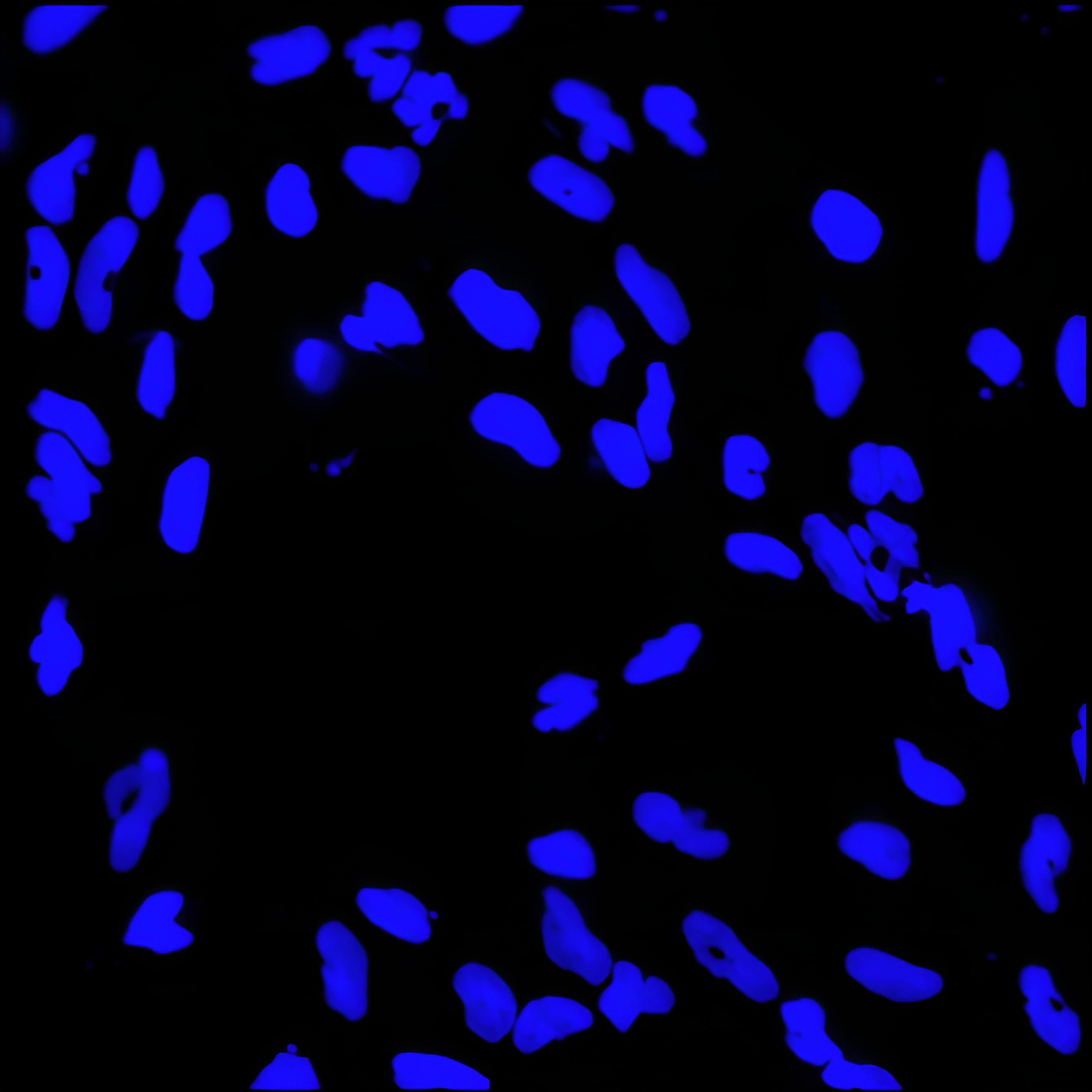

Supplement: Supplementary file 4 — Source data Fig. 2 [file 44321_2025_243_MOESM4_ESM.zip › 2A/TGF-beta_DAPI.tif]

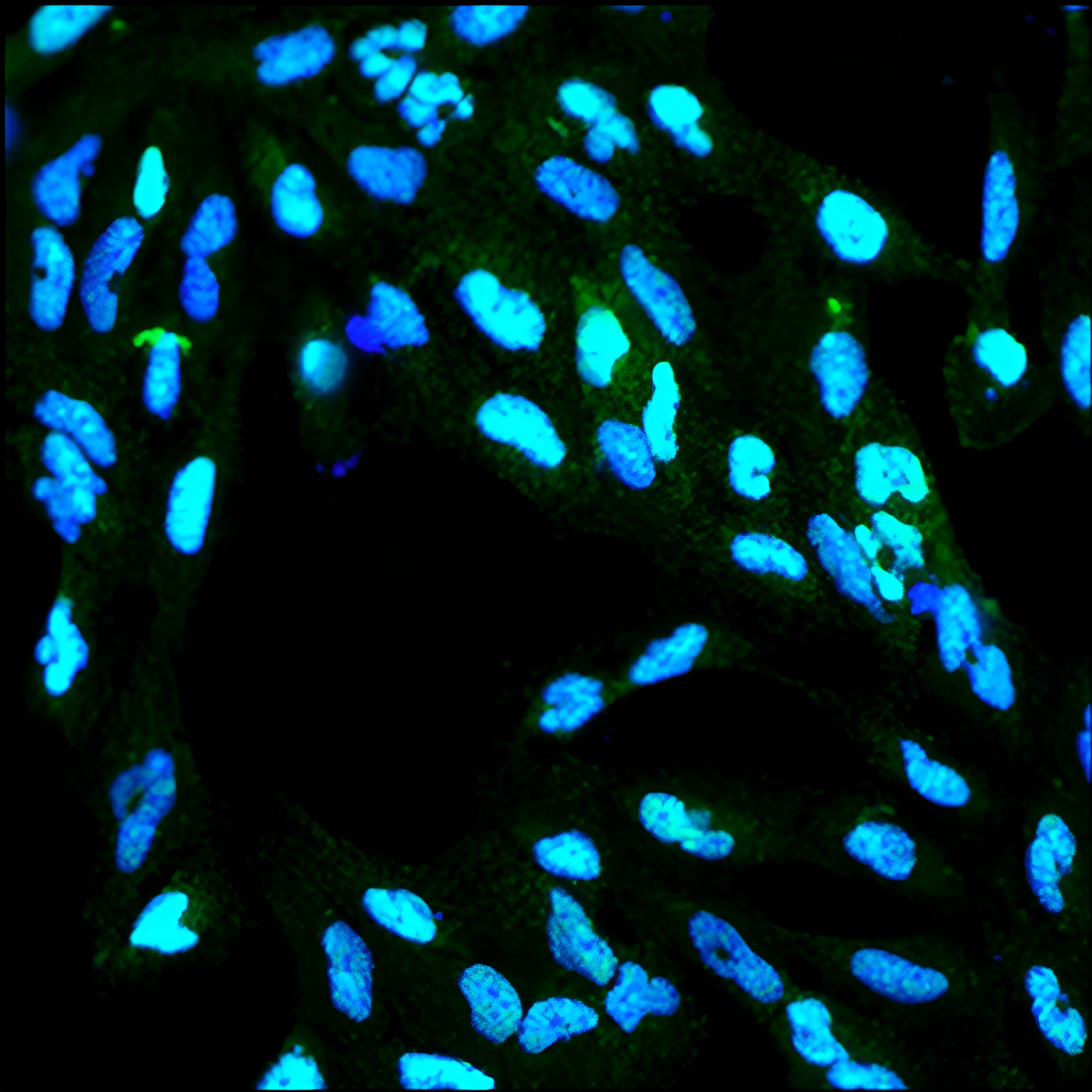

Supplement: Supplementary file 4 — Source data Fig. 2 [file 44321_2025_243_MOESM4_ESM.zip › 2A/TGF-beta_Merge.tif]

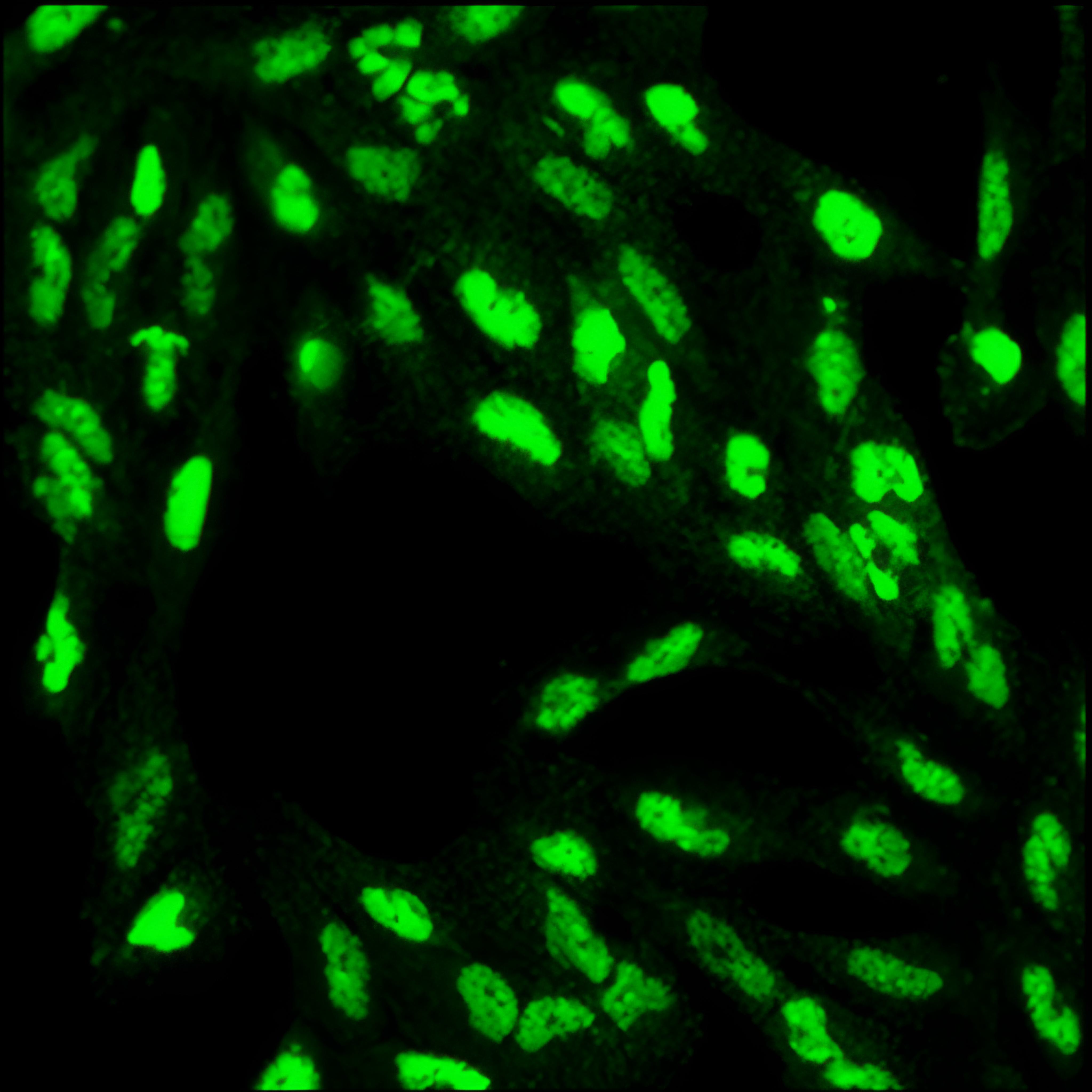

Supplement: Supplementary file 4 — Source data Fig. 2 [file 44321_2025_243_MOESM4_ESM.zip › 2A/TGF-beta_p300.tif]

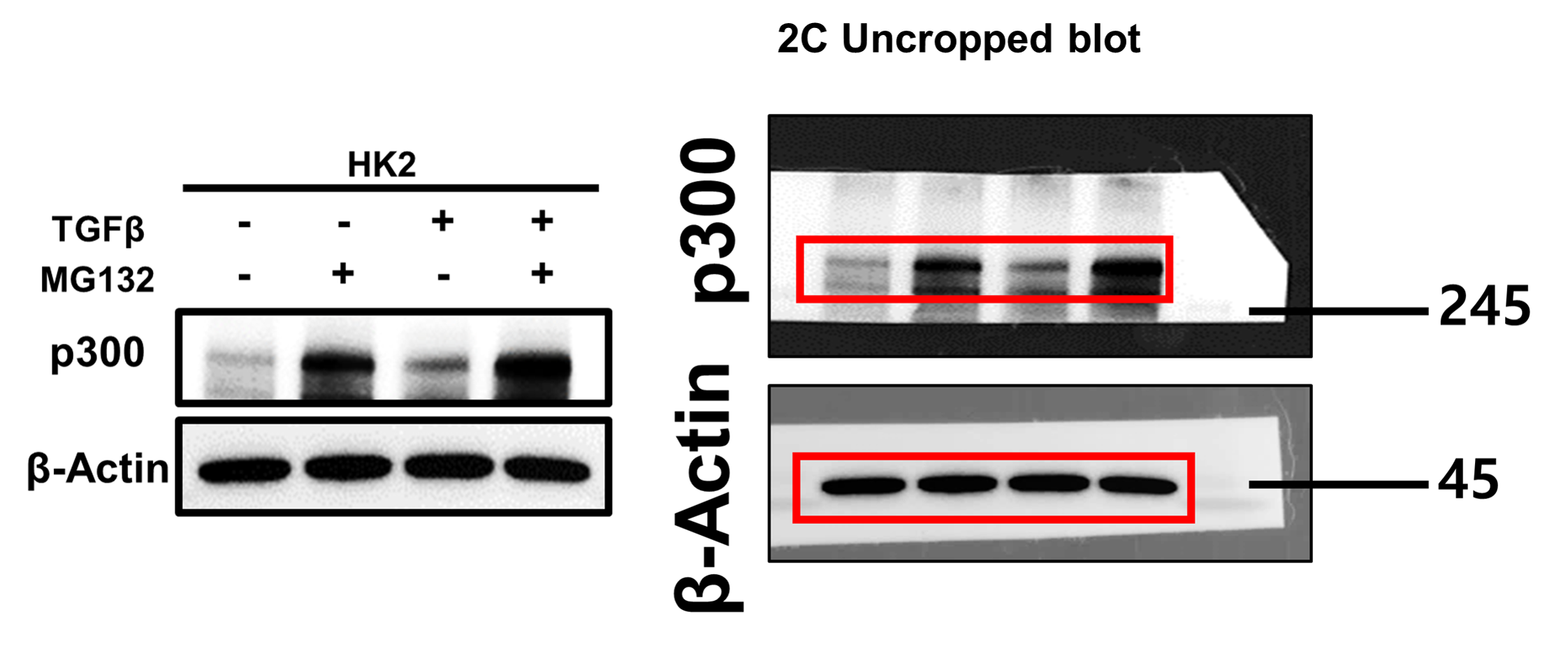

Supplement: Supplementary file 4 — Source data Fig. 2 [file 44321_2025_243_MOESM4_ESM.zip › 2C/2C_Uncropped blot.tif]

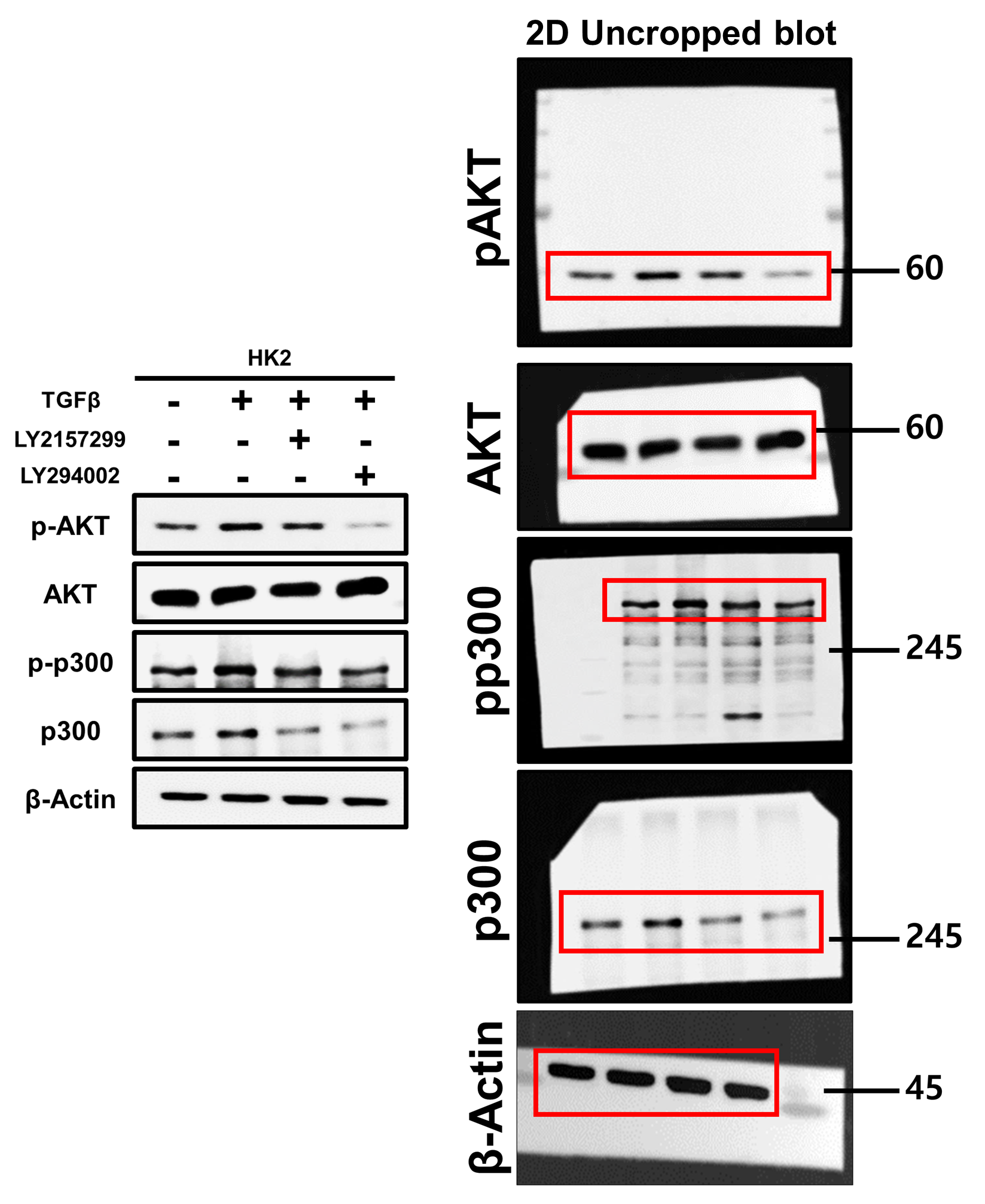

Supplement: Supplementary file 4 — Source data Fig. 2 [file 44321_2025_243_MOESM4_ESM.zip › 2D/2D_Uncropped blot.tif]

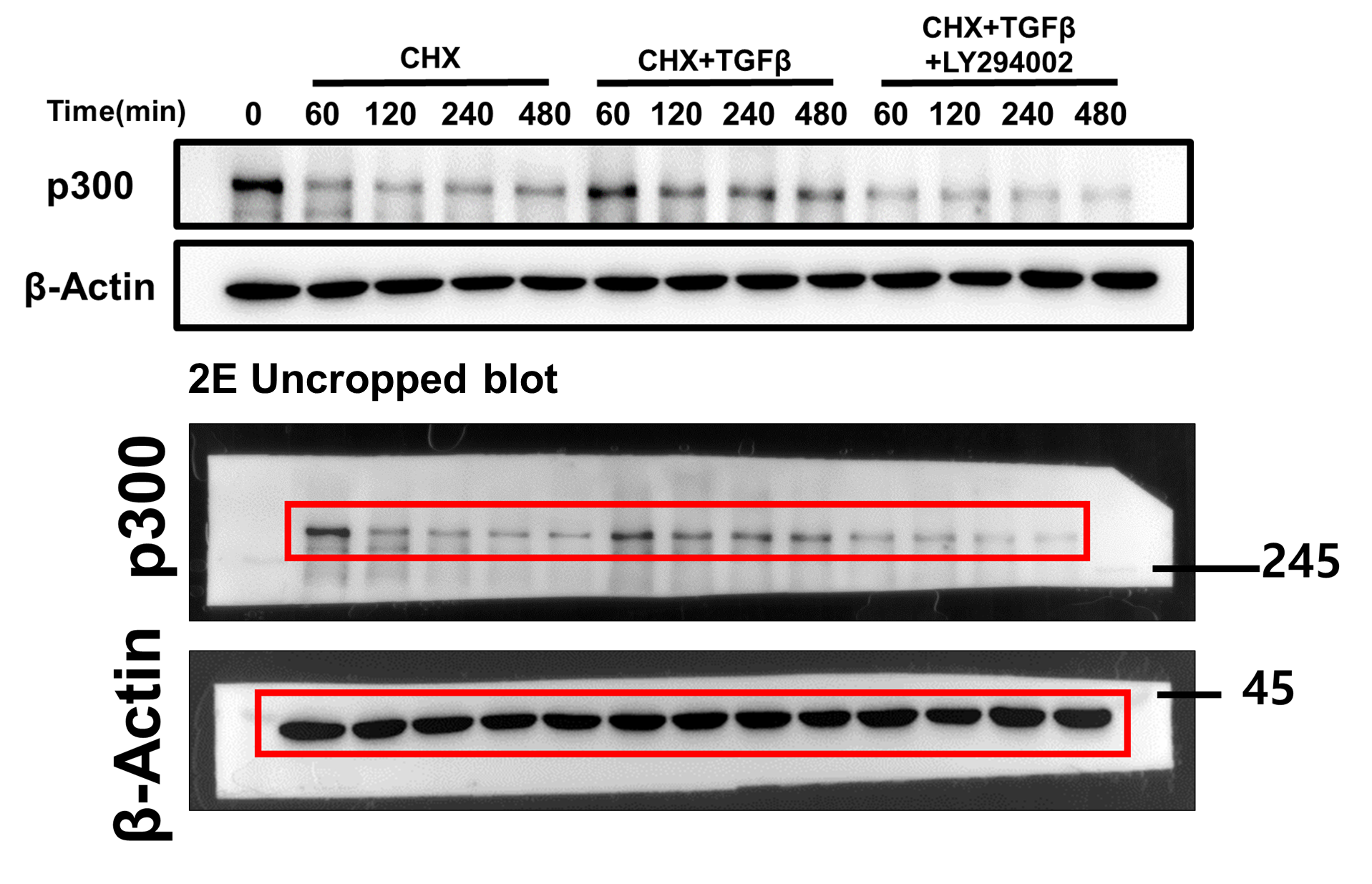

Supplement: Supplementary file 4 — Source data Fig. 2 [file 44321_2025_243_MOESM4_ESM.zip › 2E/2E_Uncropped blot.tif]

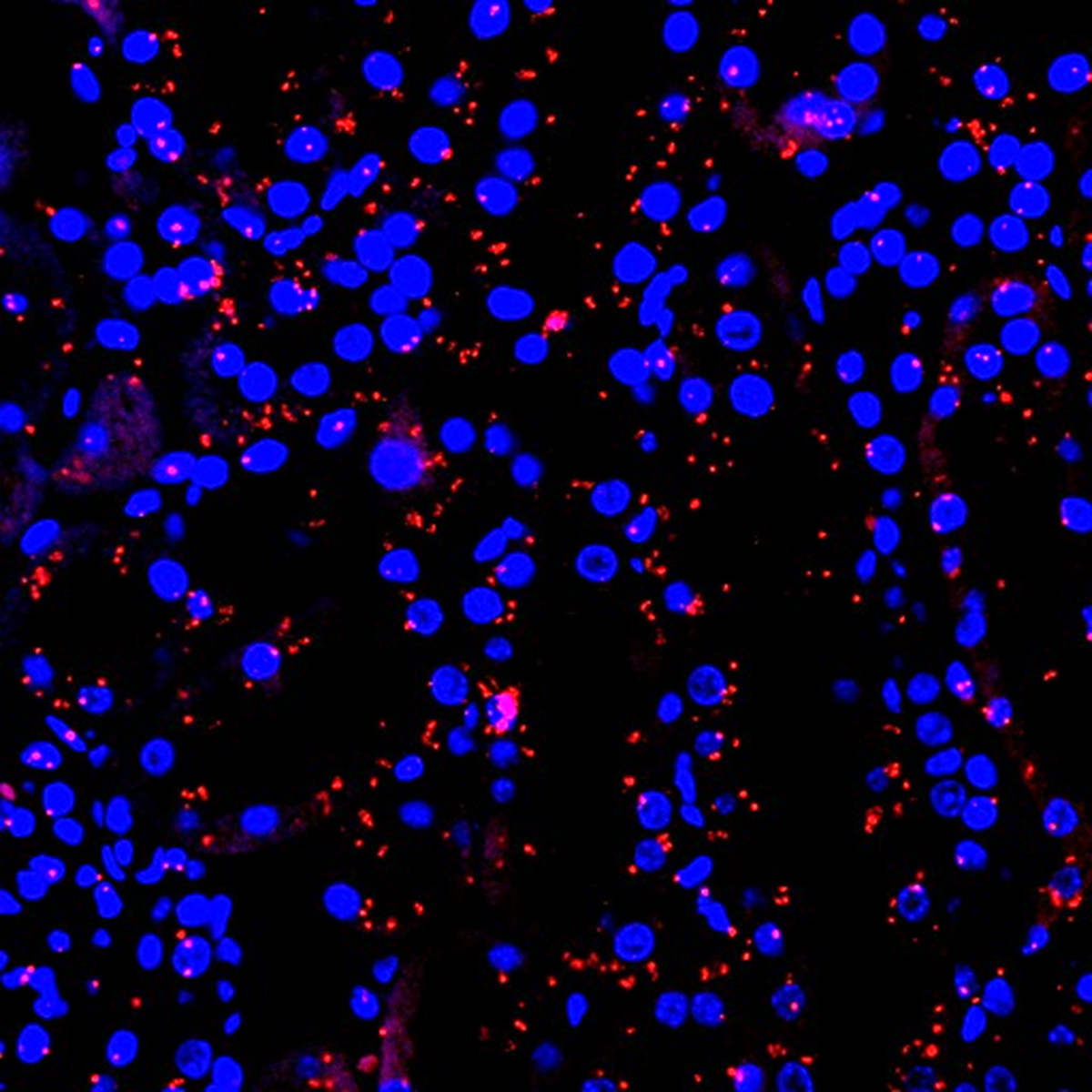

Supplement: Supplementary file 4 — Source data Fig. 2 [file 44321_2025_243_MOESM4_ESM.zip › 2I/Sham PLA-DAPI.tif]

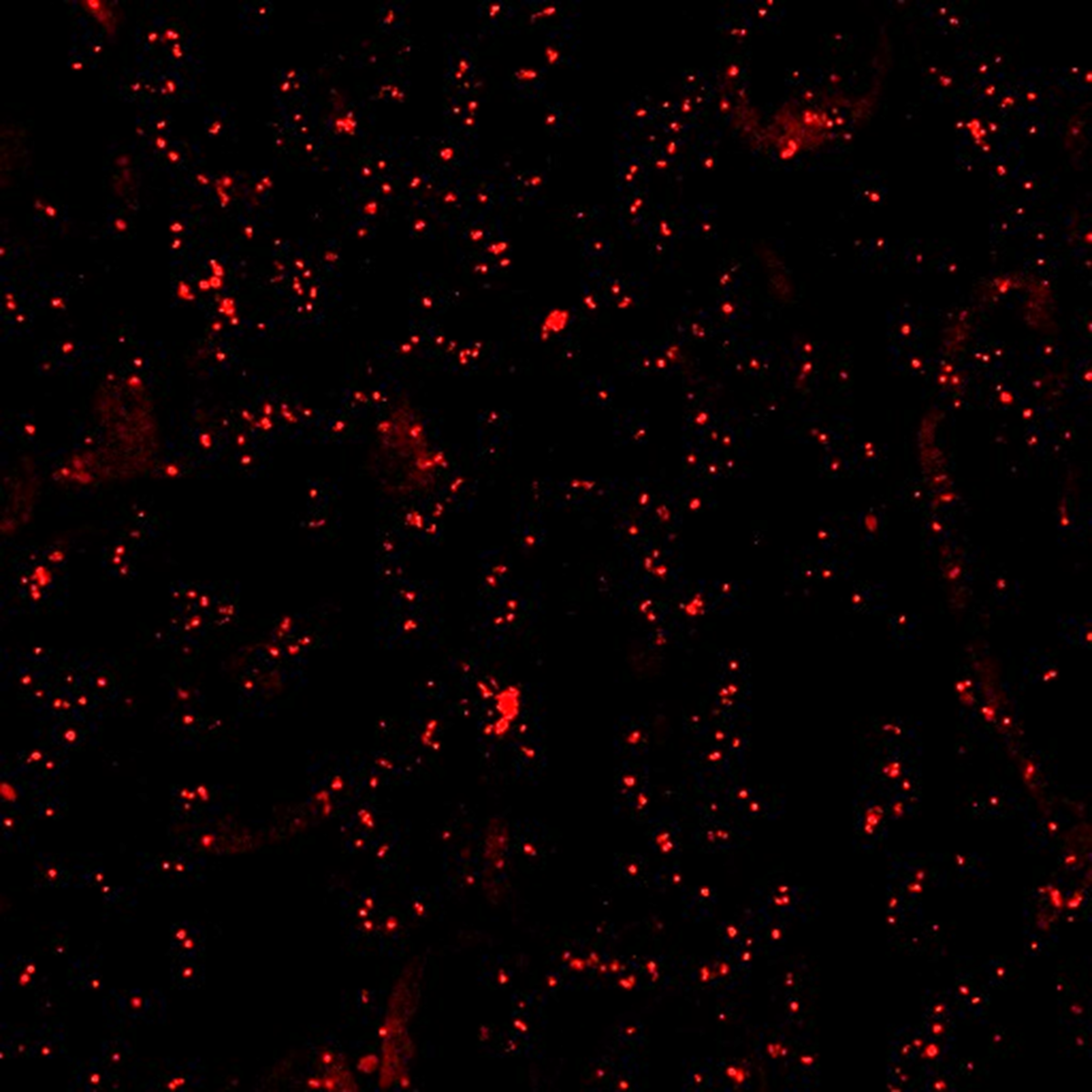

Supplement: Supplementary file 4 — Source data Fig. 2 [file 44321_2025_243_MOESM4_ESM.zip › 2I/Sham PLA.tif]

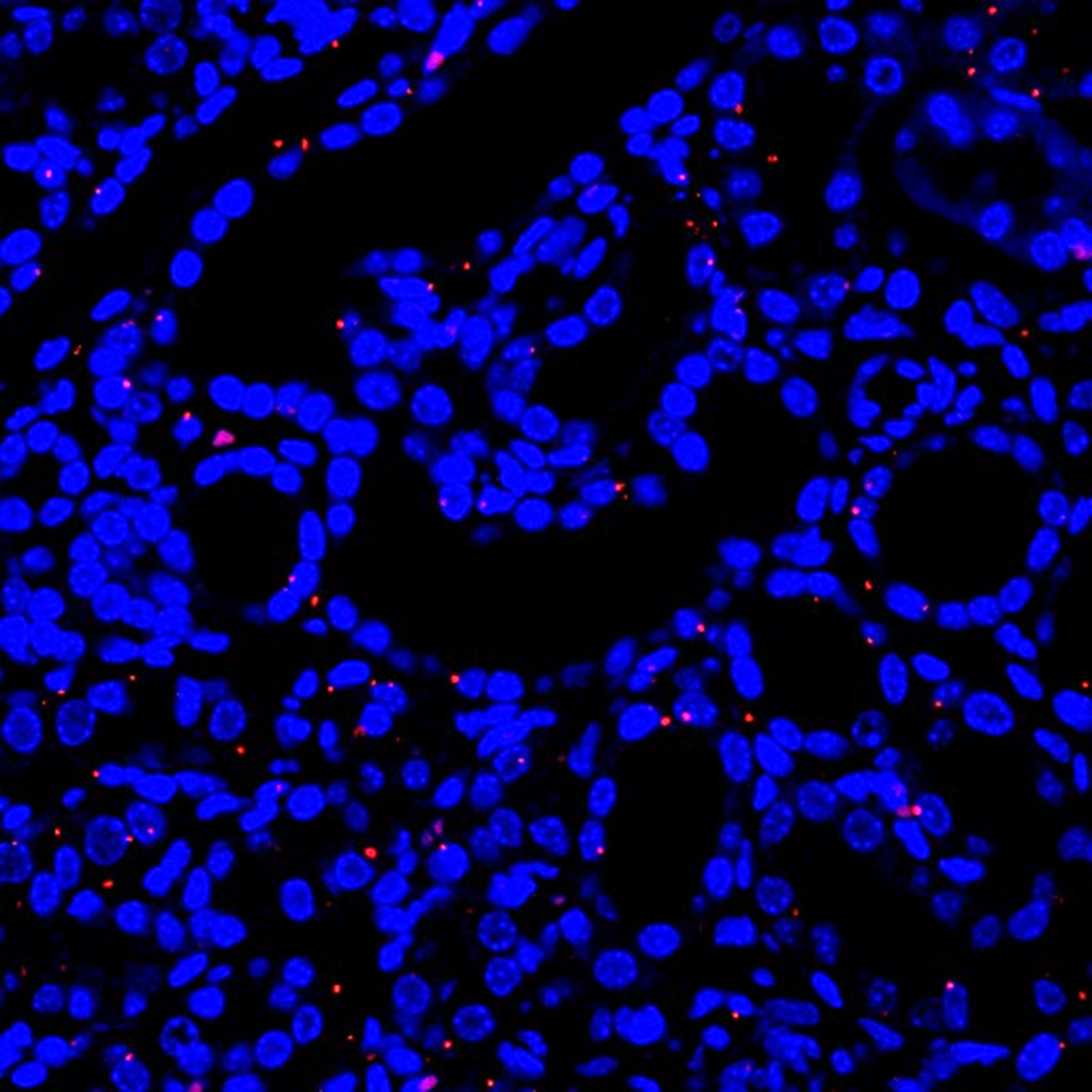

Supplement: Supplementary file 4 — Source data Fig. 2 [file 44321_2025_243_MOESM4_ESM.zip › 2I/UUO PLA-DAPI.tif]

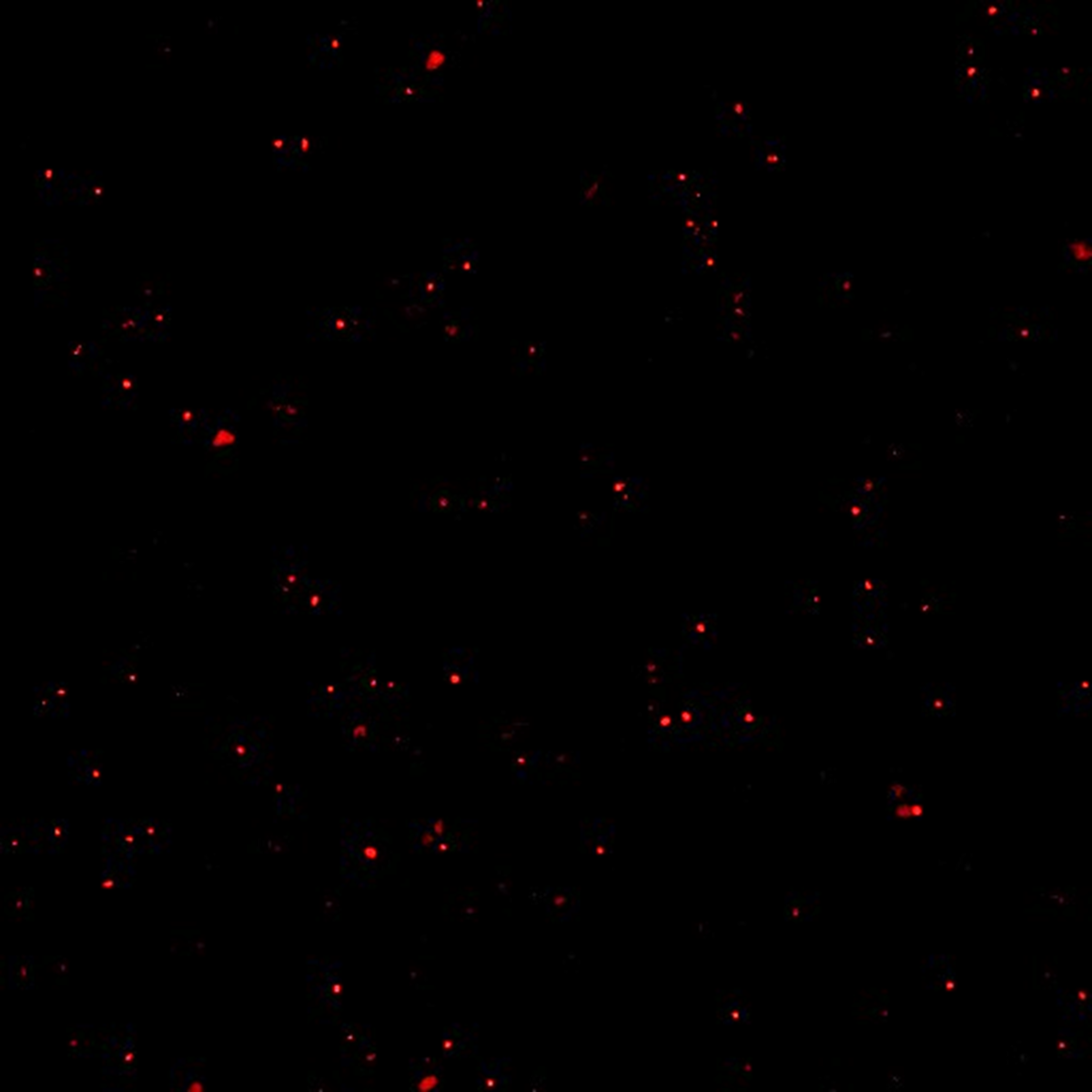

Supplement: Supplementary file 4 — Source data Fig. 2 [file 44321_2025_243_MOESM4_ESM.zip › 2I/UUO PLA.tif]

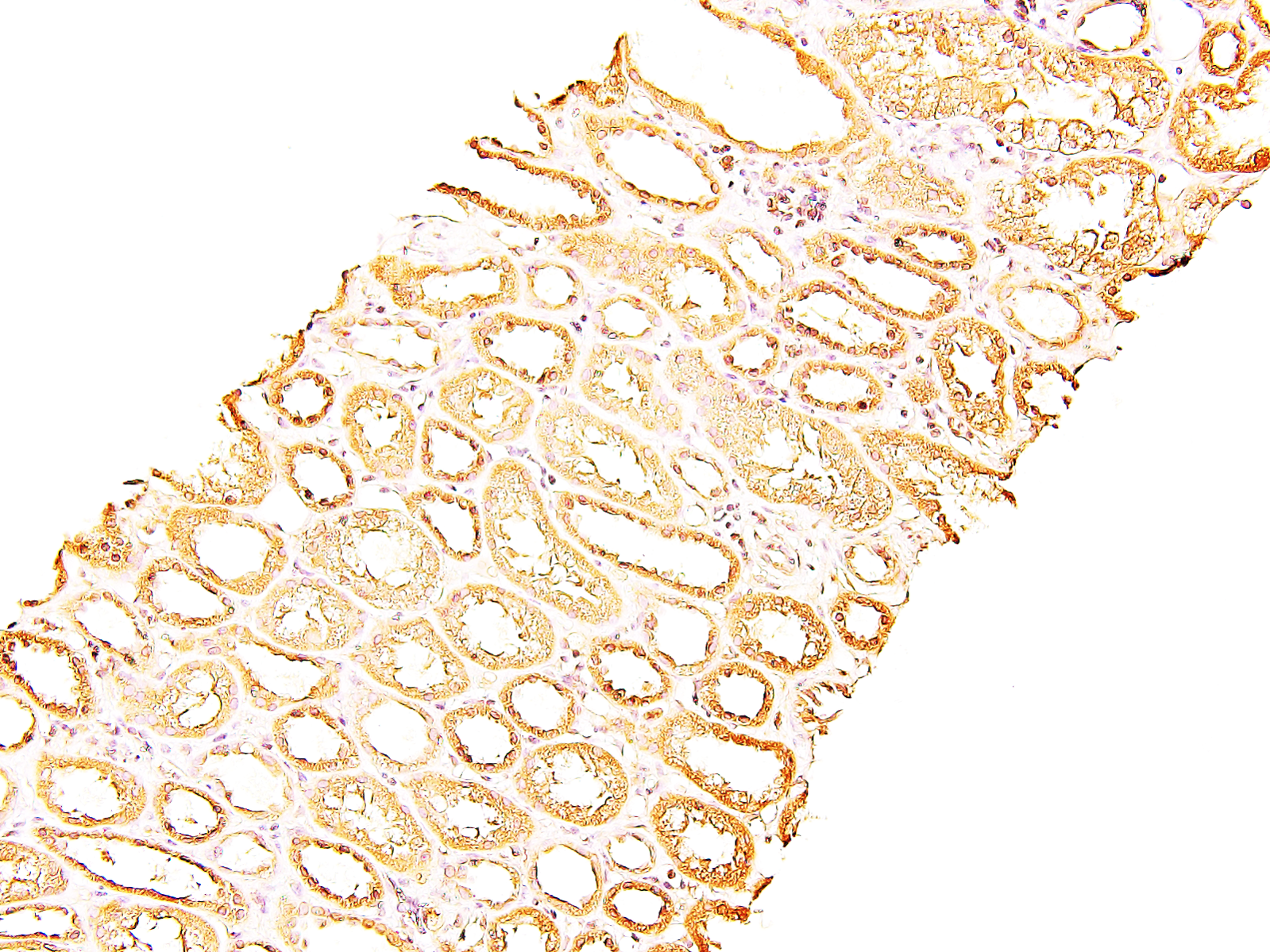

Supplement: Supplementary file 5 — Source data Fig. 3 [file 44321_2025_243_MOESM5_ESM.zip › 3A/PPM1K FSGS(200x).tif]

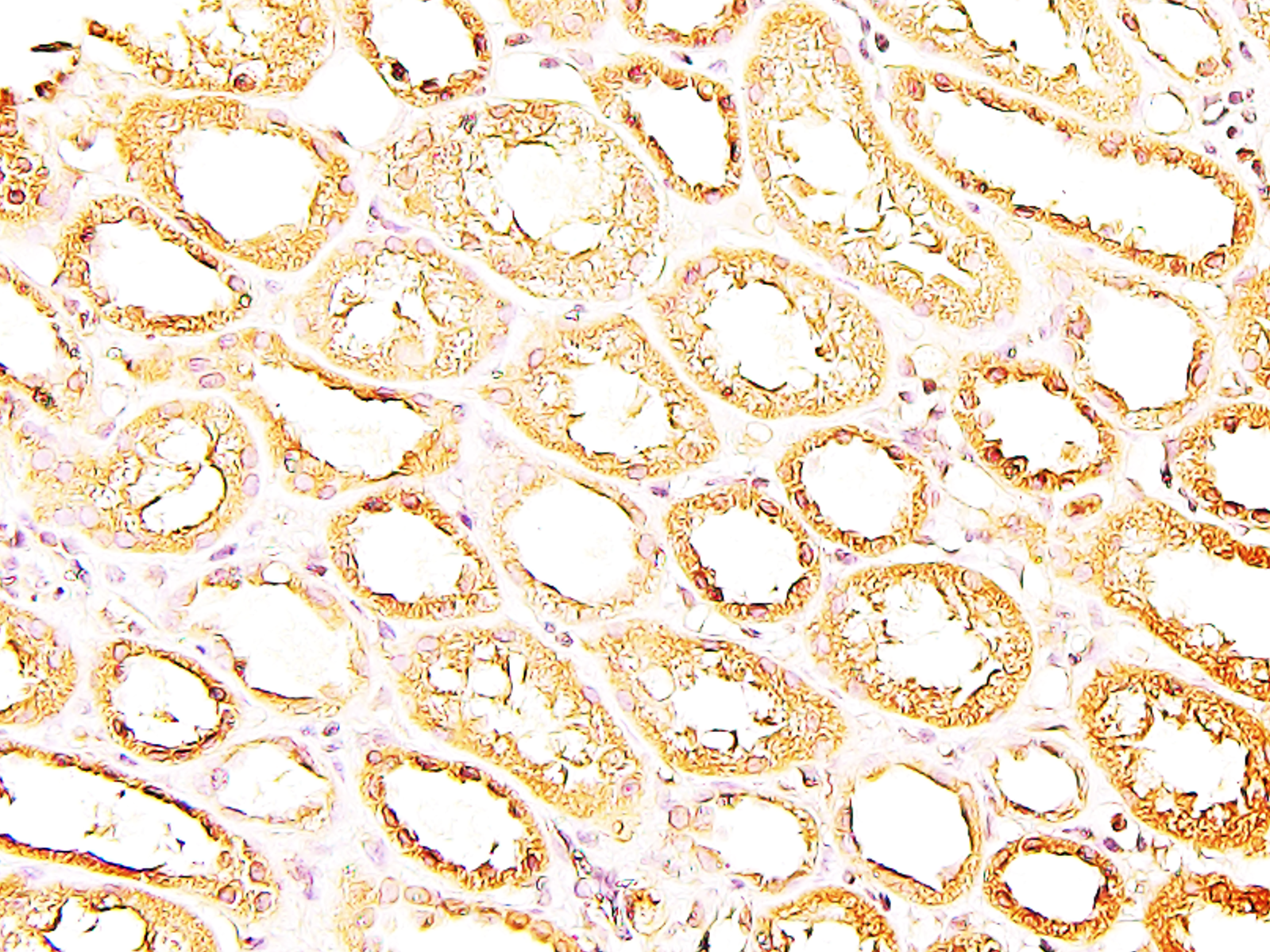

Supplement: Supplementary file 5 — Source data Fig. 3 [file 44321_2025_243_MOESM5_ESM.zip › 3A/PPM1K FSGS(400x).tif]

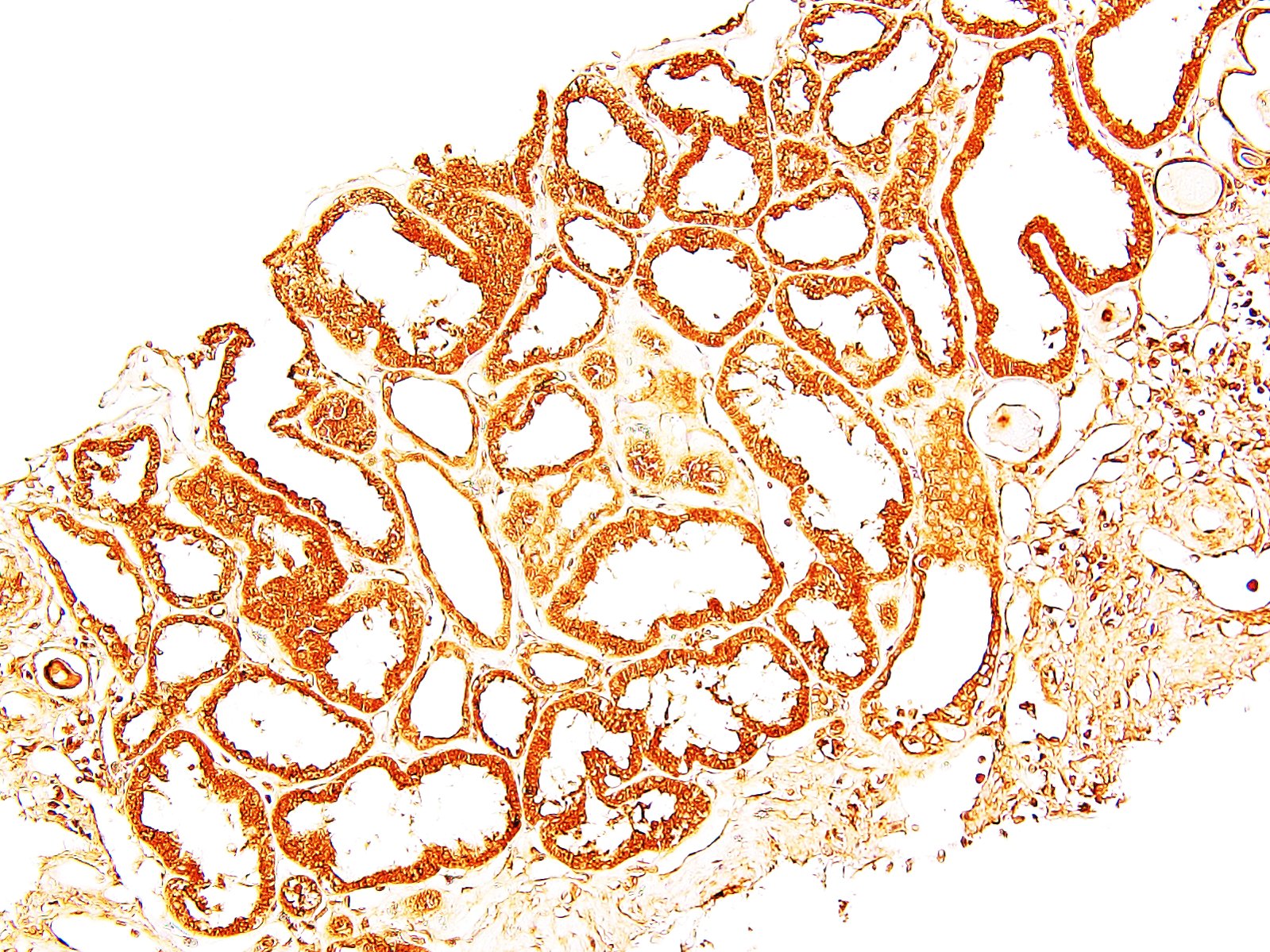

Supplement: Supplementary file 5 — Source data Fig. 3 [file 44321_2025_243_MOESM5_ESM.zip › 3A/PPM1K MCD(200x).tif]

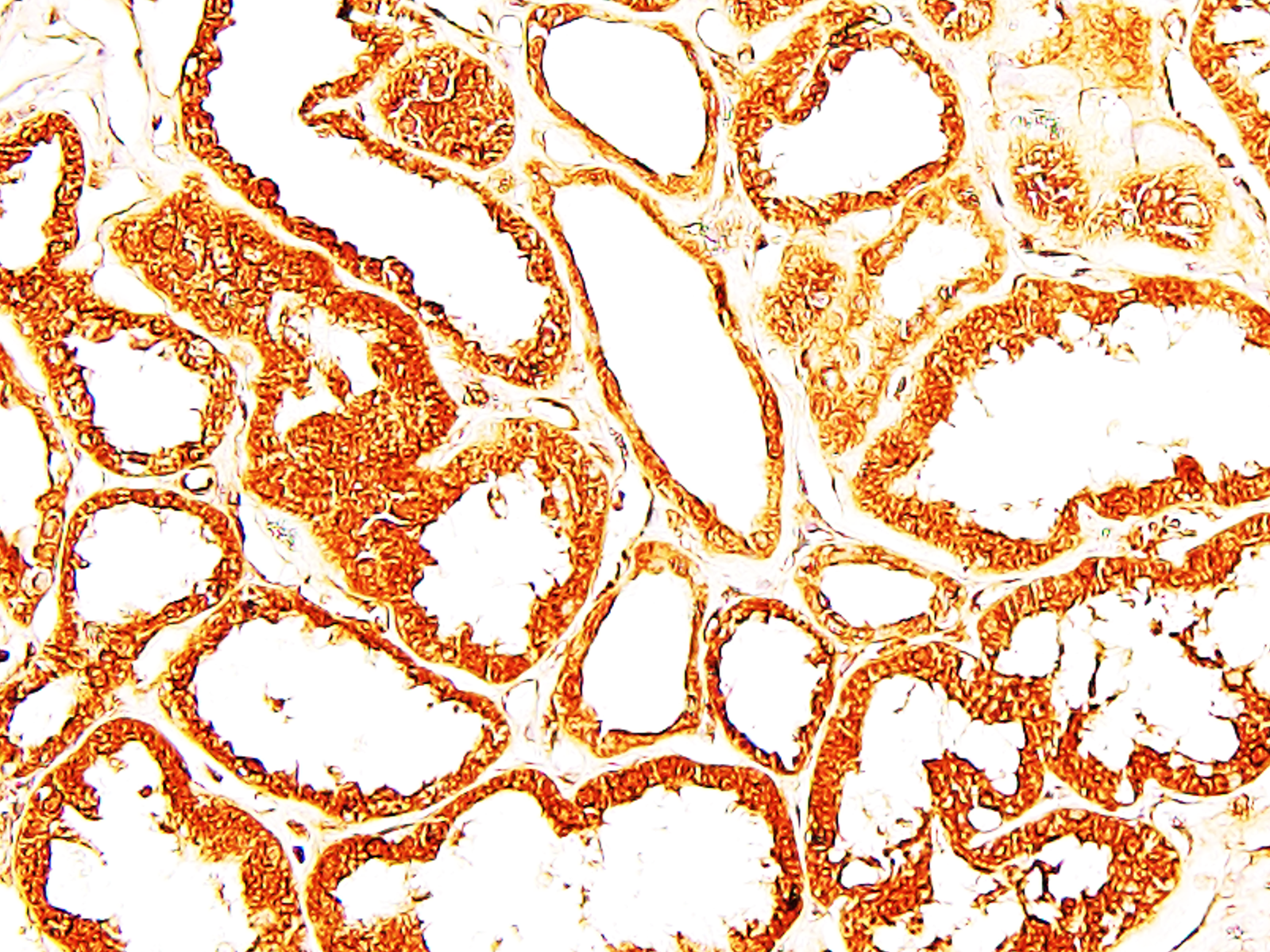

Supplement: Supplementary file 5 — Source data Fig. 3 [file 44321_2025_243_MOESM5_ESM.zip › 3A/PPM1K MCD(400x).tif]

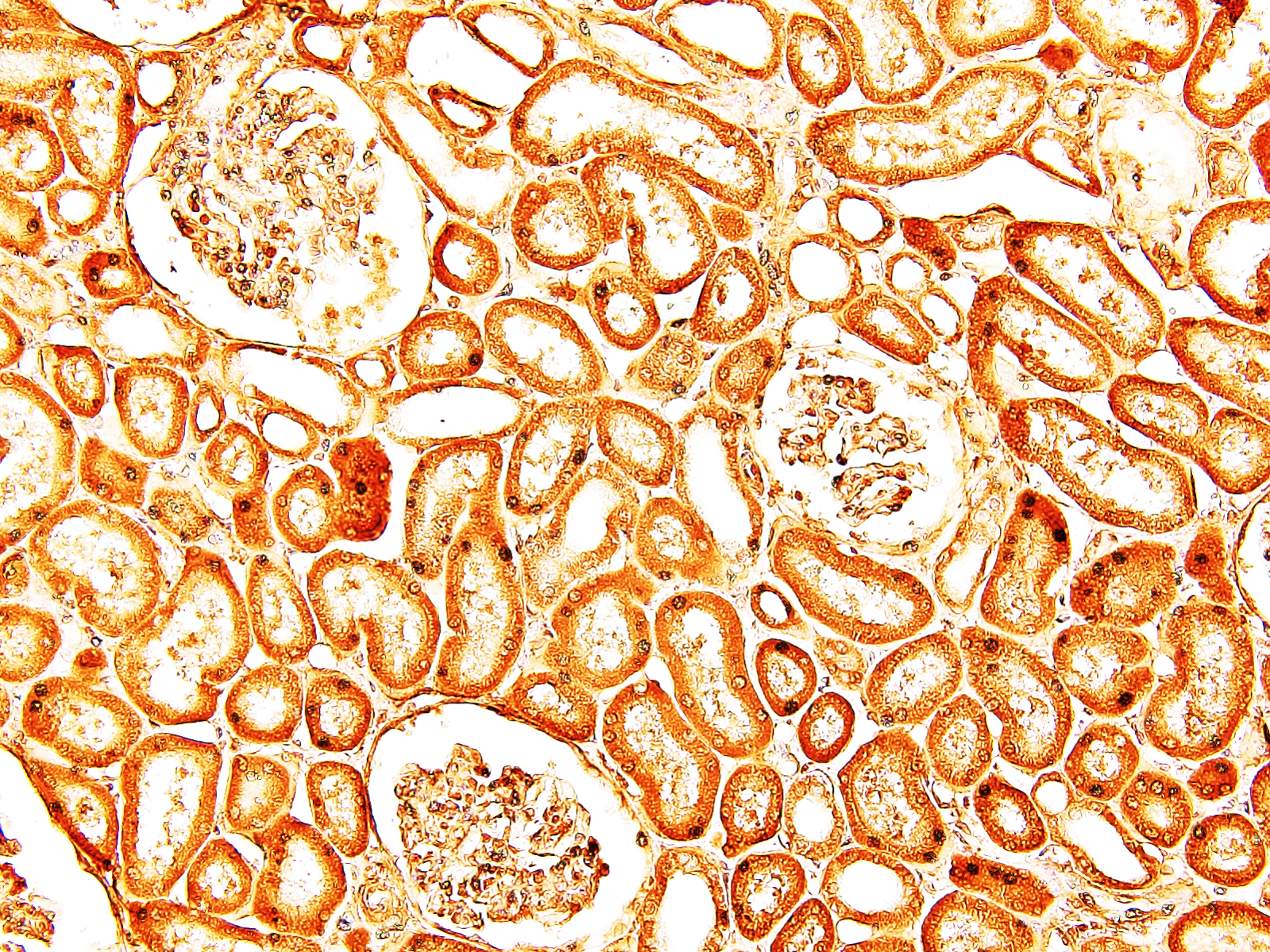

Supplement: Supplementary file 5 — Source data Fig. 3 [file 44321_2025_243_MOESM5_ESM.zip › 3A/PPM1K Normal(200x).tif]

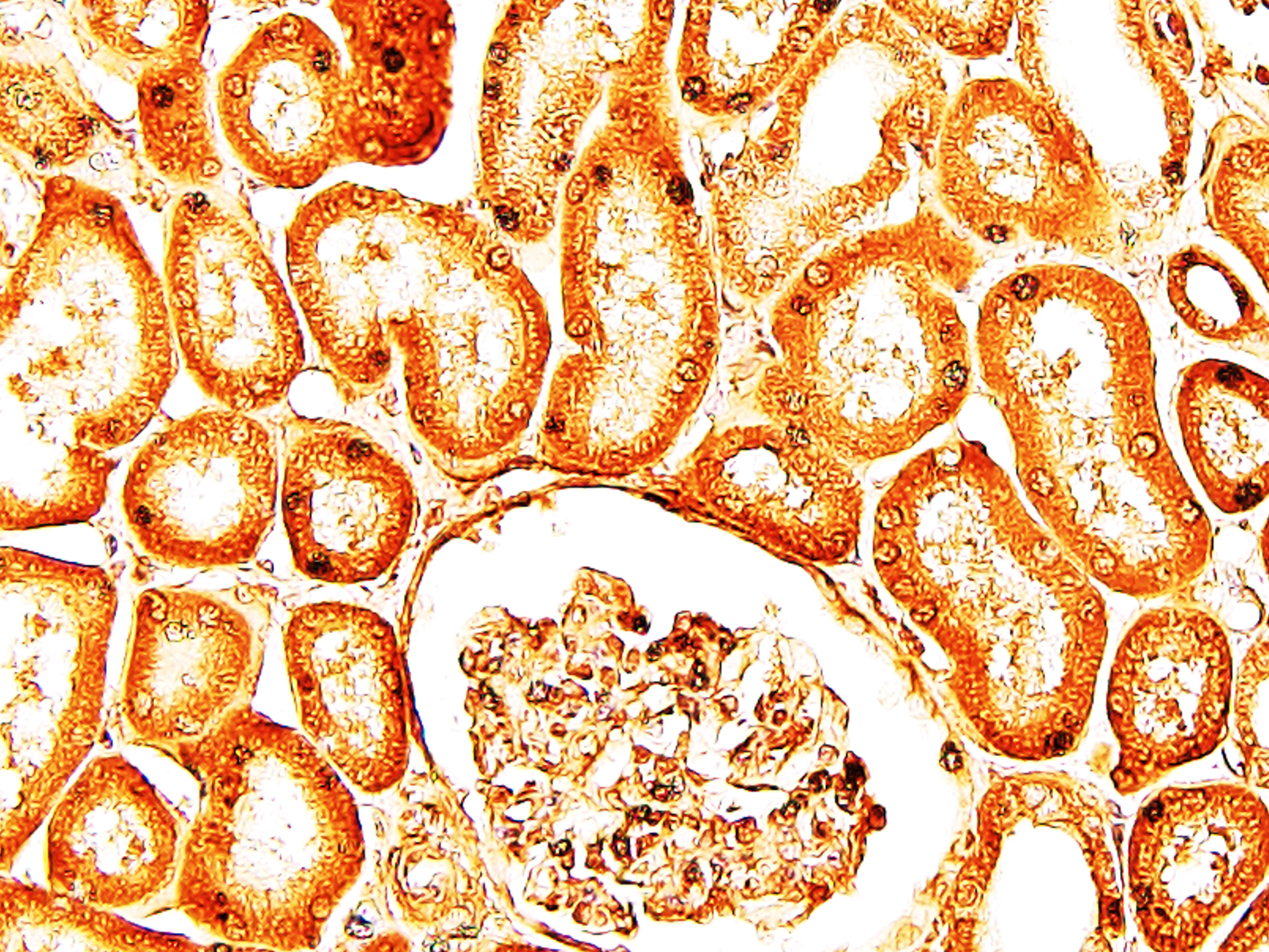

Supplement: Supplementary file 5 — Source data Fig. 3 [file 44321_2025_243_MOESM5_ESM.zip › 3A/PPM1K Normal(400x).tif]

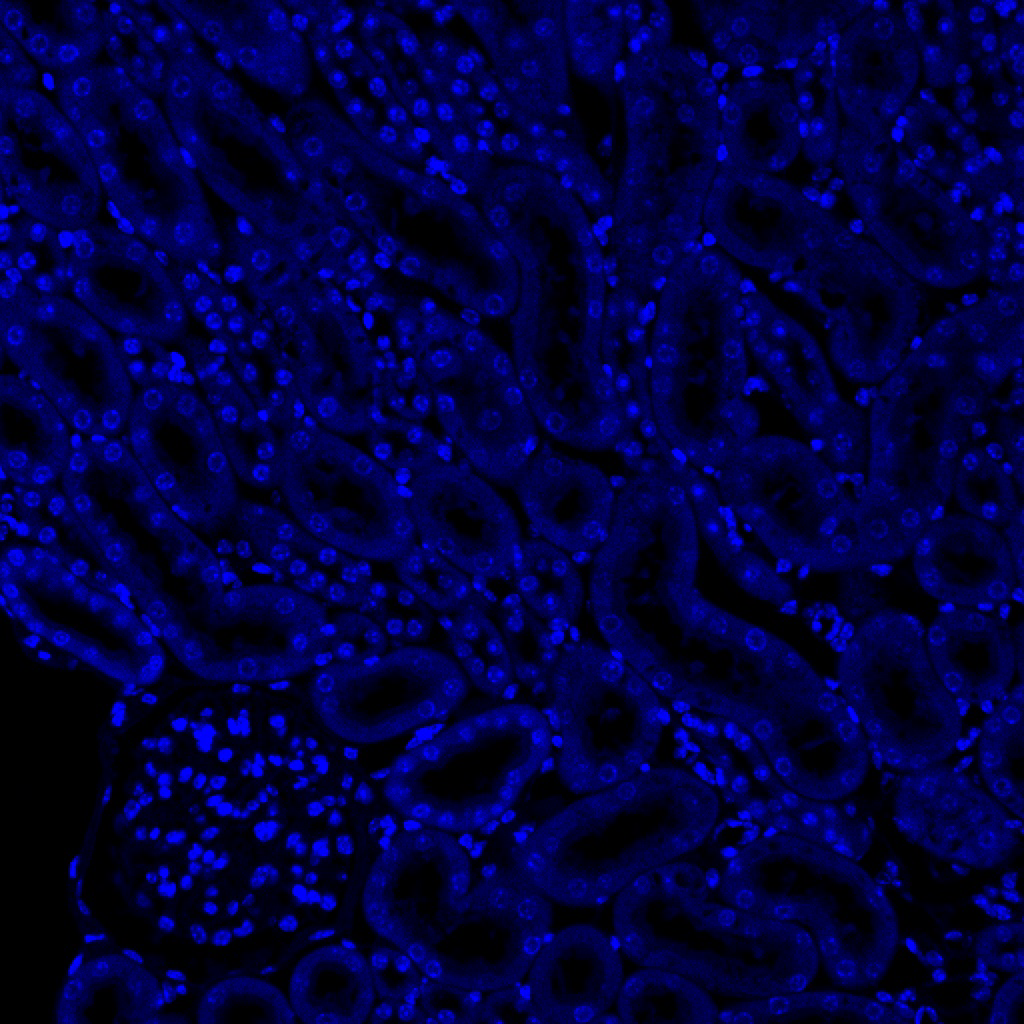

Supplement: Supplementary file 5 — Source data Fig. 3 [file 44321_2025_243_MOESM5_ESM.zip › 3C/Sham DAPI.tif]

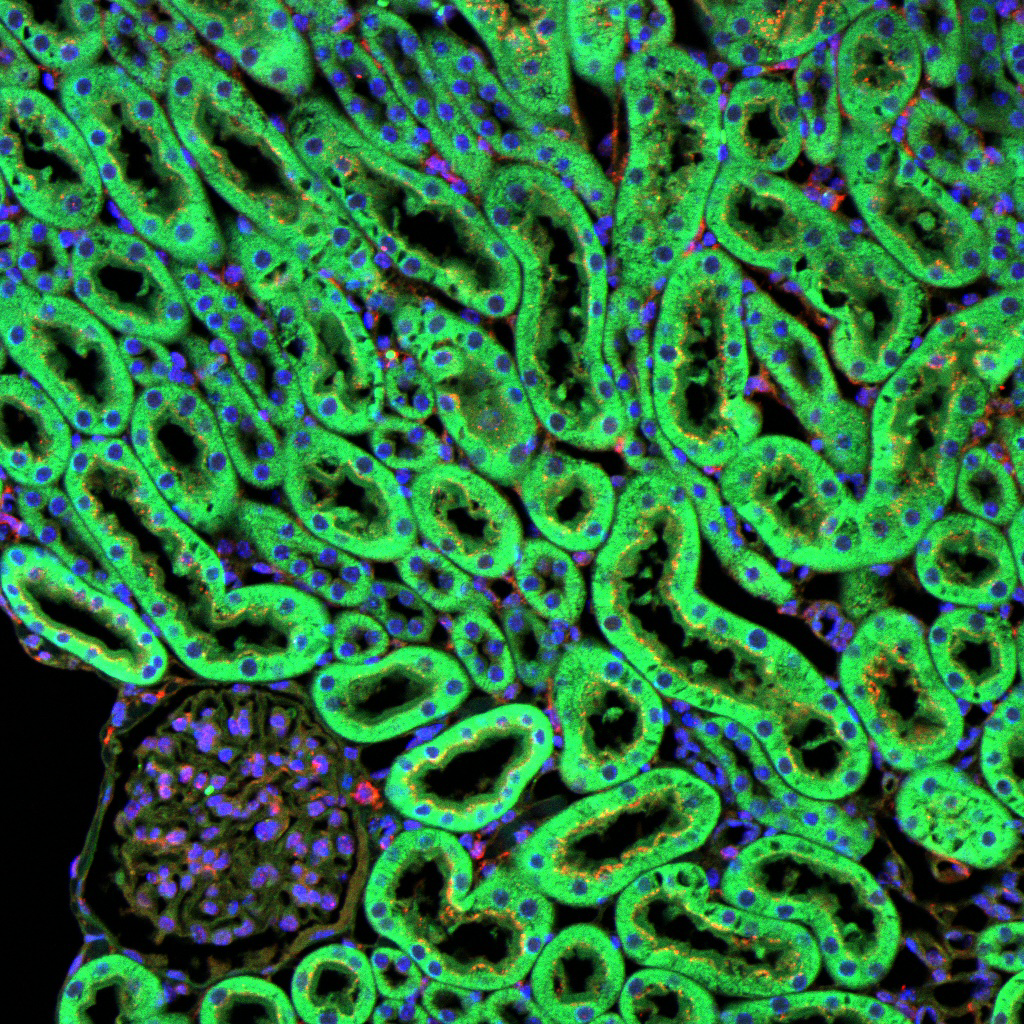

Supplement: Supplementary file 5 — Source data Fig. 3 [file 44321_2025_243_MOESM5_ESM.zip › 3C/Sham merge.tif]

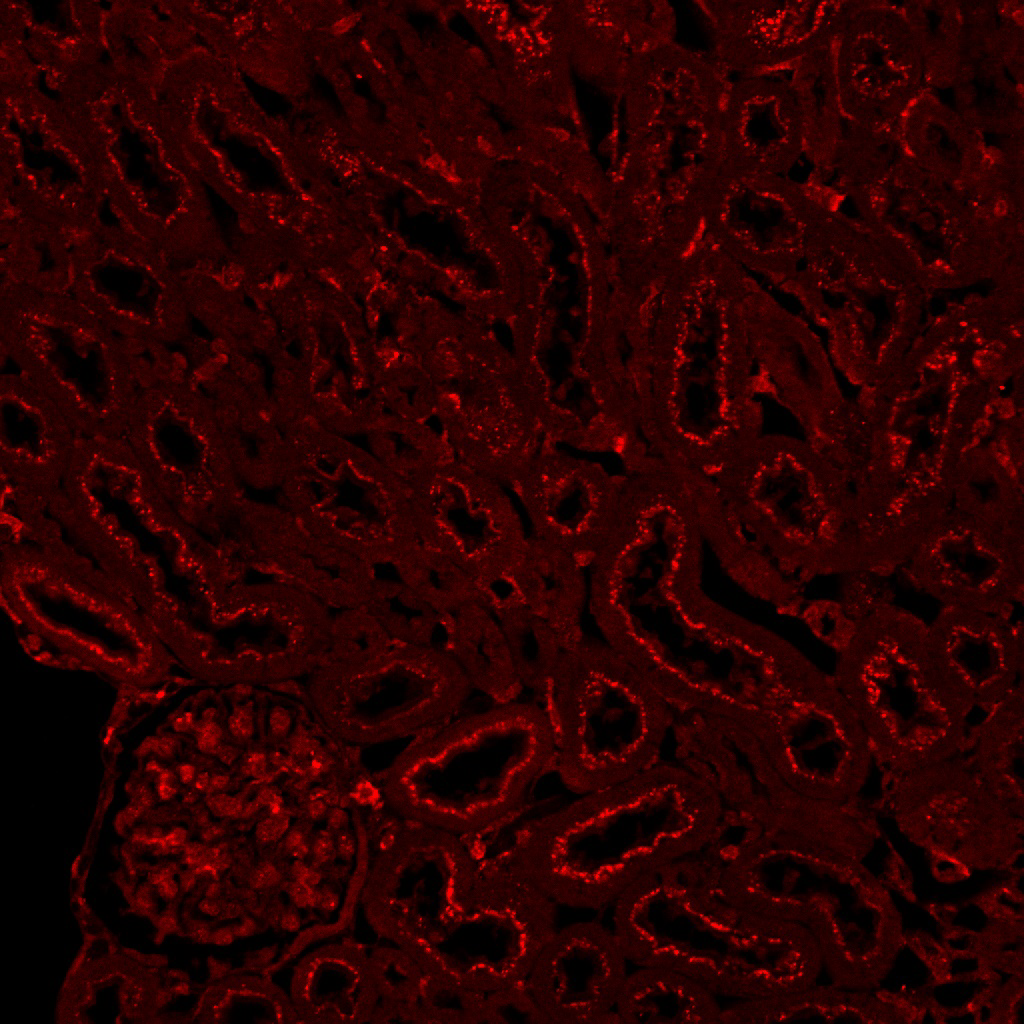

Supplement: Supplementary file 5 — Source data Fig. 3 [file 44321_2025_243_MOESM5_ESM.zip › 3C/Sham p300.tif]

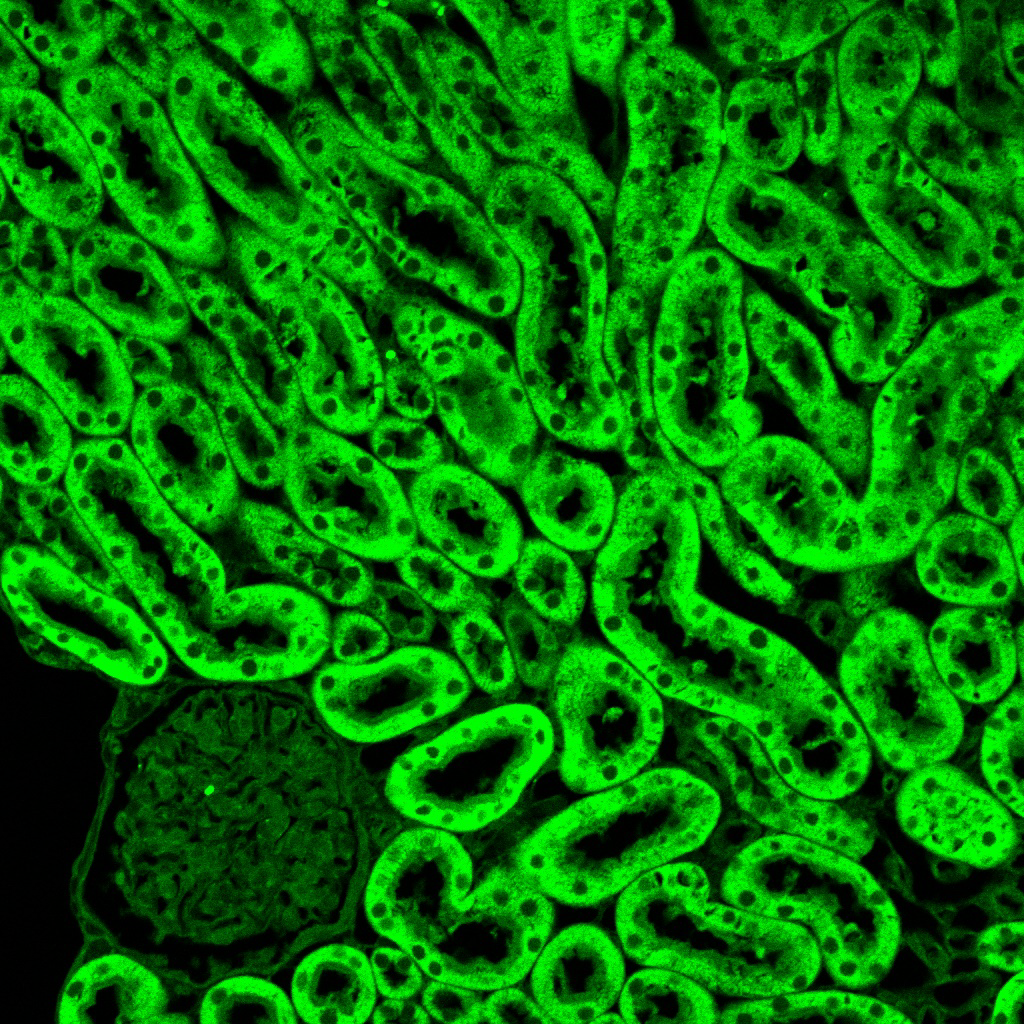

Supplement: Supplementary file 5 — Source data Fig. 3 [file 44321_2025_243_MOESM5_ESM.zip › 3C/Sham ppm1k.tif]

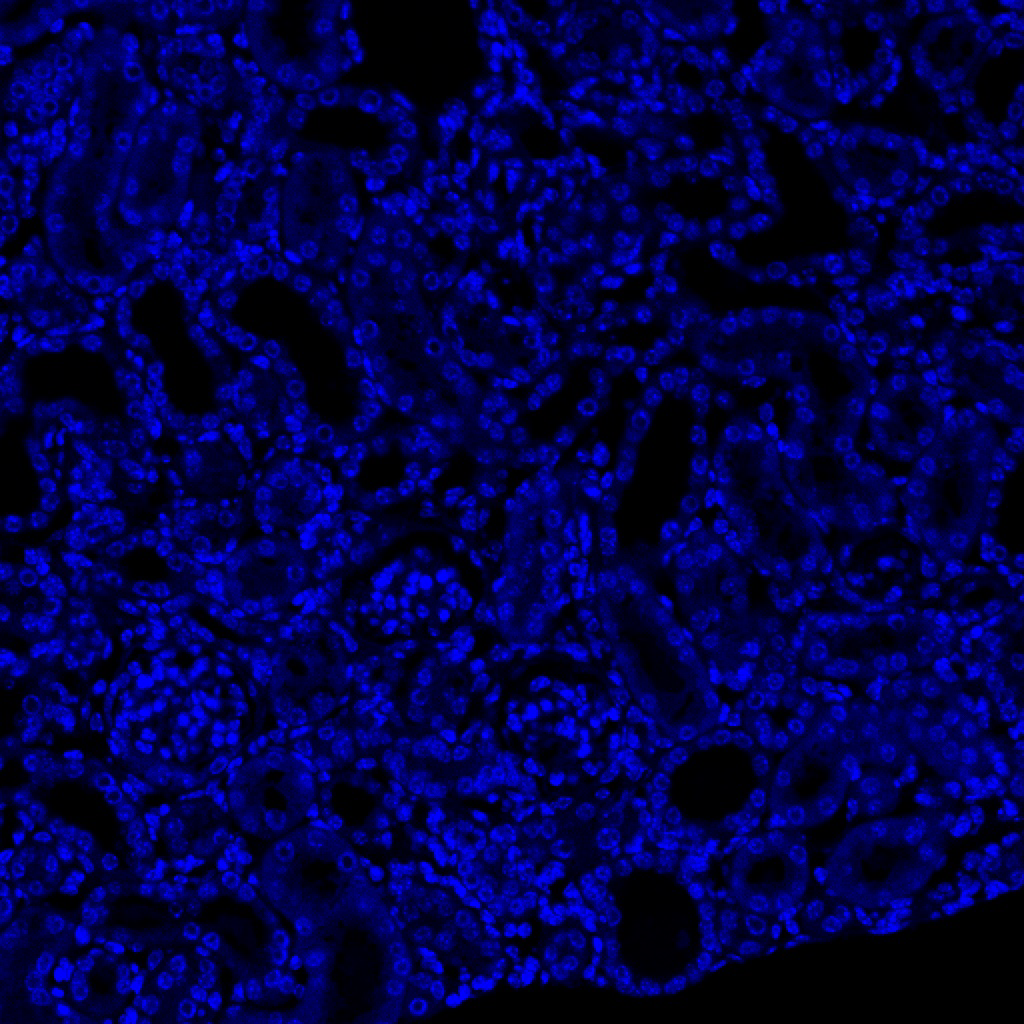

Supplement: Supplementary file 5 — Source data Fig. 3 [file 44321_2025_243_MOESM5_ESM.zip › 3C/UUO DAPI.tif]

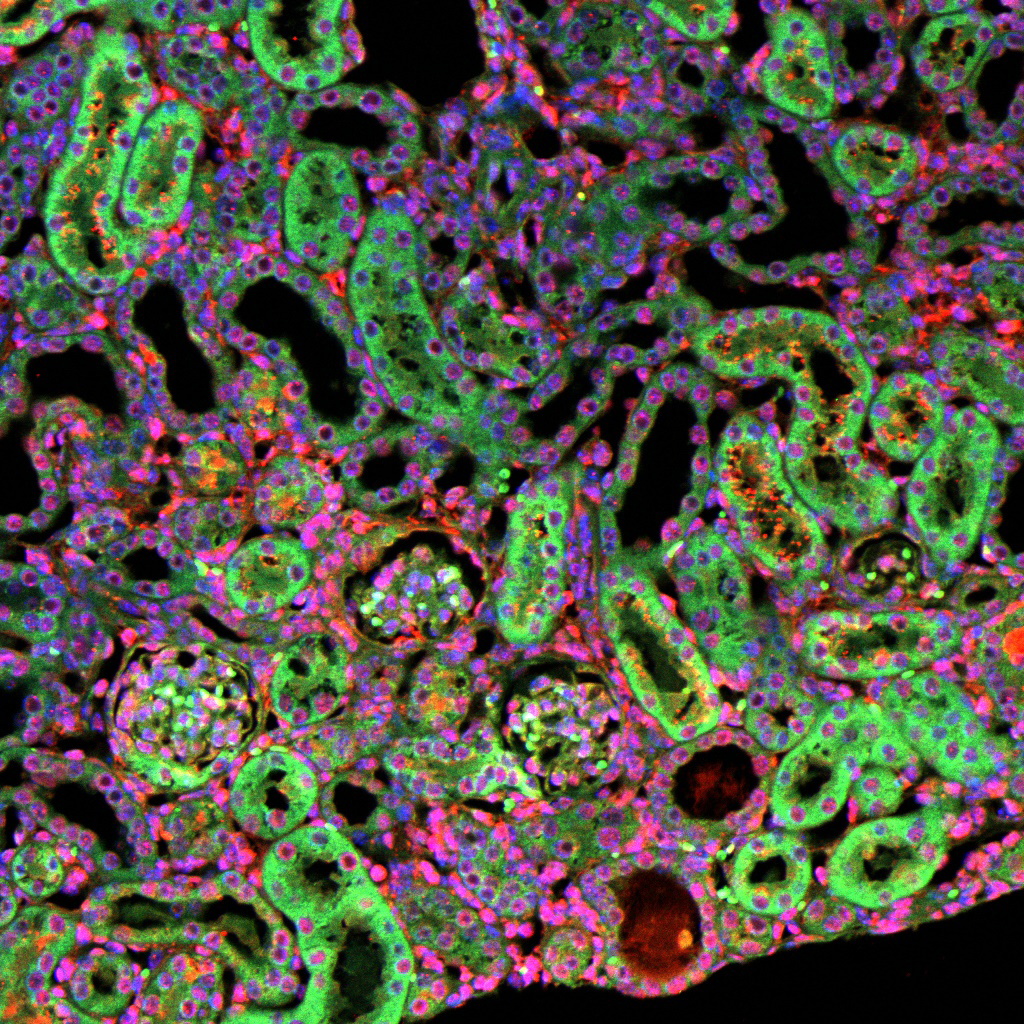

Supplement: Supplementary file 5 — Source data Fig. 3 [file 44321_2025_243_MOESM5_ESM.zip › 3C/UUO merge.tif]

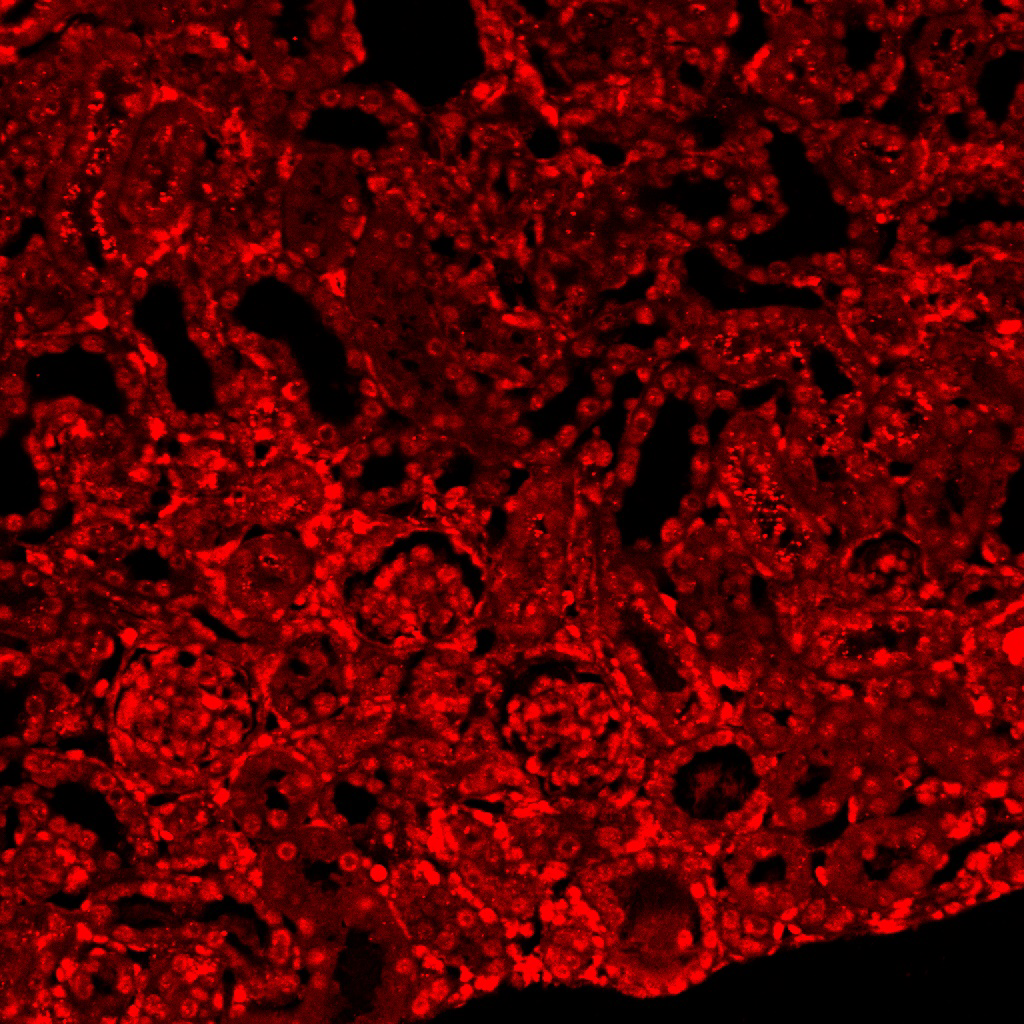

Supplement: Supplementary file 5 — Source data Fig. 3 [file 44321_2025_243_MOESM5_ESM.zip › 3C/UUO p300.tif]

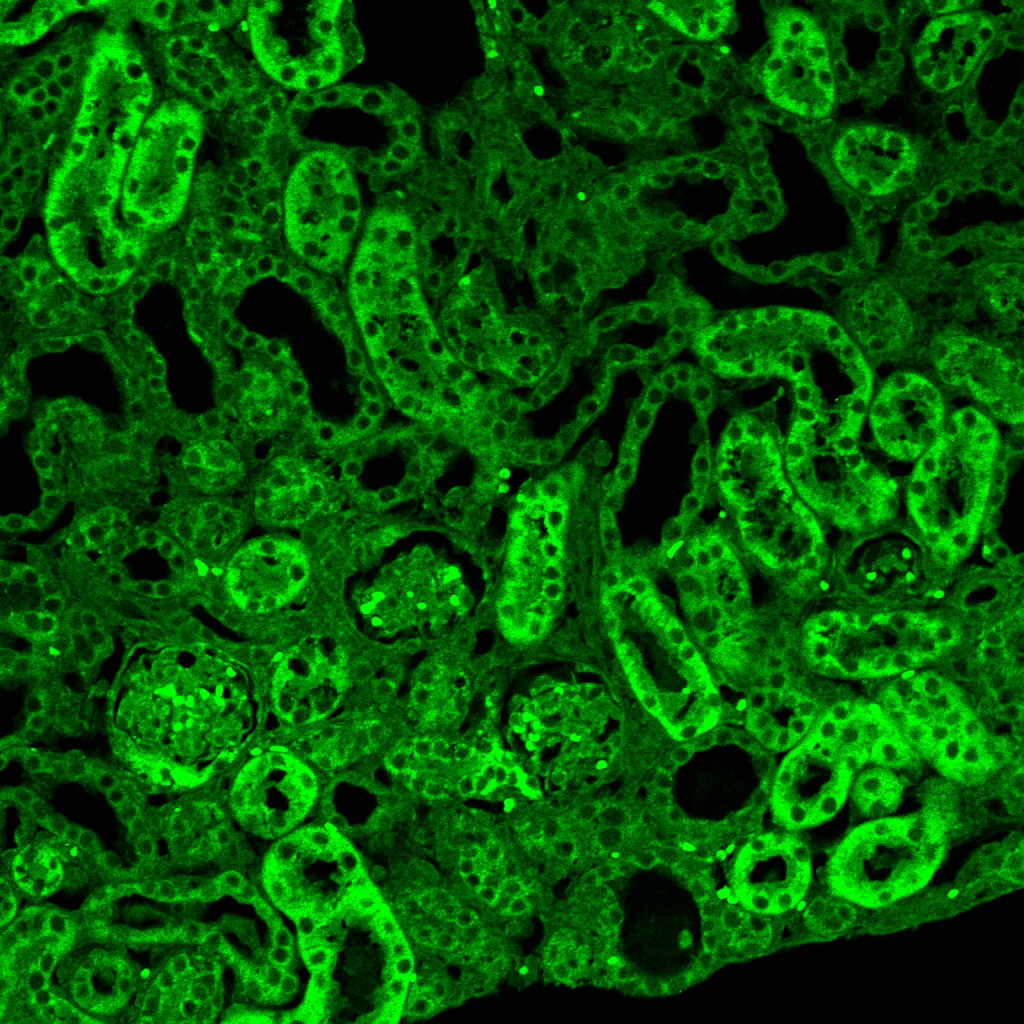

Supplement: Supplementary file 5 — Source data Fig. 3 [file 44321_2025_243_MOESM5_ESM.zip › 3C/UUO PPM1K.tif]

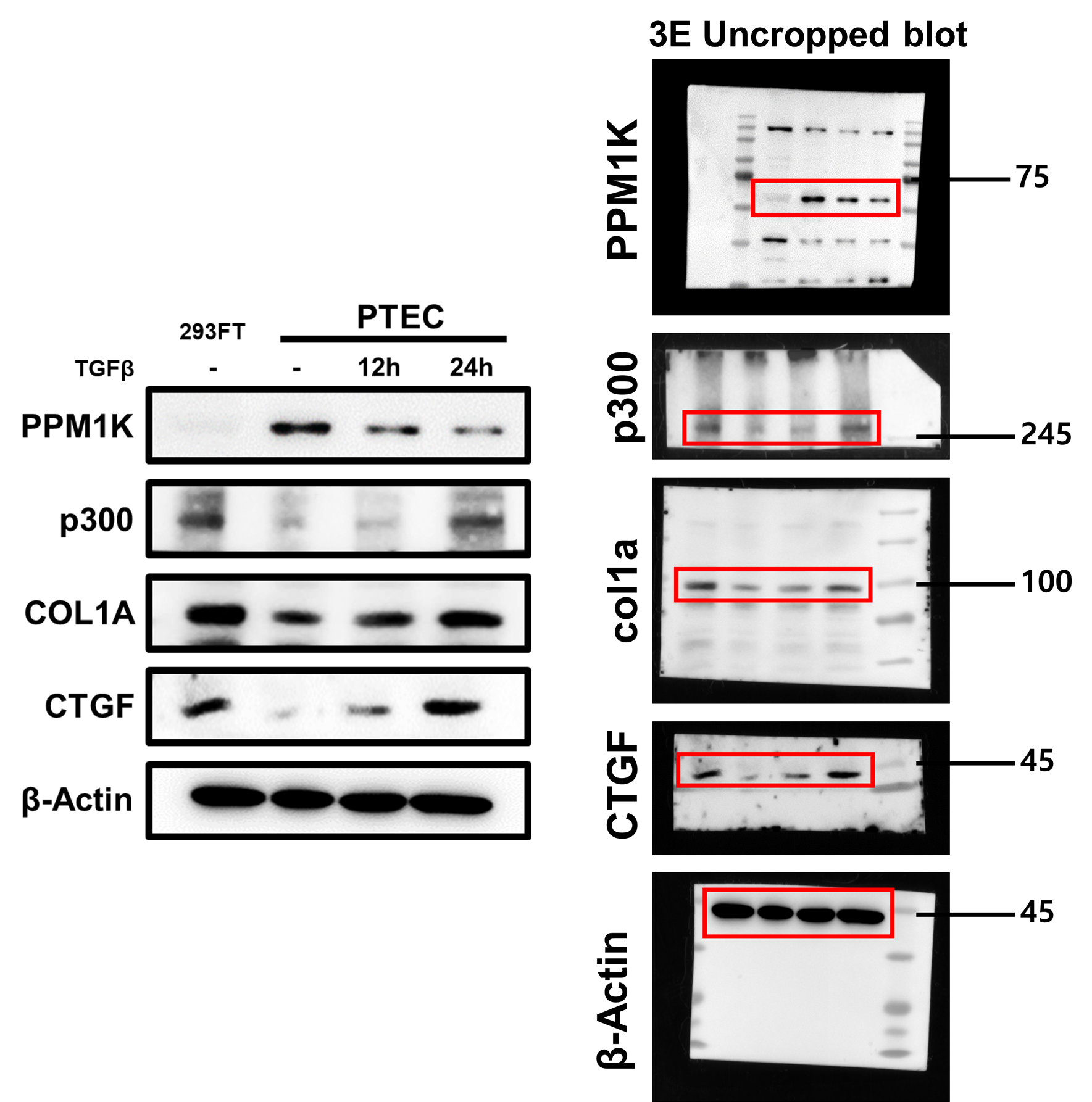

Supplement: Supplementary file 5 — Source data Fig. 3 [file 44321_2025_243_MOESM5_ESM.zip › 3E/3E_Uncropped blot.tif]

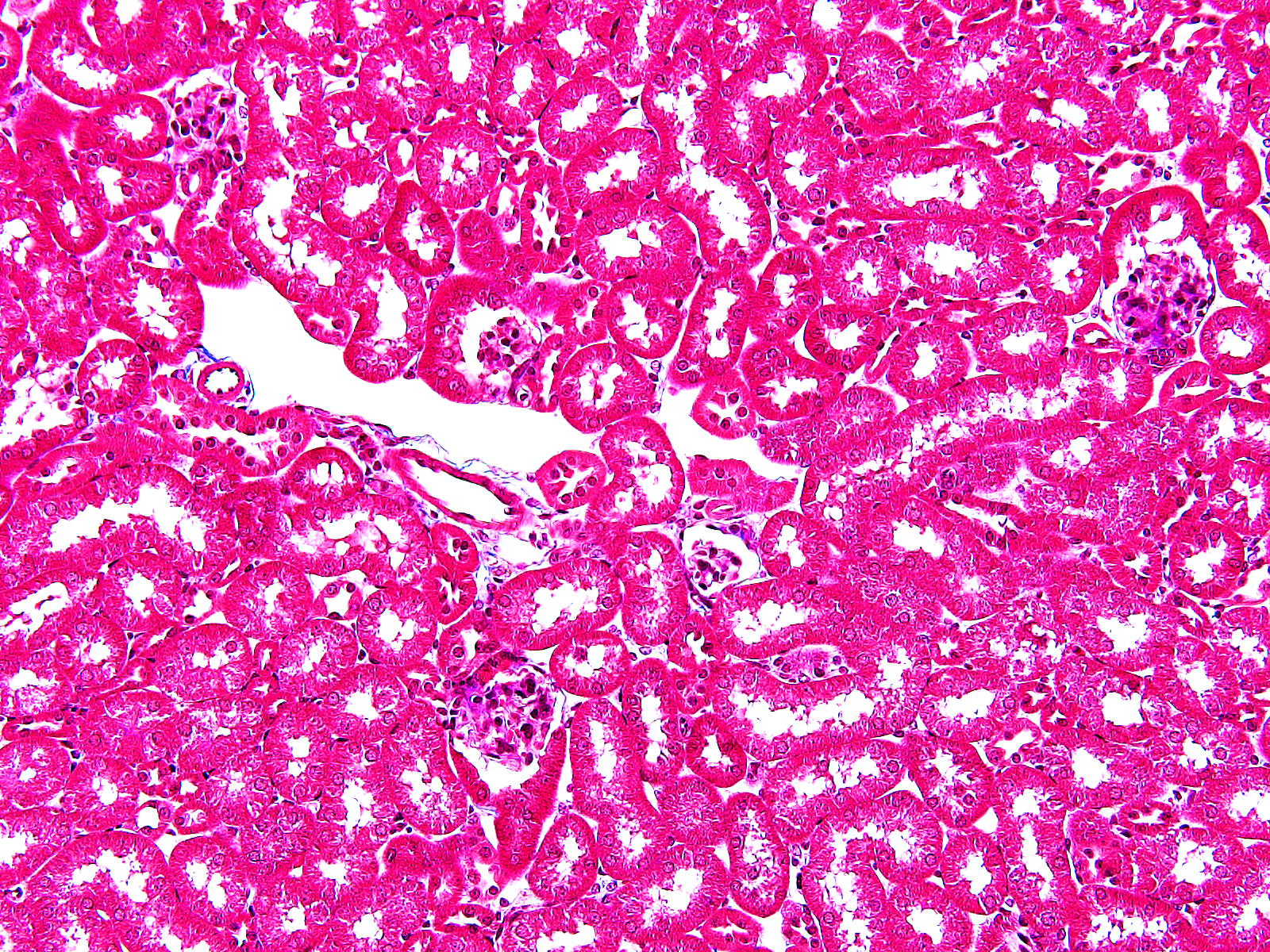

Supplement: Supplementary file 5 — Source data Fig. 3 [file 44321_2025_243_MOESM5_ESM.zip › 3F/MTS sham.tif]

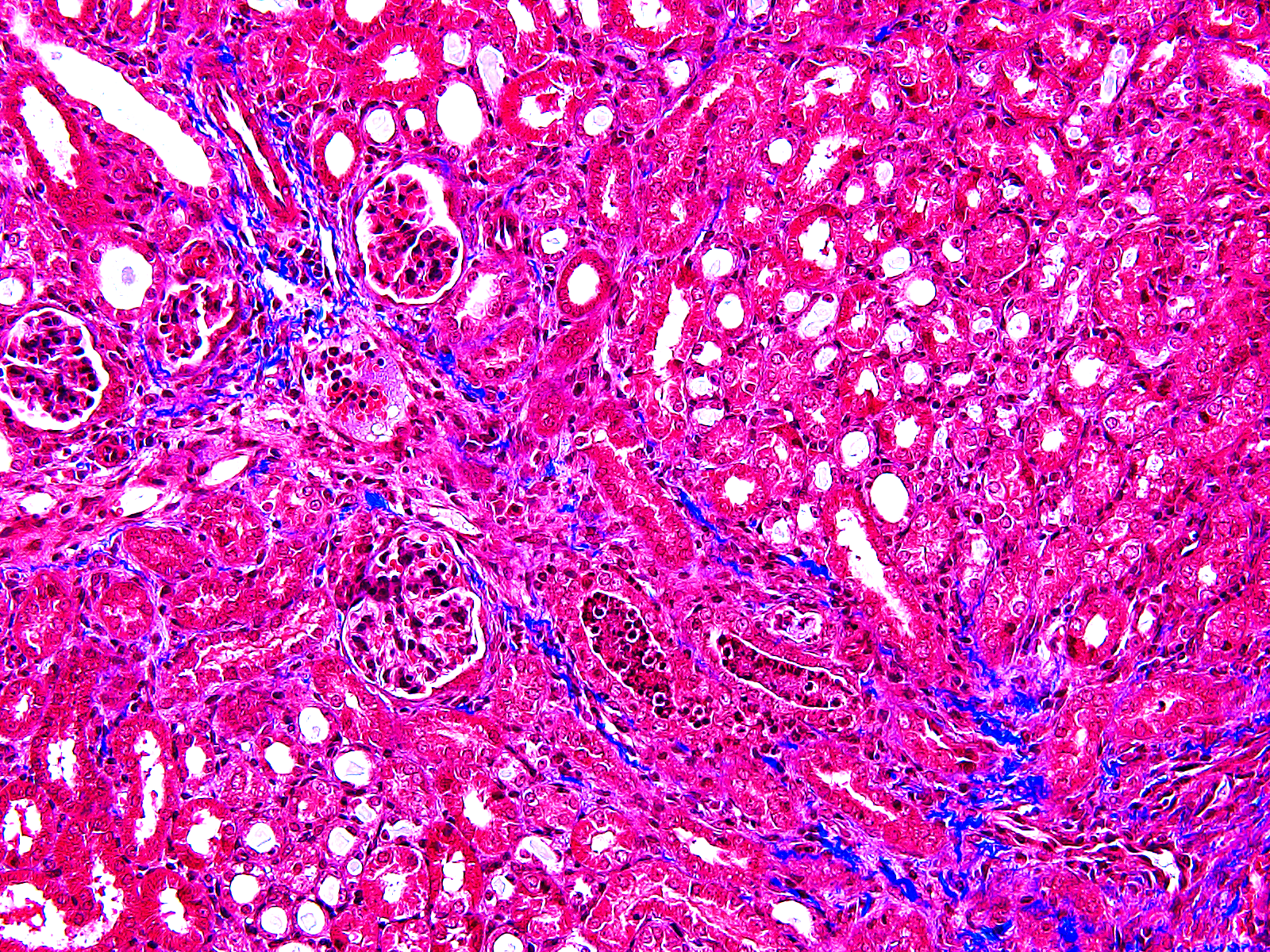

Supplement: Supplementary file 5 — Source data Fig. 3 [file 44321_2025_243_MOESM5_ESM.zip › 3F/MTS UUO+Ad5-empty.tif]

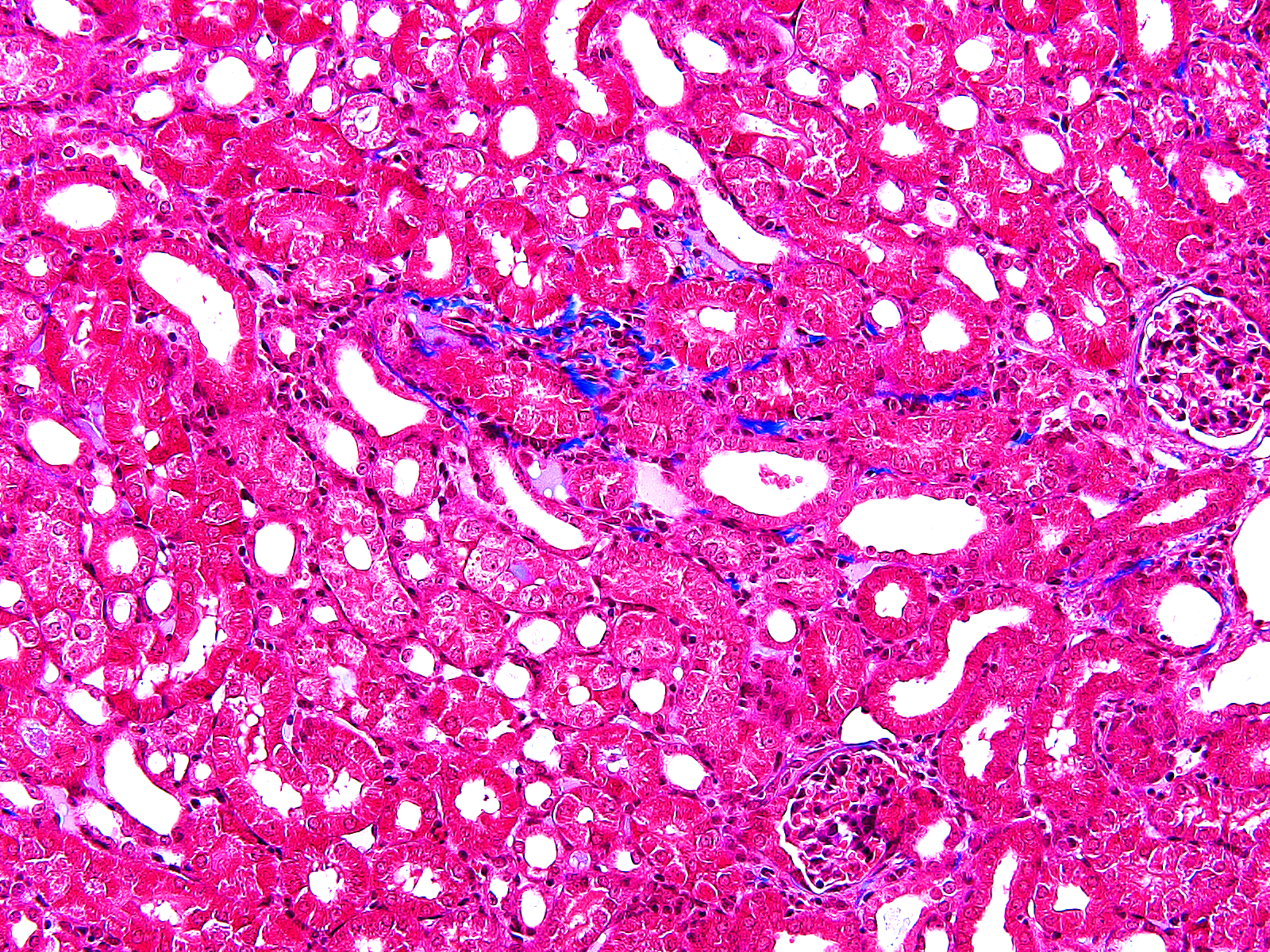

Supplement: Supplementary file 5 — Source data Fig. 3 [file 44321_2025_243_MOESM5_ESM.zip › 3F/MTS UUO+Ad5-PPM1K.tif]

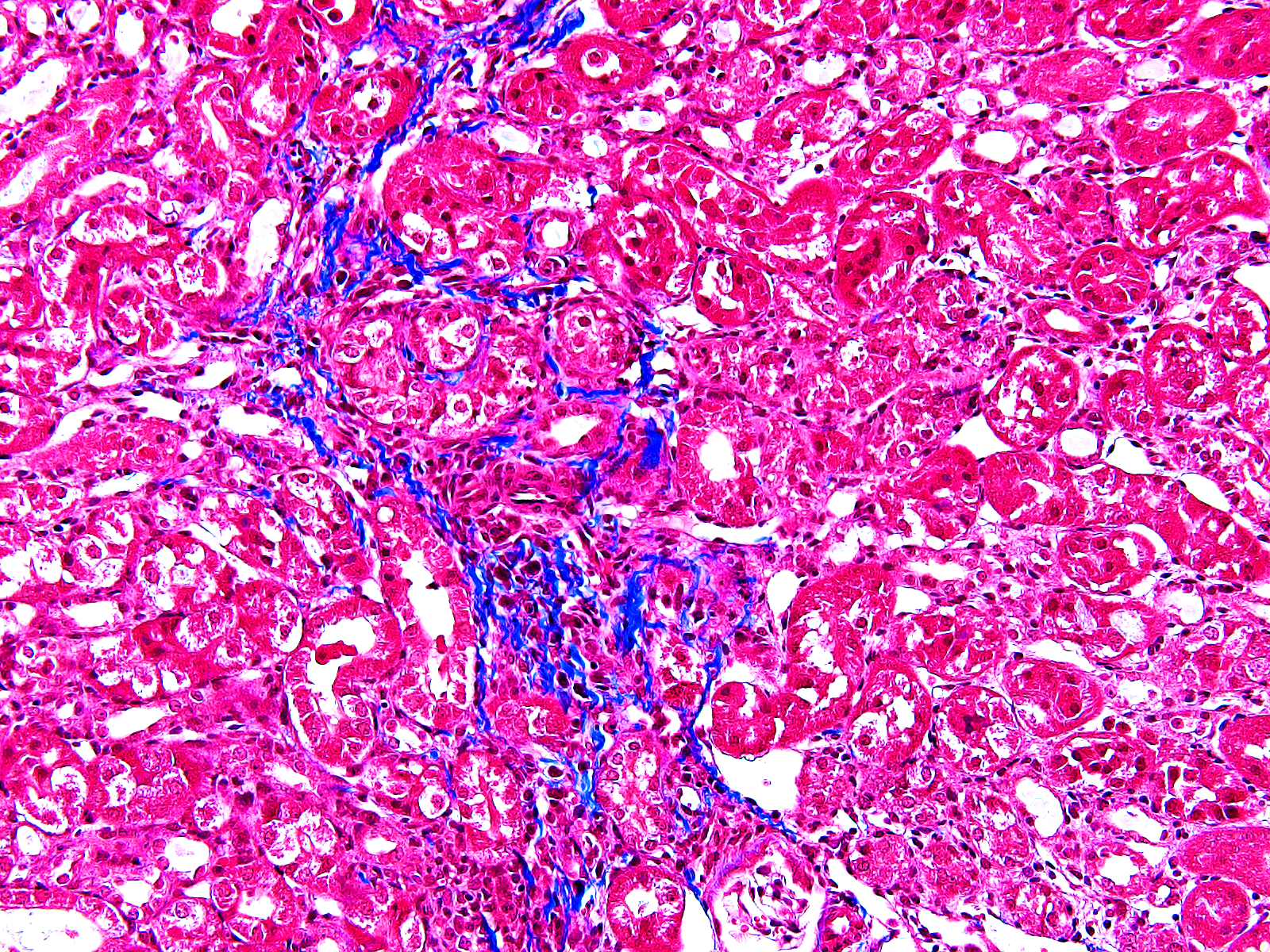

Supplement: Supplementary file 5 — Source data Fig. 3 [file 44321_2025_243_MOESM5_ESM.zip › 3F/MTS UUO+PPM1K N94K.tif]

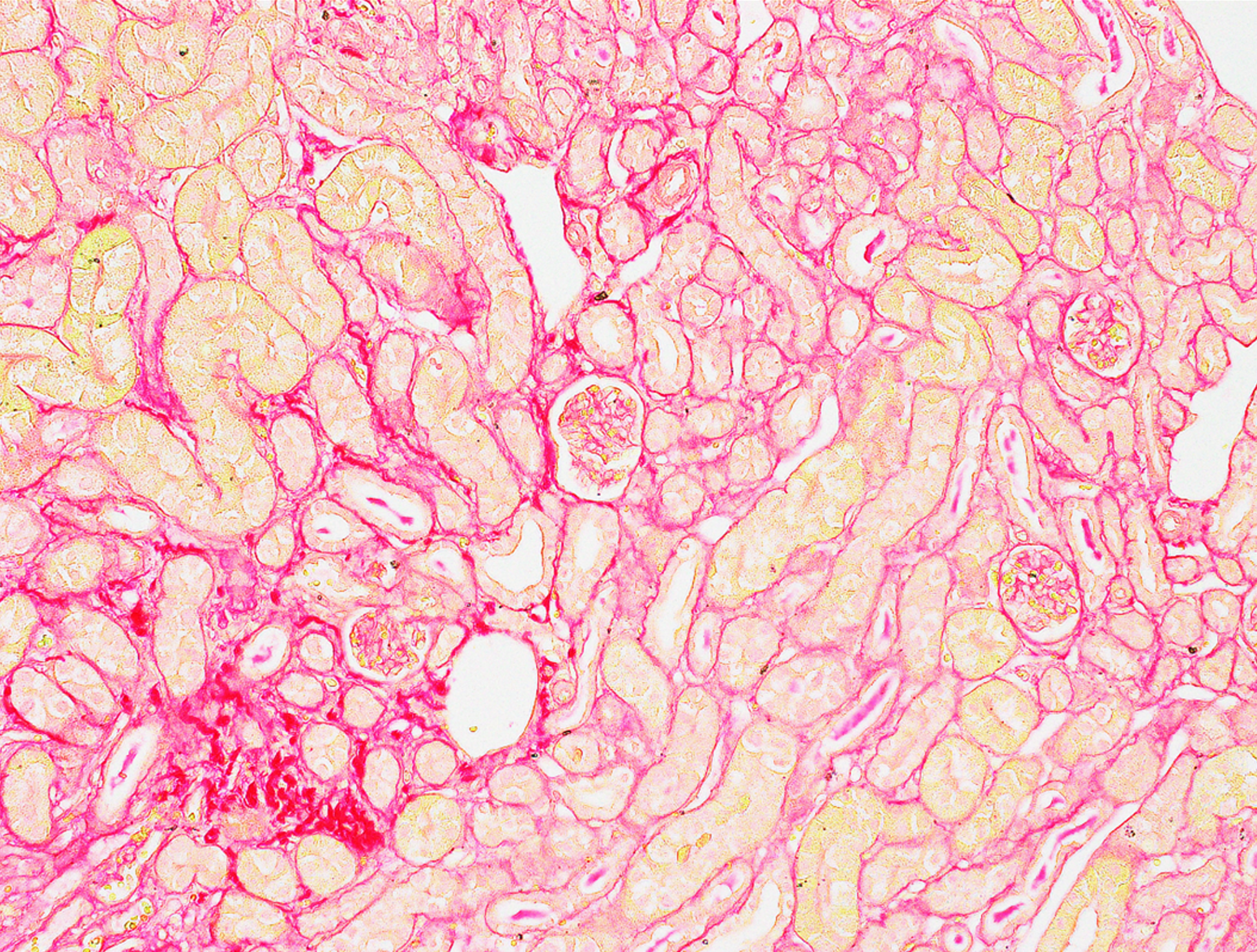

Supplement: Supplementary file 5 — Source data Fig. 3 [file 44321_2025_243_MOESM5_ESM.zip › 3F/Sirius red UUO+Ad5-PPM1K N94K .tif]

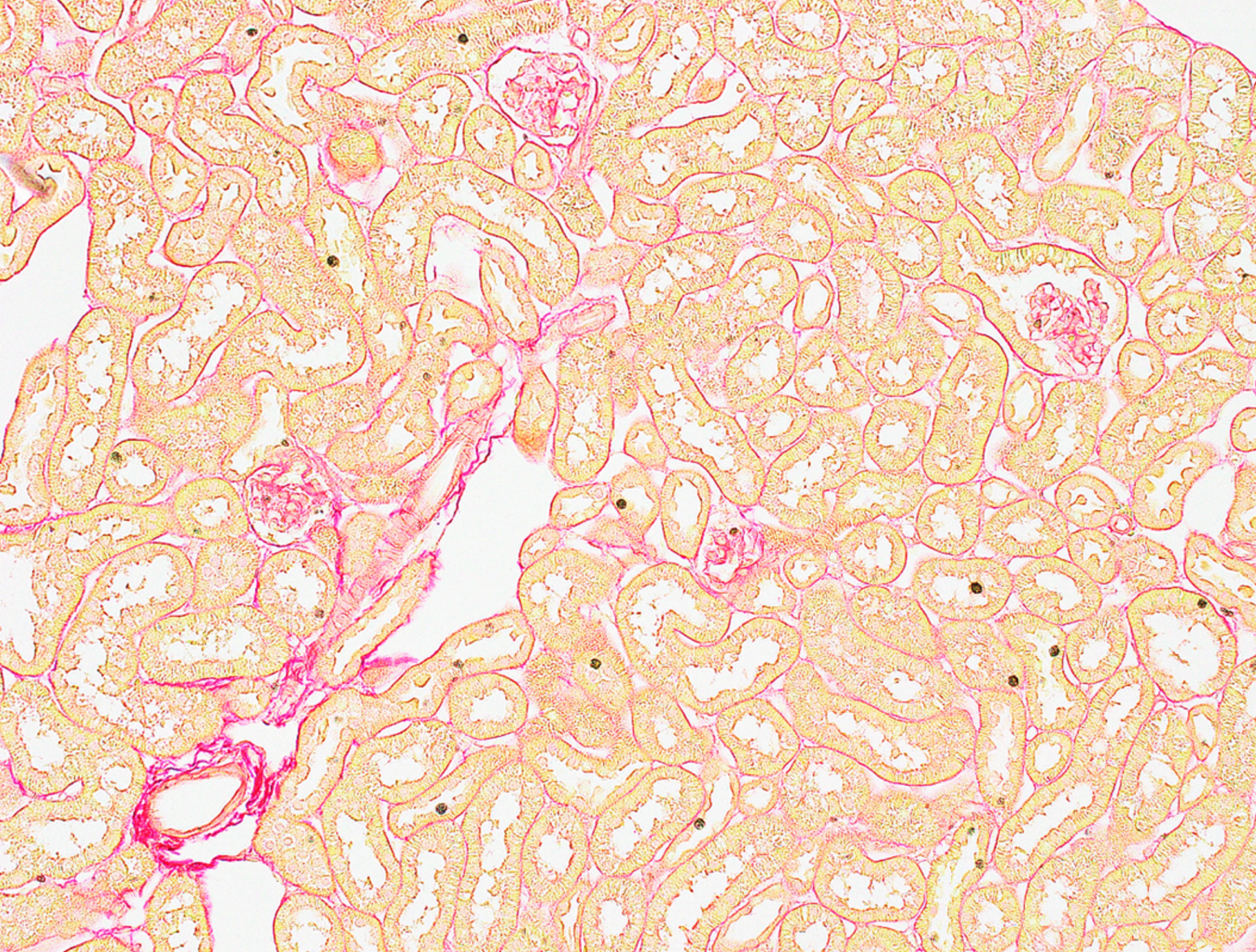

Supplement: Supplementary file 5 — Source data Fig. 3 [file 44321_2025_243_MOESM5_ESM.zip › 3F/Sirius red Sham.tif]

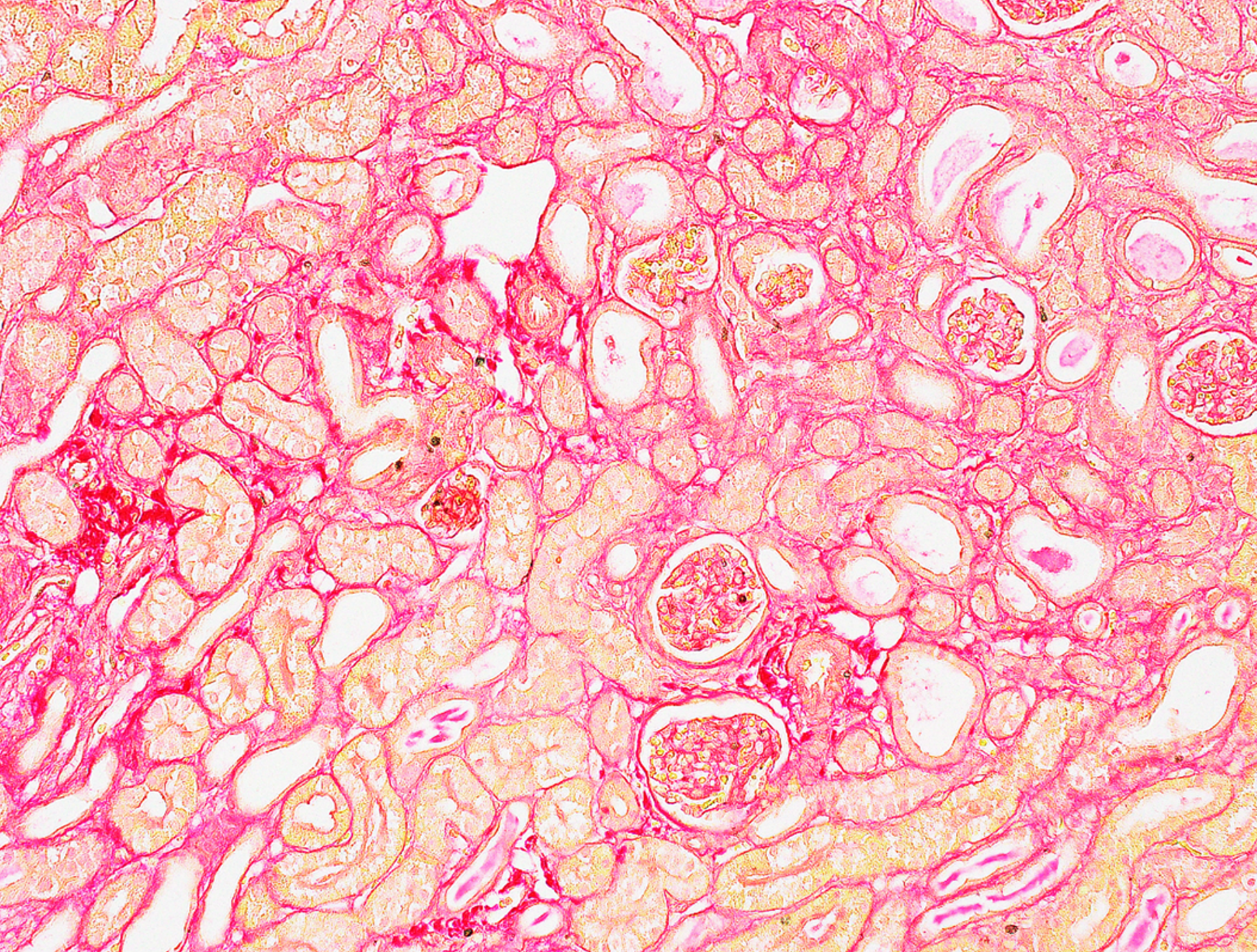

Supplement: Supplementary file 5 — Source data Fig. 3 [file 44321_2025_243_MOESM5_ESM.zip › 3F/Sirius red UUO+Ad5-empty.tif]

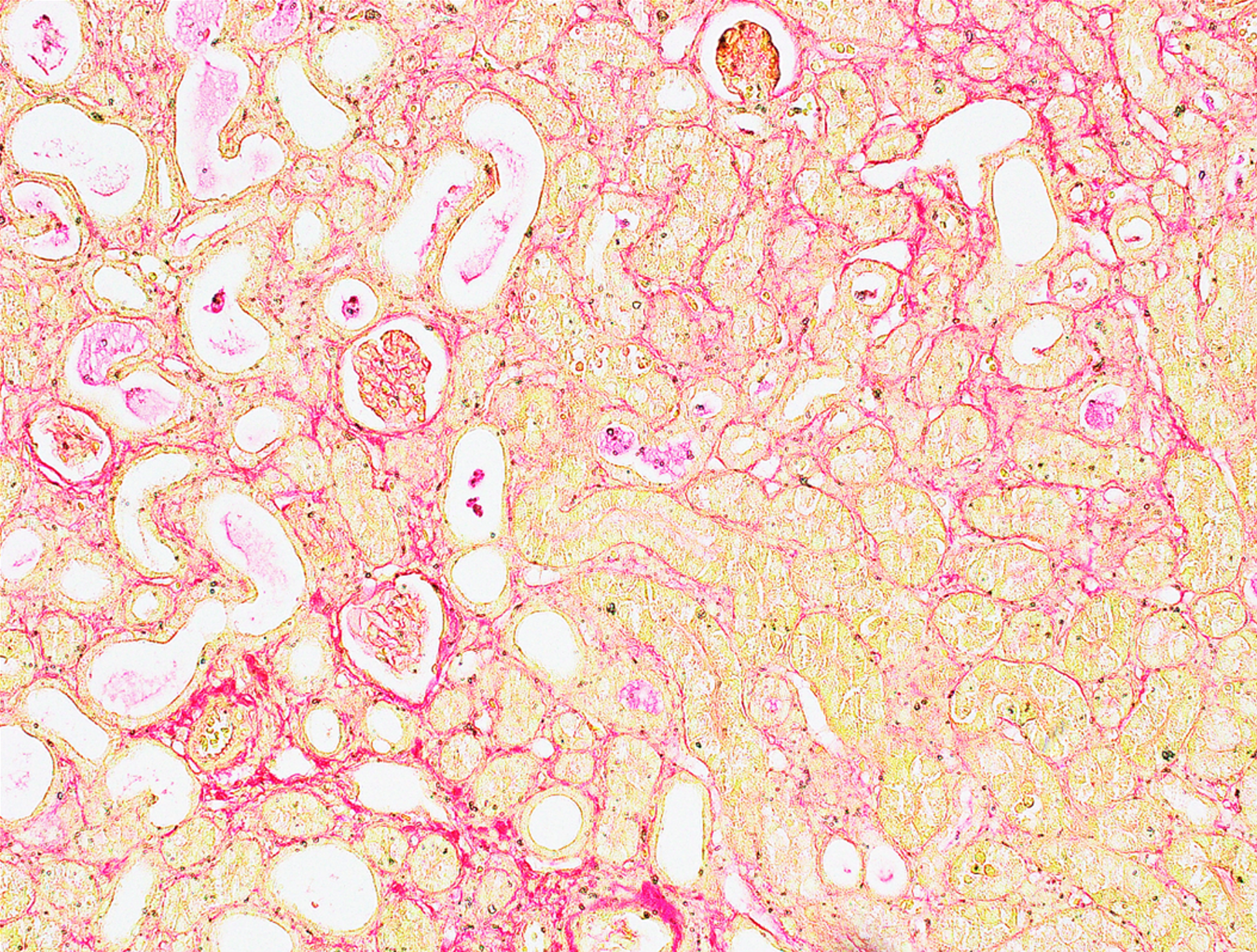

Supplement: Supplementary file 5 — Source data Fig. 3 [file 44321_2025_243_MOESM5_ESM.zip › 3F/Sirius red UUO+Ad5-PPM1K.tif]

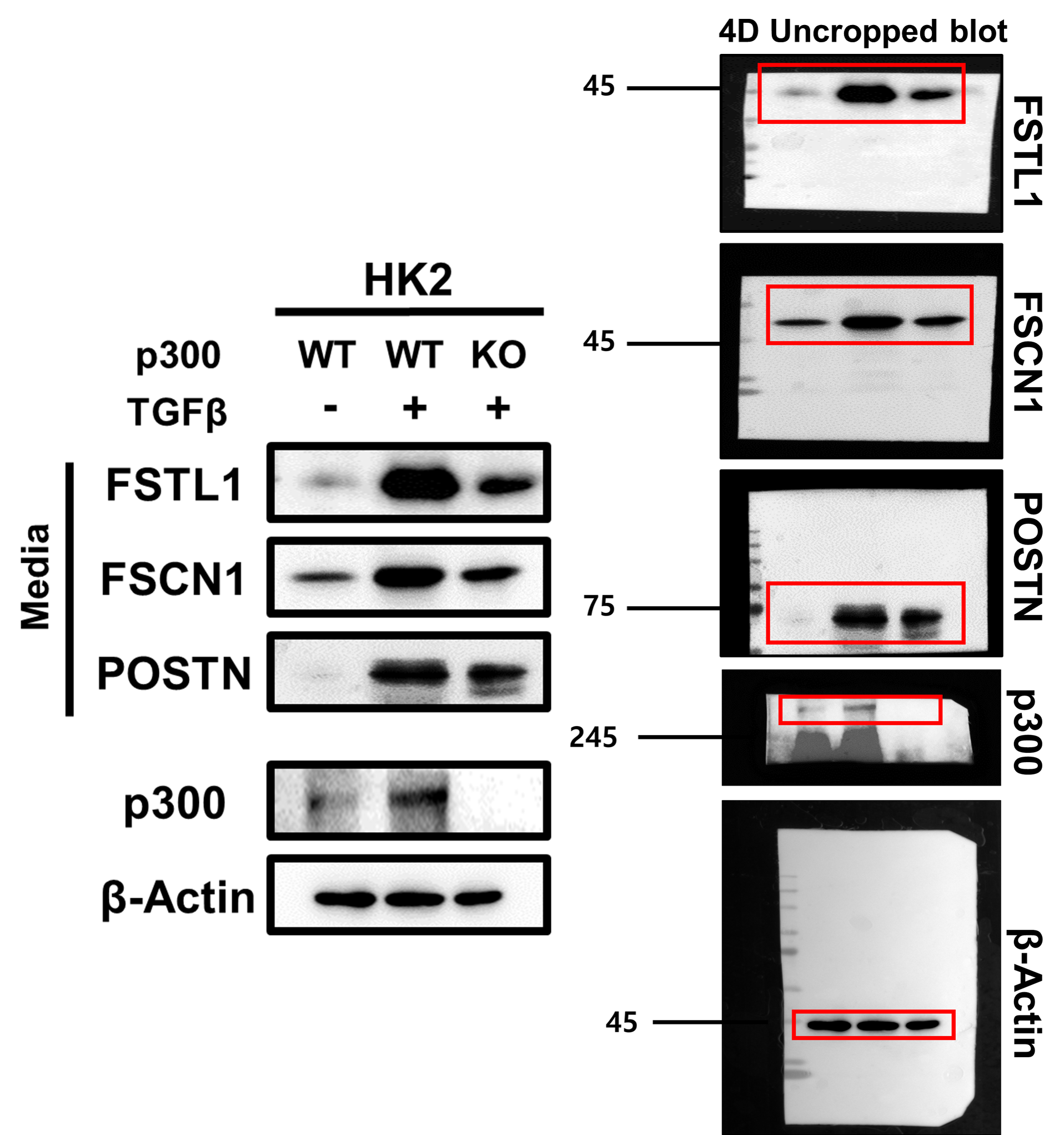

Supplement: Supplementary file 6 — Source data Fig. 4 [file 44321_2025_243_MOESM6_ESM.zip › 4D/4D_Uncropped blot.tif]

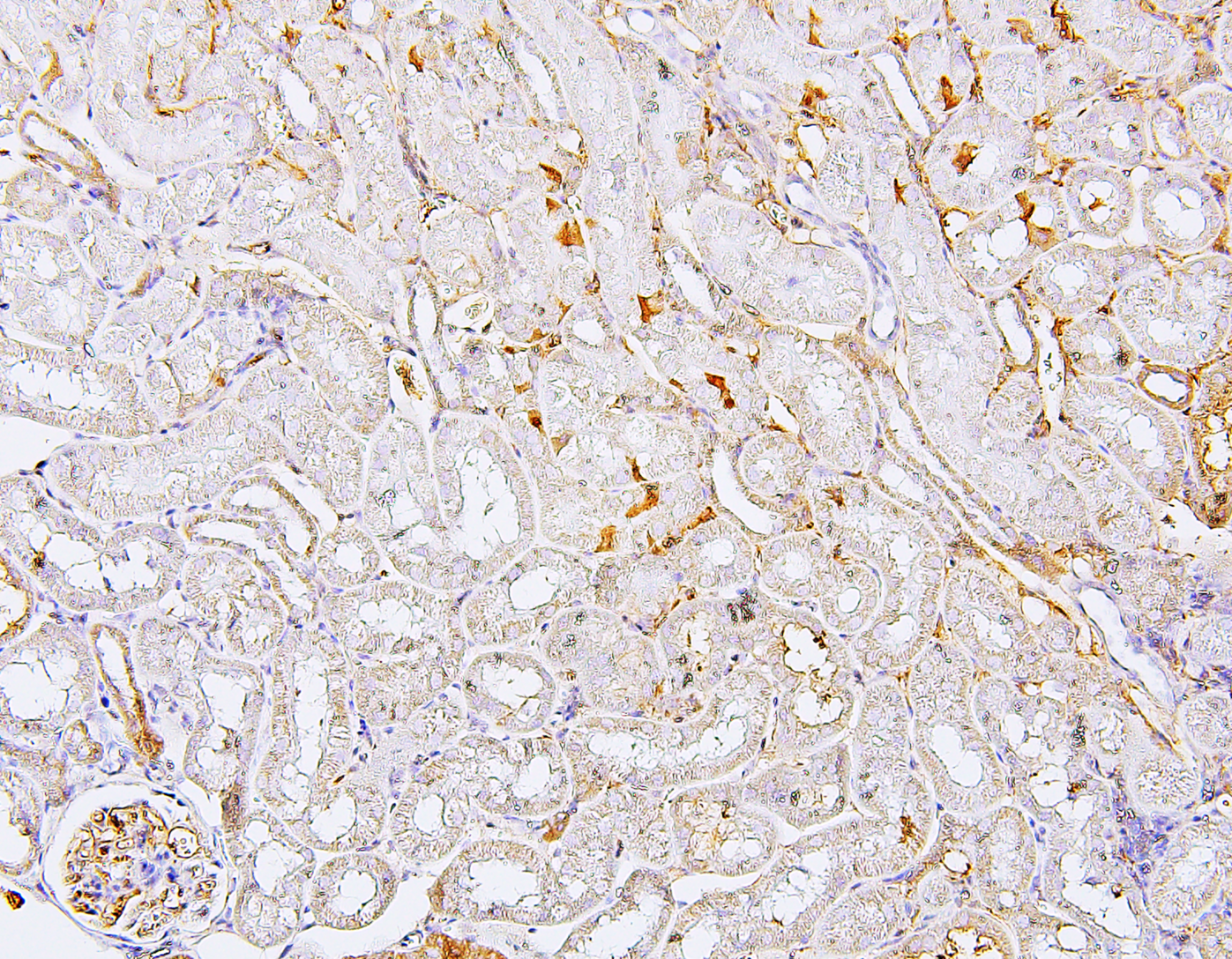

Supplement: Supplementary file 7 — Source data Fig. 5 [file 44321_2025_243_MOESM7_ESM.zip › 5A/Albumin cKO Sham.tif]

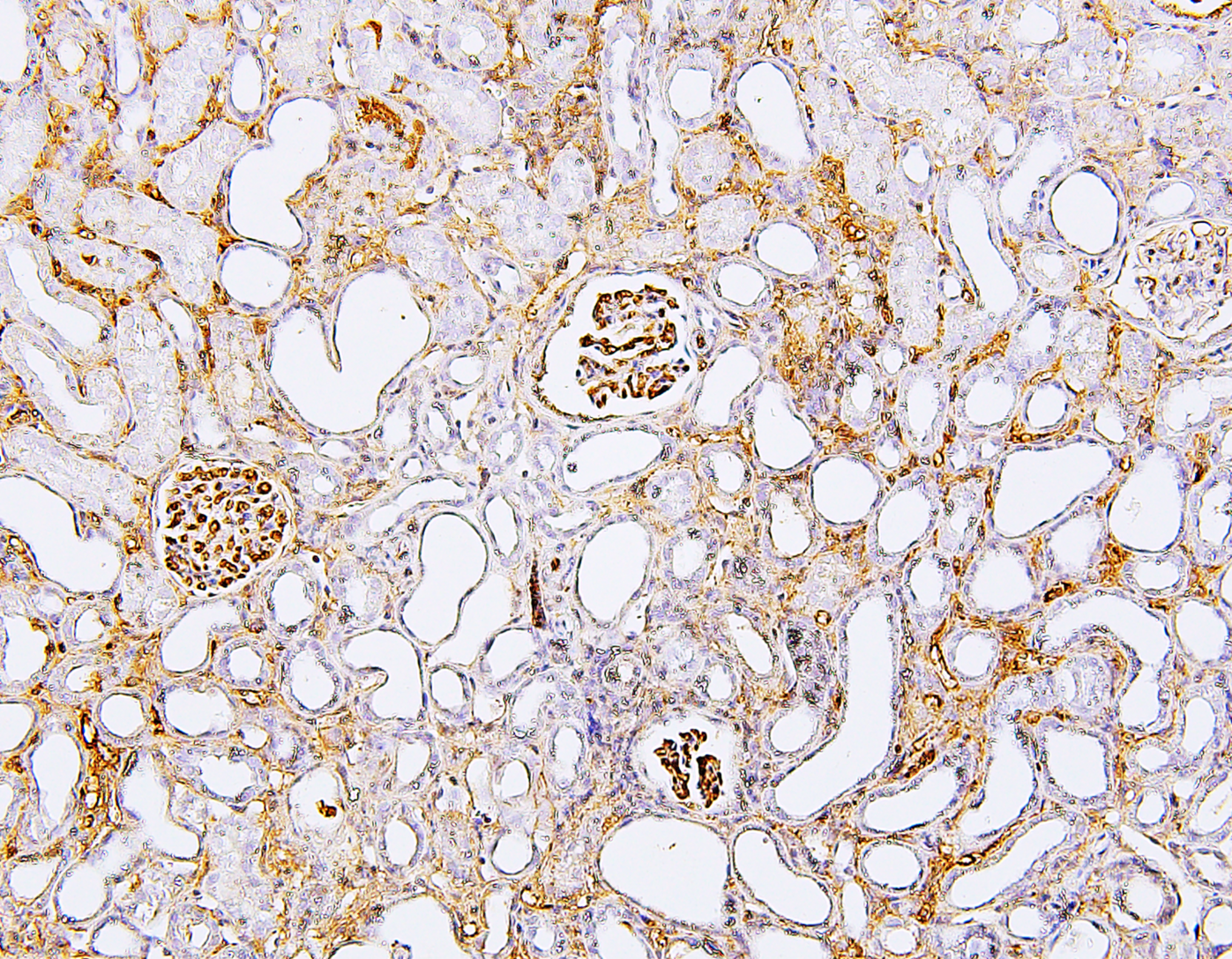

Supplement: Supplementary file 7 — Source data Fig. 5 [file 44321_2025_243_MOESM7_ESM.zip › 5A/Albumin cKO UUO.tif]

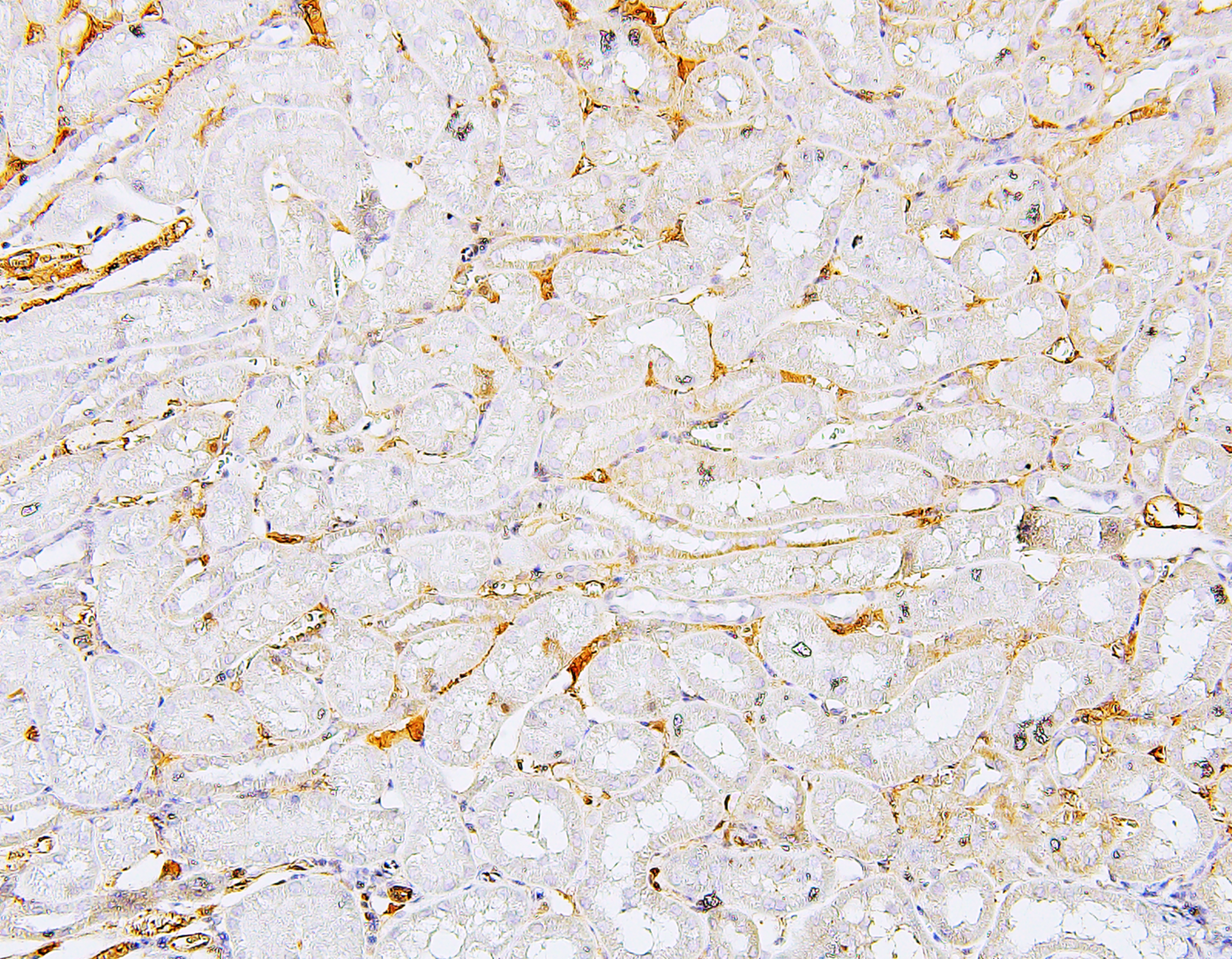

Supplement: Supplementary file 7 — Source data Fig. 5 [file 44321_2025_243_MOESM7_ESM.zip › 5A/Albumin WT sham.tif]

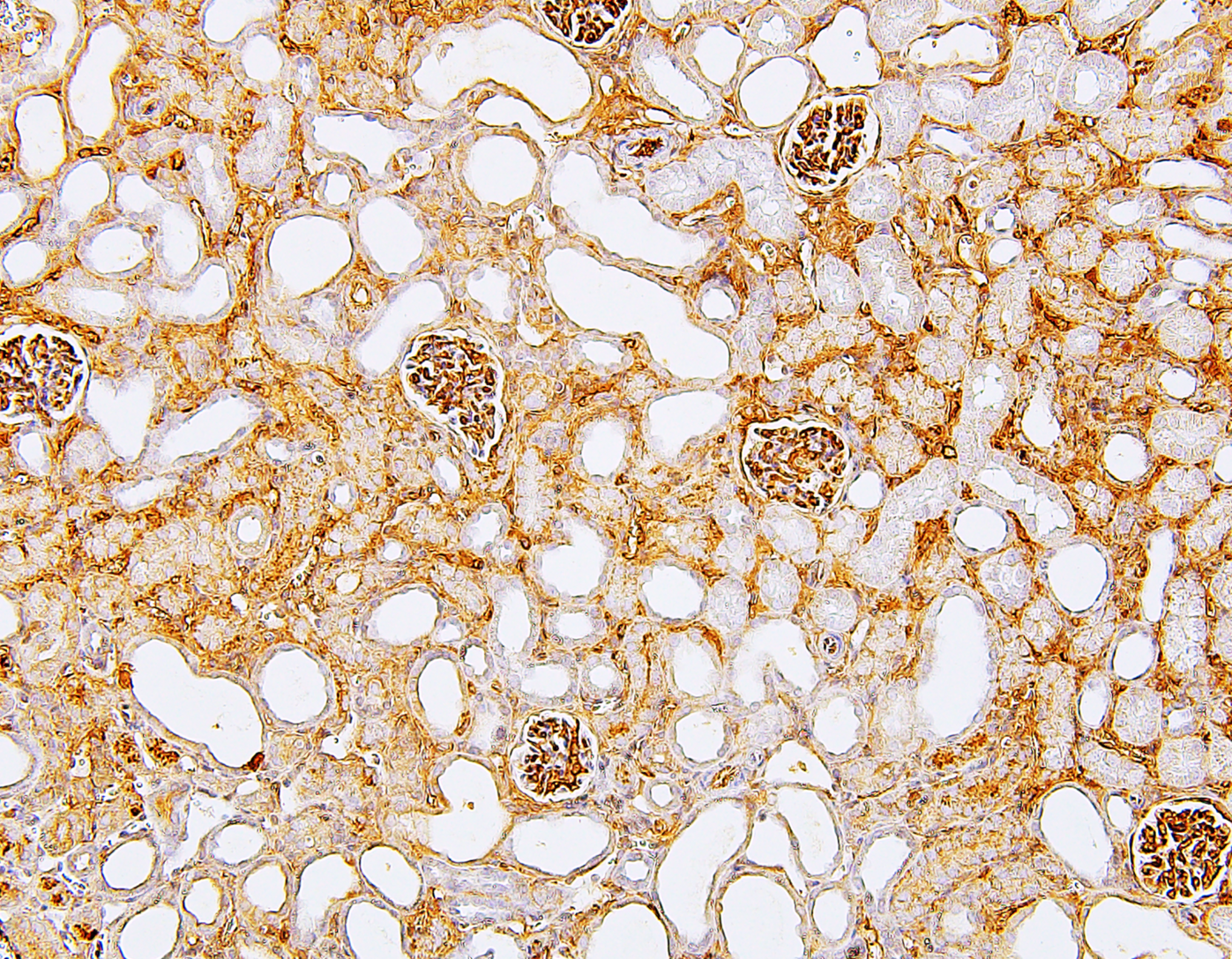

Supplement: Supplementary file 7 — Source data Fig. 5 [file 44321_2025_243_MOESM7_ESM.zip › 5A/Albumin WT UUO.tif]

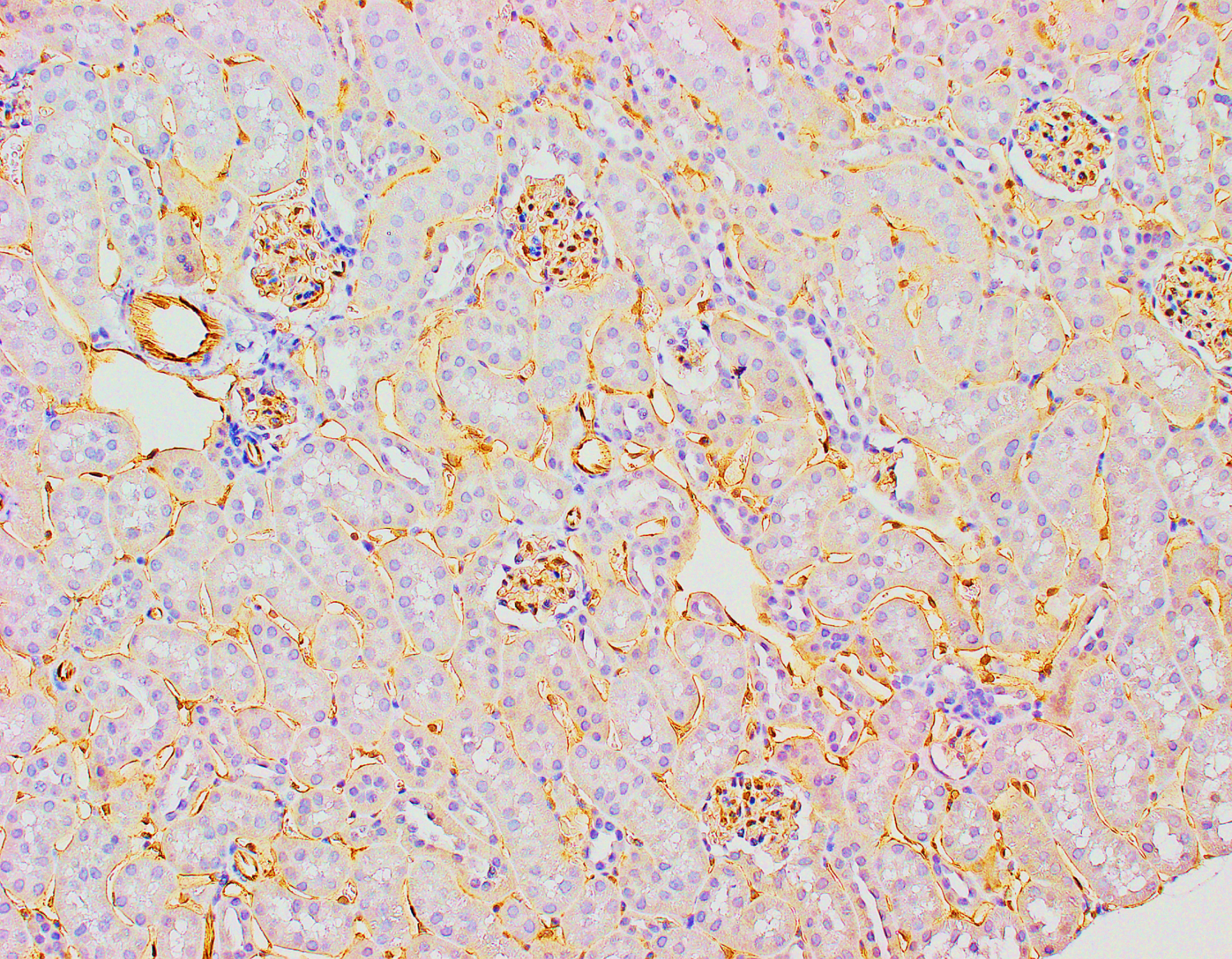

Supplement: Supplementary file 7 — Source data Fig. 5 [file 44321_2025_243_MOESM7_ESM.zip › 5A/CD31 cKO Sham.tif]

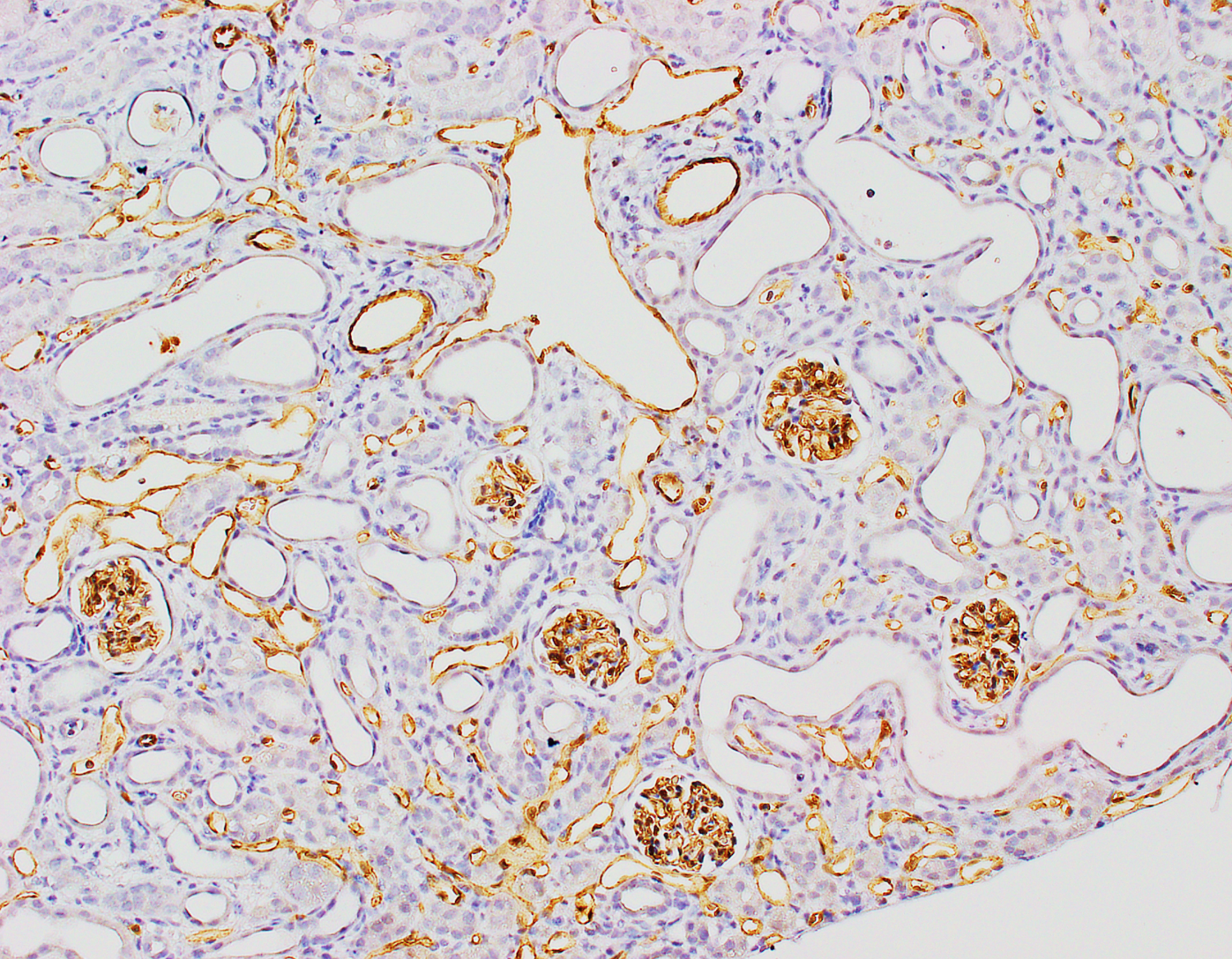

Supplement: Supplementary file 7 — Source data Fig. 5 [file 44321_2025_243_MOESM7_ESM.zip › 5A/CD31 cKO UUO.tif]

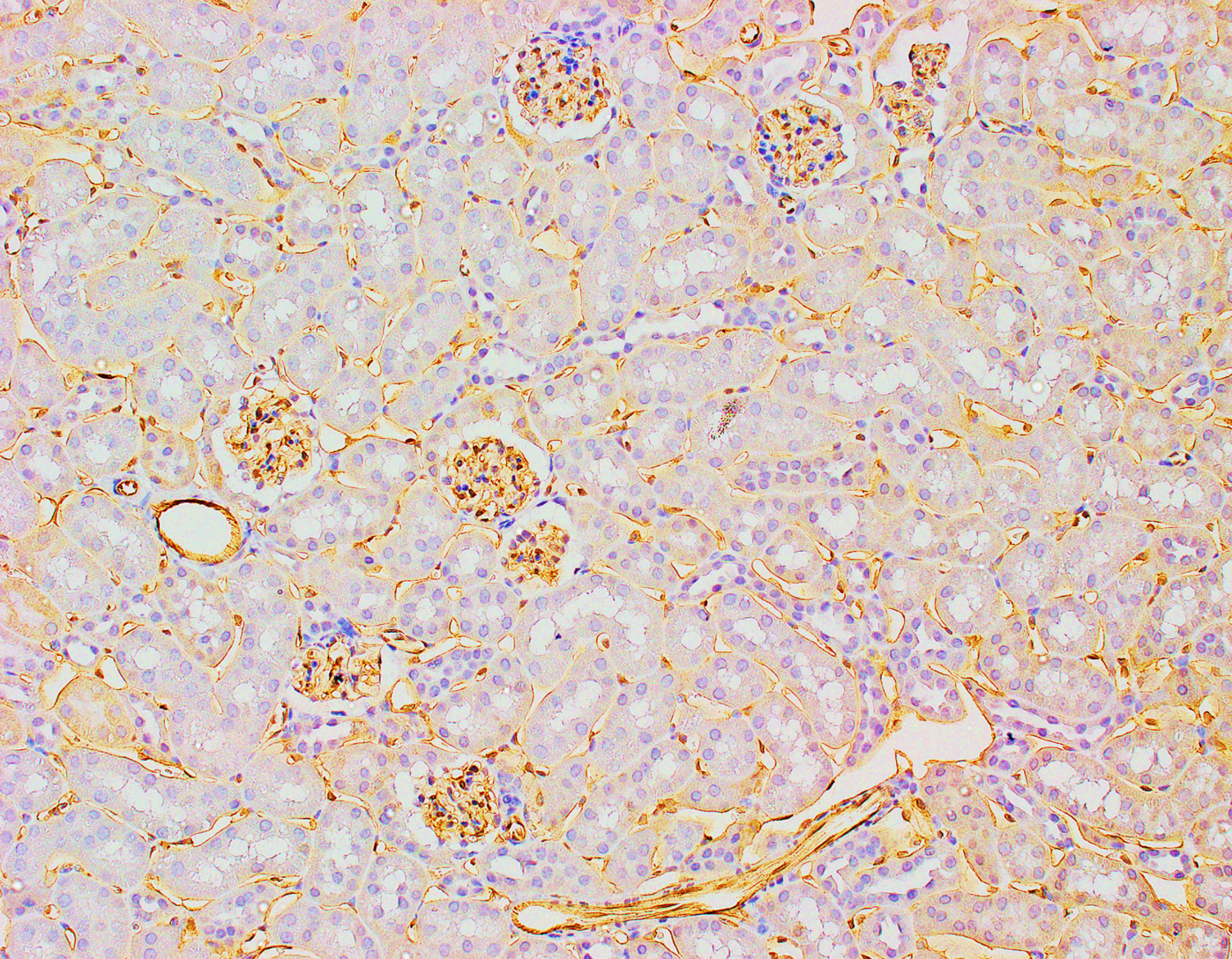

Supplement: Supplementary file 7 — Source data Fig. 5 [file 44321_2025_243_MOESM7_ESM.zip › 5A/CD31 WT sham.tif]

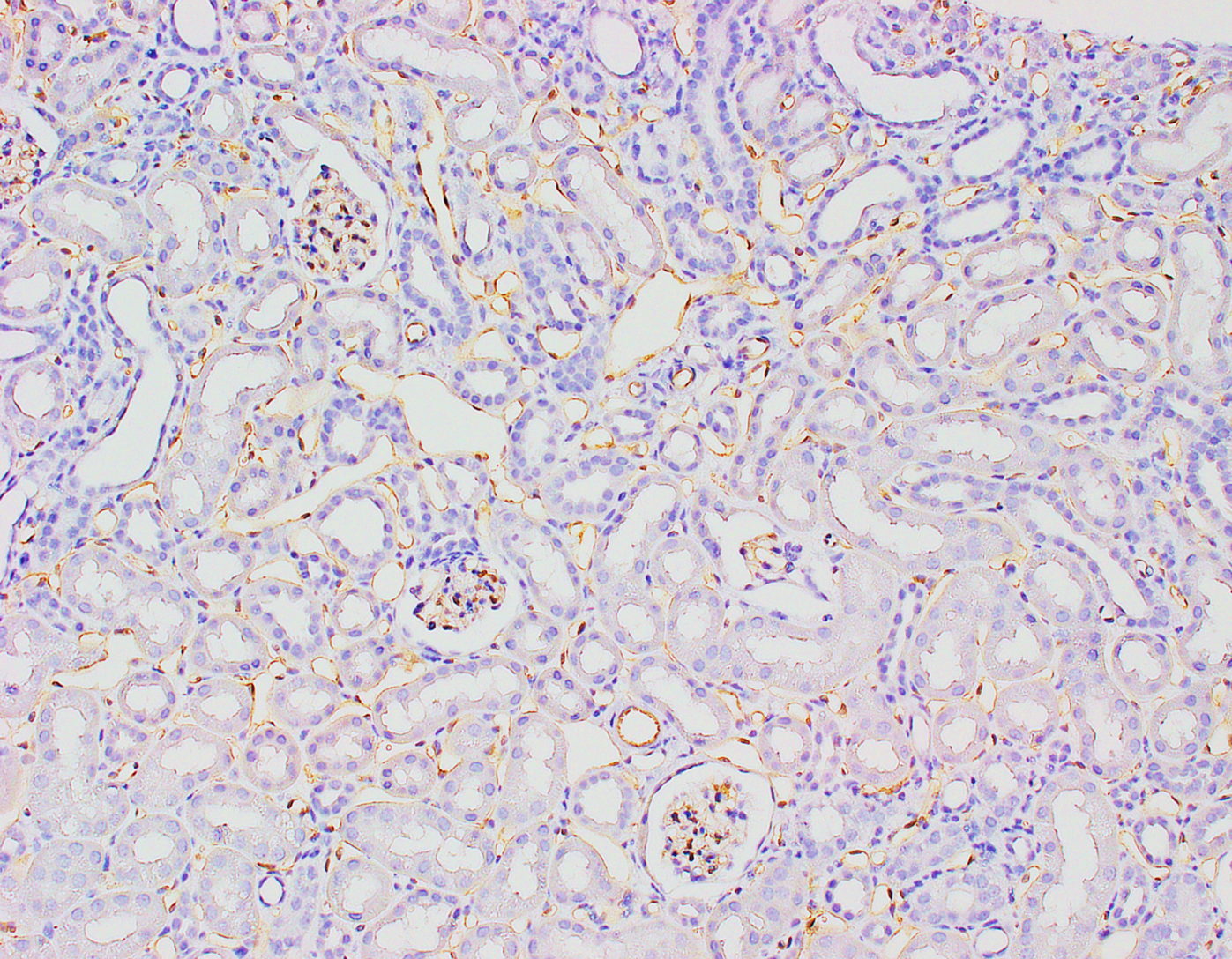

Supplement: Supplementary file 7 — Source data Fig. 5 [file 44321_2025_243_MOESM7_ESM.zip › 5A/CD31 WT UUO.tif]

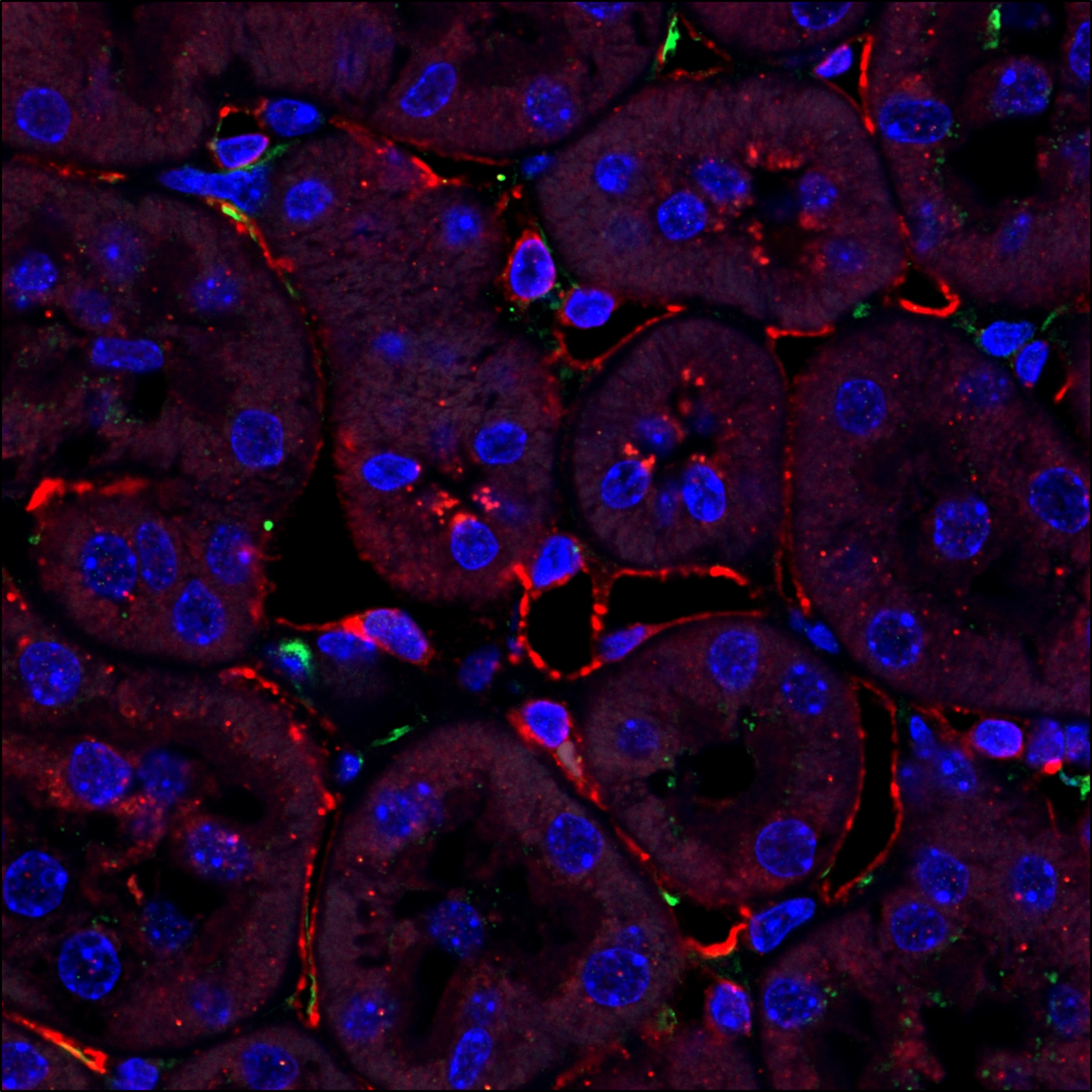

Supplement: Supplementary file 7 — Source data Fig. 5 [file 44321_2025_243_MOESM7_ESM.zip › 5B/Sham cKO.tif]

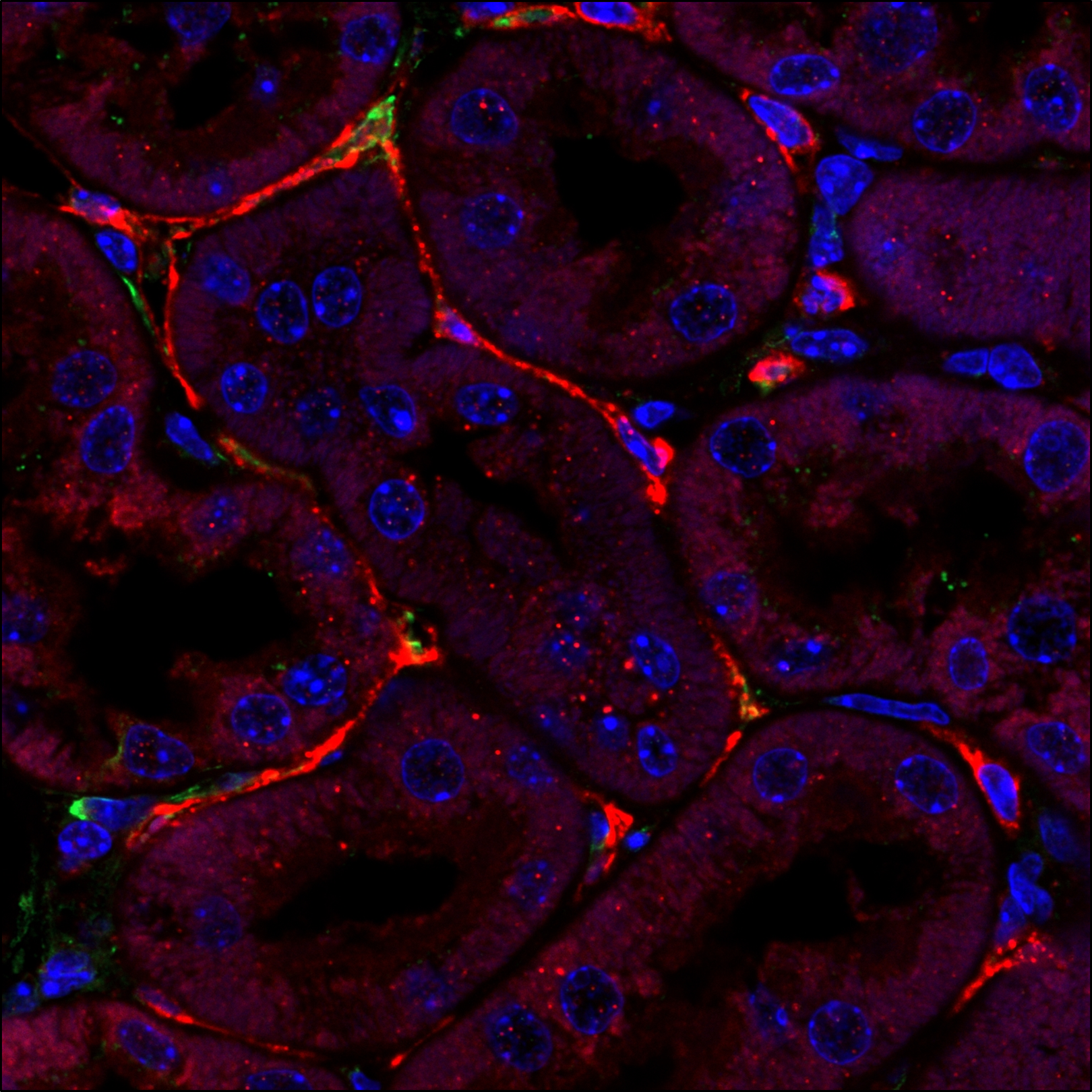

Supplement: Supplementary file 7 — Source data Fig. 5 [file 44321_2025_243_MOESM7_ESM.zip › 5B/Sham WT.tif]

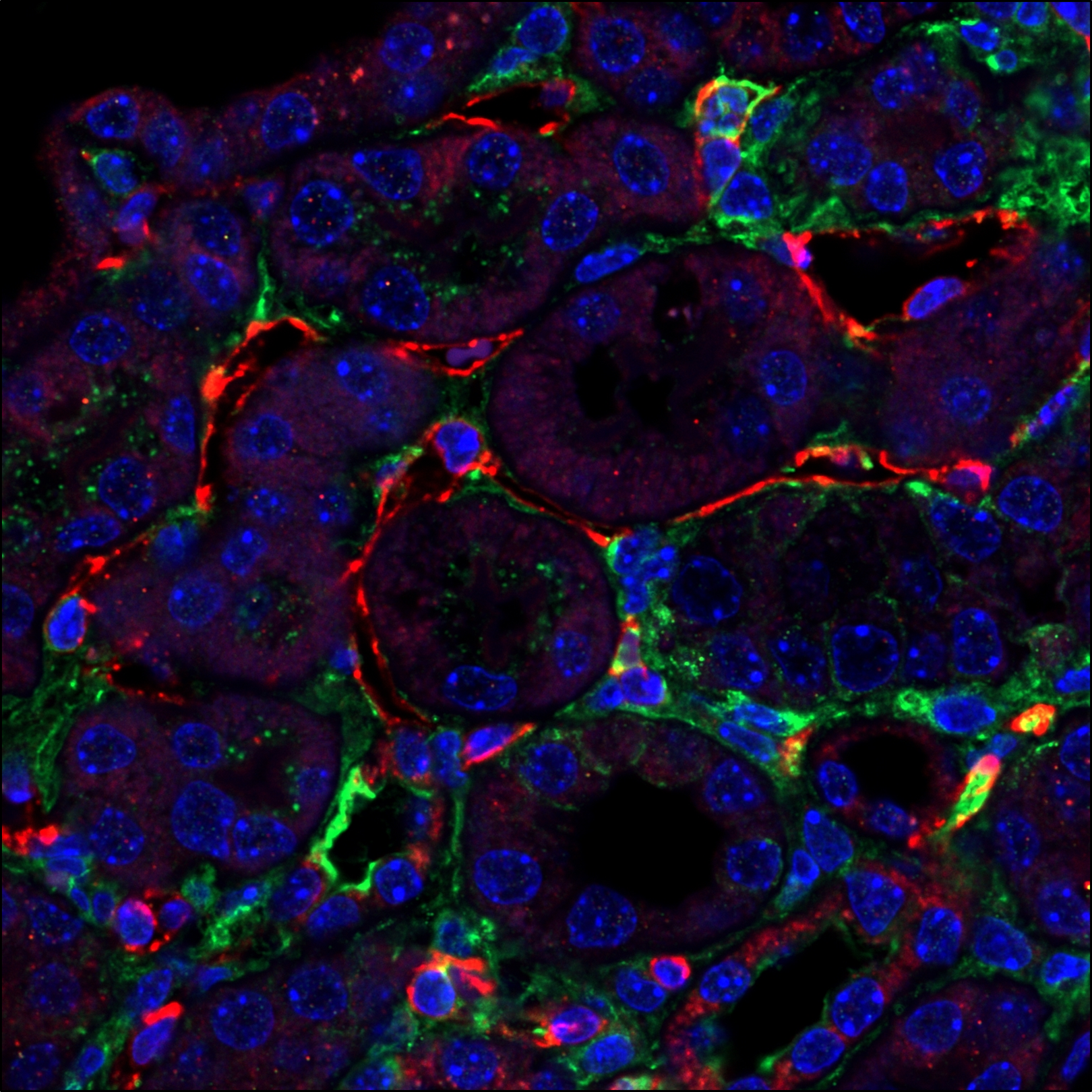

Supplement: Supplementary file 7 — Source data Fig. 5 [file 44321_2025_243_MOESM7_ESM.zip › 5B/UUO cKO.tif]

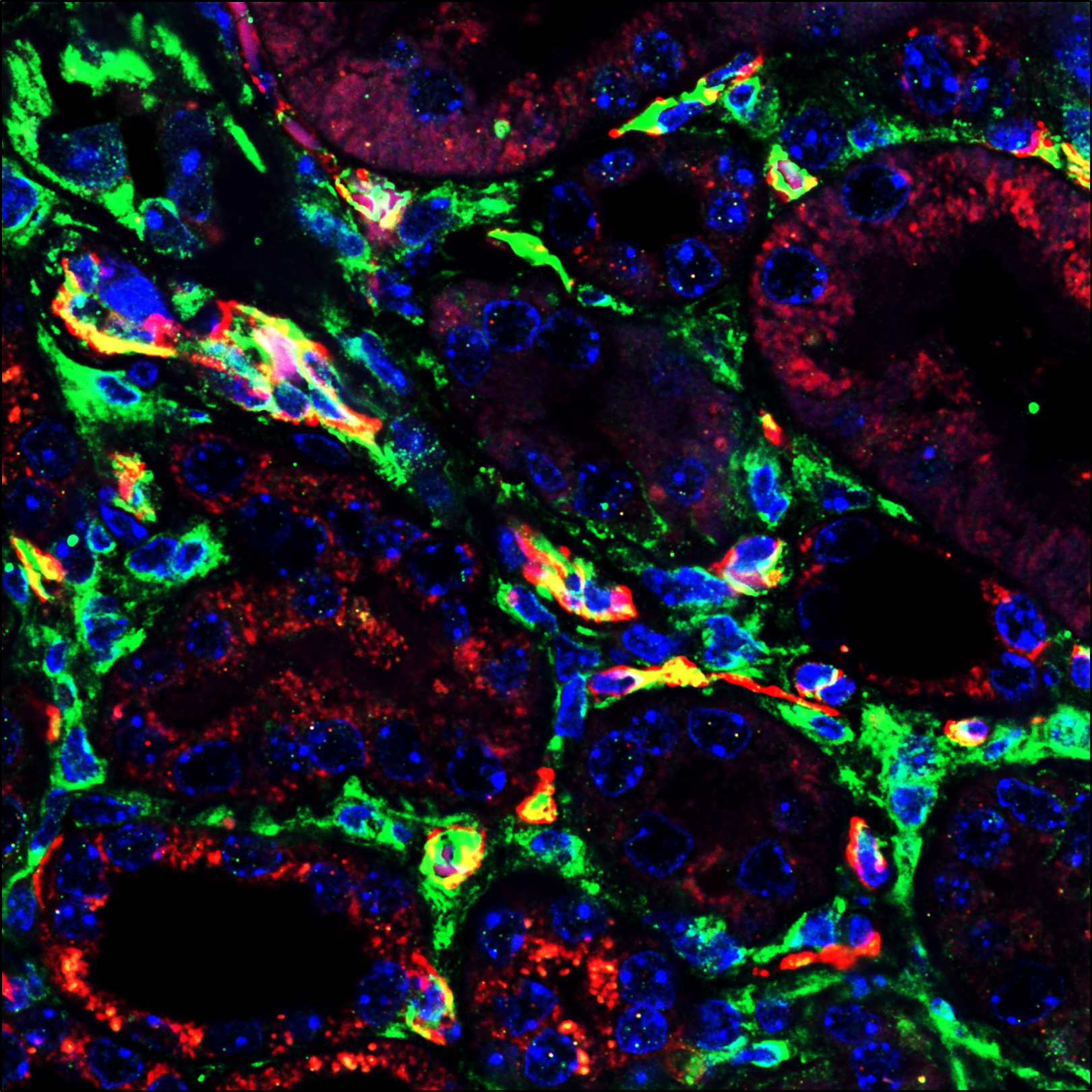

Supplement: Supplementary file 7 — Source data Fig. 5 [file 44321_2025_243_MOESM7_ESM.zip › 5B/UUO WT.tif]

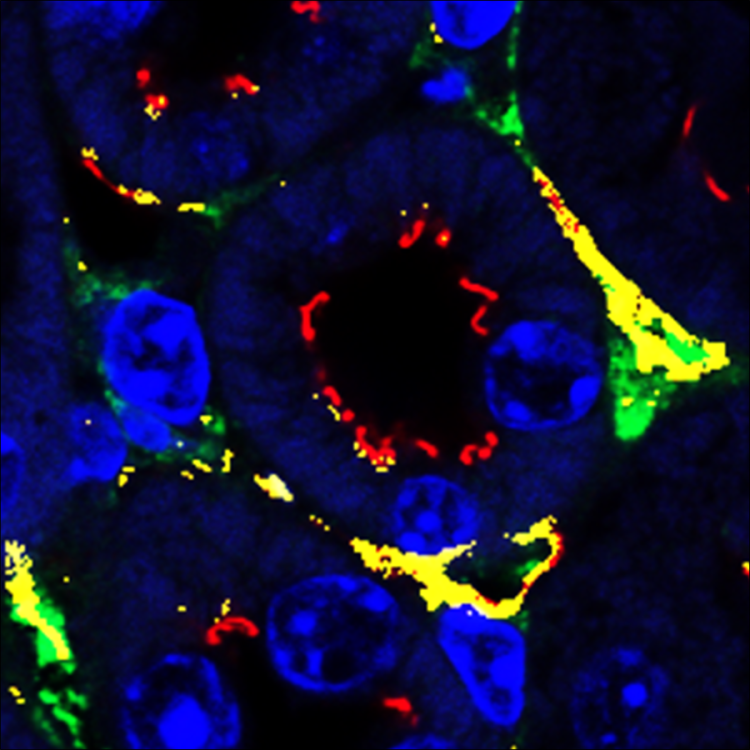

Supplement: Supplementary file 7 — Source data Fig. 5 [file 44321_2025_243_MOESM7_ESM.zip › 5D/Sham cKO.tif]

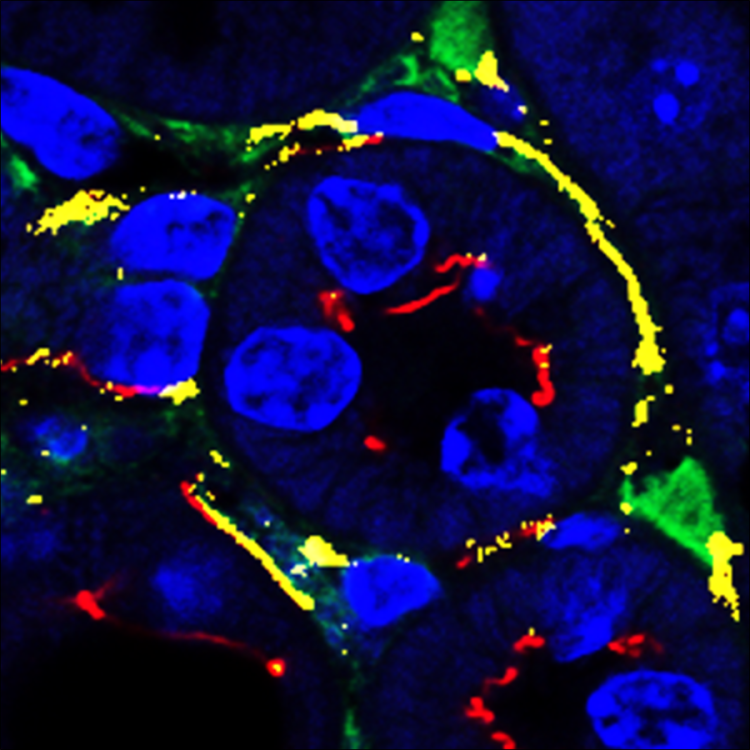

Supplement: Supplementary file 7 — Source data Fig. 5 [file 44321_2025_243_MOESM7_ESM.zip › 5D/Sham WT.tif]

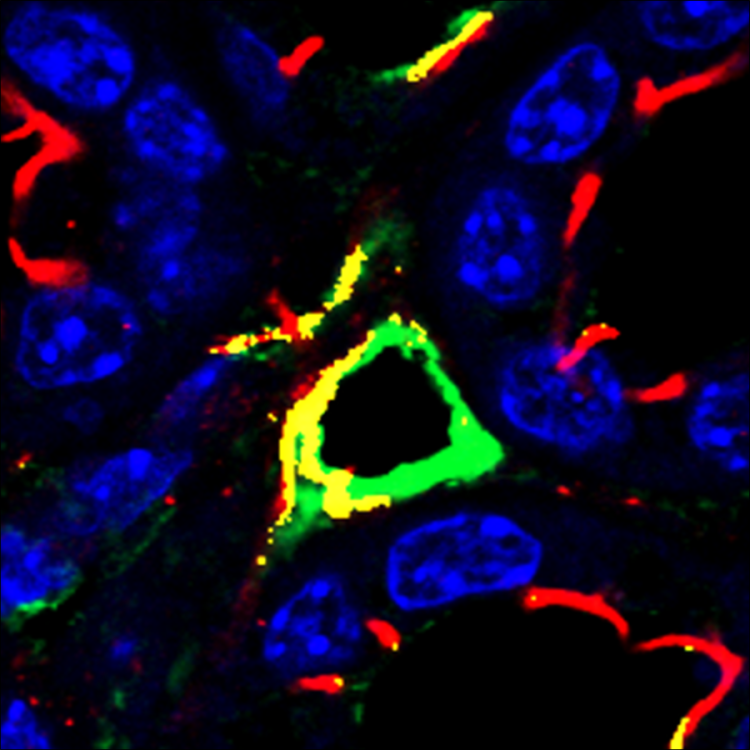

Supplement: Supplementary file 7 — Source data Fig. 5 [file 44321_2025_243_MOESM7_ESM.zip › 5D/UUO cKO.tif]

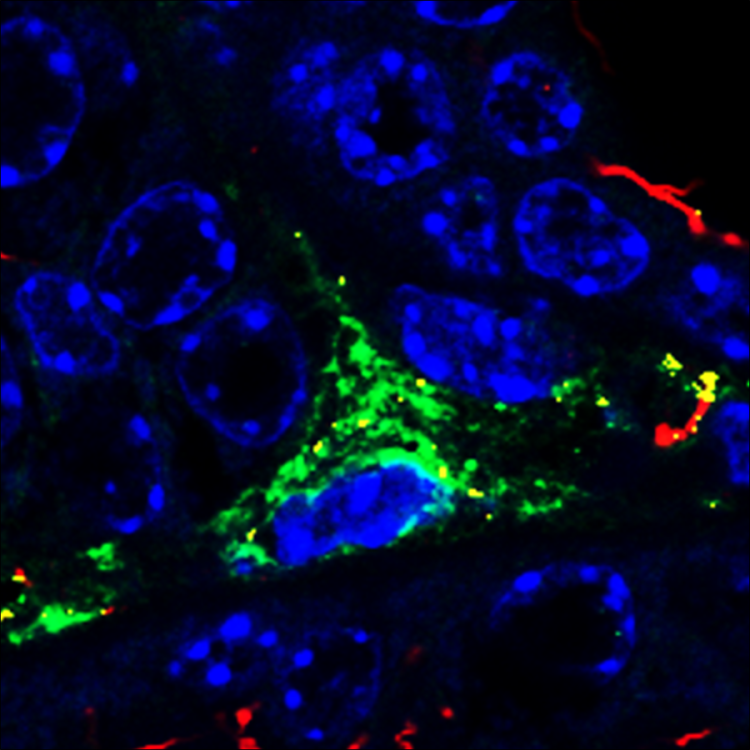

Supplement: Supplementary file 7 — Source data Fig. 5 [file 44321_2025_243_MOESM7_ESM.zip › 5D/UUO wt.tif]

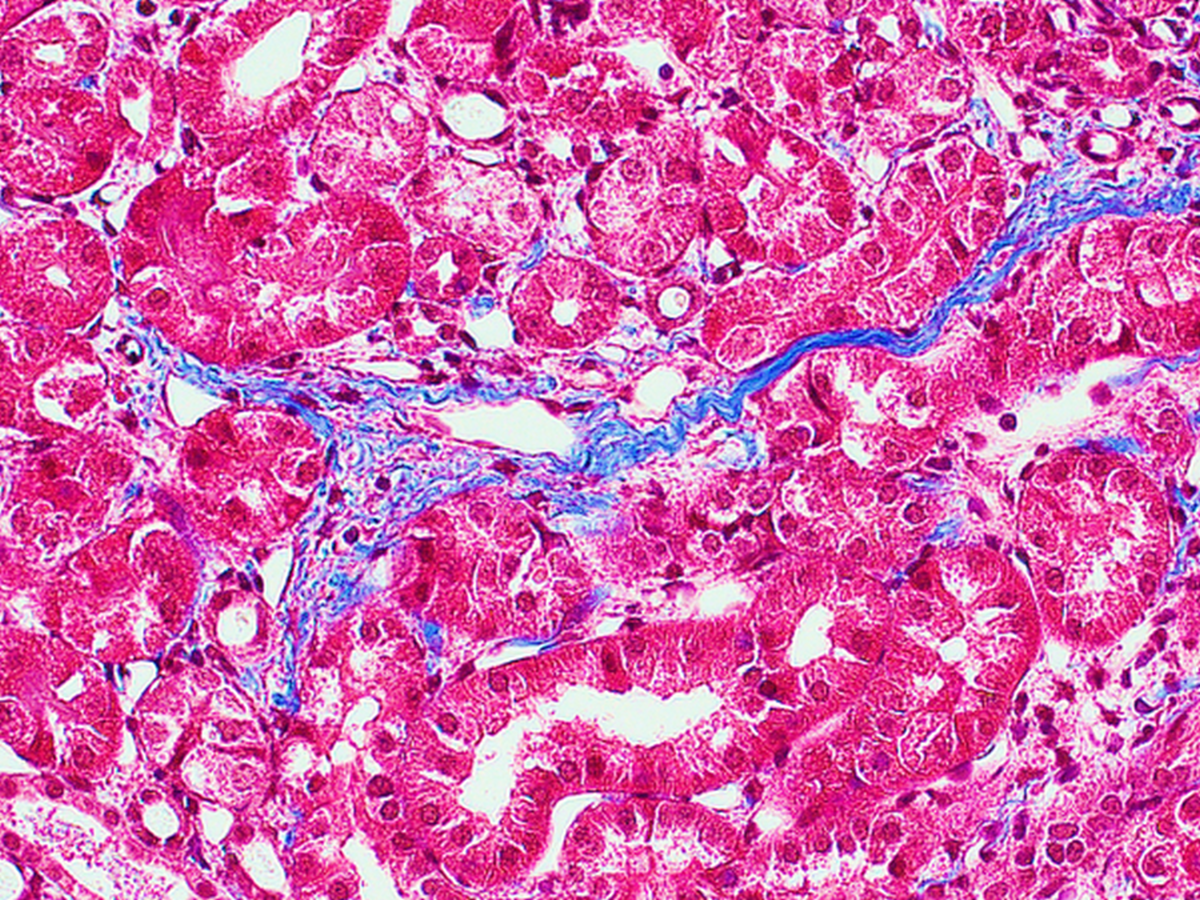

Supplement: Supplementary file 8 — Source data Fig. 6 [file 44321_2025_243_MOESM8_ESM.zip › 6A/MTS C646.tif]

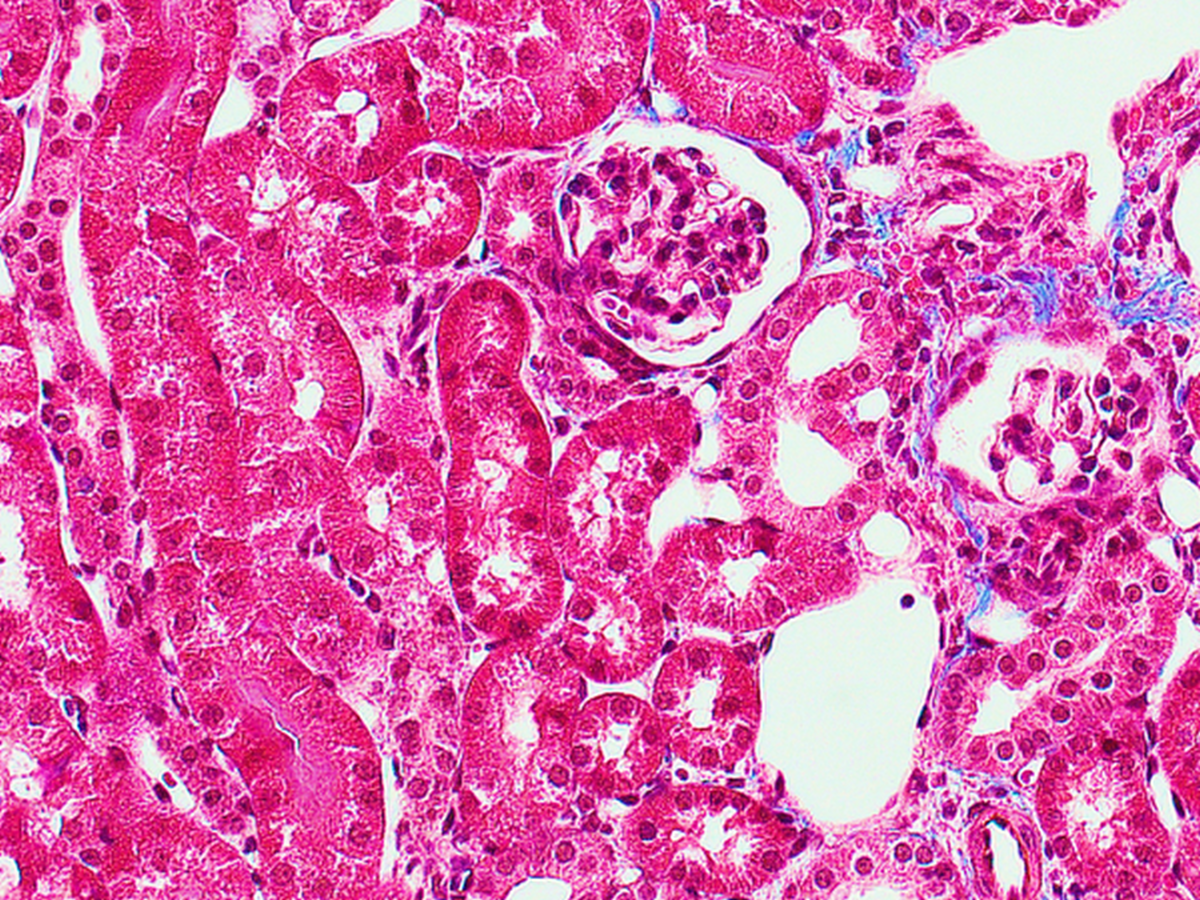

Supplement: Supplementary file 8 — Source data Fig. 6 [file 44321_2025_243_MOESM8_ESM.zip › 6A/MTS Sham.tif]

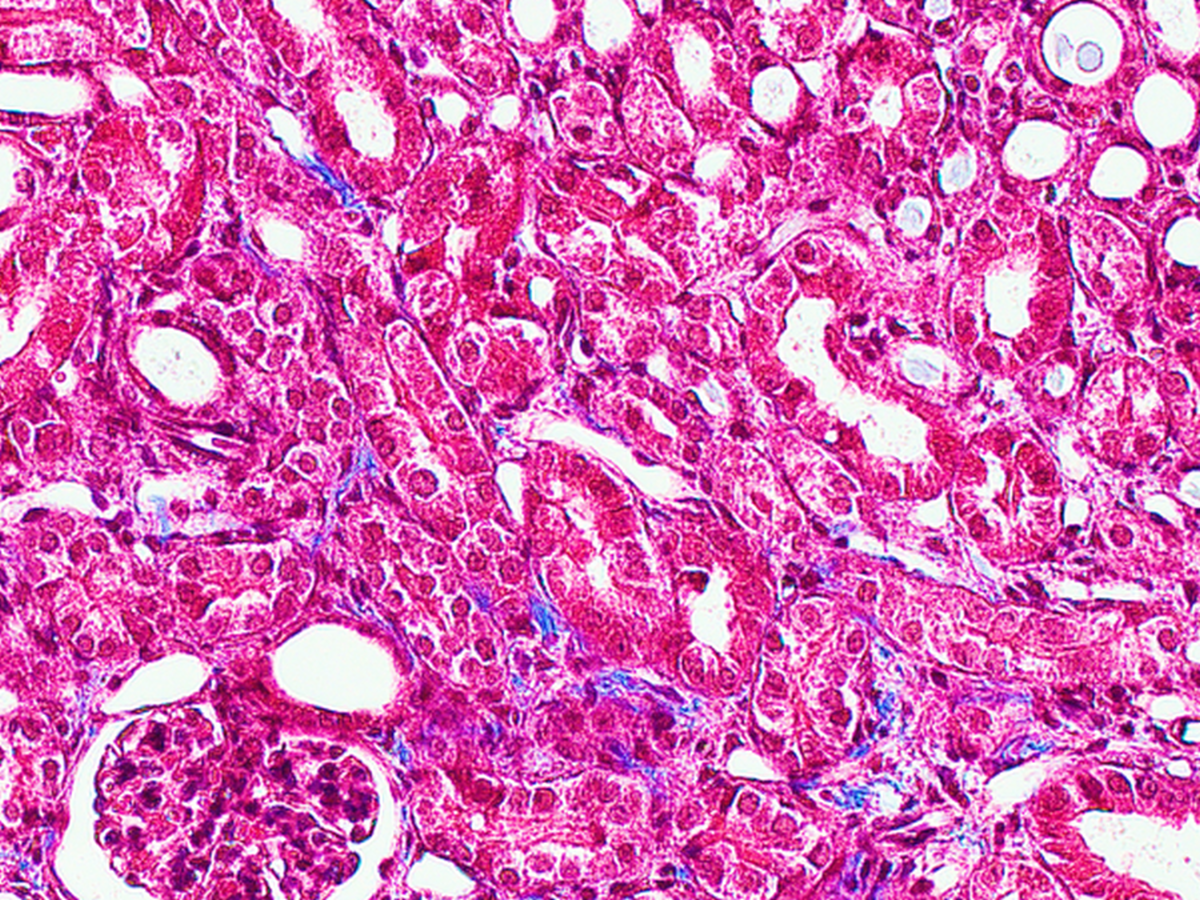

Supplement: Supplementary file 8 — Source data Fig. 6 [file 44321_2025_243_MOESM8_ESM.zip › 6A/MTS UUO A6.tif]

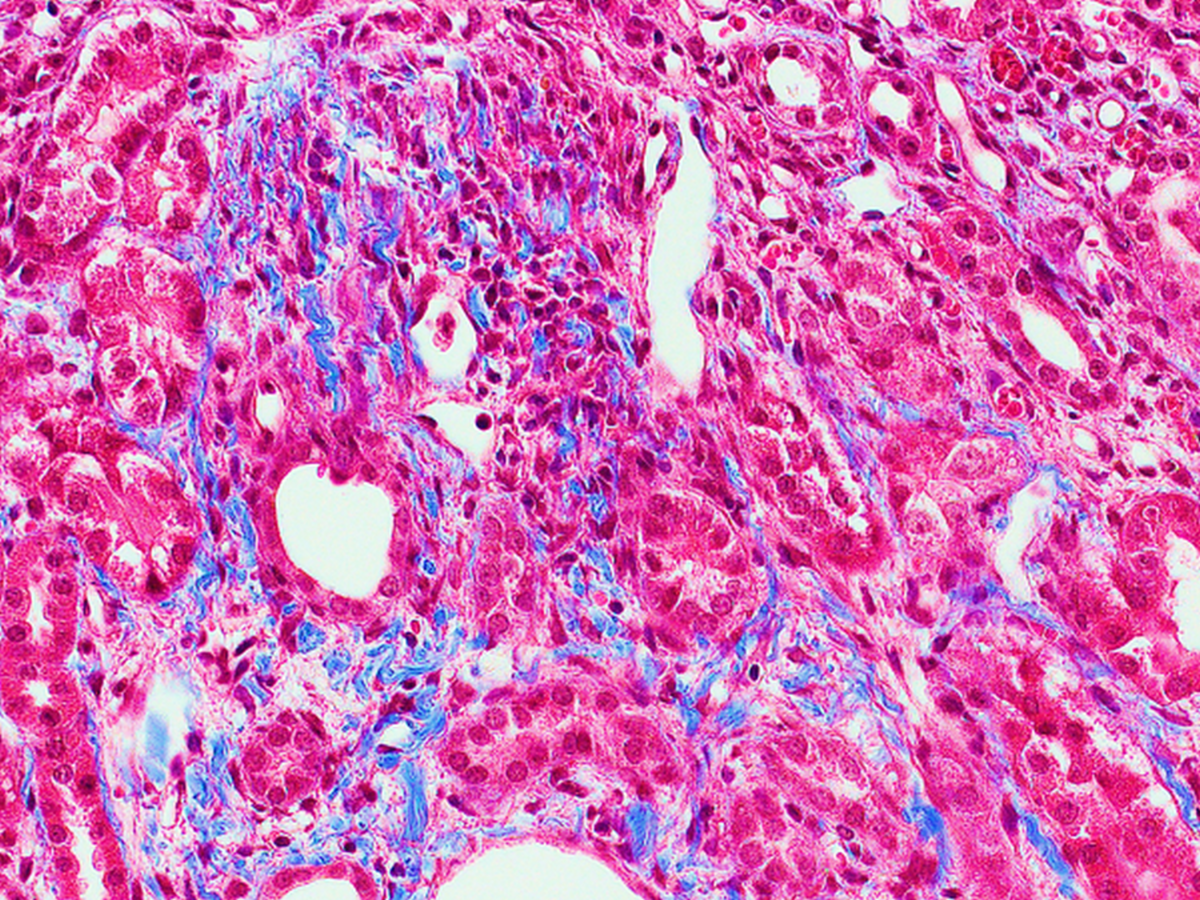

Supplement: Supplementary file 8 — Source data Fig. 6 [file 44321_2025_243_MOESM8_ESM.zip › 6A/MTS UUO.tif]

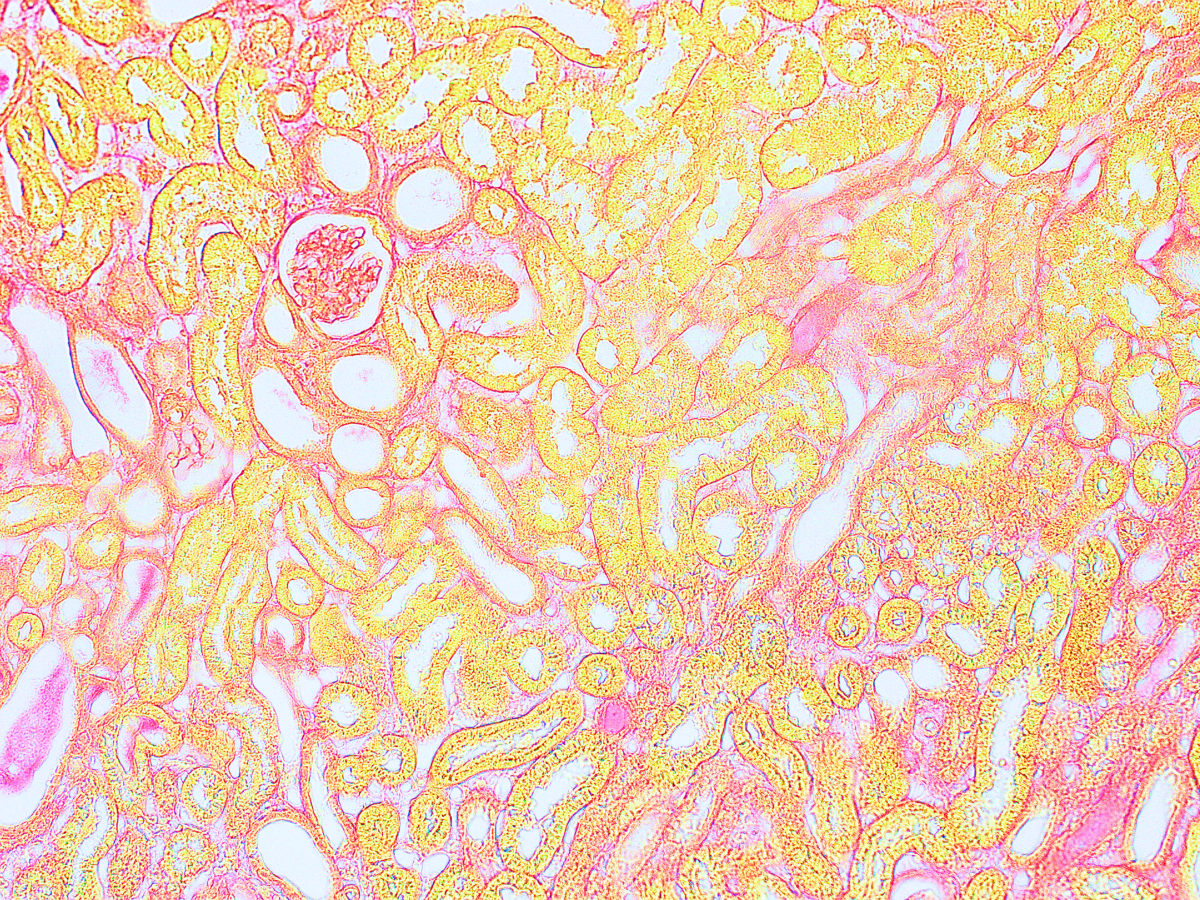

Supplement: Supplementary file 8 — Source data Fig. 6 [file 44321_2025_243_MOESM8_ESM.zip › 6A/Sirius red A6.tif]

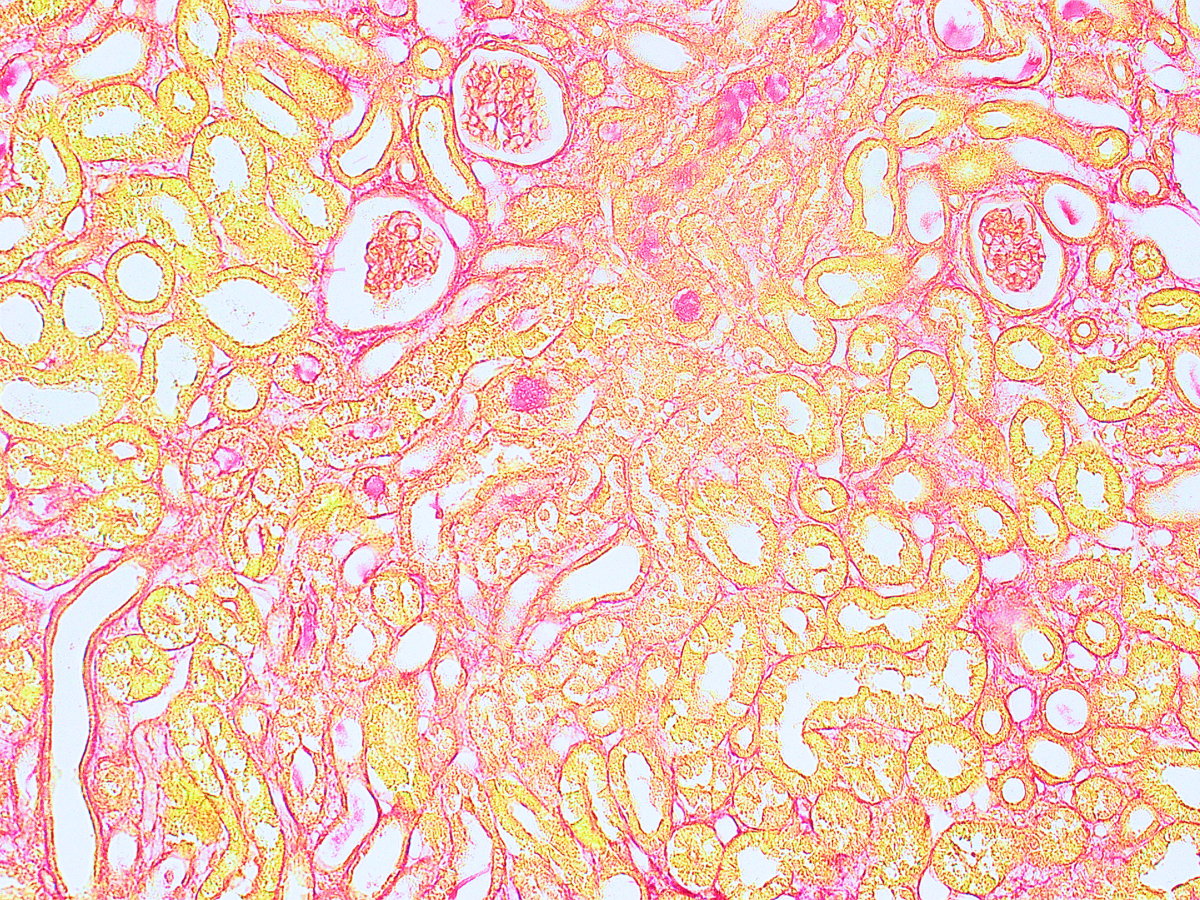

Supplement: Supplementary file 8 — Source data Fig. 6 [file 44321_2025_243_MOESM8_ESM.zip › 6A/Sirius red C646.tif]

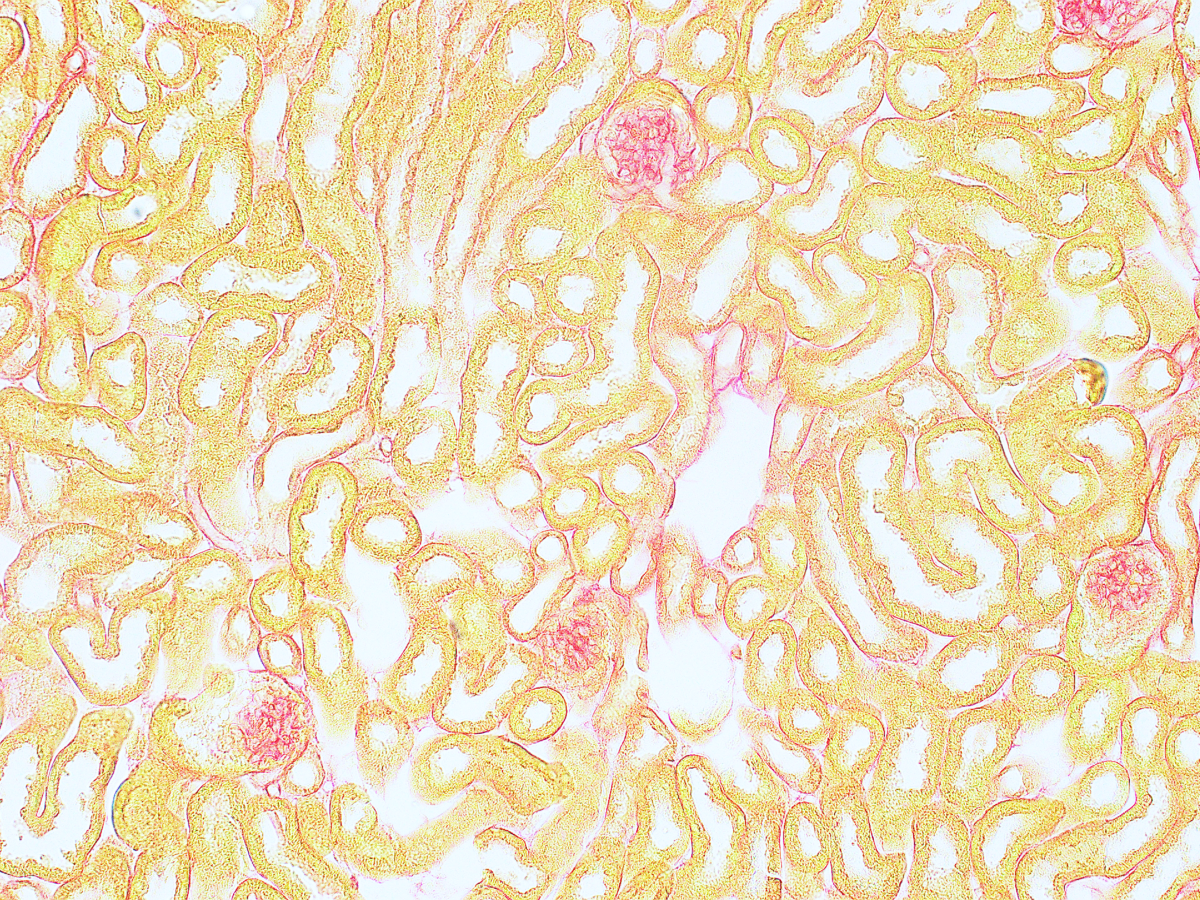

Supplement: Supplementary file 8 — Source data Fig. 6 [file 44321_2025_243_MOESM8_ESM.zip › 6A/Sirius red sham.tif]

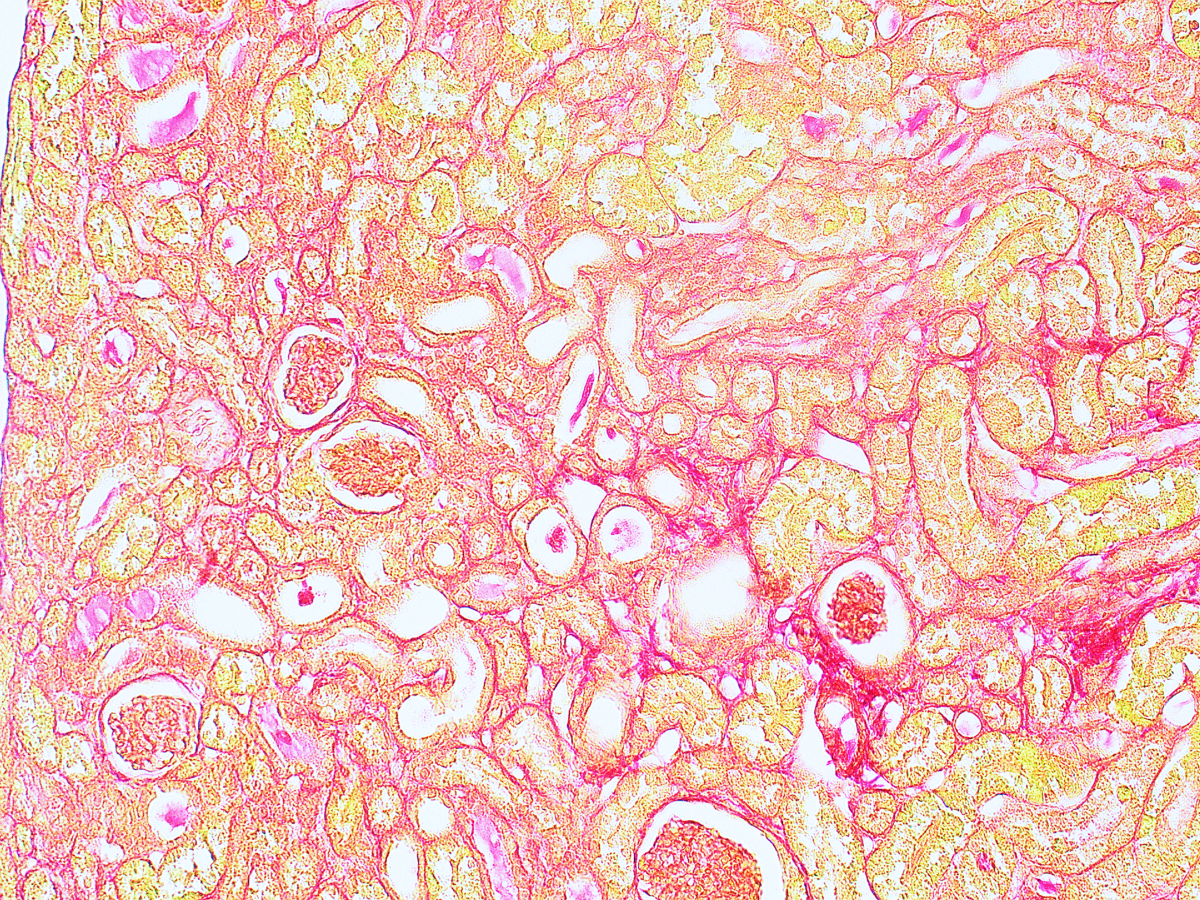

Supplement: Supplementary file 8 — Source data Fig. 6 [file 44321_2025_243_MOESM8_ESM.zip › 6A/Sirius red UUO.tif]

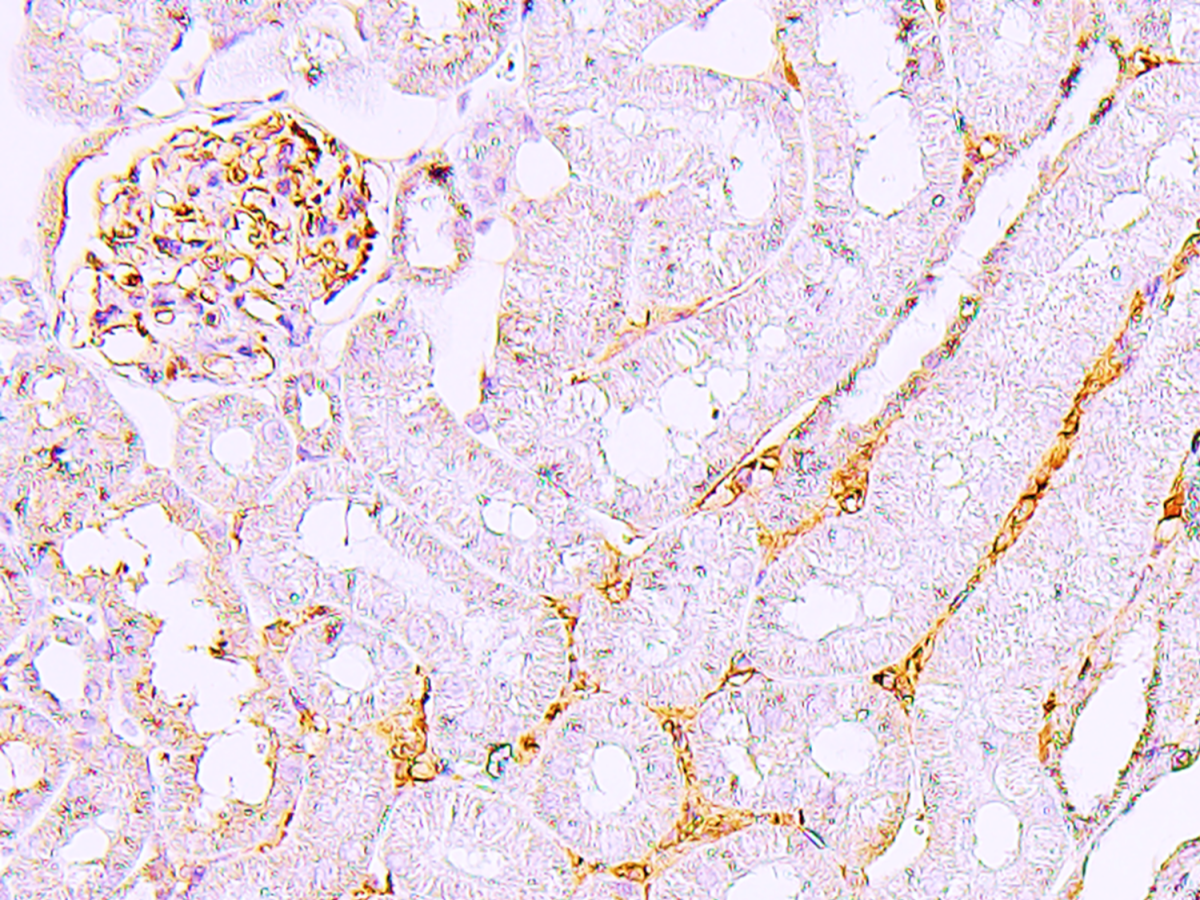

Supplement: Supplementary file 8 — Source data Fig. 6 [file 44321_2025_243_MOESM8_ESM.zip › 6D/Albumin Sham.tif]

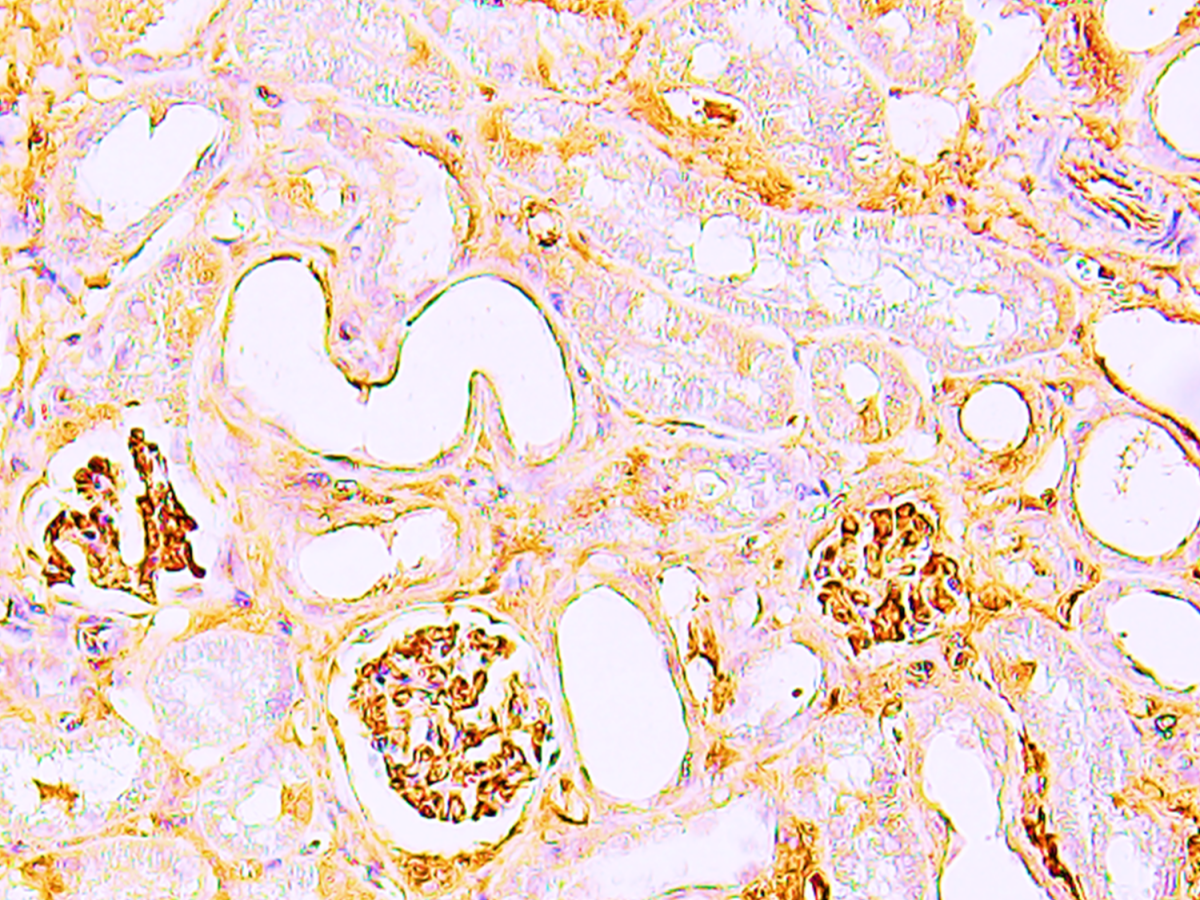

Supplement: Supplementary file 8 — Source data Fig. 6 [file 44321_2025_243_MOESM8_ESM.zip › 6D/Albumin UUO+A6.tif]

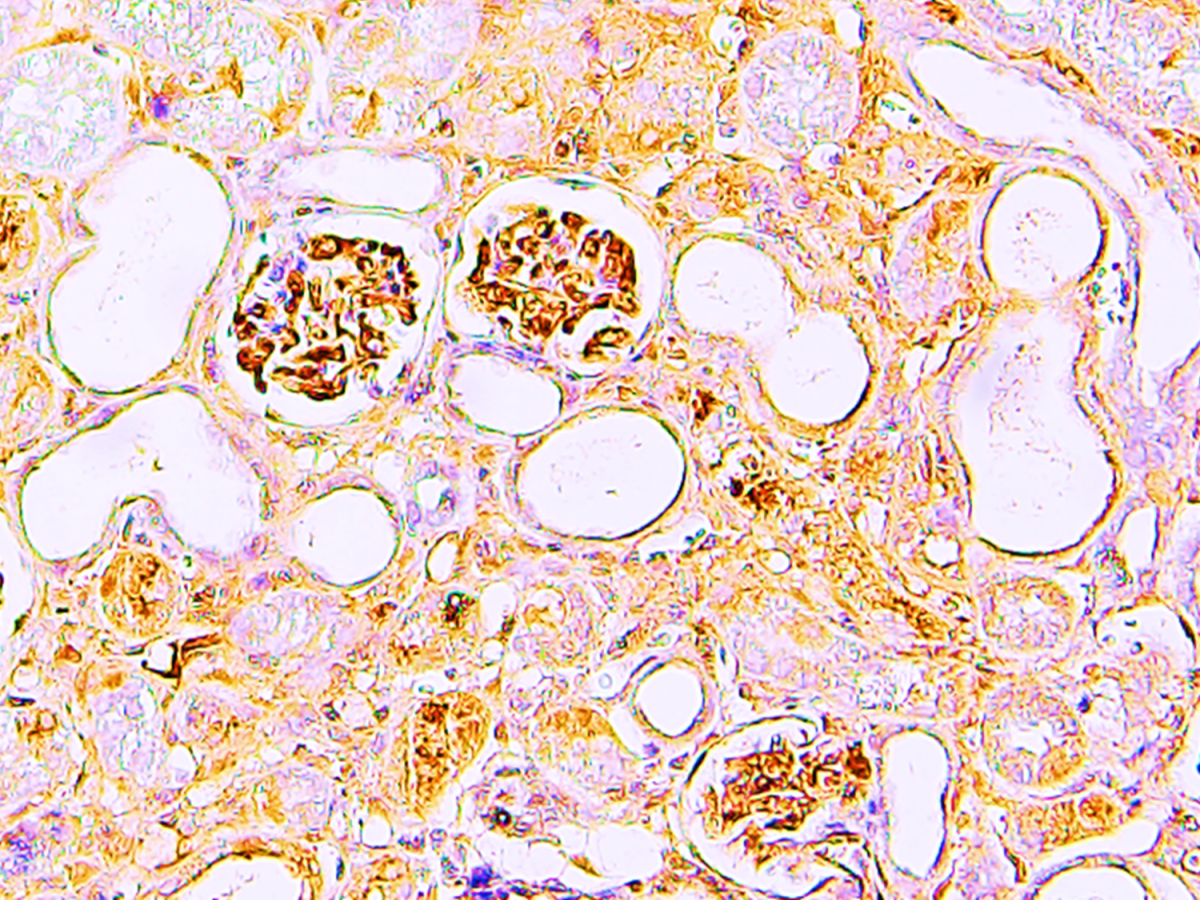

Supplement: Supplementary file 8 — Source data Fig. 6 [file 44321_2025_243_MOESM8_ESM.zip › 6D/Albumin UUO+C646.tif]

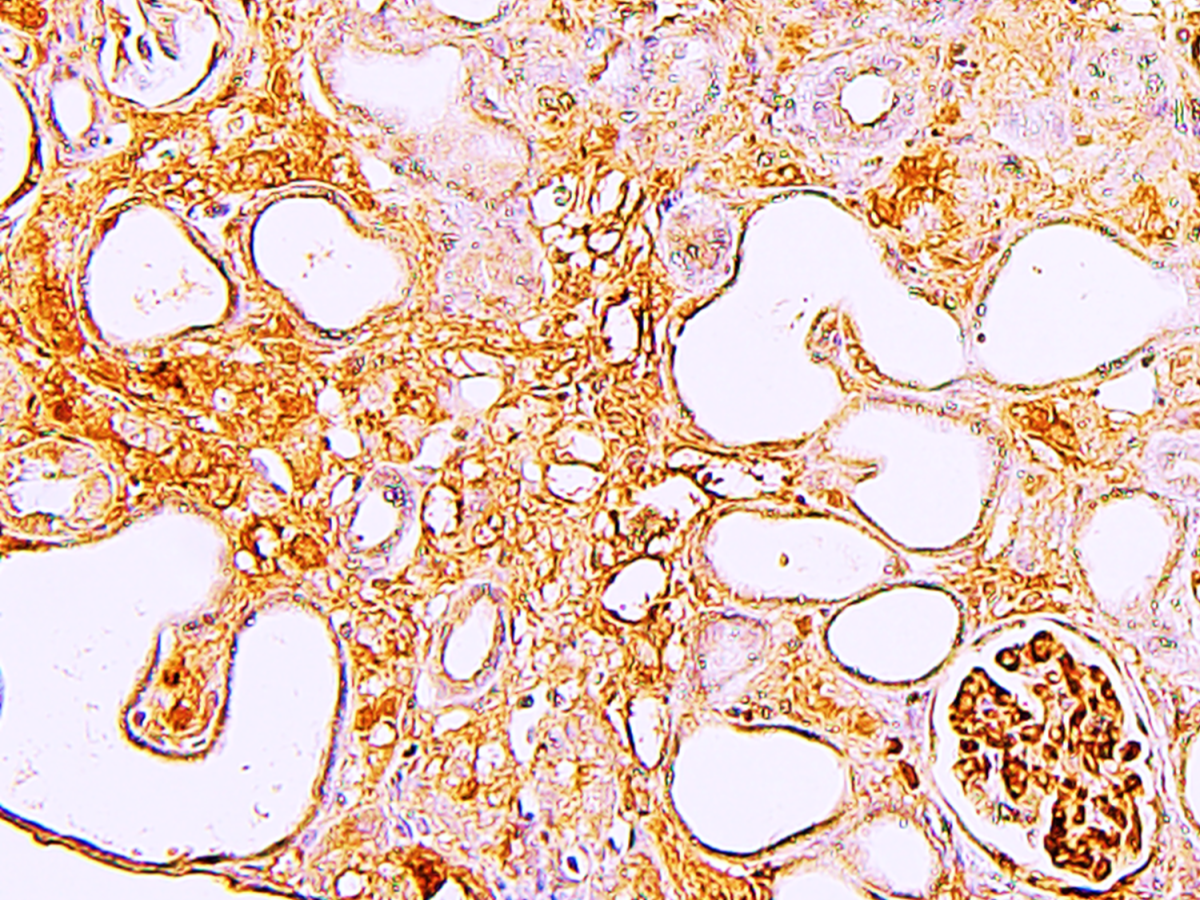

Supplement: Supplementary file 8 — Source data Fig. 6 [file 44321_2025_243_MOESM8_ESM.zip › 6D/Albumin UUO.tif]

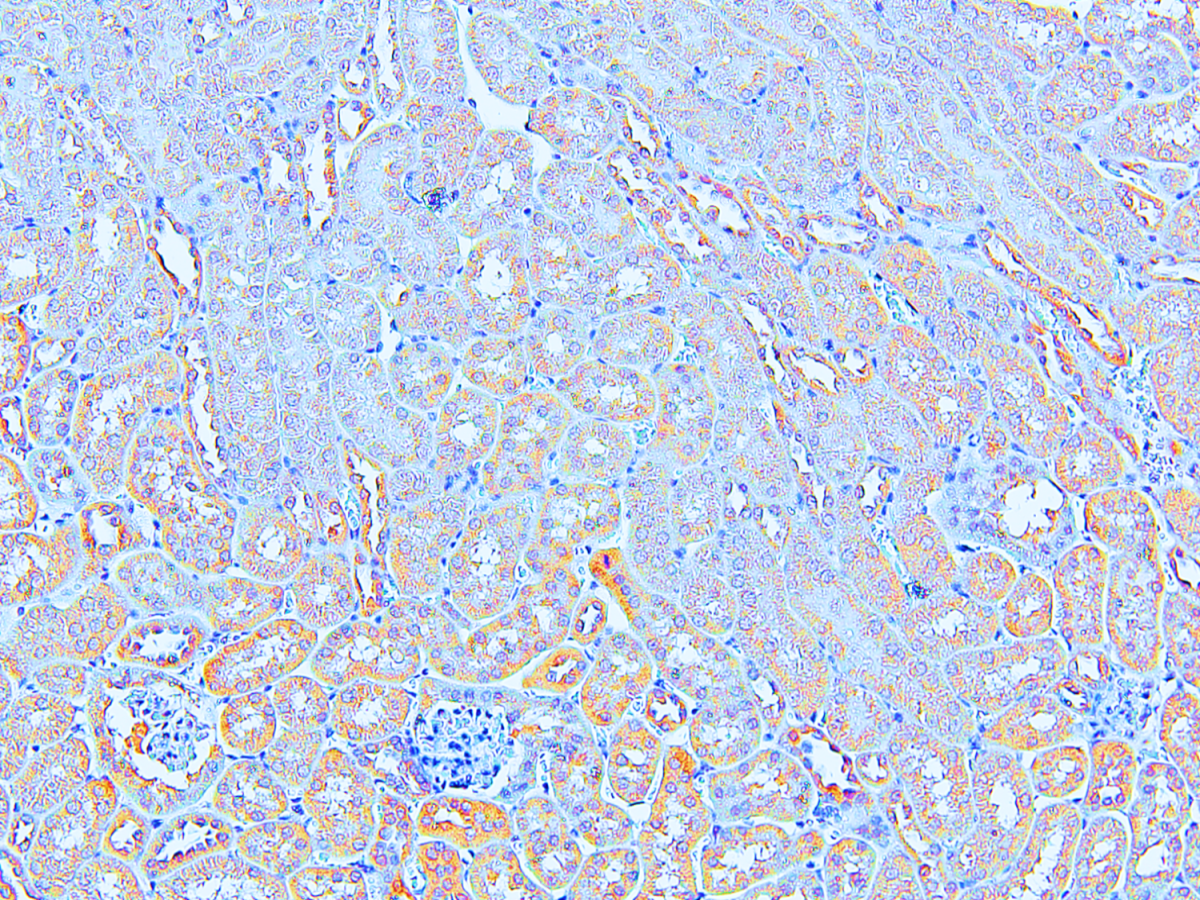

Supplement: Supplementary file 8 — Source data Fig. 6 [file 44321_2025_243_MOESM8_ESM.zip › 6D/KIM1 Sham.tif]

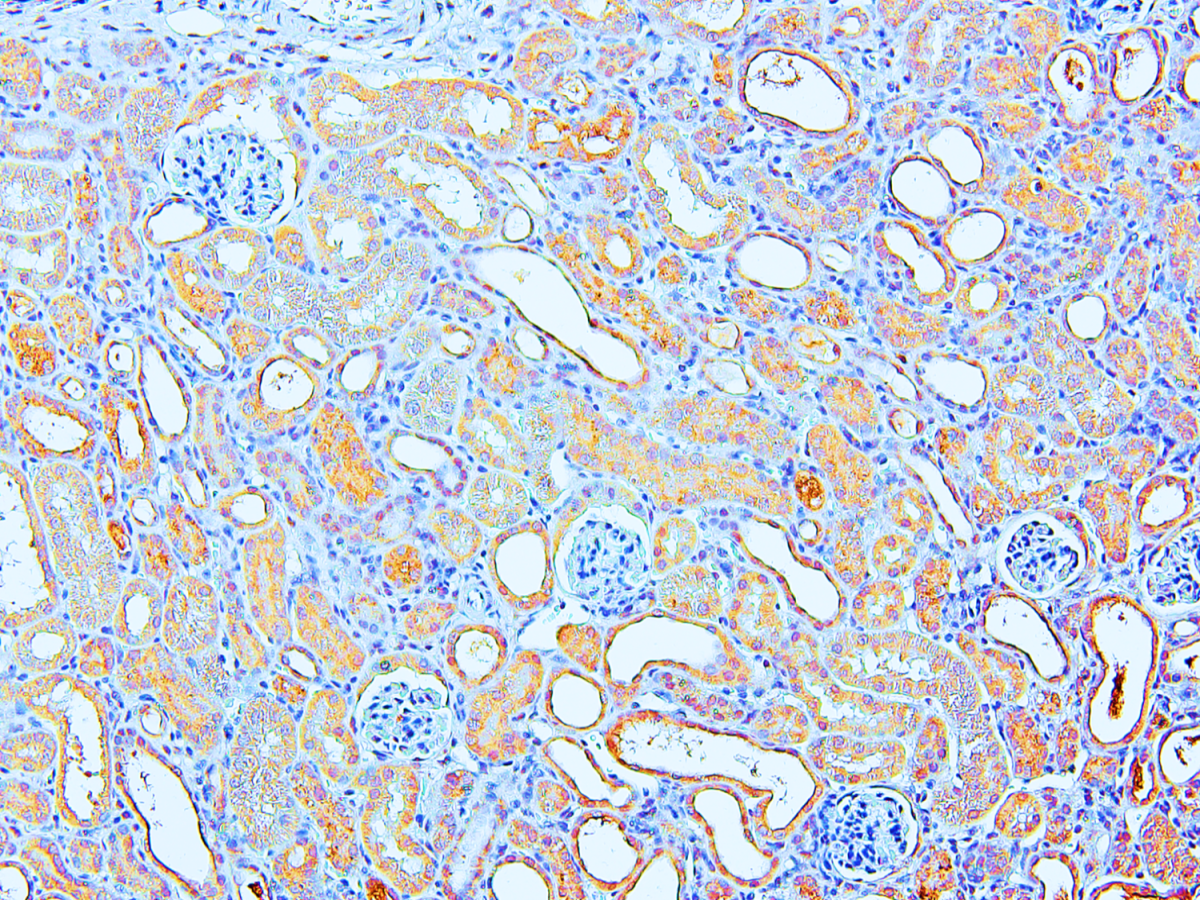

Supplement: Supplementary file 8 — Source data Fig. 6 [file 44321_2025_243_MOESM8_ESM.zip › 6D/KIM1 UUO+A6.tif]

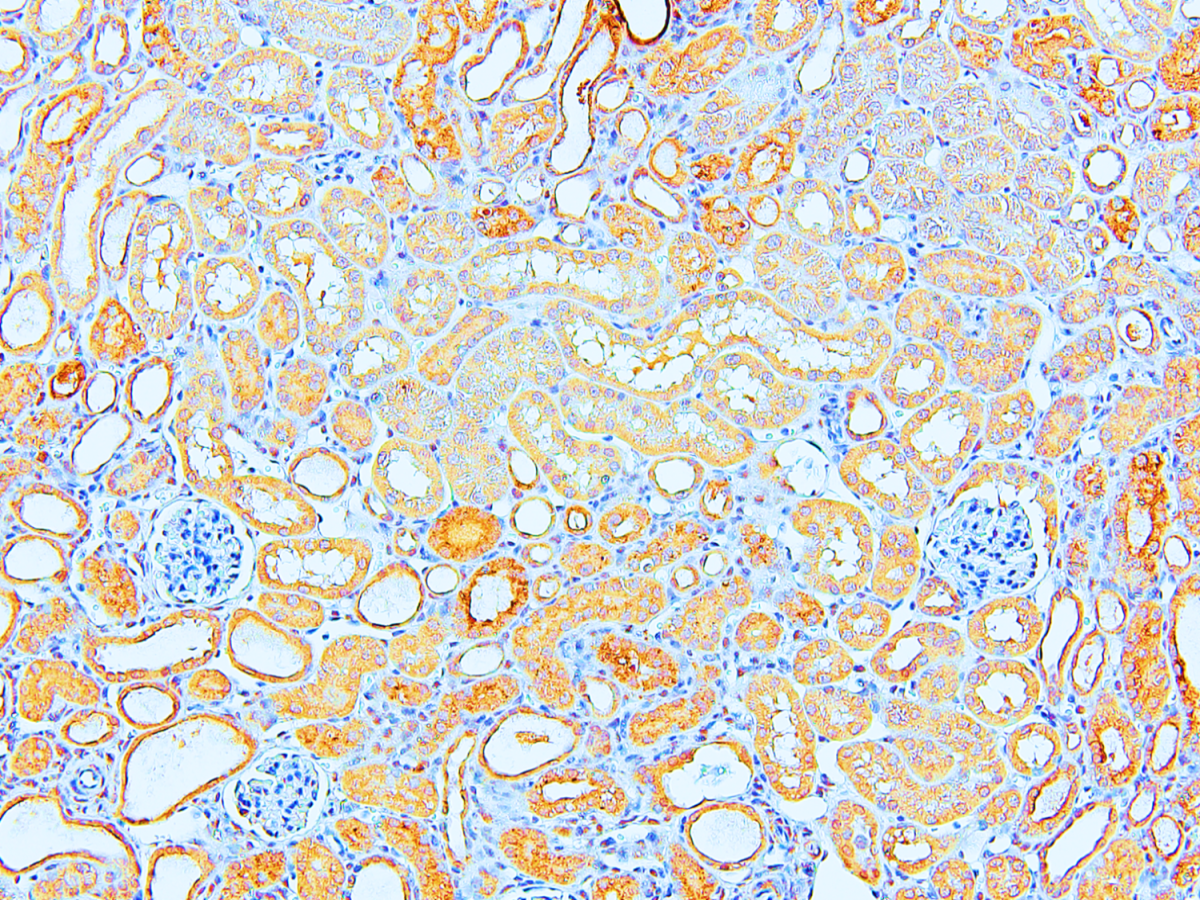

Supplement: Supplementary file 8 — Source data Fig. 6 [file 44321_2025_243_MOESM8_ESM.zip › 6D/KIM1 UUO+C646.tif]

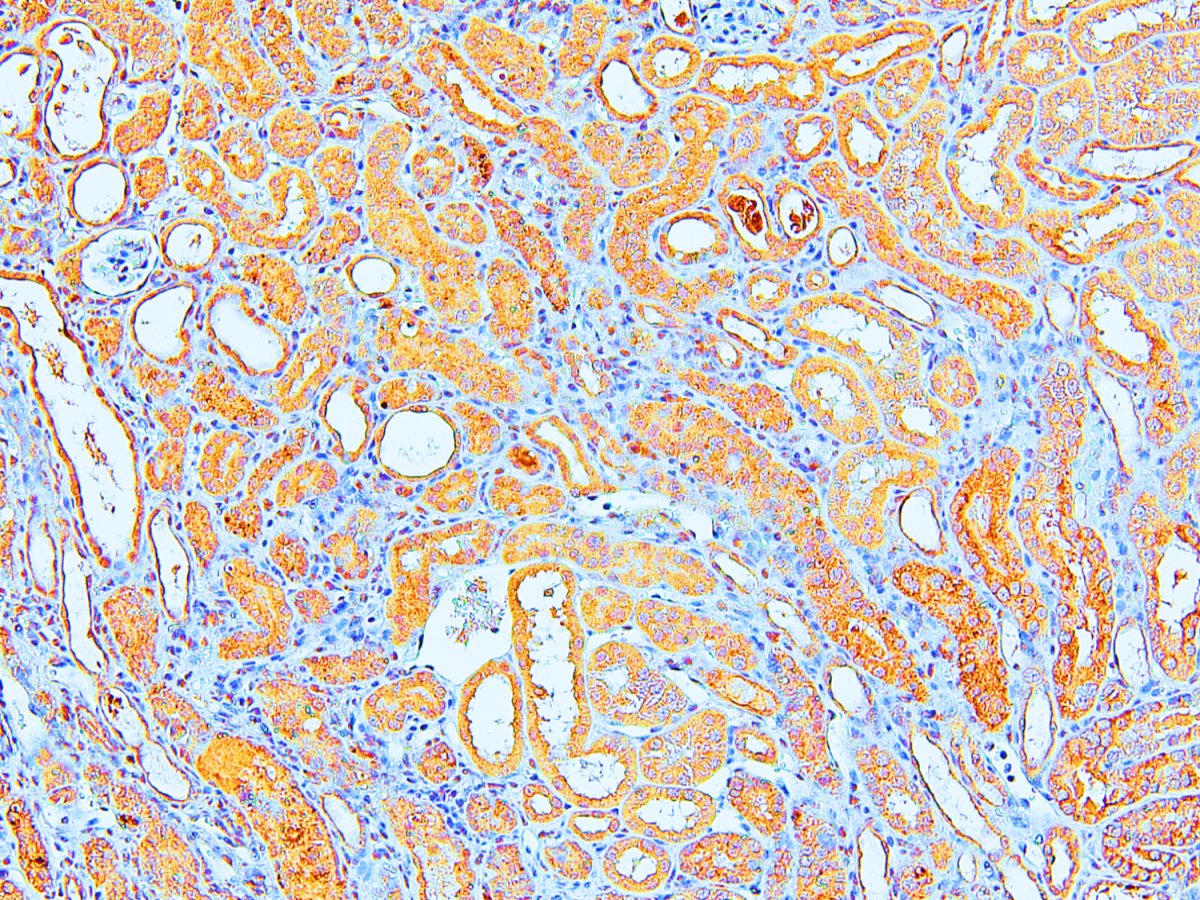

Supplement: Supplementary file 8 — Source data Fig. 6 [file 44321_2025_243_MOESM8_ESM.zip › 6D/KIM1 UUO.tif]
