# Supplementary material for: Loss of p300 in proximal tubular cells reduces renal fibrosis and endothelial-mesenchymal transition
Source: EMBO Mol Med. 2025 Jul 1;17(7):1575–98. doi: 10.1038/s44321-025-00243-1 (PMC12254316; doi:10.1038/s44321-025-00243-1)
Supplement: Supplementary file 10 — Expanded View Figures [file 44321_2025_243_MOESM10_ESM.pdf]

Expanded View Figures

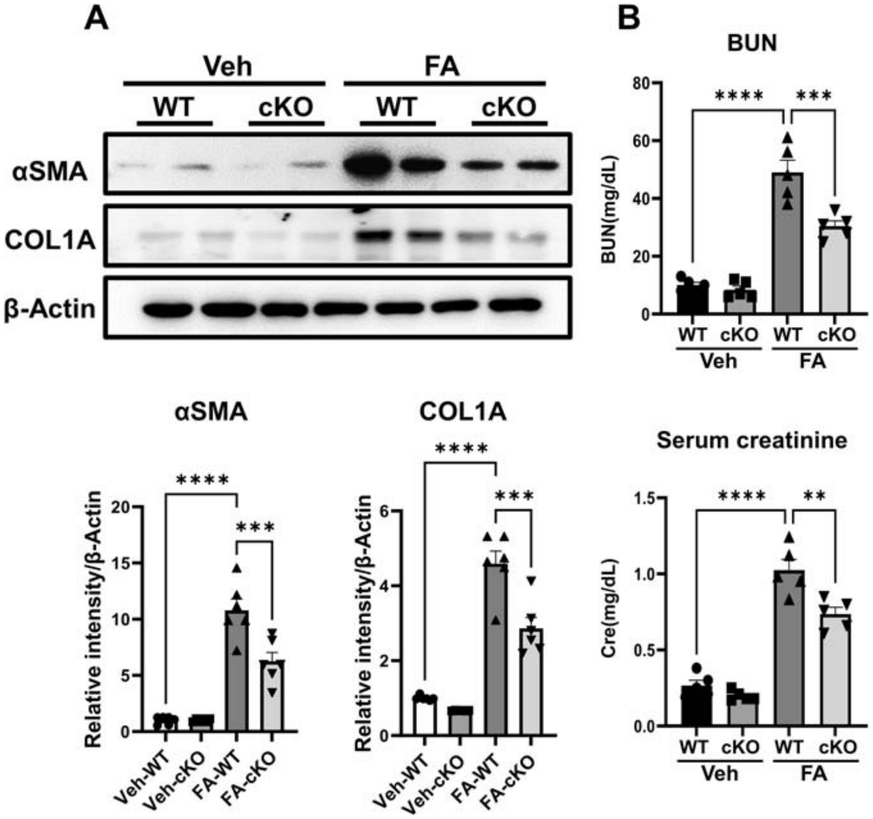

Figure EV1. Evaluation of fibrosis-related proteins and renal function in p300 cKO FA-induced fibrosis mouse models.

(**A**) Protein levels of fibrosis markers, αSMA and COL1A, in kidney tissues from wild-type and p300 knock-out (cKO) FA-induced fibrosis mouse models. β-Actin was used as the loading control. The graph represents the quantification of intensity in western blot images, normalized to β-Actin ($n = 6$ per group). αSMA: Veh-WT vs FA-WT, $P < 0.0001$; FA-WT vs FA-cKO, $P = 0.0004$. COL1A: Veh-WT vs FA-WT, $P < 0.0001$; FA-WT vs FA-cKO, $P = 0.0001$. (**B**) Renal function was assessed using serum samples from wild-type and p300 knock-out (cKO) FA-induced fibrosis mouse models ($n = 5$ per group). BUN: Veh-WT vs FA-WT, $P < 0.0001$; FA-WT vs FA-cKO, $P = 0.0003$. Serum creatinine: Veh-WT vs FA-WT, $P < 0.0001$; FA-WT vs FA-cKO, $P = 0.0021$. Data are presented as mean ± SEM, $^{**}P < 0.01$, $^{***}P < 0.001$, and $^{****}P < 0.0001$ by ordinary one-way ANOVA. Source data are available online for this figure.

 © The Author(s)

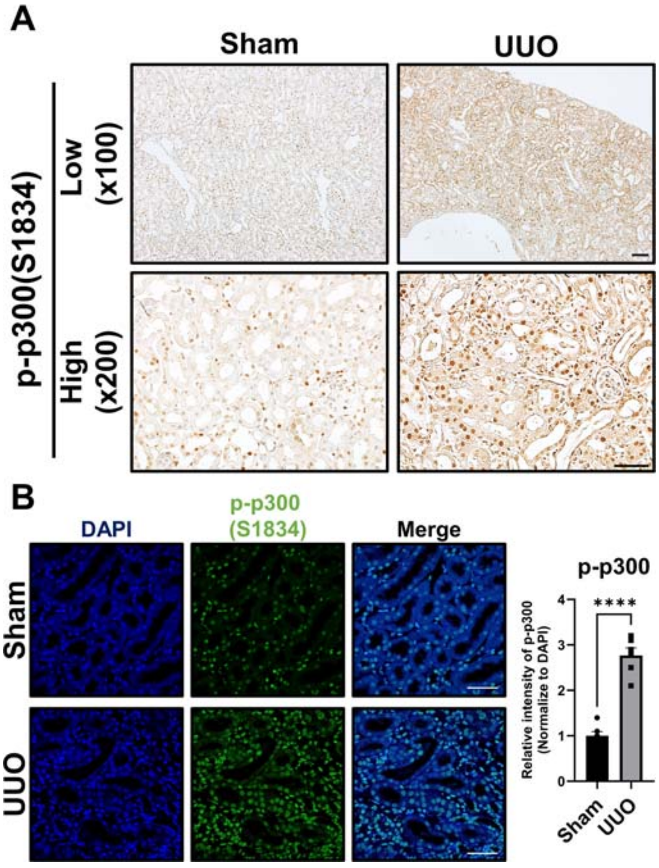

Figure EV2. Increase of p300 phosphorylation at Serine 1834 in kidney tissue from the UUO-induced mouse fibrosis model.

(**A**) Representative image of phosphorylated p300 immunohistochemistry (IHC) in mouse kidney tissue from the UUO-induced mouse fibrosis model. Bar = 100 µm. (**B**) Representative image of phosphorylated p300 immunofluorescence (IF) in mouse kidney tissue from the UUO-induced mouse fibrosis model. The graph represents the quantification of intensity of p-p300 S1834, normalized to DAPI ($n = 6$ per group). Sham vs UUO, $P < 0.0001$. Bar = 100 µm. Data are presented as mean ± SEM, ****$P < 0.0001$ by t-test. Source data are available online for this figure.

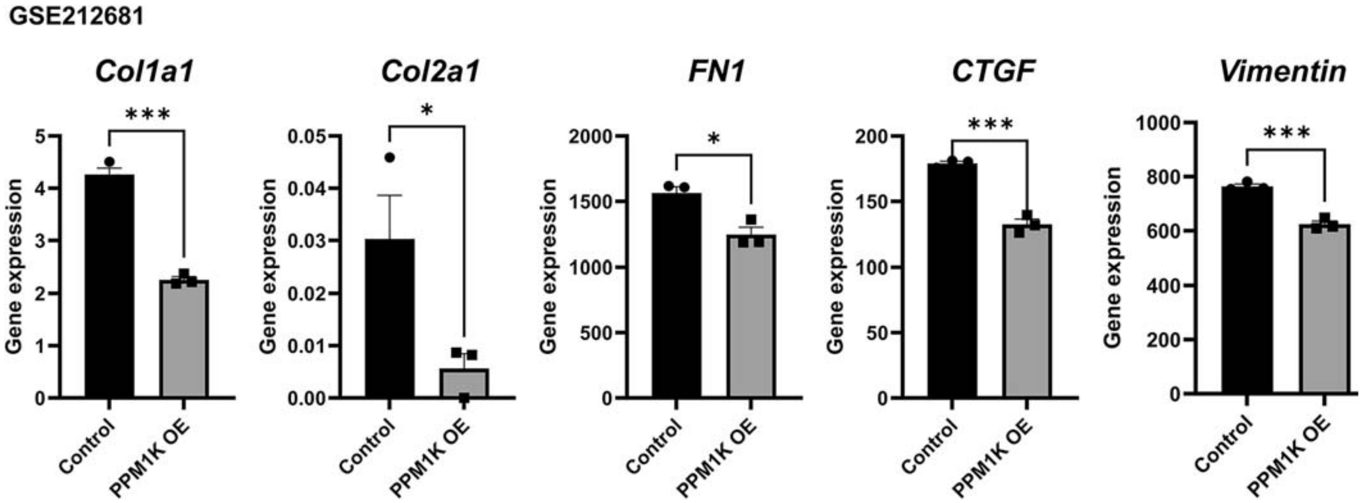

Figure EV3. Analysis of fibrosis-related gene expression in PPM1K-overexpressing HK2 cells.

mRNA levels of fibrosis-related genes in PPM1K-overexpressing HK2 cells. RNA-sequencing data retrieved from the Gene Expression Omnibus (GEO) database (GSE212681). *Col1a1*: $P = 0.0001$. *Col2a1*: $P = 0.0492$. *FN1*: $P = 0.0133$. *CTGF*: $P = 0.0004$. *Vimentin*, $P = 0.0007$. Data are presented as mean ± SEM, $^{*}P < 0.01$, $^{**}P < 0.01$, and $^{***}P < 0.001$ by t-test. Source data are available online for this figure.

 © The Author(s)

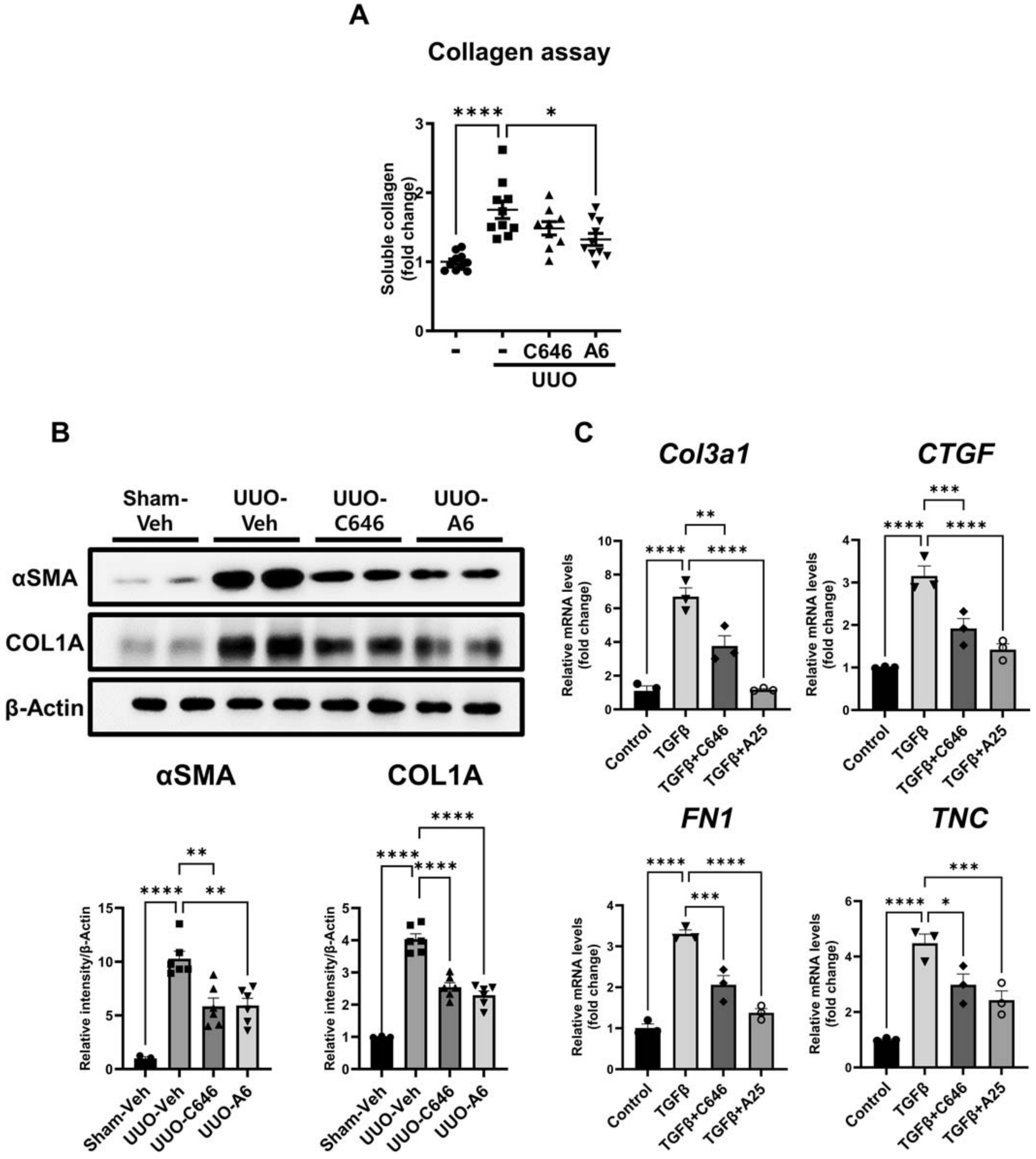
A
Collagen assay
Soluble collagen (fold change)
- - C646 A6
UUO
B
Sham-Veh UUO-Veh UUO-C646 UUO-A6
αSMA
COL1A
β-Actin
αSMA
COL1A
Relative intensity/β-Actin
Sham-Veh UUO-Veh UUO-C646 UUO-A6
C
Col3a1
CTGF
FN1
TNC
Relative mRNA levels (fold change)
Control TGFβ TGFβ+C646 TGFβ+A25

Figure EV4. Inhibition of p300 suppresses the expression of fibrosis-related marker.

(**A**) Soluble collagen assay using kidney samples from UUO-induced fibrosis mice injected with C646 and A6 (sham, $n = 10$, UUO+Veh, $n = 10$, UUO + C646, $n = 9$, UUO + A6, $n = 10$). Sham+Veh vs UUO+Veh, $P < 0.0001$; UUO+Veh vs UUO + A6, $P = 0.0114$. (**B**) Protein levels of fibrosis-related markers (αSMA and COL1A) in kidney tissues from the UUO-induced fibrosis mouse model injected with C646 and A6. β-Actin was used as the loading control. The graph represents the quantification of intensity in western blot images, normalized to β-Actin (sham, $n = 3$, UUO+Veh, $n = 6$, UUO + C646, $n = 6$, UUO + A6, $n = 6$). αSMA: Sham+Veh vs UUO+Veh, $P < 0.0001$; UUO+Veh vs UUO + C646, $P = 0.0012$; UUO+Veh vs UUO + A6, $P = 0.0015$. COL1A: Sham+Veh vs UUO+Veh, $P < 0.0001$; UUO+Veh vs UUO + C646, $P < 0.0001$; UUO+Veh vs UUO + A6, $P < 0.0001$. (**C**) mRNA levels of fibrosis-related genes in HK2 cells treated with TGFβ for 24 h and co-treated with C646 and A6 ($n = 3$ per group). *Col3a1*: Control vs TGFβ, $P < 0.0001$; TGFβ vs TGFβ + C646, $P = 0.0011$; TGFβ vs TGFβ + A6, $P < 0.0001$. *CTGF*: Control vs TGFβ, $P < 0.0001$; TGFβ vs TGFβ + C646, $P = 0.0009$; TGFβ vs TGFβ + A6, $P < 0.0001$. *FN1*: Control vs TGFβ, $P < 0.0001$; TGFβ vs TGFβ + C646, $P = 0.0001$; TGFβ vs TGFβ + A6, $P < 0.0001$. *TNC*: Control vs TGFβ, $P < 0.0001$; TGFβ vs TGFβ + C646, $P = 0.0116$; TGFβ vs TGFβ + A6, $P = 0.0009$. Data are presented as mean ± SEM, $^{*}P < 0.05$, $^{**}P < 0.01$, $^{***}P < 0.001$, and $^{****}P < 0.0001$ by ordinary one-way ANOVA test. Source data are available online for this figure.

 © The Author(s)
